# Bacterial SET domain proteins and their role in eukaryotic chromatin modification

**DOI:** 10.3389/fgene.2014.00065

**Published:** 2014-04-02

**Authors:** Raúl Alvarez-Venegas

**Affiliations:** Laboratory of Chromatin and Epigenetics, Department of Genetic Engineering, CINVESTAV Unidad-IrapuatoIrapuato, México

**Keywords:** SET-domain, bacteria, pathogen, symbiont, histone methyltransferases, epigenetics

## Abstract

It has been shown by many researchers that SET-domain containing proteins modify chromatin structure and, as expected, genes coding for SET-domain containing proteins have been found in all eukaryotic genomes sequenced to date. However, during the last years, a great number of bacterial genomes have been sequenced and an important number of putative genes involved in histone post-translational modifications (histone PTMs) have been identified in many bacterial genomes. Here, I aim at presenting an overview of SET domain genes that have been identified in numbers of bacterial genomes based on similarity to SET domains of eukaryotic histone methyltransferases. I will argue in favor of the hypothesis that SET domain genes found in extant bacteria are of bacterial origin. Then, I will focus on the available information on pathogen and symbiont SET-domain containing proteins and their targets in eukaryotic organisms, and how such histone methyltransferases allow a pathogen to inhibit transcriptional activation of host defense genes.

## Introduction

In eukaryotic organisms, genomic DNA is packaged in the form of a complex structure known as chromatin. The basic unit of chromatin is the nucleosome, consisting of approximately two superhelical turns of DNA wrapped on a histone octamer composed of four histone species: a histone H3/H4 tetramer and two histone H2A/H2B dimmers (Kornberg, 1977). This nucleoprotein complex occurs basically every 200 ± 40 bp in all eukaryotic genomes. The repeating nucleosome units further assemble into higher-order structures stabilized by the linker histone H1.

Core histones are predominantly globular except for their N-terminal “tails,” which are unstructured. A remarkable attribute of histones, and mainly of their tails, is the great number and type of modified residues they possess (Kouzarides, 2007). Thus, there are several histone post-translational modifications (histone PTMs) that correlate with either positive or negative transcriptional states. These modifications include acetylation, phosphorylation, methylation, ubiquitylation, and SUMOylation. Most modifications localize to the amino- and carboxy-terminal histone tails, and a few localize to the histone globular domains (Berger, 2007). It has been suggested that the combination of histone amino-terminal modifications on one or more histones represent a “histone code” that modulates gene expression, regulates chromatin structure, and determines cellular and epigenetic identities during development, therefore extending the information potential of the genetic code encoded in the DNA (Jenuwein and Allis, 2001; Casadio et al., 2013). Recently, with the discovery of several novel histone-binding modules the histone-code hypothesis predicts the existence of “reader proteins” that recognize chromatin covalent-modification marks to influence downstream events (Ruthenburg et al., 2007). On the other hand, all histone PTMs are removable. For example, histone deacetylases (HDACs) remove acetyl groups; Ser/Thr phosphatases remove phosphate groups; ubiquitin proteases remove mono-ubiquitin from H2B; arginine methylation is altered by deiminases; and two classes of lysine demethylases remove methyl groups from lysines: the LSD1/BHC110 class and the jumonji class (Berger, 2007).

One of the most extensively studied histone PTMs has been the methylation of lysine residues in histones. Accordingly, the first histone lysine methyltransferase (HKMT) to be identified was the human and mouse SUV39H1 that targets lysine 9 of histone H3 (H3K9) (Rea et al., 2000). Since then, numerous HKMTs have been identified, most of which methylate lysines within the histone N-terminal tails. Noticeably, all of the HKMTs that methylate N-terminal lysines contain the SET domain, a ~130 amino acid catalytic domain initially found to be conserved in the PEV modifier SU(VAR)3-9, the Polycomb-group protein E(Z), and the trithorax-group protein TRX (Jenuwein et al., 1998). Crystal structures of SET-domain proteins have revealed that the SET domain is folded into several small β sheets (packed together with pre-SET and post-SET domains or regions) surrounding a knot-like structure. This “pseudo-knot” fold brings together the two most-conserved sequence motifs of the SET domain (RFINHXCXPN and ELXFDY) to form an active site in a location immediately next to the pocket where the methyl donor binds and to the peptide-binding cleft (Dillon et al., 2005). Moreover, it has also been shown that both N- and C-terminal flanking regions to the SET-domain are as well required for HKMT activity (Rea et al., 2000).

Histone lysine residues methylated *in vivo*, in animals, include H3K4, H3K9, H3K27, H3K36, H3K79, H4K20, H2BK5, and H1K26 (Barski et al., 2007). The first H3K4 (histone H3 lysine 4) methylase, Set1/COMPASS, was isolated from *Saccharomyces cerevisiae* and was demonstrated to be capable of mono-, di-, and tri-methylate H3K4 (Miller et al., 2001; Roguev et al., 2001). Thus, methylation can occur several times on one lysine side chain and each level of modification may have different biological outcomes. Furthermore, of the large family of SET domains, a subset is encoded by bacterial pathogens and symbionts, which lack chromatin, suggesting a role in altering the host chromatin upon infection. For instance, pathogenic bacteria make use of a wide range of strategies to avoid elimination by their host. Then, aiming at histone modifications could allow a pathogen to inhibit transcriptional activation of host defense genes. Hence, pathogenic bacteria can be considered as “epimutagens” able to remodel the epigenome. Their effects might generate specific, long-lasting imprints on host cells, leading to a memory of infection that influences immunity and that might be the foundation of unexplained diseases.

Here, I will examine the available information on SET domain genes identified in bacterial genomes. I will analyze the hypothesis that SET domain genes found in bacteria are of bacterial origin. Then, I will concentrate on the information related to pathogen and symbiont SET-domain containing proteins, their targets in eukaryotic organisms, and on how such histone methyltransferases could allow a pathogen to inhibit transcriptional activation of host defense genes.

## Bacterial SET domain

Considering that SET-domain containing proteins modify chromatin structure, as expected, genes coding for SET-domain containing proteins have been found in all eukaryotic genomes sequenced to date. However, during the last years, thanks to the recent advances in biological research such as next-generation sequencing, a great number of bacterial genomes have been sequenced and, what's more, an important number of putative genes involved in histone PTMs have been identified in many bacterial genomes. For example, from 390 completely and partially sequenced bacterial genomes available in 2007 at the National Center for Biotechnology Information database (NCBI), 83 bacterial species encoding putative SET domain proteins were retrieved (Alvarez-Venegas et al., 2007). However, a basic BLASTP search performed today retrieves more than 500 bacterial genomes (and counting; Supplementary Table ST1), including very closely related genomes, all of them containing SET-domain proteins. The number of hits range from one to four SET coding genes per genome. Interestingly, species like *Gemmata obscuriglobus* (a nonpathogenic spherical budding bacteria; NCBI locus WP_010044726), *Bacillus coahuilensis* (a Gram-positive, spore-forming bacterium from a highly saline desert lagoon; NCBI locus WP_010172611), *Opitutus terrae* PB90-1 (an obligatory anaerobic bacteria isolated from rice paddy soil; NCBI locus YP_001821399), *Burkholderia rhizoxinica* HKI 454 (an intracellular symbiont of a phytopathogenic fungus; NCBI locus CBW73645), *Methanoregula boonei* 6A8 (a novel acidiphilic, hydrogenotrophic methanogen; NCBI locus WP_012107610), and many more interesting bacterial species have recently been shown to code for SET-domain containing proteins (Supplementary Table ST1).

At first, proteins with SET domain present in bacterial genomes were considered to be the result of horizontal transfer from a eukaryotic host (Stephens et al., 1998; Aravind and Iyer, 2003). However, nowadays, the expanded collection of sequenced bacterial genomes, to include not only pathogenic but also free-living and environmental species, as well as methanogenic archaea, indicate that SET domain genes have existed in the bacterial domain of life (Alvarez-Venegas et al., 2007). Furthermore, Aravind et al. (2011) have proposed that SET domain methylases, which display the β-clip fold, first emerged in prokaryotes from the SAF superfamily of carbohydrate-binding domains (based on its representative members, SAS, type III AFP and FlgA). Thus, taking into account recent evidence that supports a chromatin-related role for at least a portion of the bacterial SET domain versions, it is likely that the SET domain had matured into a primitive chromatin-remodeling enzyme in prokaryotes, prior to its transfer to eukaryotes (Aravind et al., 2011).

On the other hand, phylogenetic analysis have shown that bacterial SET domain genes have undergone an evolution of their own, unrelated to the evolution of the eukaryotic SET domain genes (Alvarez-Venegas et al., 2007; Murata et al., 2007). This can be substantiated, for example, by performing a BLASTP search at NCBI, with any SET-domain. In all “distance trees” produced using BLAST pair-wise alignments, the Newick dendrograms (as well as any phylogenetic tree) produced at NCBI show all eukaryotic entries clustered as a monophyletic group. Important to our discussion is that eukaryotic SET proteins do not mix together with SET proteins of bacterial origin and that a similar distribution pattern is continuously reproduced with different combinations of prokaryotic or eukaryotic entries (data not shown). In addition, the branching arrangement of the respective monophylies of Archaea, Eukarya, and Bacteria rejects any sort of horizontal gene transfer (HGT) involving SET genes from bacteria and vertebrates, although some ambiguous cases may arise in such analyses and could be, for example, a consequence of the poor representation of certain genomes in present-day databases (Stanhope et al., 2001). In contrast, phylogenetic and chromosome analyses of Chlorobium, Bacillus, and Methanosarcinal SET domain-containing species support an ancient HGT between bacteria and Archeae (Alvarez-Venegas et al., 2007).

## Pathogen and symbiont SET-domain containing proteins

### Chlamydiae SET domain proteins

One of the earliest reports on the identification of SET domain proteins in bacteria was the result of the genome sequencing of an obligate intracellular pathogen of humans that targets epithelial cells, *Chlamydia trachomatis* (Stephens et al., 1998). At that time, it was suggested that the SET domain protein CT737 in Chlamydia was the result of HGT based upon the assumption that the SET domain was found only in eukaryotic chromatin-associated proteins (Stephens et al., 1998).

**Figure 1:**
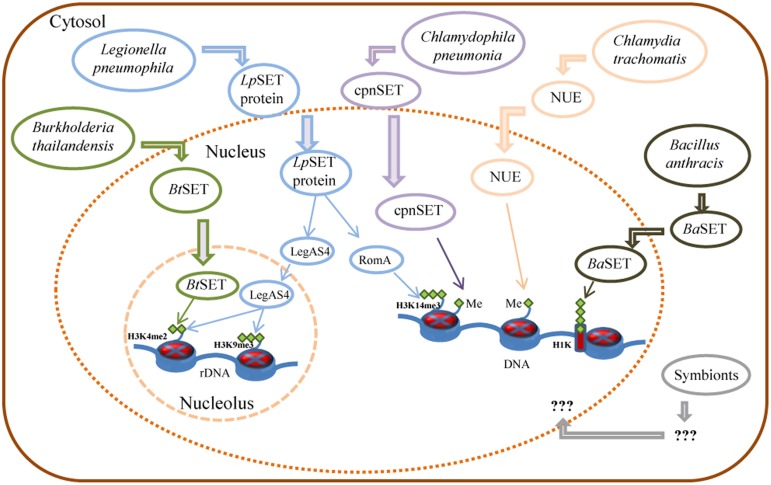
**Control of host gene expression by bacterial SET domain proteins and their mode of action**. Schematic representation of Burkholderia, Legionella, Chlamydia, and Bacillus secreted factors involved in the control of gene expression of host cells, as detailed in the text. Me: histone methylation; H3K14me3: tri-methylated lysine 14 of histone H3; H3K4me2: di-methylated lysine 4 of histone H3; H3K9me3: tri-methylated lysine 9 of histone H3; H1K: histone H1 lysine.

Unlike eukaryotes, prokaryotes do not have histones nor highly ordered chromatin. However, pathogenic bacteria must employ a wide range of tactics to avoid eradication by their host. Then, targeting histone modifications could be one of those strategies that allow a pathogen to inhibit transcriptional activation of host defense genes. With this in mind, Pennini et al. (2010) set out to characterize the chlamydial SET domain protein CT737 (named NUE, as the first “nuclear effector identified in chlamydiae”). They found a type three secretion (TTS) system signal in the N-terminal part of NUE. Also, Pennini and colleagues found that NUE acts as a TTS system effector protein, that it is secreted from bacteria, translocated to the host cell nucleus during *C. trachomatis* infection and associated with chromatin, particularly at late time points of infection. NUE has a histone methyltransferase activity that modified mammalian histones H2B, H3, and H4 but with a stronger activity toward H4 (Table 1). NUE itself automethylates, suggesting that automethylation enhances NUE enzymatic activity toward its substrate (Pennini et al., 2010). Consequently, the chlamydial SET domain protein seems to have evolved into a secreted protein capable to modify eukaryotic histones (Figure 1). Thus, by using its SET domain protein as an epigenetic control of host cells represents an advantage for Chlamydia in the persistence of chlamydial infection to maintain chronic disease progression.

**Table 1:** **Bacterial SET-domain proteins involved in eukaryotic chromatin modification**.

**Bacterial SET-domain protein**	**Bacterial species**	**GenBank accession number**	**Targeted histone(s)**	**Histone methyl mark**	**Targeted host/substrate**	**References**
CT737 or NUE	*Chlamydia trachomatis*	NP_220256	H2B, H3, H4	n.d.	Mammalian histones	Pennini et al., 2010
cpnSET	*Chlamydophila pneumoniae*	BAA99086	H3	n.d.	Murine histones	Murata et al., 2007
RomA	*Legionella pneumophila* strain Paris	YP_124001	H3	H3K14me3	Free histones; oligonucleo-somes; macrophages	Rolando et al., 2013
LegAS4	*Legionella pneumophila* strain	AAU27798	H3	H3K4me2; H3K9me3	Human macrophages	Li et al., 2013
	Philadelphia-Lp02					
LegAS4-like or *Bt*SET	*Burkholderia thailandensis*	YP_443833	H3	H3K4me and H3K4me2	Free histones	Li et al., 2013
*Ba*SET	*Bacillus anthracis*	YP_002869308	H1	Lysine residues	Macrophages	Mujtaba et al., 2013
Gö1-SET	*Methanosarcina mazei* strain Gö1	AAM32541	H4	H4K5	Bovine histones; MC1-α	Manzur and Zhou, 2005

*Abbreviations: n.d., not determined; H3K14me3, tri-methylated lysine 14 of histone H3; H3K4me2, di-methylated lysine 4 of histone H3*.

On the other hand, *Chlamydophila pneumonia*, an obligatory intracellular eubacterium that causes acute respiratory diseases (e.g., pneumonia) and chronic inflammatory processes (e.g., asthma, bronchitis, and chronic obstructive pulmonary disease; Roulis et al., 2013), has also a SET-domain coding gene, recently characterized (NCBI locus BAA99086). The *C. pneumoniae* SET domain protein (named cpnSET by Murata et al., 2007), with a murine histone H3 methyltransferase activity, has a similar expression pattern of two chlamydial histone H1-like proteins, Hc1, and Hc2. In addition, cpnSET has a physical interaction with the Hc1 and Hc2 proteins, as determined by the yeast two-hybrid system. Furthermore, Hc1 is also methylated by cpnSET, indicative that cpnSET may play an important role in chlamydial cell maturation due to modification of chlamydial histone H1-like proteins (Hc1 proteins) during the alternate morphologies between elementary bodies (EBs) and reticulate bodies (RBs) of *C. pneumonia* (Murata et al., 2007). However, localization of cpnSET was shown mainly in chlamydial cells (Murata et al., 2007), raising the possibility that the *C. pneumoniae* cpnSET protein is exported into host cells, and then cpnSET and host histones may functionally interact with each other. This is something extremely possible if we consider that the cpnSET protein has the two (“bipartite”) potential nuclear localization sequences (RHR at position 123 and KHRKKR at position 208) as those analyzed in the *C. trachomatis* translocated SET domain protein NUE (RRR at position 121 and KHRKKR at position 206; Pennini et al., 2010). Moreover, the chimeric N-terminal sequence of *C. pneumoniae* was secreted when expressed in *Shigella flexneri* ipaB (constitutive type three secretion—TTS-system), something highly indicative that chlamydiae SET domains proteins are TTS effectors (Pennini et al., 2010). Conversely, NUE did not methylate Hc1, as cpnSET did (Figure 1) (Murata et al., 2007; Pennini et al., 2010). This is something that deserves further investigation.

### Proteobacteria: *Legionella pneumophila*

The causative agent of a severe form of pneumonia called Legionnaires' disease, *Legionella pneumophila*, is a Gram-negative intracellular pathogen, ubiquitous in aquatic habitats where it survives and replicates within a wide range of protists, such as amoeba and ciliated protozoa. Upon transmission to humans, *L. pneumophila* invades and replicates in alveolar macrophages, evades the default endosome–lysosome pathway, remodels the phagosome, and escapes into the host cell cytosol. This ends in the expression of various bacterial virulence traits and bacterial escape to the extracellular environment, leading to the disease (Al-Quadan et al., 2012). *L. pneumophila* is a master manipulator of a variety of eukaryotic hosts ranging from unicellular amoebae to mammals. *L. pneumophila* facilitates this by taking control of numerous eukaryotic cellular functions through translocation of around 300 effectors into the host cell by the Dot/Icm type IVB secretion system. More than 70 of the injected effector proteins contain eukaryotic-like domains, including the ankyrin repeat, F-box, U-box, leucine-rich repeats and SET domain (Price and Abu Kwaik, 2013).

Recently, Rolando et al. (2013) identified one protein encoded by the gene *lpp1683* of the *L. pneumophila* strain Paris that contains a SET domain. It was shown that the Lpp1683 protein (named RomA, for regulator of methylation A) is a histone methyl transferase, with an apparent preference for histone H3. Specifically, RomA tri-methylates lysine 14 of histone H3 (H3K14), on free histones as well as in reconstituted oligonucleosomes. Then, Rolando and colleagues determined that RomA also conserved its activity *in vivo* during infection of human macrophages and amoeba, and that RomA localizes to the host nucleus in which it catalyzes a new histone methylation mark, H3K14. Deletion of a nuclear localization signal (NLS) in its N-terminal part altered the cellular distribution of RomA, leading to a predominant cytosolic localization. Fluorescent-based translocation assays showed that this protein is translocated in a Dot/Icm-dependent manner into the host cell. Next, it was shown that the H3K14 methylation mark, which replaces H3K14 acetylation (an active mark of ongoing transcription at the transcription start sites, TSSs), functions as a transcriptional repressive mark and results in global gene transcriptional repression, particularly in genes that are involved in innate immunity. Furthermore, deletion of RomA leaves the Paris strain defective in intracellular growth within both macrophages and amoebae, indicative that repression of host global transcription is important for *L. pneumophila* pathogenesis.

In contrast, Li et al. (2013) used the *L. pneumophila* Philadelphia-derivative Lp02 strain to characterize the protein LegAS4, a *L. pneumophila* type IV secretion system (TFSS) effector that contains a SET domain and tandem nuclear localization signals (NLS). LegAS4 is efficiently translocated from *L. pneumophila* into host macrophages and when ectopically expressed, LegAS4 was localized exclusively in the nuclei, specifically was localized in the host nucleolus, and associated with rDNA chromatin in which it catalyzes H3K4 di-methylation at the rDNA promoter and promoted rDNA transcription. Furthermore, LegAS4 interacted with host heterochromatin binding proteins HP1α and HP1γ, but extremely weakly with HP1β. The high-affinity HP1 binding is responsible for specific recruitment of LegAS4 to the transcriptionally silent rDNA chromatin region. Thus, *L. pneumophila* might exploit host ribosome activity for its own survival advantages by stimulation of rDNA transcription (Li et al., 2013).

Why those two highly homologous effector proteins have such inconsistent phenotypes in two different strains of *L. pneumophila*? This could be the result of the differences in the amino acid sequences of both proteins, as suggested by Price and Abu Kwaik (2013). For example, RomA is missing an amino-terminal fragment present in LegAS4. In addition, there is a weak sequence homology at an eight amino acid stretch almost in the middle of the proteins. These sequence differences could alter the structure of RomA from LegAS4 resulting in a change in histone methyltransferase substrate specificity. Also, LegAS4 has a robust localization to the nucleolus, which is not seen for RomA. The ability of LegAS4 to localize to the nucleolus is dependent on its tandem NLS, whereas RomA has three individual amino acid differences from LegAS4 in its corresponding NLS (Price and Abu Kwaik, 2013). Therefore, the structural differences between LegAS4 and RomA most likely explain the differential nucleolar protein targeting and distinct functional phenotypes of homologous effectors. It will be interesting to determine whether LegAS4 can also catalyze H4K14me3 in the nucleolus, as well as to determine the potential role of HP1 in RomA-mediated genome-wide repression.

Other bacterial pathogens, including *Bordetella bronchiseptica* and *Burkholderia thailandensis*, also possess LegAS4-like HKMTase effectors targeted to the host nucleolus. Specifically, the *B. thailandensis* type III effector BtSET upholds H3K4 methylation of rDNA chromatin and contributed to infection-induced rDNA transcription (Li et al., 2013). Thus, activation of rDNA transcription could be a common virulence strategy employed by bacterial pathogens for intracellular survival.

### Bacillus SET domain proteins

*Bacillus anthracis*, the etiologic agent of anthrax disease, is a Gram-positive spore-forming organism found in soil environments. *B. anthracis* spores are taken up by macrophages and/or dendritic cells, and subsequently migrate in the draining lymph nodes where they germinate, leading to bacterial multiplication, and dissemination through the whole organism (Raymond et al., 2009). Respiratory, gastrointestinal, or cutaneous entry of *B. anthracis* spores into mammals can result in a rapid systemic infection and death (mainly, in the pulmonary form and in the absence of treatment). Accordingly, the ability to drive bacterial molecules directly into host cells is a major strategy used by diverse bacterial pathogens to destabilize the host transcriptional machinery and to overcome host defenses. Strategically, most effectors aim to stabilize the NF-κB/IκB (nuclear factor-κ B/inhibitor of κ B) complex for precluding nuclear localization and transcriptional activation of the nuclear factor NF-κB (Mujtaba et al., 2013).

Recently, Mujtaba et al. (2013) characterized a SET-domain protein in *B. anthracis* (named *Ba*SET) in order to determine its role in *B. anthracis* survival in infected hosts. It was shown that *Ba*SET is a specific histone H1 trimethylase that functions as a transcriptional repressor by reducing the activation of NF-κB response elements (NF-κB _RE) in a dose-dependent expression, as well as by repressing diverse NF-κB target gene promoters. Particularly, *Ba*SET methylates eight lysine residues of the histone H1, which could be the strategy of the bacillus to hypermethylate host chromatin to silence the host inflammatory response. Furthermore, *Ba*SET is secreted by *B. anthracis* and localizes to the nucleus of infected macrophages where it methylates Histone H1, although the mechanism by which *Ba*SET translocates outside the bacillus is unclear, as the *Ba*SET sequence does not display any secretion signal (Mujtaba et al., 2013). Additionally, an engineered *Ba*SET deletion mutant (*Ba*ΔSET) indicated that *Ba*SET is not involved in the formation and germination of *B. anthracis* heat-resistant endospores, but that it plays a major role in the virulence of *B. anthracis*, as deletion of the gene eliminates the capacity for the organism to cause disease and death as well as survival in the infected host.

It will be interesting to determine the function of SET domain proteins present in other Bacillus, for example: *Bacillus cereus* (a ubiquitous soil organism and an opportunistic human pathogen most commonly associated with food poisoning), and *Bacillus thuringiensis* (an insect pathogen that is widely used as a bio-pesticide) (Han et al., 2006). But even more interesting will be the study of SET domain proteins in bacteria like *Bacillus megaterium* (a commercially available, nonpathogenic host for the biotechnological industry), and *Bacillus coahuilensis*, an ancient and a moderately halophilic, Gram-positive and rod-shaped bacterium, isolated from a Chihuahuan desert lagoon in Cuatro Ciénegas, Coahuila, México (Alcaraz et al., 2008).

### Archaeal SET domain

On the basis of their molecular properties, Archaea has been defined as a separate domain of life. Archaea lack nuclear membranes and are therefore prokaryotes, however, they are genetically and biochemically as divergent from bacteria as are eukarya. Archaea contain a set of sequence-independent DNA-binding proteins some of which undergo post-translational modifications, similar to the histone modifications in eukaryotic chromatin (Reeve, 2003). Among these archaeal DNA-binding proteins are the so-called histone-like proteins. On the other hand, SET domain encoding genes have also been identified in Archaea (Aravind and Iyer, 2003). Consequently, in order to determine the functional significance of SET domain proteins in Archaea, Manzur and Zhou (2005) characterized the first archaeal SET protein from the acetate-utilizing archaeal methanogen, *Methanosarcina mazei* strain Gö1 (referred as Gö1-SET).

The Gö1-SET protein was shown to selectively methylate *in vitro* bovine histone H4 at Lys5. However, histone H4 is not present in *M. mazei*. Alternatively, *M. mazei* has three DNA interacting proteins: a histone-like protein and two homologous MC1 proteins (methanogen chromosomal 1, MC1-α and MC1-β proteins). In view of that, *in vitro* MTase assays using the three DNA interacting proteins showed that Gö1-SET selectively methylates MC1-α but not the MC1-β and the histone-like protein, and that likely Lys37 of MC1-α is the specific target of Gö1-SET (Manzur and Zhou, 2005). Thus, archaeal SET domain proteins, like Gö1-SET, may regulate structures of archaeal chromatin composed of MC1–DNA complexes and indicate that chromatin modification by methylation took place before the separation of the archaeal and eukaryotic lineages (Manzur and Zhou, 2005).

Recently, a crenarchaeal protein lysine methyltransferase (named aKMT4, or also aKMT), which shows structural and enzymatic similarity to the eukaryotic KMT4/Dot1 family, has been characterized from *Sulfolobus islandicus* (Chu et al., 2012; Niu et al., 2013). However, such protein does not have a SET-domain and belongs to the Dot 1 family of histone lysine methyltransferases (KMTs). Nonetheless, the detection and further characterization of aKMT4/aKMT and protein homologs in other crenarchaeal species will allow a better understanding of the mechanisms involved in lysine methylation in crenarchaea and will clarify the evolutionary relationships among methyltransferases from the three domains of life.

### Symbionts

Most of the research related to complex interactions between eukaryotes and bacteria has been related to associations with microbes that are pathogenic; usually, microbial infection has been considered as deleterious, or at best irrelevant, to vigor and reproduction. However, symbionts and their metabolic potential play essential roles for many eukaryotic organisms that may benefit from enhanced fitness, survival, and even acquired virulence. Symbiosis is ubiquitous in terrestrial, freshwater, and marine ecosystems and it has played a crucial role in the appearance of major life forms on Earth and in the generation of biological diversity (Moran, 2006). Frequently, the associations are persistent for the hosts and are being transmitted vertically across generations. At times, the organisms involved in a symbiosis may be fully fused that they cannot live separately or be recognized as distinct entities without close scrutiny.

Recently, Partida-Martinez et al. (2007a,b) reported a unique symbiosis between bacteria belonging to the genus Burkholderia and the saprotrophic fungus *Rhizopus microsporus*. They have found that *Burkholderia rhizoxinica* is an intracellular symbiont of the phytopathogenic fungus *Rhizopus microspores* and that *B. rhizoxinica* is the producer of rhizoxin, the causative agent of rice seedling blight. This symbiosis represents an extraordinary example in which a fungus hosts a bacterial population for the production of a virulence factor. On the other hand, studies on the evolution of host resistance indicate that the fungus lost its ability to sporulate independently and became totally dependent on endobacteria for reproduction through spores (fungus formation of sporangia and spores is restored only upon reintroduction of endobacteria), thus warranting the persistence of the symbiosis and its efficient distribution through vegetative spores (Partida-Martinez et al., 2007a). Therefore, reproduction of the host is totally dependent on endofungal bacteria, which in return provides a highly potent toxin for defending the habitat and accessing nutrients from decaying plants.

Interestingly, *B. rhizoxinica* has a gene coding for a SET-domain protein (locus YP_004027789). However, the function of this SET protein is still unknown. On the other hand, *B. rhizoxinica* has a type II secretion pathway, an encoded type III secretion system shown to play a crucial role for the establishment of the symbiosis, and a putative type IV secretion system (Lackner et al., 2011). It is tempting to speculate that the SET-domain protein functions as a type III or type IV secretion system effector (just like SET proteins present in other proteobacteria), and that it might be translocated from *B. rhizoxinica* into the fungus *R. microspores*. The question is, for what purpose? If we take into consideration that a microbe that forms chronic infections in a host or in a host lineage may evolve to conserve or even to benefit its host, as this will help to maintain its immediate ecological resource (Moran, 2006), then we can only hypothesize that the bacterial SET protein might complement the array of post-translational modifications of histones in the fungus in order to improve bacterial intracellular replication through regulation of its host gene expression, and most important for the fungus, to formation of sporangia and spores. This is something that has to be scrutinized in the near future.

## Concluding remarks

Pathogenic or symbiotic bacteria make use of eukaryotic cell functions via specific interactions between microbial surface factors or secreted molecules and eukaryotic targets. These interactions, in turn, affect multiple signaling pathways and consequently promote a wide range of effects in host cells, including altered production of components involved in immune responses. Thus, as expected, bacteria employ a variety of strategies to affect the host cell cycle and gene expression program for their own benefit.

Recent studies have found that microbes affect a diverse set of epigenetic factors such as DNA methylation, histone modifications, chromatin-associated complexes, and non-coding RNAs (ncRNAs) to alter chromatin structure and gene expression. Intriguingly, DNA methylation plays a critical role in epigenetic gene regulation in eukaryotes as well as in prokaryotes (Kumar and Rao, 2013); Argonaute proteins, key players in RNA interference (RNAi) and related gene silencing phenomena in diverse eukaryotic species, are also present in many bacterial and archaeal species (Makarova et al., 2009); and post-translational modifications (PTMs) of proteins (e.g., histone and histone-like protein modifications) are used by both prokaryotic and eukaryotic cells to regulate the activity of key proteins. Thus, epigenetic mechanisms are clearly implicated in modulating biological interactions between hosts and bacterial pathogens and symbionts.

Chromatin modifications during animal development and in response to diverse environmental factors contribute to adult phenotypic variability and susceptibility to a number of diseases, including cancer, neurodegenerative, and neurological diseases and autoimmune disorders (Portela and Esteller, 2010). Because epigenetic modifications of chromatin may be transmitted to daughter cells during cell division, leading to heritable changes in gene expression, it is likely that a bacterial infection could generate heritable marks (Bierne et al., 2012). Therefore, understanding whether histone modification and/or DNA methylation marks imposed by bacterial proteins are maintained over time is a whole new area of research.

In plants, heritable histone modifications (e.g., histone lysine methylation) reported to regulate plant immunity against bacterial pathogens, by plant endogenous SET-domain proteins, have been associated for instance to the phenomenon of “priming” (a potentiated induction of defense genes and antimicrobial compounds for protection against biotic and/or abiotic stress). For example, Jaskiewicz et al. (2011) have demonstrated that during the interaction *Arabidopsis thaliana*-*Pseudomonas syringae* pv. *maculicola*, histone modifications on WRKY gene promoters have been detected in leaves distal to localized foliar infection for an augmented response to secondary stress. Thus, pathogen exposure induces one or more systemic signals that are stored on gene promoters in remote leaves in the form of histone modifications, mainly in the form of trimethylation of Lys 4 on histone H3 (H3K4me3) (Jaskiewicz et al., 2011). In another Arabidopsis study, *Pseudomonas syringae* pv *tomato* DC3000 (*Ps*tDC3000) inoculation increased resistance in subsequent generations. Progeny from *Ps*tDC3000-inoculated Arabidopsis were primed to activate salicylic acid-inducible defense genes and were more resistant to PstDC3000 (Luna et al., 2012). This transgenerational systemic acquired resistance indicates an epigenetic basis of the phenomenon.

However, neither *P. s*. pv. *maculicola* nor *Ps*tDC3000 have SET-domain coding genes. Thus, epigenetic changes can also contribute to and/or result from bacterial infectious diseases. Consequently, many open questions remain. For example, we should ask if events like priming take place only in plants that are being attacked by pathogens that do not have SET domain proteins, or if all bacterial pathogens induce priming. How immune priming induced protection does takes place in animals infected by pathogenic bacteria containing SET-domain proteins? What's more, it is essential to know how histone modifications might contribute to host response to infection and/or if bacteria take control of histone modifications to drive a transcriptional program beneficial for infection. Also, it is imperative to determine if SET domain proteins present in all pathogenic bacteria with a secretion system are secreted from bacteria, translocated to the host cell nucleus during infection and if they associate with chromatin. With respect to symbionts, do bacterial SET proteins complement the collection of post-translational modifications of their hosts to facilitate bacterial intracellular replication through regulation of its host gene expression? How do hosts benefit from bacteria containing SET-domain proteins in a symbiotic relationship? What group of bacterial SET-domain proteins has evolved into a chromatin-related role similar to their eukaryotic counterparts? How many new histone modifications are performed by bacterial SET-domain proteins, like RomA?

All these questions open new opportunities for future research in the subject of bacterial pathogenesis and chromatin-based regulation of host genes and may help to better understand the pathophysiology of bacterial infections and to develop efficient therapeutic approaches to treat important diseases, as well as to increase crop productivity.

### Conflict of interest statement

The author declares that the research was conducted in the absence of any commercial or financial relationships that could be construed as a potential conflict of interest.
