# Supplementary material for: Bacterial SET domain proteins and their role in eukaryotic chromatin modification
Source: Front Genet. 2014 Apr 2;5:65. doi: 10.3389/fgene.2014.00065 (PMC3980110; doi:10.3389/fgene.2014.00065)
Supplement: Supplementary file 1 [file DataSheet1.DOCX]

**Supplementary Table ST1**. Taxonomy report of bacterial SET-domain containing sequences using the “blastp suite” at the NCBI (Tax BLAST Report).

1. . [Bradyrhizobium sp. BTAi1](http://www.ncbi.nlm.nih.gov/Taxonomy/Browser/wwwtax.cgi?id=288000) ...................................... 105 [3 hits](http://blast.ncbi.nlm.nih.gov/Blast.cgi?CMD=Get&RID=5Y3VDS5A013&FORMAT_OBJECT=TaxBlast&NCBI_GI=off&DESCRIPTIONS=500&ALIGNMENTS=250&FORMAT_BLOCK_ON_RESPAGE=Top&MASK_COLOR=1&MASK_CHAR=2#288000) [[a-proteobacteria](http://www.ncbi.nlm.nih.gov/Taxonomy/Browser/wwwtax.cgi?id=28211)] [histone-lysine N-methyltransferase [Bradyrhizobium sp. BTAi](http://www.ncbi.nlm.nih.gov/entrez/query.fcgi?cmd=Retrieve&db=Protein&list_uids=148256757&dopt=GenPept)
2. . [Rhodopseudomonas palustris BisA53](http://www.ncbi.nlm.nih.gov/Taxonomy/Browser/wwwtax.cgi?id=316055) ............................. 102 [2 hits](http://blast.ncbi.nlm.nih.gov/Blast.cgi?CMD=Get&RID=5Y3VDS5A013&FORMAT_OBJECT=TaxBlast&NCBI_GI=off&DESCRIPTIONS=500&ALIGNMENTS=250&FORMAT_BLOCK_ON_RESPAGE=Top&MASK_COLOR=1&MASK_CHAR=2#316055) [[a-proteobacteria](http://www.ncbi.nlm.nih.gov/Taxonomy/Browser/wwwtax.cgi?id=28211)] [nuclear protein SET [Rhodopseudomonas palustris BisA53] >gi](http://www.ncbi.nlm.nih.gov/entrez/query.fcgi?cmd=Retrieve&db=Protein&list_uids=115523596&dopt=GenPept)
3. . [Rhodopseudomonas palustris](http://www.ncbi.nlm.nih.gov/Taxonomy/Browser/wwwtax.cgi?id=1076) .................................... 102 [7 hits](http://blast.ncbi.nlm.nih.gov/Blast.cgi?CMD=Get&RID=5Y3VDS5A013&FORMAT_OBJECT=TaxBlast&NCBI_GI=off&DESCRIPTIONS=500&ALIGNMENTS=250&FORMAT_BLOCK_ON_RESPAGE=Top&MASK_COLOR=1&MASK_CHAR=2#1076) [[a-proteobacteria](http://www.ncbi.nlm.nih.gov/Taxonomy/Browser/wwwtax.cgi?id=28211)] [nuclear protein SET [Rhodopseudomonas palustris BisA53] >gi](http://www.ncbi.nlm.nih.gov/entrez/query.fcgi?cmd=Retrieve&db=Protein&list_uids=499982295&dopt=GenPept)
4. . [Pseudoxanthomonas sp. GW2](http://www.ncbi.nlm.nih.gov/Taxonomy/Browser/wwwtax.cgi?id=1211114) ..................................... 101 [1 hit](http://blast.ncbi.nlm.nih.gov/Blast.cgi?CMD=Get&RID=5Y3VDS5A013&FORMAT_OBJECT=TaxBlast&NCBI_GI=off&DESCRIPTIONS=500&ALIGNMENTS=250&FORMAT_BLOCK_ON_RESPAGE=Top&MASK_COLOR=1&MASK_CHAR=2#1211114) [[g-proteobacteria](http://www.ncbi.nlm.nih.gov/Taxonomy/Browser/wwwtax.cgi?id=1236)] [nuclear protein SET [Pseudoxanthomonas sp. GW2]](http://www.ncbi.nlm.nih.gov/entrez/query.fcgi?cmd=Retrieve&db=Protein&list_uids=518227355&dopt=GenPept)
5. . [Bradyrhizobium sp. ORS 278](http://www.ncbi.nlm.nih.gov/Taxonomy/Browser/wwwtax.cgi?id=114615) .................................... 102 [3 hits](http://blast.ncbi.nlm.nih.gov/Blast.cgi?CMD=Get&RID=5Y3VDS5A013&FORMAT_OBJECT=TaxBlast&NCBI_GI=off&DESCRIPTIONS=500&ALIGNMENTS=250&FORMAT_BLOCK_ON_RESPAGE=Top&MASK_COLOR=1&MASK_CHAR=2#114615) [[a-proteobacteria](http://www.ncbi.nlm.nih.gov/Taxonomy/Browser/wwwtax.cgi?id=28211)] [histone-lysine N-methyltransferase [Bradyrhizobium sp. ORS](http://www.ncbi.nlm.nih.gov/entrez/query.fcgi?cmd=Retrieve&db=Protein&list_uids=146341873&dopt=GenPept)
6. . [Bordetella petrii DSM 12804](http://www.ncbi.nlm.nih.gov/Taxonomy/Browser/wwwtax.cgi?id=340100) ................................... 101 [1 hit](http://blast.ncbi.nlm.nih.gov/Blast.cgi?CMD=Get&RID=5Y3VDS5A013&FORMAT_OBJECT=TaxBlast&NCBI_GI=off&DESCRIPTIONS=500&ALIGNMENTS=250&FORMAT_BLOCK_ON_RESPAGE=Top&MASK_COLOR=1&MASK_CHAR=2#340100) [[b-proteobacteria](http://www.ncbi.nlm.nih.gov/Taxonomy/Browser/wwwtax.cgi?id=28216)] [hypothetical protein Bpet0025 [Bordetella petrii DSM 12804]](http://www.ncbi.nlm.nih.gov/entrez/query.fcgi?cmd=Retrieve&db=Protein&list_uids=163854329&dopt=GenPept)
7. . [Bordetella petrii](http://www.ncbi.nlm.nih.gov/Taxonomy/Browser/wwwtax.cgi?id=94624) ............................................. 101 [2 hits](http://blast.ncbi.nlm.nih.gov/Blast.cgi?CMD=Get&RID=5Y3VDS5A013&FORMAT_OBJECT=TaxBlast&NCBI_GI=off&DESCRIPTIONS=500&ALIGNMENTS=250&FORMAT_BLOCK_ON_RESPAGE=Top&MASK_COLOR=1&MASK_CHAR=2#94624) [[b-proteobacteria](http://www.ncbi.nlm.nih.gov/Taxonomy/Browser/wwwtax.cgi?id=28216)] [hypothetical protein Bpet0025 [Bordetella petrii DSM 12804]](http://www.ncbi.nlm.nih.gov/entrez/query.fcgi?cmd=Retrieve&db=Protein&list_uids=501204036&dopt=GenPept)
8. . [Bordetella pertussis Tohama I](http://www.ncbi.nlm.nih.gov/Taxonomy/Browser/wwwtax.cgi?id=257313) ................................. 100 [2 hits](http://blast.ncbi.nlm.nih.gov/Blast.cgi?CMD=Get&RID=5Y3VDS5A013&FORMAT_OBJECT=TaxBlast&NCBI_GI=off&DESCRIPTIONS=500&ALIGNMENTS=250&FORMAT_BLOCK_ON_RESPAGE=Top&MASK_COLOR=1&MASK_CHAR=2#257313) [[b-proteobacteria](http://www.ncbi.nlm.nih.gov/Taxonomy/Browser/wwwtax.cgi?id=28216)] [hypothetical protein BP0470 [Bordetella pertussis Tohama I]](http://www.ncbi.nlm.nih.gov/entrez/query.fcgi?cmd=Retrieve&db=Protein&list_uids=33591683&dopt=GenPept)
9. . [Bordetella pertussis CS](http://www.ncbi.nlm.nih.gov/Taxonomy/Browser/wwwtax.cgi?id=1017264) ....................................... 100 [2 hits](http://blast.ncbi.nlm.nih.gov/Blast.cgi?CMD=Get&RID=5Y3VDS5A013&FORMAT_OBJECT=TaxBlast&NCBI_GI=off&DESCRIPTIONS=500&ALIGNMENTS=250&FORMAT_BLOCK_ON_RESPAGE=Top&MASK_COLOR=1&MASK_CHAR=2#1017264) [[b-proteobacteria](http://www.ncbi.nlm.nih.gov/Taxonomy/Browser/wwwtax.cgi?id=28216)] [hypothetical protein BP0470 [Bordetella pertussis Tohama I]](http://www.ncbi.nlm.nih.gov/entrez/query.fcgi?cmd=Retrieve&db=Protein&list_uids=384202971&dopt=GenPept)
10. . [Bordetella pertussis 18323](http://www.ncbi.nlm.nih.gov/Taxonomy/Browser/wwwtax.cgi?id=568706) .................................... 100 [2 hits](http://blast.ncbi.nlm.nih.gov/Blast.cgi?CMD=Get&RID=5Y3VDS5A013&FORMAT_OBJECT=TaxBlast&NCBI_GI=off&DESCRIPTIONS=500&ALIGNMENTS=250&FORMAT_BLOCK_ON_RESPAGE=Top&MASK_COLOR=1&MASK_CHAR=2#568706) [[b-proteobacteria](http://www.ncbi.nlm.nih.gov/Taxonomy/Browser/wwwtax.cgi?id=28216)] [hypothetical protein BP0470 [Bordetella pertussis Tohama I]](http://www.ncbi.nlm.nih.gov/entrez/query.fcgi?cmd=Retrieve&db=Protein&list_uids=408414487&dopt=GenPept)
11. . [Bordetella pertussis](http://www.ncbi.nlm.nih.gov/Taxonomy/Browser/wwwtax.cgi?id=520) .......................................... 100 [1 hit](http://blast.ncbi.nlm.nih.gov/Blast.cgi?CMD=Get&RID=5Y3VDS5A013&FORMAT_OBJECT=TaxBlast&NCBI_GI=off&DESCRIPTIONS=500&ALIGNMENTS=250&FORMAT_BLOCK_ON_RESPAGE=Top&MASK_COLOR=1&MASK_CHAR=2#520) [[b-proteobacteria](http://www.ncbi.nlm.nih.gov/Taxonomy/Browser/wwwtax.cgi?id=28216)] [hypothetical protein BP0470 [Bordetella pertussis Tohama I]](http://www.ncbi.nlm.nih.gov/entrez/query.fcgi?cmd=Retrieve&db=Protein&list_uids=499232285&dopt=GenPept)
12. . [Bradyrhizobium sp. STM 3809](http://www.ncbi.nlm.nih.gov/Taxonomy/Browser/wwwtax.cgi?id=551936) ................................... 102 [2 hits](http://blast.ncbi.nlm.nih.gov/Blast.cgi?CMD=Get&RID=5Y3VDS5A013&FORMAT_OBJECT=TaxBlast&NCBI_GI=off&DESCRIPTIONS=500&ALIGNMENTS=250&FORMAT_BLOCK_ON_RESPAGE=Top&MASK_COLOR=1&MASK_CHAR=2#551936) [[a-proteobacteria](http://www.ncbi.nlm.nih.gov/Taxonomy/Browser/wwwtax.cgi?id=28211)] [histone-lysine N-methyltransferase with a SET domain [Brady](http://www.ncbi.nlm.nih.gov/entrez/query.fcgi?cmd=Retrieve&db=Protein&list_uids=496246974&dopt=GenPept)
13. . [Bordetella bronchiseptica MO149](http://www.ncbi.nlm.nih.gov/Taxonomy/Browser/wwwtax.cgi?id=1208658) ............................... 100 [2 hits](http://blast.ncbi.nlm.nih.gov/Blast.cgi?CMD=Get&RID=5Y3VDS5A013&FORMAT_OBJECT=TaxBlast&NCBI_GI=off&DESCRIPTIONS=500&ALIGNMENTS=250&FORMAT_BLOCK_ON_RESPAGE=Top&MASK_COLOR=1&MASK_CHAR=2#1208658) [[b-proteobacteria](http://www.ncbi.nlm.nih.gov/Taxonomy/Browser/wwwtax.cgi?id=28216)] [hypothetical protein BN115_4628 [Bordetella bronchiseptica](http://www.ncbi.nlm.nih.gov/entrez/query.fcgi?cmd=Retrieve&db=Protein&list_uids=410422397&dopt=GenPept)
14. . [Bordetella bronchiseptica](http://www.ncbi.nlm.nih.gov/Taxonomy/Browser/wwwtax.cgi?id=518) ..................................... 100 [1 hit](http://blast.ncbi.nlm.nih.gov/Blast.cgi?CMD=Get&RID=5Y3VDS5A013&FORMAT_OBJECT=TaxBlast&NCBI_GI=off&DESCRIPTIONS=500&ALIGNMENTS=250&FORMAT_BLOCK_ON_RESPAGE=Top&MASK_COLOR=1&MASK_CHAR=2#518) [[b-proteobacteria](http://www.ncbi.nlm.nih.gov/Taxonomy/Browser/wwwtax.cgi?id=28216)] [hypothetical protein BN115_4628 [Bordetella bronchiseptica](http://www.ncbi.nlm.nih.gov/entrez/query.fcgi?cmd=Retrieve&db=Protein&list_uids=489914829&dopt=GenPept)
15. . [Bordetella bronchiseptica D445](http://www.ncbi.nlm.nih.gov/Taxonomy/Browser/wwwtax.cgi?id=1208665) ................................ 100 [1 hit](http://blast.ncbi.nlm.nih.gov/Blast.cgi?CMD=Get&RID=5Y3VDS5A013&FORMAT_OBJECT=TaxBlast&NCBI_GI=off&DESCRIPTIONS=500&ALIGNMENTS=250&FORMAT_BLOCK_ON_RESPAGE=Top&MASK_COLOR=1&MASK_CHAR=2#1208665) [[b-proteobacteria](http://www.ncbi.nlm.nih.gov/Taxonomy/Browser/wwwtax.cgi?id=28216)] [hypothetical protein BN115_4628 [Bordetella bronchiseptica](http://www.ncbi.nlm.nih.gov/entrez/query.fcgi?cmd=Retrieve&db=Protein&list_uids=410568267&dopt=GenPept)
16. . [Bordetella bronchiseptica Bbr77](http://www.ncbi.nlm.nih.gov/Taxonomy/Browser/wwwtax.cgi?id=1208659) ............................... 100 [1 hit](http://blast.ncbi.nlm.nih.gov/Blast.cgi?CMD=Get&RID=5Y3VDS5A013&FORMAT_OBJECT=TaxBlast&NCBI_GI=off&DESCRIPTIONS=500&ALIGNMENTS=250&FORMAT_BLOCK_ON_RESPAGE=Top&MASK_COLOR=1&MASK_CHAR=2#1208659) [[b-proteobacteria](http://www.ncbi.nlm.nih.gov/Taxonomy/Browser/wwwtax.cgi?id=28216)] [hypothetical protein BN115_4628 [Bordetella bronchiseptica](http://www.ncbi.nlm.nih.gov/entrez/query.fcgi?cmd=Retrieve&db=Protein&list_uids=410588264&dopt=GenPept)
17. . [Bradyrhizobium sp. ORS 375](http://www.ncbi.nlm.nih.gov/Taxonomy/Browser/wwwtax.cgi?id=566679) .................................... 102 [2 hits](http://blast.ncbi.nlm.nih.gov/Blast.cgi?CMD=Get&RID=5Y3VDS5A013&FORMAT_OBJECT=TaxBlast&NCBI_GI=off&DESCRIPTIONS=500&ALIGNMENTS=250&FORMAT_BLOCK_ON_RESPAGE=Top&MASK_COLOR=1&MASK_CHAR=2#566679) [[a-proteobacteria](http://www.ncbi.nlm.nih.gov/Taxonomy/Browser/wwwtax.cgi?id=28211)] [histone-lysine N-methyltransferase with a SET domain [Brady](http://www.ncbi.nlm.nih.gov/entrez/query.fcgi?cmd=Retrieve&db=Protein&list_uids=496321928&dopt=GenPept)
18. . [Bradyrhizobium sp. STM 3843](http://www.ncbi.nlm.nih.gov/Taxonomy/Browser/wwwtax.cgi?id=551947) ................................... 101 [2 hits](http://blast.ncbi.nlm.nih.gov/Blast.cgi?CMD=Get&RID=5Y3VDS5A013&FORMAT_OBJECT=TaxBlast&NCBI_GI=off&DESCRIPTIONS=500&ALIGNMENTS=250&FORMAT_BLOCK_ON_RESPAGE=Top&MASK_COLOR=1&MASK_CHAR=2#551947) [[a-proteobacteria](http://www.ncbi.nlm.nih.gov/Taxonomy/Browser/wwwtax.cgi?id=28211)] [histone-lysine N-methyltransferase with a SET domain [Brady](http://www.ncbi.nlm.nih.gov/entrez/query.fcgi?cmd=Retrieve&db=Protein&list_uids=496258273&dopt=GenPept)
19. . [Bordetella parapertussis 12822](http://www.ncbi.nlm.nih.gov/Taxonomy/Browser/wwwtax.cgi?id=257311) ................................ 100 [1 hit](http://blast.ncbi.nlm.nih.gov/Blast.cgi?CMD=Get&RID=5Y3VDS5A013&FORMAT_OBJECT=TaxBlast&NCBI_GI=off&DESCRIPTIONS=500&ALIGNMENTS=250&FORMAT_BLOCK_ON_RESPAGE=Top&MASK_COLOR=1&MASK_CHAR=2#257311) [[b-proteobacteria](http://www.ncbi.nlm.nih.gov/Taxonomy/Browser/wwwtax.cgi?id=28216)] [hypothetical protein BPP4384 [Bordetella parapertussis 1282](http://www.ncbi.nlm.nih.gov/entrez/query.fcgi?cmd=Retrieve&db=Protein&list_uids=33598867&dopt=GenPept)
20. . [Bordetella parapertussis](http://www.ncbi.nlm.nih.gov/Taxonomy/Browser/wwwtax.cgi?id=519) ...................................... 100 [2 hits](http://blast.ncbi.nlm.nih.gov/Blast.cgi?CMD=Get&RID=5Y3VDS5A013&FORMAT_OBJECT=TaxBlast&NCBI_GI=off&DESCRIPTIONS=500&ALIGNMENTS=250&FORMAT_BLOCK_ON_RESPAGE=Top&MASK_COLOR=1&MASK_CHAR=2#519) [[b-proteobacteria](http://www.ncbi.nlm.nih.gov/Taxonomy/Browser/wwwtax.cgi?id=28216)] [hypothetical protein BPP4384 [Bordetella parapertussis 1282](http://www.ncbi.nlm.nih.gov/entrez/query.fcgi?cmd=Retrieve&db=Protein&list_uids=499232002&dopt=GenPept)
21. . [Chlamydia trachomatis A/HAR-13](http://www.ncbi.nlm.nih.gov/Taxonomy/Browser/wwwtax.cgi?id=315277) ................................ 101 [2 hits](http://blast.ncbi.nlm.nih.gov/Blast.cgi?CMD=Get&RID=5Y3VDS5A013&FORMAT_OBJECT=TaxBlast&NCBI_GI=off&DESCRIPTIONS=500&ALIGNMENTS=250&FORMAT_BLOCK_ON_RESPAGE=Top&MASK_COLOR=1&MASK_CHAR=2#315277) [[chlamydias](http://www.ncbi.nlm.nih.gov/Taxonomy/Browser/wwwtax.cgi?id=51291)] [SET domain-containing protein [Chlamydia trachomatis A/HAR-](http://www.ncbi.nlm.nih.gov/entrez/query.fcgi?cmd=Retrieve&db=Protein&list_uids=76789477&dopt=GenPept)
22. . [Chlamydia trachomatis 434/Bu](http://www.ncbi.nlm.nih.gov/Taxonomy/Browser/wwwtax.cgi?id=471472) .................................. 101 [2 hits](http://blast.ncbi.nlm.nih.gov/Blast.cgi?CMD=Get&RID=5Y3VDS5A013&FORMAT_OBJECT=TaxBlast&NCBI_GI=off&DESCRIPTIONS=500&ALIGNMENTS=250&FORMAT_BLOCK_ON_RESPAGE=Top&MASK_COLOR=1&MASK_CHAR=2#471472) [[chlamydias](http://www.ncbi.nlm.nih.gov/Taxonomy/Browser/wwwtax.cgi?id=51291)] [SET domain-containing protein [Chlamydia trachomatis A/HAR-](http://www.ncbi.nlm.nih.gov/entrez/query.fcgi?cmd=Retrieve&db=Protein&list_uids=166154079&dopt=GenPept)
23. . [Chlamydia trachomatis L2b/UCH-1/proctitis](http://www.ncbi.nlm.nih.gov/Taxonomy/Browser/wwwtax.cgi?id=471473) ..................... 101 [2 hits](http://blast.ncbi.nlm.nih.gov/Blast.cgi?CMD=Get&RID=5Y3VDS5A013&FORMAT_OBJECT=TaxBlast&NCBI_GI=off&DESCRIPTIONS=500&ALIGNMENTS=250&FORMAT_BLOCK_ON_RESPAGE=Top&MASK_COLOR=1&MASK_CHAR=2#471473) [[chlamydias](http://www.ncbi.nlm.nih.gov/Taxonomy/Browser/wwwtax.cgi?id=51291)] [SET domain-containing protein [Chlamydia trachomatis A/HAR-](http://www.ncbi.nlm.nih.gov/entrez/query.fcgi?cmd=Retrieve&db=Protein&list_uids=166154954&dopt=GenPept)
24. . [Chlamydia trachomatis B/Jali20/OT](http://www.ncbi.nlm.nih.gov/Taxonomy/Browser/wwwtax.cgi?id=580049) ............................. 101 [2 hits](http://blast.ncbi.nlm.nih.gov/Blast.cgi?CMD=Get&RID=5Y3VDS5A013&FORMAT_OBJECT=TaxBlast&NCBI_GI=off&DESCRIPTIONS=500&ALIGNMENTS=250&FORMAT_BLOCK_ON_RESPAGE=Top&MASK_COLOR=1&MASK_CHAR=2#580049) [[chlamydias](http://www.ncbi.nlm.nih.gov/Taxonomy/Browser/wwwtax.cgi?id=51291)] [SET domain-containing protein [Chlamydia trachomatis A/HAR-](http://www.ncbi.nlm.nih.gov/entrez/query.fcgi?cmd=Retrieve&db=Protein&list_uids=237803167&dopt=GenPept)
25. . [Chlamydia trachomatis B/TZ1A828/OT](http://www.ncbi.nlm.nih.gov/Taxonomy/Browser/wwwtax.cgi?id=672161) ............................ 101 [2 hits](http://blast.ncbi.nlm.nih.gov/Blast.cgi?CMD=Get&RID=5Y3VDS5A013&FORMAT_OBJECT=TaxBlast&NCBI_GI=off&DESCRIPTIONS=500&ALIGNMENTS=250&FORMAT_BLOCK_ON_RESPAGE=Top&MASK_COLOR=1&MASK_CHAR=2#672161) [[chlamydias](http://www.ncbi.nlm.nih.gov/Taxonomy/Browser/wwwtax.cgi?id=51291)] [SET domain-containing protein [Chlamydia trachomatis A/HAR-](http://www.ncbi.nlm.nih.gov/entrez/query.fcgi?cmd=Retrieve&db=Protein&list_uids=237805088&dopt=GenPept)
26. . [Chlamydia trachomatis L2c](http://www.ncbi.nlm.nih.gov/Taxonomy/Browser/wwwtax.cgi?id=887712) ..................................... 101 [2 hits](http://blast.ncbi.nlm.nih.gov/Blast.cgi?CMD=Get&RID=5Y3VDS5A013&FORMAT_OBJECT=TaxBlast&NCBI_GI=off&DESCRIPTIONS=500&ALIGNMENTS=250&FORMAT_BLOCK_ON_RESPAGE=Top&MASK_COLOR=1&MASK_CHAR=2#887712) [[chlamydias](http://www.ncbi.nlm.nih.gov/Taxonomy/Browser/wwwtax.cgi?id=51291)] [SET domain-containing protein [Chlamydia trachomatis A/HAR-](http://www.ncbi.nlm.nih.gov/entrez/query.fcgi?cmd=Retrieve&db=Protein&list_uids=339625485&dopt=GenPept)
27. . [Chlamydia trachomatis A2497](http://www.ncbi.nlm.nih.gov/Taxonomy/Browser/wwwtax.cgi?id=580047) ................................... 101 [4 hits](http://blast.ncbi.nlm.nih.gov/Blast.cgi?CMD=Get&RID=5Y3VDS5A013&FORMAT_OBJECT=TaxBlast&NCBI_GI=off&DESCRIPTIONS=500&ALIGNMENTS=250&FORMAT_BLOCK_ON_RESPAGE=Top&MASK_COLOR=1&MASK_CHAR=2#580047) [[chlamydias](http://www.ncbi.nlm.nih.gov/Taxonomy/Browser/wwwtax.cgi?id=51291)] [SET domain-containing protein [Chlamydia trachomatis A/HAR-](http://www.ncbi.nlm.nih.gov/entrez/query.fcgi?cmd=Retrieve&db=Protein&list_uids=376282747&dopt=GenPept)
28. . [Chlamydia trachomatis G/9768](http://www.ncbi.nlm.nih.gov/Taxonomy/Browser/wwwtax.cgi?id=707185) .................................. 101 [2 hits](http://blast.ncbi.nlm.nih.gov/Blast.cgi?CMD=Get&RID=5Y3VDS5A013&FORMAT_OBJECT=TaxBlast&NCBI_GI=off&DESCRIPTIONS=500&ALIGNMENTS=250&FORMAT_BLOCK_ON_RESPAGE=Top&MASK_COLOR=1&MASK_CHAR=2#707185) [[chlamydias](http://www.ncbi.nlm.nih.gov/Taxonomy/Browser/wwwtax.cgi?id=51291)] [SET domain-containing protein [Chlamydia trachomatis A/HAR-](http://www.ncbi.nlm.nih.gov/entrez/query.fcgi?cmd=Retrieve&db=Protein&list_uids=385240278&dopt=GenPept)
29. . [Chlamydia trachomatis G/11222](http://www.ncbi.nlm.nih.gov/Taxonomy/Browser/wwwtax.cgi?id=707186) ................................. 101 [2 hits](http://blast.ncbi.nlm.nih.gov/Blast.cgi?CMD=Get&RID=5Y3VDS5A013&FORMAT_OBJECT=TaxBlast&NCBI_GI=off&DESCRIPTIONS=500&ALIGNMENTS=250&FORMAT_BLOCK_ON_RESPAGE=Top&MASK_COLOR=1&MASK_CHAR=2#707186) [[chlamydias](http://www.ncbi.nlm.nih.gov/Taxonomy/Browser/wwwtax.cgi?id=51291)] [SET domain-containing protein [Chlamydia trachomatis A/HAR-](http://www.ncbi.nlm.nih.gov/entrez/query.fcgi?cmd=Retrieve&db=Protein&list_uids=385241204&dopt=GenPept)
30. . [Chlamydia trachomatis E/11023](http://www.ncbi.nlm.nih.gov/Taxonomy/Browser/wwwtax.cgi?id=707183) ................................. 101 [2 hits](http://blast.ncbi.nlm.nih.gov/Blast.cgi?CMD=Get&RID=5Y3VDS5A013&FORMAT_OBJECT=TaxBlast&NCBI_GI=off&DESCRIPTIONS=500&ALIGNMENTS=250&FORMAT_BLOCK_ON_RESPAGE=Top&MASK_COLOR=1&MASK_CHAR=2#707183) [[chlamydias](http://www.ncbi.nlm.nih.gov/Taxonomy/Browser/wwwtax.cgi?id=51291)] [SET domain-containing protein [Chlamydia trachomatis A/HAR-](http://www.ncbi.nlm.nih.gov/entrez/query.fcgi?cmd=Retrieve&db=Protein&list_uids=385242130&dopt=GenPept)
31. . [Chlamydia trachomatis G/9301](http://www.ncbi.nlm.nih.gov/Taxonomy/Browser/wwwtax.cgi?id=718219) .................................. 101 [2 hits](http://blast.ncbi.nlm.nih.gov/Blast.cgi?CMD=Get&RID=5Y3VDS5A013&FORMAT_OBJECT=TaxBlast&NCBI_GI=off&DESCRIPTIONS=500&ALIGNMENTS=250&FORMAT_BLOCK_ON_RESPAGE=Top&MASK_COLOR=1&MASK_CHAR=2#718219) [[chlamydias](http://www.ncbi.nlm.nih.gov/Taxonomy/Browser/wwwtax.cgi?id=51291)] [SET domain-containing protein [Chlamydia trachomatis A/HAR-](http://www.ncbi.nlm.nih.gov/entrez/query.fcgi?cmd=Retrieve&db=Protein&list_uids=385243056&dopt=GenPept)
32. . [Chlamydia trachomatis E/150](http://www.ncbi.nlm.nih.gov/Taxonomy/Browser/wwwtax.cgi?id=707184) ................................... 101 [2 hits](http://blast.ncbi.nlm.nih.gov/Blast.cgi?CMD=Get&RID=5Y3VDS5A013&FORMAT_OBJECT=TaxBlast&NCBI_GI=off&DESCRIPTIONS=500&ALIGNMENTS=250&FORMAT_BLOCK_ON_RESPAGE=Top&MASK_COLOR=1&MASK_CHAR=2#707184) [[chlamydias](http://www.ncbi.nlm.nih.gov/Taxonomy/Browser/wwwtax.cgi?id=51291)] [SET domain-containing protein [Chlamydia trachomatis A/HAR-](http://www.ncbi.nlm.nih.gov/entrez/query.fcgi?cmd=Retrieve&db=Protein&list_uids=385245740&dopt=GenPept)
33. . [Chlamydia trachomatis G/11074](http://www.ncbi.nlm.nih.gov/Taxonomy/Browser/wwwtax.cgi?id=707187) ................................. 101 [2 hits](http://blast.ncbi.nlm.nih.gov/Blast.cgi?CMD=Get&RID=5Y3VDS5A013&FORMAT_OBJECT=TaxBlast&NCBI_GI=off&DESCRIPTIONS=500&ALIGNMENTS=250&FORMAT_BLOCK_ON_RESPAGE=Top&MASK_COLOR=1&MASK_CHAR=2#707187) [[chlamydias](http://www.ncbi.nlm.nih.gov/Taxonomy/Browser/wwwtax.cgi?id=51291)] [SET domain-containing protein [Chlamydia trachomatis A/HAR-](http://www.ncbi.nlm.nih.gov/entrez/query.fcgi?cmd=Retrieve&db=Protein&list_uids=385246664&dopt=GenPept)
34. . [Chlamydia trachomatis Sweden2](http://www.ncbi.nlm.nih.gov/Taxonomy/Browser/wwwtax.cgi?id=634464) ................................. 101 [2 hits](http://blast.ncbi.nlm.nih.gov/Blast.cgi?CMD=Get&RID=5Y3VDS5A013&FORMAT_OBJECT=TaxBlast&NCBI_GI=off&DESCRIPTIONS=500&ALIGNMENTS=250&FORMAT_BLOCK_ON_RESPAGE=Top&MASK_COLOR=1&MASK_CHAR=2#634464) [[chlamydias](http://www.ncbi.nlm.nih.gov/Taxonomy/Browser/wwwtax.cgi?id=51291)] [SET domain-containing protein [Chlamydia trachomatis A/HAR-](http://www.ncbi.nlm.nih.gov/entrez/query.fcgi?cmd=Retrieve&db=Protein&list_uids=386263093&dopt=GenPept)
35. . [Chlamydia trachomatis F/SW4](http://www.ncbi.nlm.nih.gov/Taxonomy/Browser/wwwtax.cgi?id=1071762) ................................... 101 [2 hits](http://blast.ncbi.nlm.nih.gov/Blast.cgi?CMD=Get&RID=5Y3VDS5A013&FORMAT_OBJECT=TaxBlast&NCBI_GI=off&DESCRIPTIONS=500&ALIGNMENTS=250&FORMAT_BLOCK_ON_RESPAGE=Top&MASK_COLOR=1&MASK_CHAR=2#1071762) [[chlamydias](http://www.ncbi.nlm.nih.gov/Taxonomy/Browser/wwwtax.cgi?id=51291)] [SET domain-containing protein [Chlamydia trachomatis A/HAR-](http://www.ncbi.nlm.nih.gov/entrez/query.fcgi?cmd=Retrieve&db=Protein&list_uids=389858432&dopt=GenPept)
36. . [Chlamydia trachomatis E/SW3](http://www.ncbi.nlm.nih.gov/Taxonomy/Browser/wwwtax.cgi?id=1071759) ................................... 101 [2 hits](http://blast.ncbi.nlm.nih.gov/Blast.cgi?CMD=Get&RID=5Y3VDS5A013&FORMAT_OBJECT=TaxBlast&NCBI_GI=off&DESCRIPTIONS=500&ALIGNMENTS=250&FORMAT_BLOCK_ON_RESPAGE=Top&MASK_COLOR=1&MASK_CHAR=2#1071759) [[chlamydias](http://www.ncbi.nlm.nih.gov/Taxonomy/Browser/wwwtax.cgi?id=51291)] [SET domain-containing protein [Chlamydia trachomatis A/HAR-](http://www.ncbi.nlm.nih.gov/entrez/query.fcgi?cmd=Retrieve&db=Protein&list_uids=389859308&dopt=GenPept)
37. . [Chlamydia trachomatis F/SW5](http://www.ncbi.nlm.nih.gov/Taxonomy/Browser/wwwtax.cgi?id=1071763) ................................... 101 [2 hits](http://blast.ncbi.nlm.nih.gov/Blast.cgi?CMD=Get&RID=5Y3VDS5A013&FORMAT_OBJECT=TaxBlast&NCBI_GI=off&DESCRIPTIONS=500&ALIGNMENTS=250&FORMAT_BLOCK_ON_RESPAGE=Top&MASK_COLOR=1&MASK_CHAR=2#1071763) [[chlamydias](http://www.ncbi.nlm.nih.gov/Taxonomy/Browser/wwwtax.cgi?id=51291)] [SET domain-containing protein [Chlamydia trachomatis A/HAR-](http://www.ncbi.nlm.nih.gov/entrez/query.fcgi?cmd=Retrieve&db=Protein&list_uids=389860184&dopt=GenPept)
38. . [Chlamydia trachomatis IU824](http://www.ncbi.nlm.nih.gov/Taxonomy/Browser/wwwtax.cgi?id=1260222) ................................... 101 [2 hits](http://blast.ncbi.nlm.nih.gov/Blast.cgi?CMD=Get&RID=5Y3VDS5A013&FORMAT_OBJECT=TaxBlast&NCBI_GI=off&DESCRIPTIONS=500&ALIGNMENTS=250&FORMAT_BLOCK_ON_RESPAGE=Top&MASK_COLOR=1&MASK_CHAR=2#1260222) [[chlamydias](http://www.ncbi.nlm.nih.gov/Taxonomy/Browser/wwwtax.cgi?id=51291)] [SET domain-containing protein [Chlamydia trachomatis A/HAR-](http://www.ncbi.nlm.nih.gov/entrez/query.fcgi?cmd=Retrieve&db=Protein&list_uids=471328911&dopt=GenPept)
39. . [Chlamydia trachomatis IU888](http://www.ncbi.nlm.nih.gov/Taxonomy/Browser/wwwtax.cgi?id=1260223) ................................... 101 [2 hits](http://blast.ncbi.nlm.nih.gov/Blast.cgi?CMD=Get&RID=5Y3VDS5A013&FORMAT_OBJECT=TaxBlast&NCBI_GI=off&DESCRIPTIONS=500&ALIGNMENTS=250&FORMAT_BLOCK_ON_RESPAGE=Top&MASK_COLOR=1&MASK_CHAR=2#1260223) [[chlamydias](http://www.ncbi.nlm.nih.gov/Taxonomy/Browser/wwwtax.cgi?id=51291)] [SET domain-containing protein [Chlamydia trachomatis A/HAR-](http://www.ncbi.nlm.nih.gov/entrez/query.fcgi?cmd=Retrieve&db=Protein&list_uids=478428873&dopt=GenPept)
40. . [Chlamydia trachomatis L2/25667R](http://www.ncbi.nlm.nih.gov/Taxonomy/Browser/wwwtax.cgi?id=1071772) ............................... 101 [2 hits](http://blast.ncbi.nlm.nih.gov/Blast.cgi?CMD=Get&RID=5Y3VDS5A013&FORMAT_OBJECT=TaxBlast&NCBI_GI=off&DESCRIPTIONS=500&ALIGNMENTS=250&FORMAT_BLOCK_ON_RESPAGE=Top&MASK_COLOR=1&MASK_CHAR=2#1071772) [[chlamydias](http://www.ncbi.nlm.nih.gov/Taxonomy/Browser/wwwtax.cgi?id=51291)] [SET domain-containing protein [Chlamydia trachomatis A/HAR-](http://www.ncbi.nlm.nih.gov/entrez/query.fcgi?cmd=Retrieve&db=Protein&list_uids=478447579&dopt=GenPept)
41. . [Chlamydia trachomatis L1/440/LN](http://www.ncbi.nlm.nih.gov/Taxonomy/Browser/wwwtax.cgi?id=1071769) ............................... 101 [2 hits](http://blast.ncbi.nlm.nih.gov/Blast.cgi?CMD=Get&RID=5Y3VDS5A013&FORMAT_OBJECT=TaxBlast&NCBI_GI=off&DESCRIPTIONS=500&ALIGNMENTS=250&FORMAT_BLOCK_ON_RESPAGE=Top&MASK_COLOR=1&MASK_CHAR=2#1071769) [[chlamydias](http://www.ncbi.nlm.nih.gov/Taxonomy/Browser/wwwtax.cgi?id=51291)] [SET domain-containing protein [Chlamydia trachomatis A/HAR-](http://www.ncbi.nlm.nih.gov/entrez/query.fcgi?cmd=Retrieve&db=Protein&list_uids=478448472&dopt=GenPept)
42. . [Chlamydia trachomatis A/5291](http://www.ncbi.nlm.nih.gov/Taxonomy/Browser/wwwtax.cgi?id=1071754) .................................. 101 [2 hits](http://blast.ncbi.nlm.nih.gov/Blast.cgi?CMD=Get&RID=5Y3VDS5A013&FORMAT_OBJECT=TaxBlast&NCBI_GI=off&DESCRIPTIONS=500&ALIGNMENTS=250&FORMAT_BLOCK_ON_RESPAGE=Top&MASK_COLOR=1&MASK_CHAR=2#1071754) [[chlamydias](http://www.ncbi.nlm.nih.gov/Taxonomy/Browser/wwwtax.cgi?id=51291)] [SET domain-containing protein [Chlamydia trachomatis A/HAR-](http://www.ncbi.nlm.nih.gov/entrez/query.fcgi?cmd=Retrieve&db=Protein&list_uids=478449374&dopt=GenPept)
43. . [Chlamydia trachomatis L2b/Ams2](http://www.ncbi.nlm.nih.gov/Taxonomy/Browser/wwwtax.cgi?id=1071779) ................................ 101 [2 hits](http://blast.ncbi.nlm.nih.gov/Blast.cgi?CMD=Get&RID=5Y3VDS5A013&FORMAT_OBJECT=TaxBlast&NCBI_GI=off&DESCRIPTIONS=500&ALIGNMENTS=250&FORMAT_BLOCK_ON_RESPAGE=Top&MASK_COLOR=1&MASK_CHAR=2#1071779) [[chlamydias](http://www.ncbi.nlm.nih.gov/Taxonomy/Browser/wwwtax.cgi?id=51291)] [SET domain-containing protein [Chlamydia trachomatis A/HAR-](http://www.ncbi.nlm.nih.gov/entrez/query.fcgi?cmd=Retrieve&db=Protein&list_uids=478450270&dopt=GenPept)
44. . [Chlamydia trachomatis L2b/Ams5](http://www.ncbi.nlm.nih.gov/Taxonomy/Browser/wwwtax.cgi?id=1071782) ................................ 101 [2 hits](http://blast.ncbi.nlm.nih.gov/Blast.cgi?CMD=Get&RID=5Y3VDS5A013&FORMAT_OBJECT=TaxBlast&NCBI_GI=off&DESCRIPTIONS=500&ALIGNMENTS=250&FORMAT_BLOCK_ON_RESPAGE=Top&MASK_COLOR=1&MASK_CHAR=2#1071782) [[chlamydias](http://www.ncbi.nlm.nih.gov/Taxonomy/Browser/wwwtax.cgi?id=51291)] [SET domain-containing protein [Chlamydia trachomatis A/HAR-](http://www.ncbi.nlm.nih.gov/entrez/query.fcgi?cmd=Retrieve&db=Protein&list_uids=478451171&dopt=GenPept)
45. . [Chlamydia trachomatis L1/115](http://www.ncbi.nlm.nih.gov/Taxonomy/Browser/wwwtax.cgi?id=1071770) .................................. 101 [2 hits](http://blast.ncbi.nlm.nih.gov/Blast.cgi?CMD=Get&RID=5Y3VDS5A013&FORMAT_OBJECT=TaxBlast&NCBI_GI=off&DESCRIPTIONS=500&ALIGNMENTS=250&FORMAT_BLOCK_ON_RESPAGE=Top&MASK_COLOR=1&MASK_CHAR=2#1071770) [[chlamydias](http://www.ncbi.nlm.nih.gov/Taxonomy/Browser/wwwtax.cgi?id=51291)] [SET domain-containing protein [Chlamydia trachomatis A/HAR-](http://www.ncbi.nlm.nih.gov/entrez/query.fcgi?cmd=Retrieve&db=Protein&list_uids=478452071&dopt=GenPept)
46. . [Chlamydia trachomatis L2b/Canada1](http://www.ncbi.nlm.nih.gov/Taxonomy/Browser/wwwtax.cgi?id=1075085) ............................. 101 [2 hits](http://blast.ncbi.nlm.nih.gov/Blast.cgi?CMD=Get&RID=5Y3VDS5A013&FORMAT_OBJECT=TaxBlast&NCBI_GI=off&DESCRIPTIONS=500&ALIGNMENTS=250&FORMAT_BLOCK_ON_RESPAGE=Top&MASK_COLOR=1&MASK_CHAR=2#1075085) [[chlamydias](http://www.ncbi.nlm.nih.gov/Taxonomy/Browser/wwwtax.cgi?id=51291)] [SET domain-containing protein [Chlamydia trachomatis A/HAR-](http://www.ncbi.nlm.nih.gov/entrez/query.fcgi?cmd=Retrieve&db=Protein&list_uids=478452971&dopt=GenPept)
47. . [Chlamydia trachomatis L2b/Canada2](http://www.ncbi.nlm.nih.gov/Taxonomy/Browser/wwwtax.cgi?id=1075086) ............................. 101 [2 hits](http://blast.ncbi.nlm.nih.gov/Blast.cgi?CMD=Get&RID=5Y3VDS5A013&FORMAT_OBJECT=TaxBlast&NCBI_GI=off&DESCRIPTIONS=500&ALIGNMENTS=250&FORMAT_BLOCK_ON_RESPAGE=Top&MASK_COLOR=1&MASK_CHAR=2#1075086) [[chlamydias](http://www.ncbi.nlm.nih.gov/Taxonomy/Browser/wwwtax.cgi?id=51291)] [SET domain-containing protein [Chlamydia trachomatis A/HAR-](http://www.ncbi.nlm.nih.gov/entrez/query.fcgi?cmd=Retrieve&db=Protein&list_uids=478453884&dopt=GenPept)
48. . [Chlamydia trachomatis L2b/UCH-2](http://www.ncbi.nlm.nih.gov/Taxonomy/Browser/wwwtax.cgi?id=1071774) ............................... 101 [2 hits](http://blast.ncbi.nlm.nih.gov/Blast.cgi?CMD=Get&RID=5Y3VDS5A013&FORMAT_OBJECT=TaxBlast&NCBI_GI=off&DESCRIPTIONS=500&ALIGNMENTS=250&FORMAT_BLOCK_ON_RESPAGE=Top&MASK_COLOR=1&MASK_CHAR=2#1071774) [[chlamydias](http://www.ncbi.nlm.nih.gov/Taxonomy/Browser/wwwtax.cgi?id=51291)] [SET domain-containing protein [Chlamydia trachomatis A/HAR-](http://www.ncbi.nlm.nih.gov/entrez/query.fcgi?cmd=Retrieve&db=Protein&list_uids=478454776&dopt=GenPept)
49. . [Chlamydia trachomatis E/SotonE8](http://www.ncbi.nlm.nih.gov/Taxonomy/Browser/wwwtax.cgi?id=1071761) ............................... 101 [2 hits](http://blast.ncbi.nlm.nih.gov/Blast.cgi?CMD=Get&RID=5Y3VDS5A013&FORMAT_OBJECT=TaxBlast&NCBI_GI=off&DESCRIPTIONS=500&ALIGNMENTS=250&FORMAT_BLOCK_ON_RESPAGE=Top&MASK_COLOR=1&MASK_CHAR=2#1071761) [[chlamydias](http://www.ncbi.nlm.nih.gov/Taxonomy/Browser/wwwtax.cgi?id=51291)] [SET domain-containing protein [Chlamydia trachomatis A/HAR-](http://www.ncbi.nlm.nih.gov/entrez/query.fcgi?cmd=Retrieve&db=Protein&list_uids=478456597&dopt=GenPept)
50. . [Chlamydia trachomatis K/SotonK1](http://www.ncbi.nlm.nih.gov/Taxonomy/Browser/wwwtax.cgi?id=1071768) ............................... 101 [2 hits](http://blast.ncbi.nlm.nih.gov/Blast.cgi?CMD=Get&RID=5Y3VDS5A013&FORMAT_OBJECT=TaxBlast&NCBI_GI=off&DESCRIPTIONS=500&ALIGNMENTS=250&FORMAT_BLOCK_ON_RESPAGE=Top&MASK_COLOR=1&MASK_CHAR=2#1071768) [[chlamydias](http://www.ncbi.nlm.nih.gov/Taxonomy/Browser/wwwtax.cgi?id=51291)] [SET domain-containing protein [Chlamydia trachomatis A/HAR-](http://www.ncbi.nlm.nih.gov/entrez/query.fcgi?cmd=Retrieve&db=Protein&list_uids=478458400&dopt=GenPept)
51. . [Chlamydia trachomatis A/363](http://www.ncbi.nlm.nih.gov/Taxonomy/Browser/wwwtax.cgi?id=1071753) ................................... 101 [2 hits](http://blast.ncbi.nlm.nih.gov/Blast.cgi?CMD=Get&RID=5Y3VDS5A013&FORMAT_OBJECT=TaxBlast&NCBI_GI=off&DESCRIPTIONS=500&ALIGNMENTS=250&FORMAT_BLOCK_ON_RESPAGE=Top&MASK_COLOR=1&MASK_CHAR=2#1071753) [[chlamydias](http://www.ncbi.nlm.nih.gov/Taxonomy/Browser/wwwtax.cgi?id=51291)] [SET domain-containing protein [Chlamydia trachomatis A/HAR-](http://www.ncbi.nlm.nih.gov/entrez/query.fcgi?cmd=Retrieve&db=Protein&list_uids=478459302&dopt=GenPept)
52. . [Chlamydia trachomatis D/SotonD6](http://www.ncbi.nlm.nih.gov/Taxonomy/Browser/wwwtax.cgi?id=1071758) ............................... 101 [2 hits](http://blast.ncbi.nlm.nih.gov/Blast.cgi?CMD=Get&RID=5Y3VDS5A013&FORMAT_OBJECT=TaxBlast&NCBI_GI=off&DESCRIPTIONS=500&ALIGNMENTS=250&FORMAT_BLOCK_ON_RESPAGE=Top&MASK_COLOR=1&MASK_CHAR=2#1071758) [[chlamydias](http://www.ncbi.nlm.nih.gov/Taxonomy/Browser/wwwtax.cgi?id=51291)] [SET domain-containing protein [Chlamydia trachomatis A/HAR-](http://www.ncbi.nlm.nih.gov/entrez/query.fcgi?cmd=Retrieve&db=Protein&list_uids=478460203&dopt=GenPept)
53. . [Chlamydia trachomatis Ia/SotonIa1](http://www.ncbi.nlm.nih.gov/Taxonomy/Browser/wwwtax.cgi?id=1071766) ............................. 101 [2 hits](http://blast.ncbi.nlm.nih.gov/Blast.cgi?CMD=Get&RID=5Y3VDS5A013&FORMAT_OBJECT=TaxBlast&NCBI_GI=off&DESCRIPTIONS=500&ALIGNMENTS=250&FORMAT_BLOCK_ON_RESPAGE=Top&MASK_COLOR=1&MASK_CHAR=2#1071766) [[chlamydias](http://www.ncbi.nlm.nih.gov/Taxonomy/Browser/wwwtax.cgi?id=51291)] [SET domain-containing protein [Chlamydia trachomatis A/HAR-](http://www.ncbi.nlm.nih.gov/entrez/query.fcgi?cmd=Retrieve&db=Protein&list_uids=478461107&dopt=GenPept)
54. . [Chlamydia trachomatis E/Bour](http://www.ncbi.nlm.nih.gov/Taxonomy/Browser/wwwtax.cgi?id=596777) .................................. 101 [2 hits](http://blast.ncbi.nlm.nih.gov/Blast.cgi?CMD=Get&RID=5Y3VDS5A013&FORMAT_OBJECT=TaxBlast&NCBI_GI=off&DESCRIPTIONS=500&ALIGNMENTS=250&FORMAT_BLOCK_ON_RESPAGE=Top&MASK_COLOR=1&MASK_CHAR=2#596777) [[chlamydias](http://www.ncbi.nlm.nih.gov/Taxonomy/Browser/wwwtax.cgi?id=51291)] [SET domain-containing protein [Chlamydia trachomatis A/HAR-](http://www.ncbi.nlm.nih.gov/entrez/query.fcgi?cmd=Retrieve&db=Protein&list_uids=478462003&dopt=GenPept)
55. . [Chlamydia trachomatis L1/224](http://www.ncbi.nlm.nih.gov/Taxonomy/Browser/wwwtax.cgi?id=1071771) .................................. 101 [2 hits](http://blast.ncbi.nlm.nih.gov/Blast.cgi?CMD=Get&RID=5Y3VDS5A013&FORMAT_OBJECT=TaxBlast&NCBI_GI=off&DESCRIPTIONS=500&ALIGNMENTS=250&FORMAT_BLOCK_ON_RESPAGE=Top&MASK_COLOR=1&MASK_CHAR=2#1071771) [[chlamydias](http://www.ncbi.nlm.nih.gov/Taxonomy/Browser/wwwtax.cgi?id=51291)] [SET domain-containing protein [Chlamydia trachomatis A/HAR-](http://www.ncbi.nlm.nih.gov/entrez/query.fcgi?cmd=Retrieve&db=Protein&list_uids=478462910&dopt=GenPept)
56. . [Chlamydia trachomatis L3/404/LN](http://www.ncbi.nlm.nih.gov/Taxonomy/Browser/wwwtax.cgi?id=1071783) ............................... 101 [2 hits](http://blast.ncbi.nlm.nih.gov/Blast.cgi?CMD=Get&RID=5Y3VDS5A013&FORMAT_OBJECT=TaxBlast&NCBI_GI=off&DESCRIPTIONS=500&ALIGNMENTS=250&FORMAT_BLOCK_ON_RESPAGE=Top&MASK_COLOR=1&MASK_CHAR=2#1071783) [[chlamydias](http://www.ncbi.nlm.nih.gov/Taxonomy/Browser/wwwtax.cgi?id=51291)] [SET domain-containing protein [Chlamydia trachomatis A/HAR-](http://www.ncbi.nlm.nih.gov/entrez/query.fcgi?cmd=Retrieve&db=Protein&list_uids=478463808&dopt=GenPept)
57. . [Chlamydia trachomatis L2b/CV204](http://www.ncbi.nlm.nih.gov/Taxonomy/Browser/wwwtax.cgi?id=1071776) ............................... 101 [2 hits](http://blast.ncbi.nlm.nih.gov/Blast.cgi?CMD=Get&RID=5Y3VDS5A013&FORMAT_OBJECT=TaxBlast&NCBI_GI=off&DESCRIPTIONS=500&ALIGNMENTS=250&FORMAT_BLOCK_ON_RESPAGE=Top&MASK_COLOR=1&MASK_CHAR=2#1071776) [[chlamydias](http://www.ncbi.nlm.nih.gov/Taxonomy/Browser/wwwtax.cgi?id=51291)] [SET domain-containing protein [Chlamydia trachomatis A/HAR-](http://www.ncbi.nlm.nih.gov/entrez/query.fcgi?cmd=Retrieve&db=Protein&list_uids=478464706&dopt=GenPept)
58. . [Chlamydia trachomatis L2b/Ams3](http://www.ncbi.nlm.nih.gov/Taxonomy/Browser/wwwtax.cgi?id=1071780) ................................ 101 [2 hits](http://blast.ncbi.nlm.nih.gov/Blast.cgi?CMD=Get&RID=5Y3VDS5A013&FORMAT_OBJECT=TaxBlast&NCBI_GI=off&DESCRIPTIONS=500&ALIGNMENTS=250&FORMAT_BLOCK_ON_RESPAGE=Top&MASK_COLOR=1&MASK_CHAR=2#1071780) [[chlamydias](http://www.ncbi.nlm.nih.gov/Taxonomy/Browser/wwwtax.cgi?id=51291)] [SET domain-containing protein [Chlamydia trachomatis A/HAR-](http://www.ncbi.nlm.nih.gov/entrez/query.fcgi?cmd=Retrieve&db=Protein&list_uids=478465606&dopt=GenPept)
59. . [Chlamydia trachomatis A/7249](http://www.ncbi.nlm.nih.gov/Taxonomy/Browser/wwwtax.cgi?id=1071755) .................................. 101 [2 hits](http://blast.ncbi.nlm.nih.gov/Blast.cgi?CMD=Get&RID=5Y3VDS5A013&FORMAT_OBJECT=TaxBlast&NCBI_GI=off&DESCRIPTIONS=500&ALIGNMENTS=250&FORMAT_BLOCK_ON_RESPAGE=Top&MASK_COLOR=1&MASK_CHAR=2#1071755) [[chlamydias](http://www.ncbi.nlm.nih.gov/Taxonomy/Browser/wwwtax.cgi?id=51291)] [SET domain-containing protein [Chlamydia trachomatis A/HAR-](http://www.ncbi.nlm.nih.gov/entrez/query.fcgi?cmd=Retrieve&db=Protein&list_uids=478466518&dopt=GenPept)
60. . [Chlamydia trachomatis L2b/Ams1](http://www.ncbi.nlm.nih.gov/Taxonomy/Browser/wwwtax.cgi?id=1071778) ................................ 101 [2 hits](http://blast.ncbi.nlm.nih.gov/Blast.cgi?CMD=Get&RID=5Y3VDS5A013&FORMAT_OBJECT=TaxBlast&NCBI_GI=off&DESCRIPTIONS=500&ALIGNMENTS=250&FORMAT_BLOCK_ON_RESPAGE=Top&MASK_COLOR=1&MASK_CHAR=2#1071778) [[chlamydias](http://www.ncbi.nlm.nih.gov/Taxonomy/Browser/wwwtax.cgi?id=51291)] [SET domain-containing protein [Chlamydia trachomatis A/HAR-](http://www.ncbi.nlm.nih.gov/entrez/query.fcgi?cmd=Retrieve&db=Protein&list_uids=478467405&dopt=GenPept)
61. . [Chlamydia trachomatis L2b/Ams4](http://www.ncbi.nlm.nih.gov/Taxonomy/Browser/wwwtax.cgi?id=1071781) ................................ 101 [2 hits](http://blast.ncbi.nlm.nih.gov/Blast.cgi?CMD=Get&RID=5Y3VDS5A013&FORMAT_OBJECT=TaxBlast&NCBI_GI=off&DESCRIPTIONS=500&ALIGNMENTS=250&FORMAT_BLOCK_ON_RESPAGE=Top&MASK_COLOR=1&MASK_CHAR=2#1071781) [[chlamydias](http://www.ncbi.nlm.nih.gov/Taxonomy/Browser/wwwtax.cgi?id=51291)] [SET domain-containing protein [Chlamydia trachomatis A/HAR-](http://www.ncbi.nlm.nih.gov/entrez/query.fcgi?cmd=Retrieve&db=Protein&list_uids=478468305&dopt=GenPept)
62. . [Chlamydia trachomatis L1/1322/p2](http://www.ncbi.nlm.nih.gov/Taxonomy/Browser/wwwtax.cgi?id=1075087) .............................. 101 [2 hits](http://blast.ncbi.nlm.nih.gov/Blast.cgi?CMD=Get&RID=5Y3VDS5A013&FORMAT_OBJECT=TaxBlast&NCBI_GI=off&DESCRIPTIONS=500&ALIGNMENTS=250&FORMAT_BLOCK_ON_RESPAGE=Top&MASK_COLOR=1&MASK_CHAR=2#1075087) [[chlamydias](http://www.ncbi.nlm.nih.gov/Taxonomy/Browser/wwwtax.cgi?id=51291)] [SET domain-containing protein [Chlamydia trachomatis A/HAR-](http://www.ncbi.nlm.nih.gov/entrez/query.fcgi?cmd=Retrieve&db=Protein&list_uids=478469215&dopt=GenPept)
63. . [Chlamydia trachomatis L2b/795](http://www.ncbi.nlm.nih.gov/Taxonomy/Browser/wwwtax.cgi?id=1071777) ................................. 101 [2 hits](http://blast.ncbi.nlm.nih.gov/Blast.cgi?CMD=Get&RID=5Y3VDS5A013&FORMAT_OBJECT=TaxBlast&NCBI_GI=off&DESCRIPTIONS=500&ALIGNMENTS=250&FORMAT_BLOCK_ON_RESPAGE=Top&MASK_COLOR=1&MASK_CHAR=2#1071777) [[chlamydias](http://www.ncbi.nlm.nih.gov/Taxonomy/Browser/wwwtax.cgi?id=51291)] [SET domain-containing protein [Chlamydia trachomatis A/HAR-](http://www.ncbi.nlm.nih.gov/entrez/query.fcgi?cmd=Retrieve&db=Protein&list_uids=478470114&dopt=GenPept)
64. . [Chlamydia trachomatis L2b/8200/07](http://www.ncbi.nlm.nih.gov/Taxonomy/Browser/wwwtax.cgi?id=1071773) ............................. 101 [2 hits](http://blast.ncbi.nlm.nih.gov/Blast.cgi?CMD=Get&RID=5Y3VDS5A013&FORMAT_OBJECT=TaxBlast&NCBI_GI=off&DESCRIPTIONS=500&ALIGNMENTS=250&FORMAT_BLOCK_ON_RESPAGE=Top&MASK_COLOR=1&MASK_CHAR=2#1071773) [[chlamydias](http://www.ncbi.nlm.nih.gov/Taxonomy/Browser/wwwtax.cgi?id=51291)] [SET domain-containing protein [Chlamydia trachomatis A/HAR-](http://www.ncbi.nlm.nih.gov/entrez/query.fcgi?cmd=Retrieve&db=Protein&list_uids=478471014&dopt=GenPept)
65. . [Chlamydia trachomatis L2b/LST](http://www.ncbi.nlm.nih.gov/Taxonomy/Browser/wwwtax.cgi?id=1071775) ................................. 101 [2 hits](http://blast.ncbi.nlm.nih.gov/Blast.cgi?CMD=Get&RID=5Y3VDS5A013&FORMAT_OBJECT=TaxBlast&NCBI_GI=off&DESCRIPTIONS=500&ALIGNMENTS=250&FORMAT_BLOCK_ON_RESPAGE=Top&MASK_COLOR=1&MASK_CHAR=2#1071775) [[chlamydias](http://www.ncbi.nlm.nih.gov/Taxonomy/Browser/wwwtax.cgi?id=51291)] [SET domain-containing protein [Chlamydia trachomatis A/HAR-](http://www.ncbi.nlm.nih.gov/entrez/query.fcgi?cmd=Retrieve&db=Protein&list_uids=478471905&dopt=GenPept)
66. . [Chlamydia trachomatis D/SotonD1](http://www.ncbi.nlm.nih.gov/Taxonomy/Browser/wwwtax.cgi?id=1071756) ............................... 101 [2 hits](http://blast.ncbi.nlm.nih.gov/Blast.cgi?CMD=Get&RID=5Y3VDS5A013&FORMAT_OBJECT=TaxBlast&NCBI_GI=off&DESCRIPTIONS=500&ALIGNMENTS=250&FORMAT_BLOCK_ON_RESPAGE=Top&MASK_COLOR=1&MASK_CHAR=2#1071756) [[chlamydias](http://www.ncbi.nlm.nih.gov/Taxonomy/Browser/wwwtax.cgi?id=51291)] [SET domain-containing protein [Chlamydia trachomatis A/HAR-](http://www.ncbi.nlm.nih.gov/entrez/query.fcgi?cmd=Retrieve&db=Protein&list_uids=478472812&dopt=GenPept)
67. . [Chlamydia trachomatis E/SotonE4](http://www.ncbi.nlm.nih.gov/Taxonomy/Browser/wwwtax.cgi?id=1071760) ............................... 101 [2 hits](http://blast.ncbi.nlm.nih.gov/Blast.cgi?CMD=Get&RID=5Y3VDS5A013&FORMAT_OBJECT=TaxBlast&NCBI_GI=off&DESCRIPTIONS=500&ALIGNMENTS=250&FORMAT_BLOCK_ON_RESPAGE=Top&MASK_COLOR=1&MASK_CHAR=2#1071760) [[chlamydias](http://www.ncbi.nlm.nih.gov/Taxonomy/Browser/wwwtax.cgi?id=51291)] [SET domain-containing protein [Chlamydia trachomatis A/HAR-](http://www.ncbi.nlm.nih.gov/entrez/query.fcgi?cmd=Retrieve&db=Protein&list_uids=478473713&dopt=GenPept)
68. . [Chlamydia trachomatis F/SotonF3](http://www.ncbi.nlm.nih.gov/Taxonomy/Browser/wwwtax.cgi?id=1071764) ............................... 101 [2 hits](http://blast.ncbi.nlm.nih.gov/Blast.cgi?CMD=Get&RID=5Y3VDS5A013&FORMAT_OBJECT=TaxBlast&NCBI_GI=off&DESCRIPTIONS=500&ALIGNMENTS=250&FORMAT_BLOCK_ON_RESPAGE=Top&MASK_COLOR=1&MASK_CHAR=2#1071764) [[chlamydias](http://www.ncbi.nlm.nih.gov/Taxonomy/Browser/wwwtax.cgi?id=51291)] [SET domain-containing protein [Chlamydia trachomatis A/HAR-](http://www.ncbi.nlm.nih.gov/entrez/query.fcgi?cmd=Retrieve&db=Protein&list_uids=478474617&dopt=GenPept)
69. . [Chlamydia trachomatis Ia/SotonIa3](http://www.ncbi.nlm.nih.gov/Taxonomy/Browser/wwwtax.cgi?id=1071767) ............................. 101 [2 hits](http://blast.ncbi.nlm.nih.gov/Blast.cgi?CMD=Get&RID=5Y3VDS5A013&FORMAT_OBJECT=TaxBlast&NCBI_GI=off&DESCRIPTIONS=500&ALIGNMENTS=250&FORMAT_BLOCK_ON_RESPAGE=Top&MASK_COLOR=1&MASK_CHAR=2#1071767) [[chlamydias](http://www.ncbi.nlm.nih.gov/Taxonomy/Browser/wwwtax.cgi?id=51291)] [SET domain-containing protein [Chlamydia trachomatis A/HAR-](http://www.ncbi.nlm.nih.gov/entrez/query.fcgi?cmd=Retrieve&db=Protein&list_uids=478475522&dopt=GenPept)
70. . [Chlamydia trachomatis L2/434/Bu(i)](http://www.ncbi.nlm.nih.gov/Taxonomy/Browser/wwwtax.cgi?id=1263406) ............................ 101 [2 hits](http://blast.ncbi.nlm.nih.gov/Blast.cgi?CMD=Get&RID=5Y3VDS5A013&FORMAT_OBJECT=TaxBlast&NCBI_GI=off&DESCRIPTIONS=500&ALIGNMENTS=250&FORMAT_BLOCK_ON_RESPAGE=Top&MASK_COLOR=1&MASK_CHAR=2#1263406) [[chlamydias](http://www.ncbi.nlm.nih.gov/Taxonomy/Browser/wwwtax.cgi?id=51291)] [SET domain-containing protein [Chlamydia trachomatis A/HAR-](http://www.ncbi.nlm.nih.gov/entrez/query.fcgi?cmd=Retrieve&db=Protein&list_uids=482545198&dopt=GenPept)
71. . [Chlamydia trachomatis L2/434/Bu(f)](http://www.ncbi.nlm.nih.gov/Taxonomy/Browser/wwwtax.cgi?id=1262673) ............................ 101 [2 hits](http://blast.ncbi.nlm.nih.gov/Blast.cgi?CMD=Get&RID=5Y3VDS5A013&FORMAT_OBJECT=TaxBlast&NCBI_GI=off&DESCRIPTIONS=500&ALIGNMENTS=250&FORMAT_BLOCK_ON_RESPAGE=Top&MASK_COLOR=1&MASK_CHAR=2#1262673) [[chlamydias](http://www.ncbi.nlm.nih.gov/Taxonomy/Browser/wwwtax.cgi?id=51291)] [SET domain-containing protein [Chlamydia trachomatis A/HAR-](http://www.ncbi.nlm.nih.gov/entrez/query.fcgi?cmd=Retrieve&db=Protein&list_uids=482546140&dopt=GenPept)
72. . [Chlamydia trachomatis RC-L2(s)/46](http://www.ncbi.nlm.nih.gov/Taxonomy/Browser/wwwtax.cgi?id=907264) ............................. 101 [2 hits](http://blast.ncbi.nlm.nih.gov/Blast.cgi?CMD=Get&RID=5Y3VDS5A013&FORMAT_OBJECT=TaxBlast&NCBI_GI=off&DESCRIPTIONS=500&ALIGNMENTS=250&FORMAT_BLOCK_ON_RESPAGE=Top&MASK_COLOR=1&MASK_CHAR=2#907264) [[chlamydias](http://www.ncbi.nlm.nih.gov/Taxonomy/Browser/wwwtax.cgi?id=51291)] [SET domain-containing protein [Chlamydia trachomatis A/HAR-](http://www.ncbi.nlm.nih.gov/entrez/query.fcgi?cmd=Retrieve&db=Protein&list_uids=527318583&dopt=GenPept)
73. . [Chlamydia trachomatis RC-F(s)/852](http://www.ncbi.nlm.nih.gov/Taxonomy/Browser/wwwtax.cgi?id=907265) ............................. 101 [2 hits](http://blast.ncbi.nlm.nih.gov/Blast.cgi?CMD=Get&RID=5Y3VDS5A013&FORMAT_OBJECT=TaxBlast&NCBI_GI=off&DESCRIPTIONS=500&ALIGNMENTS=250&FORMAT_BLOCK_ON_RESPAGE=Top&MASK_COLOR=1&MASK_CHAR=2#907265) [[chlamydias](http://www.ncbi.nlm.nih.gov/Taxonomy/Browser/wwwtax.cgi?id=51291)] [SET domain-containing protein [Chlamydia trachomatis A/HAR-](http://www.ncbi.nlm.nih.gov/entrez/query.fcgi?cmd=Retrieve&db=Protein&list_uids=527319509&dopt=GenPept)
74. . [Chlamydia trachomatis RC-J/953](http://www.ncbi.nlm.nih.gov/Taxonomy/Browser/wwwtax.cgi?id=907267) ................................ 101 [2 hits](http://blast.ncbi.nlm.nih.gov/Blast.cgi?CMD=Get&RID=5Y3VDS5A013&FORMAT_OBJECT=TaxBlast&NCBI_GI=off&DESCRIPTIONS=500&ALIGNMENTS=250&FORMAT_BLOCK_ON_RESPAGE=Top&MASK_COLOR=1&MASK_CHAR=2#907267) [[chlamydias](http://www.ncbi.nlm.nih.gov/Taxonomy/Browser/wwwtax.cgi?id=51291)] [SET domain-containing protein [Chlamydia trachomatis A/HAR-](http://www.ncbi.nlm.nih.gov/entrez/query.fcgi?cmd=Retrieve&db=Protein&list_uids=527320459&dopt=GenPept)
75. . [Chlamydia trachomatis RC-F(s)/342](http://www.ncbi.nlm.nih.gov/Taxonomy/Browser/wwwtax.cgi?id=907269) ............................. 101 [2 hits](http://blast.ncbi.nlm.nih.gov/Blast.cgi?CMD=Get&RID=5Y3VDS5A013&FORMAT_OBJECT=TaxBlast&NCBI_GI=off&DESCRIPTIONS=500&ALIGNMENTS=250&FORMAT_BLOCK_ON_RESPAGE=Top&MASK_COLOR=1&MASK_CHAR=2#907269) [[chlamydias](http://www.ncbi.nlm.nih.gov/Taxonomy/Browser/wwwtax.cgi?id=51291)] [SET domain-containing protein [Chlamydia trachomatis A/HAR-](http://www.ncbi.nlm.nih.gov/entrez/query.fcgi?cmd=Retrieve&db=Protein&list_uids=527321383&dopt=GenPept)
76. . [Chlamydia trachomatis RC-J(s)/122](http://www.ncbi.nlm.nih.gov/Taxonomy/Browser/wwwtax.cgi?id=907270) ............................. 101 [2 hits](http://blast.ncbi.nlm.nih.gov/Blast.cgi?CMD=Get&RID=5Y3VDS5A013&FORMAT_OBJECT=TaxBlast&NCBI_GI=off&DESCRIPTIONS=500&ALIGNMENTS=250&FORMAT_BLOCK_ON_RESPAGE=Top&MASK_COLOR=1&MASK_CHAR=2#907270) [[chlamydias](http://www.ncbi.nlm.nih.gov/Taxonomy/Browser/wwwtax.cgi?id=51291)] [SET domain-containing protein [Chlamydia trachomatis A/HAR-](http://www.ncbi.nlm.nih.gov/entrez/query.fcgi?cmd=Retrieve&db=Protein&list_uids=527322329&dopt=GenPept)
77. . [Chlamydia trachomatis J/6276tet1](http://www.ncbi.nlm.nih.gov/Taxonomy/Browser/wwwtax.cgi?id=907272) .............................. 101 [2 hits](http://blast.ncbi.nlm.nih.gov/Blast.cgi?CMD=Get&RID=5Y3VDS5A013&FORMAT_OBJECT=TaxBlast&NCBI_GI=off&DESCRIPTIONS=500&ALIGNMENTS=250&FORMAT_BLOCK_ON_RESPAGE=Top&MASK_COLOR=1&MASK_CHAR=2#907272) [[chlamydias](http://www.ncbi.nlm.nih.gov/Taxonomy/Browser/wwwtax.cgi?id=51291)] [SET domain-containing protein [Chlamydia trachomatis A/HAR-](http://www.ncbi.nlm.nih.gov/entrez/query.fcgi?cmd=Retrieve&db=Protein&list_uids=527323275&dopt=GenPept)
78. . [Chlamydia trachomatis RC-L2/55](http://www.ncbi.nlm.nih.gov/Taxonomy/Browser/wwwtax.cgi?id=1007870) ................................ 101 [2 hits](http://blast.ncbi.nlm.nih.gov/Blast.cgi?CMD=Get&RID=5Y3VDS5A013&FORMAT_OBJECT=TaxBlast&NCBI_GI=off&DESCRIPTIONS=500&ALIGNMENTS=250&FORMAT_BLOCK_ON_RESPAGE=Top&MASK_COLOR=1&MASK_CHAR=2#1007870) [[chlamydias](http://www.ncbi.nlm.nih.gov/Taxonomy/Browser/wwwtax.cgi?id=51291)] [SET domain-containing protein [Chlamydia trachomatis A/HAR-](http://www.ncbi.nlm.nih.gov/entrez/query.fcgi?cmd=Retrieve&db=Protein&list_uids=527324232&dopt=GenPept)
79. . [Chlamydia trachomatis RC-F/69](http://www.ncbi.nlm.nih.gov/Taxonomy/Browser/wwwtax.cgi?id=907263) ................................. 101 [2 hits](http://blast.ncbi.nlm.nih.gov/Blast.cgi?CMD=Get&RID=5Y3VDS5A013&FORMAT_OBJECT=TaxBlast&NCBI_GI=off&DESCRIPTIONS=500&ALIGNMENTS=250&FORMAT_BLOCK_ON_RESPAGE=Top&MASK_COLOR=1&MASK_CHAR=2#907263) [[chlamydias](http://www.ncbi.nlm.nih.gov/Taxonomy/Browser/wwwtax.cgi?id=51291)] [SET domain-containing protein [Chlamydia trachomatis A/HAR-](http://www.ncbi.nlm.nih.gov/entrez/query.fcgi?cmd=Retrieve&db=Protein&list_uids=527325365&dopt=GenPept)
80. . [Chlamydia trachomatis RC-J/943](http://www.ncbi.nlm.nih.gov/Taxonomy/Browser/wwwtax.cgi?id=907266) ................................ 101 [2 hits](http://blast.ncbi.nlm.nih.gov/Blast.cgi?CMD=Get&RID=5Y3VDS5A013&FORMAT_OBJECT=TaxBlast&NCBI_GI=off&DESCRIPTIONS=500&ALIGNMENTS=250&FORMAT_BLOCK_ON_RESPAGE=Top&MASK_COLOR=1&MASK_CHAR=2#907266) [[chlamydias](http://www.ncbi.nlm.nih.gov/Taxonomy/Browser/wwwtax.cgi?id=51291)] [SET domain-containing protein [Chlamydia trachomatis A/HAR-](http://www.ncbi.nlm.nih.gov/entrez/query.fcgi?cmd=Retrieve&db=Protein&list_uids=527326291&dopt=GenPept)
81. . [Chlamydia trachomatis RC-L2(s)/3](http://www.ncbi.nlm.nih.gov/Taxonomy/Browser/wwwtax.cgi?id=907268) .............................. 101 [2 hits](http://blast.ncbi.nlm.nih.gov/Blast.cgi?CMD=Get&RID=5Y3VDS5A013&FORMAT_OBJECT=TaxBlast&NCBI_GI=off&DESCRIPTIONS=500&ALIGNMENTS=250&FORMAT_BLOCK_ON_RESPAGE=Top&MASK_COLOR=1&MASK_CHAR=2#907268) [[chlamydias](http://www.ncbi.nlm.nih.gov/Taxonomy/Browser/wwwtax.cgi?id=51291)] [SET domain-containing protein [Chlamydia trachomatis A/HAR-](http://www.ncbi.nlm.nih.gov/entrez/query.fcgi?cmd=Retrieve&db=Protein&list_uids=527327213&dopt=GenPept)
82. . [Chlamydia trachomatis RC-J/966](http://www.ncbi.nlm.nih.gov/Taxonomy/Browser/wwwtax.cgi?id=907271) ................................ 101 [2 hits](http://blast.ncbi.nlm.nih.gov/Blast.cgi?CMD=Get&RID=5Y3VDS5A013&FORMAT_OBJECT=TaxBlast&NCBI_GI=off&DESCRIPTIONS=500&ALIGNMENTS=250&FORMAT_BLOCK_ON_RESPAGE=Top&MASK_COLOR=1&MASK_CHAR=2#907271) [[chlamydias](http://www.ncbi.nlm.nih.gov/Taxonomy/Browser/wwwtax.cgi?id=51291)] [SET domain-containing protein [Chlamydia trachomatis A/HAR-](http://www.ncbi.nlm.nih.gov/entrez/query.fcgi?cmd=Retrieve&db=Protein&list_uids=527328138&dopt=GenPept)
83. . [Chlamydia trachomatis RC-J/971](http://www.ncbi.nlm.nih.gov/Taxonomy/Browser/wwwtax.cgi?id=1007871) ................................ 101 [2 hits](http://blast.ncbi.nlm.nih.gov/Blast.cgi?CMD=Get&RID=5Y3VDS5A013&FORMAT_OBJECT=TaxBlast&NCBI_GI=off&DESCRIPTIONS=500&ALIGNMENTS=250&FORMAT_BLOCK_ON_RESPAGE=Top&MASK_COLOR=1&MASK_CHAR=2#1007871) [[chlamydias](http://www.ncbi.nlm.nih.gov/Taxonomy/Browser/wwwtax.cgi?id=51291)] [SET domain-containing protein [Chlamydia trachomatis A/HAR-](http://www.ncbi.nlm.nih.gov/entrez/query.fcgi?cmd=Retrieve&db=Protein&list_uids=527329064&dopt=GenPept)
84. . [Chlamydia trachomatis](http://www.ncbi.nlm.nih.gov/Taxonomy/Browser/wwwtax.cgi?id=813) ......................................... 101 [20 hits](http://blast.ncbi.nlm.nih.gov/Blast.cgi?CMD=Get&RID=5Y3VDS5A013&FORMAT_OBJECT=TaxBlast&NCBI_GI=off&DESCRIPTIONS=500&ALIGNMENTS=250&FORMAT_BLOCK_ON_RESPAGE=Top&MASK_COLOR=1&MASK_CHAR=2#813) [[chlamydias](http://www.ncbi.nlm.nih.gov/Taxonomy/Browser/wwwtax.cgi?id=51291)] [SET domain-containing protein [Chlamydia trachomatis A/HAR-](http://www.ncbi.nlm.nih.gov/entrez/query.fcgi?cmd=Retrieve&db=Protein&list_uids=532350215&dopt=GenPept)
85. . [Chlamydia trachomatis F/11-96](http://www.ncbi.nlm.nih.gov/Taxonomy/Browser/wwwtax.cgi?id=1340853) ................................. 101 [2 hits](http://blast.ncbi.nlm.nih.gov/Blast.cgi?CMD=Get&RID=5Y3VDS5A013&FORMAT_OBJECT=TaxBlast&NCBI_GI=off&DESCRIPTIONS=500&ALIGNMENTS=250&FORMAT_BLOCK_ON_RESPAGE=Top&MASK_COLOR=1&MASK_CHAR=2#1340853) [[chlamydias](http://www.ncbi.nlm.nih.gov/Taxonomy/Browser/wwwtax.cgi?id=51291)] [SET domain-containing protein [Chlamydia trachomatis A/HAR-](http://www.ncbi.nlm.nih.gov/entrez/query.fcgi?cmd=Retrieve&db=Protein&list_uids=532351141&dopt=GenPept)
86. . [Chlamydia trachomatis E/C599](http://www.ncbi.nlm.nih.gov/Taxonomy/Browser/wwwtax.cgi?id=1100832) .................................. 101 [1 hit](http://blast.ncbi.nlm.nih.gov/Blast.cgi?CMD=Get&RID=5Y3VDS5A013&FORMAT_OBJECT=TaxBlast&NCBI_GI=off&DESCRIPTIONS=500&ALIGNMENTS=250&FORMAT_BLOCK_ON_RESPAGE=Top&MASK_COLOR=1&MASK_CHAR=2#1100832) [[chlamydias](http://www.ncbi.nlm.nih.gov/Taxonomy/Browser/wwwtax.cgi?id=51291)] [SET domain-containing protein [Chlamydia trachomatis A/HAR-](http://www.ncbi.nlm.nih.gov/entrez/query.fcgi?cmd=Retrieve&db=Protein&list_uids=549136285&dopt=GenPept)
87. . [Chlamydia trachomatis F/SWFPminus](http://www.ncbi.nlm.nih.gov/Taxonomy/Browser/wwwtax.cgi?id=1100833) ............................. 101 [1 hit](http://blast.ncbi.nlm.nih.gov/Blast.cgi?CMD=Get&RID=5Y3VDS5A013&FORMAT_OBJECT=TaxBlast&NCBI_GI=off&DESCRIPTIONS=500&ALIGNMENTS=250&FORMAT_BLOCK_ON_RESPAGE=Top&MASK_COLOR=1&MASK_CHAR=2#1100833) [[chlamydias](http://www.ncbi.nlm.nih.gov/Taxonomy/Browser/wwwtax.cgi?id=51291)] [SET domain-containing protein [Chlamydia trachomatis A/HAR-](http://www.ncbi.nlm.nih.gov/entrez/query.fcgi?cmd=Retrieve&db=Protein&list_uids=549137239&dopt=GenPept)
88. . [Chlamydia trachomatis D/UW-3/CX](http://www.ncbi.nlm.nih.gov/Taxonomy/Browser/wwwtax.cgi?id=272561) ............................... 101 [2 hits](http://blast.ncbi.nlm.nih.gov/Blast.cgi?CMD=Get&RID=5Y3VDS5A013&FORMAT_OBJECT=TaxBlast&NCBI_GI=off&DESCRIPTIONS=500&ALIGNMENTS=250&FORMAT_BLOCK_ON_RESPAGE=Top&MASK_COLOR=1&MASK_CHAR=2#272561) [[chlamydias](http://www.ncbi.nlm.nih.gov/Taxonomy/Browser/wwwtax.cgi?id=51291)] [SET domain containing protein [Chlamydia trachomatis D/UW-3](http://www.ncbi.nlm.nih.gov/entrez/query.fcgi?cmd=Retrieve&db=Protein&list_uids=15605470&dopt=GenPept)
89. . [Chlamydia trachomatis D-EC](http://www.ncbi.nlm.nih.gov/Taxonomy/Browser/wwwtax.cgi?id=759363) .................................... 101 [2 hits](http://blast.ncbi.nlm.nih.gov/Blast.cgi?CMD=Get&RID=5Y3VDS5A013&FORMAT_OBJECT=TaxBlast&NCBI_GI=off&DESCRIPTIONS=500&ALIGNMENTS=250&FORMAT_BLOCK_ON_RESPAGE=Top&MASK_COLOR=1&MASK_CHAR=2#759363) [[chlamydias](http://www.ncbi.nlm.nih.gov/Taxonomy/Browser/wwwtax.cgi?id=51291)] [SET domain containing protein [Chlamydia trachomatis D/UW-3](http://www.ncbi.nlm.nih.gov/entrez/query.fcgi?cmd=Retrieve&db=Protein&list_uids=385243945&dopt=GenPept)
90. . [Chlamydia trachomatis D-LC](http://www.ncbi.nlm.nih.gov/Taxonomy/Browser/wwwtax.cgi?id=759364) .................................... 101 [2 hits](http://blast.ncbi.nlm.nih.gov/Blast.cgi?CMD=Get&RID=5Y3VDS5A013&FORMAT_OBJECT=TaxBlast&NCBI_GI=off&DESCRIPTIONS=500&ALIGNMENTS=250&FORMAT_BLOCK_ON_RESPAGE=Top&MASK_COLOR=1&MASK_CHAR=2#759364) [[chlamydias](http://www.ncbi.nlm.nih.gov/Taxonomy/Browser/wwwtax.cgi?id=51291)] [SET domain containing protein [Chlamydia trachomatis D/UW-3](http://www.ncbi.nlm.nih.gov/entrez/query.fcgi?cmd=Retrieve&db=Protein&list_uids=385244825&dopt=GenPept)
91. . [Chlamydia trachomatis D/SotonD5](http://www.ncbi.nlm.nih.gov/Taxonomy/Browser/wwwtax.cgi?id=1071757) ............................... 101 [2 hits](http://blast.ncbi.nlm.nih.gov/Blast.cgi?CMD=Get&RID=5Y3VDS5A013&FORMAT_OBJECT=TaxBlast&NCBI_GI=off&DESCRIPTIONS=500&ALIGNMENTS=250&FORMAT_BLOCK_ON_RESPAGE=Top&MASK_COLOR=1&MASK_CHAR=2#1071757) [[chlamydias](http://www.ncbi.nlm.nih.gov/Taxonomy/Browser/wwwtax.cgi?id=51291)] [SET domain containing protein [Chlamydia trachomatis D/UW-3](http://www.ncbi.nlm.nih.gov/entrez/query.fcgi?cmd=Retrieve&db=Protein&list_uids=478455681&dopt=GenPept)
92. . [Chlamydia trachomatis G/SotonG1](http://www.ncbi.nlm.nih.gov/Taxonomy/Browser/wwwtax.cgi?id=1071765) ............................... 101 [2 hits](http://blast.ncbi.nlm.nih.gov/Blast.cgi?CMD=Get&RID=5Y3VDS5A013&FORMAT_OBJECT=TaxBlast&NCBI_GI=off&DESCRIPTIONS=500&ALIGNMENTS=250&FORMAT_BLOCK_ON_RESPAGE=Top&MASK_COLOR=1&MASK_CHAR=2#1071765) [[chlamydias](http://www.ncbi.nlm.nih.gov/Taxonomy/Browser/wwwtax.cgi?id=51291)] [SET domain containing protein [Chlamydia trachomatis D/UW-3](http://www.ncbi.nlm.nih.gov/entrez/query.fcgi?cmd=Retrieve&db=Protein&list_uids=478457490&dopt=GenPept)
93. . [Bordetella bronchiseptica RB50](http://www.ncbi.nlm.nih.gov/Taxonomy/Browser/wwwtax.cgi?id=257310) ................................ 99 [2 hits](http://blast.ncbi.nlm.nih.gov/Blast.cgi?CMD=Get&RID=5Y3VDS5A013&FORMAT_OBJECT=TaxBlast&NCBI_GI=off&DESCRIPTIONS=500&ALIGNMENTS=250&FORMAT_BLOCK_ON_RESPAGE=Top&MASK_COLOR=1&MASK_CHAR=2#257310) [[b-proteobacteria](http://www.ncbi.nlm.nih.gov/Taxonomy/Browser/wwwtax.cgi?id=28216)] [hypothetical protein BB4970 [Bordetella bronchiseptica RB50](http://www.ncbi.nlm.nih.gov/entrez/query.fcgi?cmd=Retrieve&db=Protein&list_uids=33603944&dopt=GenPept)
94. . [Bordetella parapertussis Bpp5](http://www.ncbi.nlm.nih.gov/Taxonomy/Browser/wwwtax.cgi?id=1208660) ................................. 99 [2 hits](http://blast.ncbi.nlm.nih.gov/Blast.cgi?CMD=Get&RID=5Y3VDS5A013&FORMAT_OBJECT=TaxBlast&NCBI_GI=off&DESCRIPTIONS=500&ALIGNMENTS=250&FORMAT_BLOCK_ON_RESPAGE=Top&MASK_COLOR=1&MASK_CHAR=2#1208660) [[b-proteobacteria](http://www.ncbi.nlm.nih.gov/Taxonomy/Browser/wwwtax.cgi?id=28216)] [hypothetical protein BB4970 [Bordetella bronchiseptica RB50](http://www.ncbi.nlm.nih.gov/entrez/query.fcgi?cmd=Retrieve&db=Protein&list_uids=410474946&dopt=GenPept)
95. . [Bordetella bronchiseptica 253](http://www.ncbi.nlm.nih.gov/Taxonomy/Browser/wwwtax.cgi?id=568707) ................................. 99 [2 hits](http://blast.ncbi.nlm.nih.gov/Blast.cgi?CMD=Get&RID=5Y3VDS5A013&FORMAT_OBJECT=TaxBlast&NCBI_GI=off&DESCRIPTIONS=500&ALIGNMENTS=250&FORMAT_BLOCK_ON_RESPAGE=Top&MASK_COLOR=1&MASK_CHAR=2#568707) [[b-proteobacteria](http://www.ncbi.nlm.nih.gov/Taxonomy/Browser/wwwtax.cgi?id=28216)] [hypothetical protein BB4970 [Bordetella bronchiseptica RB50](http://www.ncbi.nlm.nih.gov/entrez/query.fcgi?cmd=Retrieve&db=Protein&list_uids=412340740&dopt=GenPept)
96. . [Bordetella](http://www.ncbi.nlm.nih.gov/Taxonomy/Browser/wwwtax.cgi?id=517) .................................................... 99 [1 hit](http://blast.ncbi.nlm.nih.gov/Blast.cgi?CMD=Get&RID=5Y3VDS5A013&FORMAT_OBJECT=TaxBlast&NCBI_GI=off&DESCRIPTIONS=500&ALIGNMENTS=250&FORMAT_BLOCK_ON_RESPAGE=Top&MASK_COLOR=1&MASK_CHAR=2#517) [[b-proteobacteria](http://www.ncbi.nlm.nih.gov/Taxonomy/Browser/wwwtax.cgi?id=28216)] [hypothetical protein BB4970 [Bordetella bronchiseptica RB50](http://www.ncbi.nlm.nih.gov/entrez/query.fcgi?cmd=Retrieve&db=Protein&list_uids=489912582&dopt=GenPept)
97. . [Bordetella bronchiseptica 1289](http://www.ncbi.nlm.nih.gov/Taxonomy/Browser/wwwtax.cgi?id=1208657) ................................ 99 [1 hit](http://blast.ncbi.nlm.nih.gov/Blast.cgi?CMD=Get&RID=5Y3VDS5A013&FORMAT_OBJECT=TaxBlast&NCBI_GI=off&DESCRIPTIONS=500&ALIGNMENTS=250&FORMAT_BLOCK_ON_RESPAGE=Top&MASK_COLOR=1&MASK_CHAR=2#1208657) [[b-proteobacteria](http://www.ncbi.nlm.nih.gov/Taxonomy/Browser/wwwtax.cgi?id=28216)] [hypothetical protein BB4970 [Bordetella bronchiseptica RB50](http://www.ncbi.nlm.nih.gov/entrez/query.fcgi?cmd=Retrieve&db=Protein&list_uids=410567953&dopt=GenPept)
98. . [Chlorobium phaeobacteroides BS1](http://www.ncbi.nlm.nih.gov/Taxonomy/Browser/wwwtax.cgi?id=331678) ............................... 99 [2 hits](http://blast.ncbi.nlm.nih.gov/Blast.cgi?CMD=Get&RID=5Y3VDS5A013&FORMAT_OBJECT=TaxBlast&NCBI_GI=off&DESCRIPTIONS=500&ALIGNMENTS=250&FORMAT_BLOCK_ON_RESPAGE=Top&MASK_COLOR=1&MASK_CHAR=2#331678) [[green sulfur bacteria](http://www.ncbi.nlm.nih.gov/Taxonomy/Browser/wwwtax.cgi?id=1090)] [nuclear protein SET [Chlorobium phaeobacteroides BS1] >gi|5](http://www.ncbi.nlm.nih.gov/entrez/query.fcgi?cmd=Retrieve&db=Protein&list_uids=189500419&dopt=GenPept)
99. . [Chlorobium phaeobacteroides](http://www.ncbi.nlm.nih.gov/Taxonomy/Browser/wwwtax.cgi?id=1096) ................................... 99 [1 hit](http://blast.ncbi.nlm.nih.gov/Blast.cgi?CMD=Get&RID=5Y3VDS5A013&FORMAT_OBJECT=TaxBlast&NCBI_GI=off&DESCRIPTIONS=500&ALIGNMENTS=250&FORMAT_BLOCK_ON_RESPAGE=Top&MASK_COLOR=1&MASK_CHAR=2#1096) [[green sulfur bacteria](http://www.ncbi.nlm.nih.gov/Taxonomy/Browser/wwwtax.cgi?id=1090)] [nuclear protein SET [Chlorobium phaeobacteroides BS1] >gi|5](http://www.ncbi.nlm.nih.gov/entrez/query.fcgi?cmd=Retrieve&db=Protein&list_uids=501451440&dopt=GenPept)
100. . [Pseudoxanthomonas suwonensis 11-1](http://www.ncbi.nlm.nih.gov/Taxonomy/Browser/wwwtax.cgi?id=743721) ............................. 99 [2 hits](http://blast.ncbi.nlm.nih.gov/Blast.cgi?CMD=Get&RID=5Y3VDS5A013&FORMAT_OBJECT=TaxBlast&NCBI_GI=off&DESCRIPTIONS=500&ALIGNMENTS=250&FORMAT_BLOCK_ON_RESPAGE=Top&MASK_COLOR=1&MASK_CHAR=2#743721) [[g-proteobacteria](http://www.ncbi.nlm.nih.gov/Taxonomy/Browser/wwwtax.cgi?id=1236)] [nuclear protein SET [Pseudoxanthomonas suwonensis 11-1] >gi](http://www.ncbi.nlm.nih.gov/entrez/query.fcgi?cmd=Retrieve&db=Protein&list_uids=319786707&dopt=GenPept)
101. . [Pseudoxanthomonas suwonensis](http://www.ncbi.nlm.nih.gov/Taxonomy/Browser/wwwtax.cgi?id=314722) .................................. 99 [1 hit](http://blast.ncbi.nlm.nih.gov/Blast.cgi?CMD=Get&RID=5Y3VDS5A013&FORMAT_OBJECT=TaxBlast&NCBI_GI=off&DESCRIPTIONS=500&ALIGNMENTS=250&FORMAT_BLOCK_ON_RESPAGE=Top&MASK_COLOR=1&MASK_CHAR=2#314722) [[g-proteobacteria](http://www.ncbi.nlm.nih.gov/Taxonomy/Browser/wwwtax.cgi?id=1236)] [nuclear protein SET [Pseudoxanthomonas suwonensis 11-1] >gi](http://www.ncbi.nlm.nih.gov/entrez/query.fcgi?cmd=Retrieve&db=Protein&list_uids=503300120&dopt=GenPept)
102. . [Achromobacter piechaudii](http://www.ncbi.nlm.nih.gov/Taxonomy/Browser/wwwtax.cgi?id=72556) ...................................... 100 [2 hits](http://blast.ncbi.nlm.nih.gov/Blast.cgi?CMD=Get&RID=5Y3VDS5A013&FORMAT_OBJECT=TaxBlast&NCBI_GI=off&DESCRIPTIONS=500&ALIGNMENTS=250&FORMAT_BLOCK_ON_RESPAGE=Top&MASK_COLOR=1&MASK_CHAR=2#72556) [[b-proteobacteria](http://www.ncbi.nlm.nih.gov/Taxonomy/Browser/wwwtax.cgi?id=28216)] [lysine methyltransferase [Achromobacter piechaudii] >gi|292](http://www.ncbi.nlm.nih.gov/entrez/query.fcgi?cmd=Retrieve&db=Protein&list_uids=493248122&dopt=GenPept)
103. . [Achromobacter piechaudii ATCC 43553](http://www.ncbi.nlm.nih.gov/Taxonomy/Browser/wwwtax.cgi?id=742159) ........................... 100 [1 hit](http://blast.ncbi.nlm.nih.gov/Blast.cgi?CMD=Get&RID=5Y3VDS5A013&FORMAT_OBJECT=TaxBlast&NCBI_GI=off&DESCRIPTIONS=500&ALIGNMENTS=250&FORMAT_BLOCK_ON_RESPAGE=Top&MASK_COLOR=1&MASK_CHAR=2#742159) [[b-proteobacteria](http://www.ncbi.nlm.nih.gov/Taxonomy/Browser/wwwtax.cgi?id=28216)] [lysine methyltransferase [Achromobacter piechaudii] >gi|292](http://www.ncbi.nlm.nih.gov/entrez/query.fcgi?cmd=Retrieve&db=Protein&list_uids=292818940&dopt=GenPept)
104. . [Rhodanobacter thiooxydans](http://www.ncbi.nlm.nih.gov/Taxonomy/Browser/wwwtax.cgi?id=416169) ..................................... 98 [1 hit](http://blast.ncbi.nlm.nih.gov/Blast.cgi?CMD=Get&RID=5Y3VDS5A013&FORMAT_OBJECT=TaxBlast&NCBI_GI=off&DESCRIPTIONS=500&ALIGNMENTS=250&FORMAT_BLOCK_ON_RESPAGE=Top&MASK_COLOR=1&MASK_CHAR=2#416169) [[g-proteobacteria](http://www.ncbi.nlm.nih.gov/Taxonomy/Browser/wwwtax.cgi?id=1236)] [SET domain-containing protein [Rhodanobacter thiooxydans] >](http://www.ncbi.nlm.nih.gov/entrez/query.fcgi?cmd=Retrieve&db=Protein&list_uids=495709281&dopt=GenPept)
105. . [Rhodanobacter thiooxydans LCS2](http://www.ncbi.nlm.nih.gov/Taxonomy/Browser/wwwtax.cgi?id=1163409) ................................ 98 [1 hit](http://blast.ncbi.nlm.nih.gov/Blast.cgi?CMD=Get&RID=5Y3VDS5A013&FORMAT_OBJECT=TaxBlast&NCBI_GI=off&DESCRIPTIONS=500&ALIGNMENTS=250&FORMAT_BLOCK_ON_RESPAGE=Top&MASK_COLOR=1&MASK_CHAR=2#1163409) [[g-proteobacteria](http://www.ncbi.nlm.nih.gov/Taxonomy/Browser/wwwtax.cgi?id=1236)] [SET domain-containing protein [Rhodanobacter thiooxydans] >](http://www.ncbi.nlm.nih.gov/entrez/query.fcgi?cmd=Retrieve&db=Protein&list_uids=388446376&dopt=GenPept)
106. . [Rhodanobacter sp. 2APBS1](http://www.ncbi.nlm.nih.gov/Taxonomy/Browser/wwwtax.cgi?id=666685) ...................................... 98 [2 hits](http://blast.ncbi.nlm.nih.gov/Blast.cgi?CMD=Get&RID=5Y3VDS5A013&FORMAT_OBJECT=TaxBlast&NCBI_GI=off&DESCRIPTIONS=500&ALIGNMENTS=250&FORMAT_BLOCK_ON_RESPAGE=Top&MASK_COLOR=1&MASK_CHAR=2#666685) [[g-proteobacteria](http://www.ncbi.nlm.nih.gov/Taxonomy/Browser/wwwtax.cgi?id=1236)] [SET domain-containing protein [Rhodanobacter sp. 2APBS1] >g](http://www.ncbi.nlm.nih.gov/entrez/query.fcgi?cmd=Retrieve&db=Protein&list_uids=469819443&dopt=GenPept)
107. . [Rhodanobacter](http://www.ncbi.nlm.nih.gov/Taxonomy/Browser/wwwtax.cgi?id=75309) ................................................. 98 [1 hit](http://blast.ncbi.nlm.nih.gov/Blast.cgi?CMD=Get&RID=5Y3VDS5A013&FORMAT_OBJECT=TaxBlast&NCBI_GI=off&DESCRIPTIONS=500&ALIGNMENTS=250&FORMAT_BLOCK_ON_RESPAGE=Top&MASK_COLOR=1&MASK_CHAR=2#75309) [[g-proteobacteria](http://www.ncbi.nlm.nih.gov/Taxonomy/Browser/wwwtax.cgi?id=1236)] [SET domain-containing protein [Rhodanobacter sp. 2APBS1] >g](http://www.ncbi.nlm.nih.gov/entrez/query.fcgi?cmd=Retrieve&db=Protein&list_uids=494778059&dopt=GenPept)
108. . [Rhodanobacter sp. 116-2](http://www.ncbi.nlm.nih.gov/Taxonomy/Browser/wwwtax.cgi?id=1076650) ....................................... 98 [1 hit](http://blast.ncbi.nlm.nih.gov/Blast.cgi?CMD=Get&RID=5Y3VDS5A013&FORMAT_OBJECT=TaxBlast&NCBI_GI=off&DESCRIPTIONS=500&ALIGNMENTS=250&FORMAT_BLOCK_ON_RESPAGE=Top&MASK_COLOR=1&MASK_CHAR=2#1076650) [[g-proteobacteria](http://www.ncbi.nlm.nih.gov/Taxonomy/Browser/wwwtax.cgi?id=1236)] [SET domain-containing protein [Rhodanobacter sp. 2APBS1] >g](http://www.ncbi.nlm.nih.gov/entrez/query.fcgi?cmd=Retrieve&db=Protein&list_uids=388443877&dopt=GenPept)
109. . [Pusillimonas noertemannii](http://www.ncbi.nlm.nih.gov/Taxonomy/Browser/wwwtax.cgi?id=305977) ..................................... 99 [1 hit](http://blast.ncbi.nlm.nih.gov/Blast.cgi?CMD=Get&RID=5Y3VDS5A013&FORMAT_OBJECT=TaxBlast&NCBI_GI=off&DESCRIPTIONS=500&ALIGNMENTS=250&FORMAT_BLOCK_ON_RESPAGE=Top&MASK_COLOR=1&MASK_CHAR=2#305977) [[b-proteobacteria](http://www.ncbi.nlm.nih.gov/Taxonomy/Browser/wwwtax.cgi?id=28216)] [hypothetical protein [Pusillimonas noertemannii]](http://www.ncbi.nlm.nih.gov/entrez/query.fcgi?cmd=Retrieve&db=Protein&list_uids=516093739&dopt=GenPept)
110. . [Frateuria aurantia DSM 6220](http://www.ncbi.nlm.nih.gov/Taxonomy/Browser/wwwtax.cgi?id=767434) ................................... 98 [2 hits](http://blast.ncbi.nlm.nih.gov/Blast.cgi?CMD=Get&RID=5Y3VDS5A013&FORMAT_OBJECT=TaxBlast&NCBI_GI=off&DESCRIPTIONS=500&ALIGNMENTS=250&FORMAT_BLOCK_ON_RESPAGE=Top&MASK_COLOR=1&MASK_CHAR=2#767434) [[g-proteobacteria](http://www.ncbi.nlm.nih.gov/Taxonomy/Browser/wwwtax.cgi?id=1236)] [SET domain-containing protein [Frateuria aurantia DSM 6220]](http://www.ncbi.nlm.nih.gov/entrez/query.fcgi?cmd=Retrieve&db=Protein&list_uids=383316103&dopt=GenPept)
111. . [Frateuria aurantia](http://www.ncbi.nlm.nih.gov/Taxonomy/Browser/wwwtax.cgi?id=81475) ............................................ 98 [1 hit](http://blast.ncbi.nlm.nih.gov/Blast.cgi?CMD=Get&RID=5Y3VDS5A013&FORMAT_OBJECT=TaxBlast&NCBI_GI=off&DESCRIPTIONS=500&ALIGNMENTS=250&FORMAT_BLOCK_ON_RESPAGE=Top&MASK_COLOR=1&MASK_CHAR=2#81475) [[g-proteobacteria](http://www.ncbi.nlm.nih.gov/Taxonomy/Browser/wwwtax.cgi?id=1236)] [SET domain-containing protein [Frateuria aurantia DSM 6220]](http://www.ncbi.nlm.nih.gov/entrez/query.fcgi?cmd=Retrieve&db=Protein&list_uids=504215167&dopt=GenPept)
112. . [Chlamydophila felis Fe/C-56](http://www.ncbi.nlm.nih.gov/Taxonomy/Browser/wwwtax.cgi?id=264202) ................................... 99 [2 hits](http://blast.ncbi.nlm.nih.gov/Blast.cgi?CMD=Get&RID=5Y3VDS5A013&FORMAT_OBJECT=TaxBlast&NCBI_GI=off&DESCRIPTIONS=500&ALIGNMENTS=250&FORMAT_BLOCK_ON_RESPAGE=Top&MASK_COLOR=1&MASK_CHAR=2#264202) [[chlamydias](http://www.ncbi.nlm.nih.gov/Taxonomy/Browser/wwwtax.cgi?id=51291)] [SET domain-containing protein [Chlamydophila felis Fe/C-56]](http://www.ncbi.nlm.nih.gov/entrez/query.fcgi?cmd=Retrieve&db=Protein&list_uids=89897932&dopt=GenPept)
113. . [Chlamydophila felis](http://www.ncbi.nlm.nih.gov/Taxonomy/Browser/wwwtax.cgi?id=83556) ........................................... 99 [1 hit](http://blast.ncbi.nlm.nih.gov/Blast.cgi?CMD=Get&RID=5Y3VDS5A013&FORMAT_OBJECT=TaxBlast&NCBI_GI=off&DESCRIPTIONS=500&ALIGNMENTS=250&FORMAT_BLOCK_ON_RESPAGE=Top&MASK_COLOR=1&MASK_CHAR=2#83556) [[chlamydias](http://www.ncbi.nlm.nih.gov/Taxonomy/Browser/wwwtax.cgi?id=51291)] [SET domain-containing protein [Chlamydophila felis Fe/C-56]](http://www.ncbi.nlm.nih.gov/entrez/query.fcgi?cmd=Retrieve&db=Protein&list_uids=499776948&dopt=GenPept)
114. . [Bordetella holmesii](http://www.ncbi.nlm.nih.gov/Taxonomy/Browser/wwwtax.cgi?id=35814) ........................................... 98 [2 hits](http://blast.ncbi.nlm.nih.gov/Blast.cgi?CMD=Get&RID=5Y3VDS5A013&FORMAT_OBJECT=TaxBlast&NCBI_GI=off&DESCRIPTIONS=500&ALIGNMENTS=250&FORMAT_BLOCK_ON_RESPAGE=Top&MASK_COLOR=1&MASK_CHAR=2#35814) [[b-proteobacteria](http://www.ncbi.nlm.nih.gov/Taxonomy/Browser/wwwtax.cgi?id=28216)] [hypothetical protein [Bordetella holmesii] >gi|451921814|gb](http://www.ncbi.nlm.nih.gov/entrez/query.fcgi?cmd=Retrieve&db=Protein&list_uids=491158758&dopt=GenPept)
115. . [Bordetella holmesii F627](http://www.ncbi.nlm.nih.gov/Taxonomy/Browser/wwwtax.cgi?id=1266729) ...................................... 98 [1 hit](http://blast.ncbi.nlm.nih.gov/Blast.cgi?CMD=Get&RID=5Y3VDS5A013&FORMAT_OBJECT=TaxBlast&NCBI_GI=off&DESCRIPTIONS=500&ALIGNMENTS=250&FORMAT_BLOCK_ON_RESPAGE=Top&MASK_COLOR=1&MASK_CHAR=2#1266729) [[b-proteobacteria](http://www.ncbi.nlm.nih.gov/Taxonomy/Browser/wwwtax.cgi?id=28216)] [hypothetical protein [Bordetella holmesii] >gi|451921814|gb](http://www.ncbi.nlm.nih.gov/entrez/query.fcgi?cmd=Retrieve&db=Protein&list_uids=451921814&dopt=GenPept)
116. . [Bordetella holmesii H558](http://www.ncbi.nlm.nih.gov/Taxonomy/Browser/wwwtax.cgi?id=1281885) ...................................... 98 [1 hit](http://blast.ncbi.nlm.nih.gov/Blast.cgi?CMD=Get&RID=5Y3VDS5A013&FORMAT_OBJECT=TaxBlast&NCBI_GI=off&DESCRIPTIONS=500&ALIGNMENTS=250&FORMAT_BLOCK_ON_RESPAGE=Top&MASK_COLOR=1&MASK_CHAR=2#1281885) [[b-proteobacteria](http://www.ncbi.nlm.nih.gov/Taxonomy/Browser/wwwtax.cgi?id=28216)] [hypothetical protein [Bordetella holmesii] >gi|451921814|gb](http://www.ncbi.nlm.nih.gov/entrez/query.fcgi?cmd=Retrieve&db=Protein&list_uids=451922955&dopt=GenPept)
117. . [Dyella japonica](http://www.ncbi.nlm.nih.gov/Taxonomy/Browser/wwwtax.cgi?id=231455) ............................................... 98 [1 hit](http://blast.ncbi.nlm.nih.gov/Blast.cgi?CMD=Get&RID=5Y3VDS5A013&FORMAT_OBJECT=TaxBlast&NCBI_GI=off&DESCRIPTIONS=500&ALIGNMENTS=250&FORMAT_BLOCK_ON_RESPAGE=Top&MASK_COLOR=1&MASK_CHAR=2#231455) [[g-proteobacteria](http://www.ncbi.nlm.nih.gov/Taxonomy/Browser/wwwtax.cgi?id=1236)] [nuclear protein SET [Dyella japonica]](http://www.ncbi.nlm.nih.gov/entrez/query.fcgi?cmd=Retrieve&db=Protein&list_uids=518296156&dopt=GenPept)
118. . [Rhodanobacter spathiphylli](http://www.ncbi.nlm.nih.gov/Taxonomy/Browser/wwwtax.cgi?id=347483) .................................... 97 [1 hit](http://blast.ncbi.nlm.nih.gov/Blast.cgi?CMD=Get&RID=5Y3VDS5A013&FORMAT_OBJECT=TaxBlast&NCBI_GI=off&DESCRIPTIONS=500&ALIGNMENTS=250&FORMAT_BLOCK_ON_RESPAGE=Top&MASK_COLOR=1&MASK_CHAR=2#347483) [[g-proteobacteria](http://www.ncbi.nlm.nih.gov/Taxonomy/Browser/wwwtax.cgi?id=1236)] [SET domain-containing protein [Rhodanobacter spathiphylli]](http://www.ncbi.nlm.nih.gov/entrez/query.fcgi?cmd=Retrieve&db=Protein&list_uids=495079318&dopt=GenPept)
119. . [Rhodanobacter spathiphylli B39](http://www.ncbi.nlm.nih.gov/Taxonomy/Browser/wwwtax.cgi?id=1163407) ................................ 97 [1 hit](http://blast.ncbi.nlm.nih.gov/Blast.cgi?CMD=Get&RID=5Y3VDS5A013&FORMAT_OBJECT=TaxBlast&NCBI_GI=off&DESCRIPTIONS=500&ALIGNMENTS=250&FORMAT_BLOCK_ON_RESPAGE=Top&MASK_COLOR=1&MASK_CHAR=2#1163407) [[g-proteobacteria](http://www.ncbi.nlm.nih.gov/Taxonomy/Browser/wwwtax.cgi?id=1236)] [SET domain-containing protein [Rhodanobacter spathiphylli]](http://www.ncbi.nlm.nih.gov/entrez/query.fcgi?cmd=Retrieve&db=Protein&list_uids=388438708&dopt=GenPept)
120. . [Achromobacter xylosoxidans A8](http://www.ncbi.nlm.nih.gov/Taxonomy/Browser/wwwtax.cgi?id=762376) ................................. 98 [2 hits](http://blast.ncbi.nlm.nih.gov/Blast.cgi?CMD=Get&RID=5Y3VDS5A013&FORMAT_OBJECT=TaxBlast&NCBI_GI=off&DESCRIPTIONS=500&ALIGNMENTS=250&FORMAT_BLOCK_ON_RESPAGE=Top&MASK_COLOR=1&MASK_CHAR=2#762376) [[b-proteobacteria](http://www.ncbi.nlm.nih.gov/Taxonomy/Browser/wwwtax.cgi?id=28216)] [SET domain-containing protein [Achromobacter xylosoxidans A](http://www.ncbi.nlm.nih.gov/entrez/query.fcgi?cmd=Retrieve&db=Protein&list_uids=311103281&dopt=GenPept)
121. . [Achromobacter xylosoxidans](http://www.ncbi.nlm.nih.gov/Taxonomy/Browser/wwwtax.cgi?id=85698) .................................... 98 [4 hits](http://blast.ncbi.nlm.nih.gov/Blast.cgi?CMD=Get&RID=5Y3VDS5A013&FORMAT_OBJECT=TaxBlast&NCBI_GI=off&DESCRIPTIONS=500&ALIGNMENTS=250&FORMAT_BLOCK_ON_RESPAGE=Top&MASK_COLOR=1&MASK_CHAR=2#85698) [[b-proteobacteria](http://www.ncbi.nlm.nih.gov/Taxonomy/Browser/wwwtax.cgi?id=28216)] [SET domain-containing protein [Achromobacter xylosoxidans A](http://www.ncbi.nlm.nih.gov/entrez/query.fcgi?cmd=Retrieve&db=Protein&list_uids=503156160&dopt=GenPept)
122. . [Delftia sp. Cs1-4](http://www.ncbi.nlm.nih.gov/Taxonomy/Browser/wwwtax.cgi?id=742013) ............................................. 98 [3 hits](http://blast.ncbi.nlm.nih.gov/Blast.cgi?CMD=Get&RID=5Y3VDS5A013&FORMAT_OBJECT=TaxBlast&NCBI_GI=off&DESCRIPTIONS=500&ALIGNMENTS=250&FORMAT_BLOCK_ON_RESPAGE=Top&MASK_COLOR=1&MASK_CHAR=2#742013) [[b-proteobacteria](http://www.ncbi.nlm.nih.gov/Taxonomy/Browser/wwwtax.cgi?id=28216)] [nuclear protein SET [Delftia sp. Cs1-4] >gi|503570495|ref|W](http://www.ncbi.nlm.nih.gov/entrez/query.fcgi?cmd=Retrieve&db=Protein&list_uids=333917236&dopt=GenPept)
123. . [Chlorobium chlorochromatii CaD3](http://www.ncbi.nlm.nih.gov/Taxonomy/Browser/wwwtax.cgi?id=340177) ............................... 97 [2 hits](http://blast.ncbi.nlm.nih.gov/Blast.cgi?CMD=Get&RID=5Y3VDS5A013&FORMAT_OBJECT=TaxBlast&NCBI_GI=off&DESCRIPTIONS=500&ALIGNMENTS=250&FORMAT_BLOCK_ON_RESPAGE=Top&MASK_COLOR=1&MASK_CHAR=2#340177) [[green sulfur bacteria](http://www.ncbi.nlm.nih.gov/Taxonomy/Browser/wwwtax.cgi?id=1090)] [nuclear protein SET [Chlorobium chlorochromatii CaD3] >gi|4](http://www.ncbi.nlm.nih.gov/entrez/query.fcgi?cmd=Retrieve&db=Protein&list_uids=78189457&dopt=GenPept)
124. . [Chlorobium chlorochromatii](http://www.ncbi.nlm.nih.gov/Taxonomy/Browser/wwwtax.cgi?id=337090) (epibiont of the photot...) ........ 97 [1 hit](http://blast.ncbi.nlm.nih.gov/Blast.cgi?CMD=Get&RID=5Y3VDS5A013&FORMAT_OBJECT=TaxBlast&NCBI_GI=off&DESCRIPTIONS=500&ALIGNMENTS=250&FORMAT_BLOCK_ON_RESPAGE=Top&MASK_COLOR=1&MASK_CHAR=2#337090) [[green sulfur bacteria](http://www.ncbi.nlm.nih.gov/Taxonomy/Browser/wwwtax.cgi?id=1090)] [nuclear protein SET [Chlorobium chlorochromatii CaD3] >gi|4](http://www.ncbi.nlm.nih.gov/entrez/query.fcgi?cmd=Retrieve&db=Protein&list_uids=499681782&dopt=GenPept)
125. . [Bradyrhizobium sp. ORS 285](http://www.ncbi.nlm.nih.gov/Taxonomy/Browser/wwwtax.cgi?id=115808) .................................... 99 [2 hits](http://blast.ncbi.nlm.nih.gov/Blast.cgi?CMD=Get&RID=5Y3VDS5A013&FORMAT_OBJECT=TaxBlast&NCBI_GI=off&DESCRIPTIONS=500&ALIGNMENTS=250&FORMAT_BLOCK_ON_RESPAGE=Top&MASK_COLOR=1&MASK_CHAR=2#115808) [[a-proteobacteria](http://www.ncbi.nlm.nih.gov/Taxonomy/Browser/wwwtax.cgi?id=28211)] [histone-lysine N-methyltransferase with a SET domain [Brady](http://www.ncbi.nlm.nih.gov/entrez/query.fcgi?cmd=Retrieve&db=Protein&list_uids=493658771&dopt=GenPept)
126. . [Alicycliphilus denitrificans BC](http://www.ncbi.nlm.nih.gov/Taxonomy/Browser/wwwtax.cgi?id=596153) ............................... 98 [2 hits](http://blast.ncbi.nlm.nih.gov/Blast.cgi?CMD=Get&RID=5Y3VDS5A013&FORMAT_OBJECT=TaxBlast&NCBI_GI=off&DESCRIPTIONS=500&ALIGNMENTS=250&FORMAT_BLOCK_ON_RESPAGE=Top&MASK_COLOR=1&MASK_CHAR=2#596153) [[b-proteobacteria](http://www.ncbi.nlm.nih.gov/Taxonomy/Browser/wwwtax.cgi?id=28216)] [nuclear protein set [Alicycliphilus denitrificans BC] >gi|3](http://www.ncbi.nlm.nih.gov/entrez/query.fcgi?cmd=Retrieve&db=Protein&list_uids=319764851&dopt=GenPept)
127. . [Alicycliphilus denitrificans K601](http://www.ncbi.nlm.nih.gov/Taxonomy/Browser/wwwtax.cgi?id=596154) ............................. 98 [2 hits](http://blast.ncbi.nlm.nih.gov/Blast.cgi?CMD=Get&RID=5Y3VDS5A013&FORMAT_OBJECT=TaxBlast&NCBI_GI=off&DESCRIPTIONS=500&ALIGNMENTS=250&FORMAT_BLOCK_ON_RESPAGE=Top&MASK_COLOR=1&MASK_CHAR=2#596154) [[b-proteobacteria](http://www.ncbi.nlm.nih.gov/Taxonomy/Browser/wwwtax.cgi?id=28216)] [nuclear protein set [Alicycliphilus denitrificans BC] >gi|3](http://www.ncbi.nlm.nih.gov/entrez/query.fcgi?cmd=Retrieve&db=Protein&list_uids=330827056&dopt=GenPept)
128. . [Alicycliphilus denitrificans](http://www.ncbi.nlm.nih.gov/Taxonomy/Browser/wwwtax.cgi?id=179636) .................................. 98 [1 hit](http://blast.ncbi.nlm.nih.gov/Blast.cgi?CMD=Get&RID=5Y3VDS5A013&FORMAT_OBJECT=TaxBlast&NCBI_GI=off&DESCRIPTIONS=500&ALIGNMENTS=250&FORMAT_BLOCK_ON_RESPAGE=Top&MASK_COLOR=1&MASK_CHAR=2#179636) [[b-proteobacteria](http://www.ncbi.nlm.nih.gov/Taxonomy/Browser/wwwtax.cgi?id=28216)] [nuclear protein set [Alicycliphilus denitrificans BC] >gi|3](http://www.ncbi.nlm.nih.gov/entrez/query.fcgi?cmd=Retrieve&db=Protein&list_uids=503286281&dopt=GenPept)
129. . [Chlorobium tepidum TLS](http://www.ncbi.nlm.nih.gov/Taxonomy/Browser/wwwtax.cgi?id=194439) ........................................ 97 [2 hits](http://blast.ncbi.nlm.nih.gov/Blast.cgi?CMD=Get&RID=5Y3VDS5A013&FORMAT_OBJECT=TaxBlast&NCBI_GI=off&DESCRIPTIONS=500&ALIGNMENTS=250&FORMAT_BLOCK_ON_RESPAGE=Top&MASK_COLOR=1&MASK_CHAR=2#194439) [[green sulfur bacteria](http://www.ncbi.nlm.nih.gov/Taxonomy/Browser/wwwtax.cgi?id=1090)] [hypothetical protein CT0952 [Chlorobium tepidum TLS] >gi|49](http://www.ncbi.nlm.nih.gov/entrez/query.fcgi?cmd=Retrieve&db=Protein&list_uids=21673780&dopt=GenPept)
130. . [Chlorobaculum tepidum](http://www.ncbi.nlm.nih.gov/Taxonomy/Browser/wwwtax.cgi?id=1097) ......................................... 97 [1 hit](http://blast.ncbi.nlm.nih.gov/Blast.cgi?CMD=Get&RID=5Y3VDS5A013&FORMAT_OBJECT=TaxBlast&NCBI_GI=off&DESCRIPTIONS=500&ALIGNMENTS=250&FORMAT_BLOCK_ON_RESPAGE=Top&MASK_COLOR=1&MASK_CHAR=2#1097) [[green sulfur bacteria](http://www.ncbi.nlm.nih.gov/Taxonomy/Browser/wwwtax.cgi?id=1090)] [hypothetical protein CT0952 [Chlorobium tepidum TLS] >gi|49](http://www.ncbi.nlm.nih.gov/entrez/query.fcgi?cmd=Retrieve&db=Protein&list_uids=499235092&dopt=GenPept)
131. . [Delftia acidovorans](http://www.ncbi.nlm.nih.gov/Taxonomy/Browser/wwwtax.cgi?id=80866) ........................................... 98 [2 hits](http://blast.ncbi.nlm.nih.gov/Blast.cgi?CMD=Get&RID=5Y3VDS5A013&FORMAT_OBJECT=TaxBlast&NCBI_GI=off&DESCRIPTIONS=500&ALIGNMENTS=250&FORMAT_BLOCK_ON_RESPAGE=Top&MASK_COLOR=1&MASK_CHAR=2#80866) [[b-proteobacteria](http://www.ncbi.nlm.nih.gov/Taxonomy/Browser/wwwtax.cgi?id=28216)] [hypothetical protein [Delftia acidovorans] >gi|512036446|gb](http://www.ncbi.nlm.nih.gov/entrez/query.fcgi?cmd=Retrieve&db=Protein&list_uids=512565765&dopt=GenPept)
132. . [Delftia acidovorans CCUG 274B](http://www.ncbi.nlm.nih.gov/Taxonomy/Browser/wwwtax.cgi?id=883101) ................................. 98 [1 hit](http://blast.ncbi.nlm.nih.gov/Blast.cgi?CMD=Get&RID=5Y3VDS5A013&FORMAT_OBJECT=TaxBlast&NCBI_GI=off&DESCRIPTIONS=500&ALIGNMENTS=250&FORMAT_BLOCK_ON_RESPAGE=Top&MASK_COLOR=1&MASK_CHAR=2#883101) [[b-proteobacteria](http://www.ncbi.nlm.nih.gov/Taxonomy/Browser/wwwtax.cgi?id=28216)] [hypothetical protein [Delftia acidovorans] >gi|512036446|gb](http://www.ncbi.nlm.nih.gov/entrez/query.fcgi?cmd=Retrieve&db=Protein&list_uids=512036446&dopt=GenPept)
133. . [Delftia acidovorans CCUG 15835](http://www.ncbi.nlm.nih.gov/Taxonomy/Browser/wwwtax.cgi?id=883100) ................................ 98 [1 hit](http://blast.ncbi.nlm.nih.gov/Blast.cgi?CMD=Get&RID=5Y3VDS5A013&FORMAT_OBJECT=TaxBlast&NCBI_GI=off&DESCRIPTIONS=500&ALIGNMENTS=250&FORMAT_BLOCK_ON_RESPAGE=Top&MASK_COLOR=1&MASK_CHAR=2#883100) [[b-proteobacteria](http://www.ncbi.nlm.nih.gov/Taxonomy/Browser/wwwtax.cgi?id=28216)] [hypothetical protein [Delftia acidovorans] >gi|512036446|gb](http://www.ncbi.nlm.nih.gov/entrez/query.fcgi?cmd=Retrieve&db=Protein&list_uids=512036621&dopt=GenPept)
134. . [Chryseobacterium taeanense](http://www.ncbi.nlm.nih.gov/Taxonomy/Browser/wwwtax.cgi?id=311334) .................................... 98 [1 hit](http://blast.ncbi.nlm.nih.gov/Blast.cgi?CMD=Get&RID=5Y3VDS5A013&FORMAT_OBJECT=TaxBlast&NCBI_GI=off&DESCRIPTIONS=500&ALIGNMENTS=250&FORMAT_BLOCK_ON_RESPAGE=Top&MASK_COLOR=1&MASK_CHAR=2#311334) [[CFB group bacteria](http://www.ncbi.nlm.nih.gov/Taxonomy/Browser/wwwtax.cgi?id=976)] [lysine methyltransferase [Chryseobacterium taeanense]](http://www.ncbi.nlm.nih.gov/entrez/query.fcgi?cmd=Retrieve&db=Protein&list_uids=515976467&dopt=GenPept)
135. . [Chlamydia muridarum](http://www.ncbi.nlm.nih.gov/Taxonomy/Browser/wwwtax.cgi?id=83560) (agent of mouse pneumon...) ............... 98 [2 hits](http://blast.ncbi.nlm.nih.gov/Blast.cgi?CMD=Get&RID=5Y3VDS5A013&FORMAT_OBJECT=TaxBlast&NCBI_GI=off&DESCRIPTIONS=500&ALIGNMENTS=250&FORMAT_BLOCK_ON_RESPAGE=Top&MASK_COLOR=1&MASK_CHAR=2#83560) [[chlamydias](http://www.ncbi.nlm.nih.gov/Taxonomy/Browser/wwwtax.cgi?id=51291)] [lysine methyltransferase [Chlamydia muridarum]](http://www.ncbi.nlm.nih.gov/entrez/query.fcgi?cmd=Retrieve&db=Protein&list_uids=497918555&dopt=GenPept)
136. . [Burkholderia phymatum STM815](http://www.ncbi.nlm.nih.gov/Taxonomy/Browser/wwwtax.cgi?id=391038) .................................. 97 [4 hits](http://blast.ncbi.nlm.nih.gov/Blast.cgi?CMD=Get&RID=5Y3VDS5A013&FORMAT_OBJECT=TaxBlast&NCBI_GI=off&DESCRIPTIONS=500&ALIGNMENTS=250&FORMAT_BLOCK_ON_RESPAGE=Top&MASK_COLOR=1&MASK_CHAR=2#391038) [[b-proteobacteria](http://www.ncbi.nlm.nih.gov/Taxonomy/Browser/wwwtax.cgi?id=28216)] [nuclear protein SET [Burkholderia phymatum STM815] >gi|5013](http://www.ncbi.nlm.nih.gov/entrez/query.fcgi?cmd=Retrieve&db=Protein&list_uids=186474601&dopt=GenPept)
137. . [Burkholderia phymatum](http://www.ncbi.nlm.nih.gov/Taxonomy/Browser/wwwtax.cgi?id=148447) ......................................... 97 [2 hits](http://blast.ncbi.nlm.nih.gov/Blast.cgi?CMD=Get&RID=5Y3VDS5A013&FORMAT_OBJECT=TaxBlast&NCBI_GI=off&DESCRIPTIONS=500&ALIGNMENTS=250&FORMAT_BLOCK_ON_RESPAGE=Top&MASK_COLOR=1&MASK_CHAR=2#148447) [[b-proteobacteria](http://www.ncbi.nlm.nih.gov/Taxonomy/Browser/wwwtax.cgi?id=28216)] [nuclear protein SET [Burkholderia phymatum STM815] >gi|5013](http://www.ncbi.nlm.nih.gov/entrez/query.fcgi?cmd=Retrieve&db=Protein&list_uids=501375107&dopt=GenPept)
138. . [Bradyrhizobium japonicum](http://www.ncbi.nlm.nih.gov/Taxonomy/Browser/wwwtax.cgi?id=375) ...................................... 98 [3 hits](http://blast.ncbi.nlm.nih.gov/Blast.cgi?CMD=Get&RID=5Y3VDS5A013&FORMAT_OBJECT=TaxBlast&NCBI_GI=off&DESCRIPTIONS=500&ALIGNMENTS=250&FORMAT_BLOCK_ON_RESPAGE=Top&MASK_COLOR=1&MASK_CHAR=2#375) [[a-proteobacteria](http://www.ncbi.nlm.nih.gov/Taxonomy/Browser/wwwtax.cgi?id=28211)] [histone-lysine N-methyltransferase with a SET domain [Brady](http://www.ncbi.nlm.nih.gov/entrez/query.fcgi?cmd=Retrieve&db=Protein&list_uids=521997515&dopt=GenPept)
139. . [Chlamydia muridarum Nigg](http://www.ncbi.nlm.nih.gov/Taxonomy/Browser/wwwtax.cgi?id=243161) ...................................... 98 [2 hits](http://blast.ncbi.nlm.nih.gov/Blast.cgi?CMD=Get&RID=5Y3VDS5A013&FORMAT_OBJECT=TaxBlast&NCBI_GI=off&DESCRIPTIONS=500&ALIGNMENTS=250&FORMAT_BLOCK_ON_RESPAGE=Top&MASK_COLOR=1&MASK_CHAR=2#243161) [[chlamydias](http://www.ncbi.nlm.nih.gov/Taxonomy/Browser/wwwtax.cgi?id=51291)] [conserved hypothetical protein [Chlamydia muridarum Nigg] >](http://www.ncbi.nlm.nih.gov/entrez/query.fcgi?cmd=Retrieve&db=Protein&list_uids=15834735&dopt=GenPept)
140. . [Oligella ureolytica](http://www.ncbi.nlm.nih.gov/Taxonomy/Browser/wwwtax.cgi?id=90244) ........................................... 97 [1 hit](http://blast.ncbi.nlm.nih.gov/Blast.cgi?CMD=Get&RID=5Y3VDS5A013&FORMAT_OBJECT=TaxBlast&NCBI_GI=off&DESCRIPTIONS=500&ALIGNMENTS=250&FORMAT_BLOCK_ON_RESPAGE=Top&MASK_COLOR=1&MASK_CHAR=2#90244) [[b-proteobacteria](http://www.ncbi.nlm.nih.gov/Taxonomy/Browser/wwwtax.cgi?id=28216)] [hypothetical protein [Oligella ureolytica]](http://www.ncbi.nlm.nih.gov/entrez/query.fcgi?cmd=Retrieve&db=Protein&list_uids=517401304&dopt=GenPept)
141. . [Achromobacter xylosoxidans C54](http://www.ncbi.nlm.nih.gov/Taxonomy/Browser/wwwtax.cgi?id=562971) ................................ 97 [1 hit](http://blast.ncbi.nlm.nih.gov/Blast.cgi?CMD=Get&RID=5Y3VDS5A013&FORMAT_OBJECT=TaxBlast&NCBI_GI=off&DESCRIPTIONS=500&ALIGNMENTS=250&FORMAT_BLOCK_ON_RESPAGE=Top&MASK_COLOR=1&MASK_CHAR=2#562971) [[b-proteobacteria](http://www.ncbi.nlm.nih.gov/Taxonomy/Browser/wwwtax.cgi?id=28216)] [hypothetical protein [Achromobacter xylosoxidans] >gi|31740](http://www.ncbi.nlm.nih.gov/entrez/query.fcgi?cmd=Retrieve&db=Protein&list_uids=317401717&dopt=GenPept)
142. . [Achromobacter piechaudii HLE](http://www.ncbi.nlm.nih.gov/Taxonomy/Browser/wwwtax.cgi?id=1156919) .................................. 97 [1 hit](http://blast.ncbi.nlm.nih.gov/Blast.cgi?CMD=Get&RID=5Y3VDS5A013&FORMAT_OBJECT=TaxBlast&NCBI_GI=off&DESCRIPTIONS=500&ALIGNMENTS=250&FORMAT_BLOCK_ON_RESPAGE=Top&MASK_COLOR=1&MASK_CHAR=2#1156919) [[b-proteobacteria](http://www.ncbi.nlm.nih.gov/Taxonomy/Browser/wwwtax.cgi?id=28216)] [SET domain-containing protein [Achromobacter piechaudii] >g](http://www.ncbi.nlm.nih.gov/entrez/query.fcgi?cmd=Retrieve&db=Protein&list_uids=400194894&dopt=GenPept)
143. . [uncultured bacterium](http://www.ncbi.nlm.nih.gov/Taxonomy/Browser/wwwtax.cgi?id=77133) .......................................... 96 [3 hits](http://blast.ncbi.nlm.nih.gov/Blast.cgi?CMD=Get&RID=5Y3VDS5A013&FORMAT_OBJECT=TaxBlast&NCBI_GI=off&DESCRIPTIONS=500&ALIGNMENTS=250&FORMAT_BLOCK_ON_RESPAGE=Top&MASK_COLOR=1&MASK_CHAR=2#77133) [[bacteria](http://www.ncbi.nlm.nih.gov/Taxonomy/Browser/wwwtax.cgi?id=2)] [Nuclear protein SET [uncultured bacterium]](http://www.ncbi.nlm.nih.gov/entrez/query.fcgi?cmd=Retrieve&db=Protein&list_uids=406992621&dopt=GenPept)
144. . [Achromobacter xylosoxidans NH44784-1996](http://www.ncbi.nlm.nih.gov/Taxonomy/Browser/wwwtax.cgi?id=1167634) ....................... 97 [2 hits](http://blast.ncbi.nlm.nih.gov/Blast.cgi?CMD=Get&RID=5Y3VDS5A013&FORMAT_OBJECT=TaxBlast&NCBI_GI=off&DESCRIPTIONS=500&ALIGNMENTS=250&FORMAT_BLOCK_ON_RESPAGE=Top&MASK_COLOR=1&MASK_CHAR=2#1167634) [[b-proteobacteria](http://www.ncbi.nlm.nih.gov/Taxonomy/Browser/wwwtax.cgi?id=28216)] [Proteins containing SET domain [Achromobacter xylosoxidans](http://www.ncbi.nlm.nih.gov/entrez/query.fcgi?cmd=Retrieve&db=Protein&list_uids=528983917&dopt=GenPept)
145. . [Stenotrophomonas maltophilia](http://www.ncbi.nlm.nih.gov/Taxonomy/Browser/wwwtax.cgi?id=40324) .................................. 97 [11 hits](http://blast.ncbi.nlm.nih.gov/Blast.cgi?CMD=Get&RID=5Y3VDS5A013&FORMAT_OBJECT=TaxBlast&NCBI_GI=off&DESCRIPTIONS=500&ALIGNMENTS=250&FORMAT_BLOCK_ON_RESPAGE=Top&MASK_COLOR=1&MASK_CHAR=2#40324) [[g-proteobacteria](http://www.ncbi.nlm.nih.gov/Taxonomy/Browser/wwwtax.cgi?id=1236)] [Histone-lysine N-methyltransferase SETD1B [Stenotrophomonas](http://www.ncbi.nlm.nih.gov/entrez/query.fcgi?cmd=Retrieve&db=Protein&list_uids=493468028&dopt=GenPept)
146. . [Stenotrophomonas maltophilia SKK35](http://www.ncbi.nlm.nih.gov/Taxonomy/Browser/wwwtax.cgi?id=1118156) ............................ 97 [1 hit](http://blast.ncbi.nlm.nih.gov/Blast.cgi?CMD=Get&RID=5Y3VDS5A013&FORMAT_OBJECT=TaxBlast&NCBI_GI=off&DESCRIPTIONS=500&ALIGNMENTS=250&FORMAT_BLOCK_ON_RESPAGE=Top&MASK_COLOR=1&MASK_CHAR=2#1118156) [[g-proteobacteria](http://www.ncbi.nlm.nih.gov/Taxonomy/Browser/wwwtax.cgi?id=1236)] [Histone-lysine N-methyltransferase SETD1B [Stenotrophomonas](http://www.ncbi.nlm.nih.gov/entrez/query.fcgi?cmd=Retrieve&db=Protein&list_uids=475010697&dopt=GenPept)
147. . [Achromobacter arsenitoxydans](http://www.ncbi.nlm.nih.gov/Taxonomy/Browser/wwwtax.cgi?id=1147684) .................................. 97 [1 hit](http://blast.ncbi.nlm.nih.gov/Blast.cgi?CMD=Get&RID=5Y3VDS5A013&FORMAT_OBJECT=TaxBlast&NCBI_GI=off&DESCRIPTIONS=500&ALIGNMENTS=250&FORMAT_BLOCK_ON_RESPAGE=Top&MASK_COLOR=1&MASK_CHAR=2#1147684) [[b-proteobacteria](http://www.ncbi.nlm.nih.gov/Taxonomy/Browser/wwwtax.cgi?id=28216)] [SET domain-containing protein [Achromobacter arsenitoxydans](http://www.ncbi.nlm.nih.gov/entrez/query.fcgi?cmd=Retrieve&db=Protein&list_uids=495432916&dopt=GenPept)
148. . [Achromobacter arsenitoxydans SY8](http://www.ncbi.nlm.nih.gov/Taxonomy/Browser/wwwtax.cgi?id=477184) .............................. 97 [1 hit](http://blast.ncbi.nlm.nih.gov/Blast.cgi?CMD=Get&RID=5Y3VDS5A013&FORMAT_OBJECT=TaxBlast&NCBI_GI=off&DESCRIPTIONS=500&ALIGNMENTS=250&FORMAT_BLOCK_ON_RESPAGE=Top&MASK_COLOR=1&MASK_CHAR=2#477184) [[b-proteobacteria](http://www.ncbi.nlm.nih.gov/Taxonomy/Browser/wwwtax.cgi?id=28216)] [SET domain-containing protein [Achromobacter arsenitoxydans](http://www.ncbi.nlm.nih.gov/entrez/query.fcgi?cmd=Retrieve&db=Protein&list_uids=359366739&dopt=GenPept)
149. . [Rhodopseudomonas palustris TIE-1](http://www.ncbi.nlm.nih.gov/Taxonomy/Browser/wwwtax.cgi?id=395960) .............................. 98 [2 hits](http://blast.ncbi.nlm.nih.gov/Blast.cgi?CMD=Get&RID=5Y3VDS5A013&FORMAT_OBJECT=TaxBlast&NCBI_GI=off&DESCRIPTIONS=500&ALIGNMENTS=250&FORMAT_BLOCK_ON_RESPAGE=Top&MASK_COLOR=1&MASK_CHAR=2#395960) [[a-proteobacteria](http://www.ncbi.nlm.nih.gov/Taxonomy/Browser/wwwtax.cgi?id=28211)] [nuclear protein SET [Rhodopseudomonas palustris TIE-1] >gi|](http://www.ncbi.nlm.nih.gov/entrez/query.fcgi?cmd=Retrieve&db=Protein&list_uids=192292758&dopt=GenPept)
150. . [Rhodopseudomonas palustris CGA009](http://www.ncbi.nlm.nih.gov/Taxonomy/Browser/wwwtax.cgi?id=258594) ............................. 98 [2 hits](http://blast.ncbi.nlm.nih.gov/Blast.cgi?CMD=Get&RID=5Y3VDS5A013&FORMAT_OBJECT=TaxBlast&NCBI_GI=off&DESCRIPTIONS=500&ALIGNMENTS=250&FORMAT_BLOCK_ON_RESPAGE=Top&MASK_COLOR=1&MASK_CHAR=2#258594) [[a-proteobacteria](http://www.ncbi.nlm.nih.gov/Taxonomy/Browser/wwwtax.cgi?id=28211)] [nuclear protein SET [Rhodopseudomonas palustris CGA009] >gi](http://www.ncbi.nlm.nih.gov/entrez/query.fcgi?cmd=Retrieve&db=Protein&list_uids=39936932&dopt=GenPept)
151. . [Bradyrhizobium sp. WSM471](http://www.ncbi.nlm.nih.gov/Taxonomy/Browser/wwwtax.cgi?id=319017) ..................................... 97 [2 hits](http://blast.ncbi.nlm.nih.gov/Blast.cgi?CMD=Get&RID=5Y3VDS5A013&FORMAT_OBJECT=TaxBlast&NCBI_GI=off&DESCRIPTIONS=500&ALIGNMENTS=250&FORMAT_BLOCK_ON_RESPAGE=Top&MASK_COLOR=1&MASK_CHAR=2#319017) [[a-proteobacteria](http://www.ncbi.nlm.nih.gov/Taxonomy/Browser/wwwtax.cgi?id=28211)] [histone-lysine N-methyltransferase with a SET domain [Brady](http://www.ncbi.nlm.nih.gov/entrez/query.fcgi?cmd=Retrieve&db=Protein&list_uids=494886185&dopt=GenPept)
152. . [Chlamydia psittaci NJ1](http://www.ncbi.nlm.nih.gov/Taxonomy/Browser/wwwtax.cgi?id=1050221) ........................................ 97 [2 hits](http://blast.ncbi.nlm.nih.gov/Blast.cgi?CMD=Get&RID=5Y3VDS5A013&FORMAT_OBJECT=TaxBlast&NCBI_GI=off&DESCRIPTIONS=500&ALIGNMENTS=250&FORMAT_BLOCK_ON_RESPAGE=Top&MASK_COLOR=1&MASK_CHAR=2#1050221) [[chlamydias](http://www.ncbi.nlm.nih.gov/Taxonomy/Browser/wwwtax.cgi?id=51291)] [SET domain-containing protein [Chlamydia psittaci NJ1] >gi|](http://www.ncbi.nlm.nih.gov/entrez/query.fcgi?cmd=Retrieve&db=Protein&list_uids=406593837&dopt=GenPept)
153. . [Chlamydia psittaci](http://www.ncbi.nlm.nih.gov/Taxonomy/Browser/wwwtax.cgi?id=83554) ............................................ 97 [8 hits](http://blast.ncbi.nlm.nih.gov/Blast.cgi?CMD=Get&RID=5Y3VDS5A013&FORMAT_OBJECT=TaxBlast&NCBI_GI=off&DESCRIPTIONS=500&ALIGNMENTS=250&FORMAT_BLOCK_ON_RESPAGE=Top&MASK_COLOR=1&MASK_CHAR=2#83554) [[chlamydias](http://www.ncbi.nlm.nih.gov/Taxonomy/Browser/wwwtax.cgi?id=51291)] [SET domain-containing protein [Chlamydia psittaci NJ1] >gi|](http://www.ncbi.nlm.nih.gov/entrez/query.fcgi?cmd=Retrieve&db=Protein&list_uids=504759822&dopt=GenPept)
154. . [Chlamydia psittaci M56](http://www.ncbi.nlm.nih.gov/Taxonomy/Browser/wwwtax.cgi?id=1218357) ........................................ 97 [2 hits](http://blast.ncbi.nlm.nih.gov/Blast.cgi?CMD=Get&RID=5Y3VDS5A013&FORMAT_OBJECT=TaxBlast&NCBI_GI=off&DESCRIPTIONS=500&ALIGNMENTS=250&FORMAT_BLOCK_ON_RESPAGE=Top&MASK_COLOR=1&MASK_CHAR=2#1218357) [[chlamydias](http://www.ncbi.nlm.nih.gov/Taxonomy/Browser/wwwtax.cgi?id=51291)] [SET domain-containing protein [Chlamydia psittaci M56] >gi|](http://www.ncbi.nlm.nih.gov/entrez/query.fcgi?cmd=Retrieve&db=Protein&list_uids=407459705&dopt=GenPept)
155. . [Achromobacter xylosoxidans AXX-A](http://www.ncbi.nlm.nih.gov/Taxonomy/Browser/wwwtax.cgi?id=1003200) .............................. 98 [1 hit](http://blast.ncbi.nlm.nih.gov/Blast.cgi?CMD=Get&RID=5Y3VDS5A013&FORMAT_OBJECT=TaxBlast&NCBI_GI=off&DESCRIPTIONS=500&ALIGNMENTS=250&FORMAT_BLOCK_ON_RESPAGE=Top&MASK_COLOR=1&MASK_CHAR=2#1003200) [[b-proteobacteria](http://www.ncbi.nlm.nih.gov/Taxonomy/Browser/wwwtax.cgi?id=28216)] [lysine methyltransferase [Achromobacter xylosoxidans] >gi|3](http://www.ncbi.nlm.nih.gov/entrez/query.fcgi?cmd=Retrieve&db=Protein&list_uids=338781972&dopt=GenPept)
156. . [Chlamydia psittaci 6BC](http://www.ncbi.nlm.nih.gov/Taxonomy/Browser/wwwtax.cgi?id=331636) ........................................ 97 [4 hits](http://blast.ncbi.nlm.nih.gov/Blast.cgi?CMD=Get&RID=5Y3VDS5A013&FORMAT_OBJECT=TaxBlast&NCBI_GI=off&DESCRIPTIONS=500&ALIGNMENTS=250&FORMAT_BLOCK_ON_RESPAGE=Top&MASK_COLOR=1&MASK_CHAR=2#331636) [[chlamydias](http://www.ncbi.nlm.nih.gov/Taxonomy/Browser/wwwtax.cgi?id=51291)] [SET domain-containing protein [Chlamydia psittaci 6BC] >gi|](http://www.ncbi.nlm.nih.gov/entrez/query.fcgi?cmd=Retrieve&db=Protein&list_uids=332287805&dopt=GenPept)
157. . [Chlamydia psittaci RD1](http://www.ncbi.nlm.nih.gov/Taxonomy/Browser/wwwtax.cgi?id=929557) ........................................ 97 [2 hits](http://blast.ncbi.nlm.nih.gov/Blast.cgi?CMD=Get&RID=5Y3VDS5A013&FORMAT_OBJECT=TaxBlast&NCBI_GI=off&DESCRIPTIONS=500&ALIGNMENTS=250&FORMAT_BLOCK_ON_RESPAGE=Top&MASK_COLOR=1&MASK_CHAR=2#929557) [[chlamydias](http://www.ncbi.nlm.nih.gov/Taxonomy/Browser/wwwtax.cgi?id=51291)] [SET domain-containing protein [Chlamydia psittaci 6BC] >gi|](http://www.ncbi.nlm.nih.gov/entrez/query.fcgi?cmd=Retrieve&db=Protein&list_uids=392377034&dopt=GenPept)
158. . [Chlamydia psittaci 84/55](http://www.ncbi.nlm.nih.gov/Taxonomy/Browser/wwwtax.cgi?id=1218176) ...................................... 97 [2 hits](http://blast.ncbi.nlm.nih.gov/Blast.cgi?CMD=Get&RID=5Y3VDS5A013&FORMAT_OBJECT=TaxBlast&NCBI_GI=off&DESCRIPTIONS=500&ALIGNMENTS=250&FORMAT_BLOCK_ON_RESPAGE=Top&MASK_COLOR=1&MASK_CHAR=2#1218176) [[chlamydias](http://www.ncbi.nlm.nih.gov/Taxonomy/Browser/wwwtax.cgi?id=51291)] [SET domain-containing protein [Chlamydia psittaci 6BC] >gi|](http://www.ncbi.nlm.nih.gov/entrez/query.fcgi?cmd=Retrieve&db=Protein&list_uids=407454457&dopt=GenPept)
159. . [Chlamydia psittaci GR9](http://www.ncbi.nlm.nih.gov/Taxonomy/Browser/wwwtax.cgi?id=1218353) ........................................ 97 [2 hits](http://blast.ncbi.nlm.nih.gov/Blast.cgi?CMD=Get&RID=5Y3VDS5A013&FORMAT_OBJECT=TaxBlast&NCBI_GI=off&DESCRIPTIONS=500&ALIGNMENTS=250&FORMAT_BLOCK_ON_RESPAGE=Top&MASK_COLOR=1&MASK_CHAR=2#1218353) [[chlamydias](http://www.ncbi.nlm.nih.gov/Taxonomy/Browser/wwwtax.cgi?id=51291)] [SET domain-containing protein [Chlamydia psittaci 6BC] >gi|](http://www.ncbi.nlm.nih.gov/entrez/query.fcgi?cmd=Retrieve&db=Protein&list_uids=407455726&dopt=GenPept)
160. . [Chlamydia psittaci WS/RT/E30](http://www.ncbi.nlm.nih.gov/Taxonomy/Browser/wwwtax.cgi?id=1218356) .................................. 97 [2 hits](http://blast.ncbi.nlm.nih.gov/Blast.cgi?CMD=Get&RID=5Y3VDS5A013&FORMAT_OBJECT=TaxBlast&NCBI_GI=off&DESCRIPTIONS=500&ALIGNMENTS=250&FORMAT_BLOCK_ON_RESPAGE=Top&MASK_COLOR=1&MASK_CHAR=2#1218356) [[chlamydias](http://www.ncbi.nlm.nih.gov/Taxonomy/Browser/wwwtax.cgi?id=51291)] [SET domain-containing protein [Chlamydia psittaci 6BC] >gi|](http://www.ncbi.nlm.nih.gov/entrez/query.fcgi?cmd=Retrieve&db=Protein&list_uids=407458465&dopt=GenPept)
161. . [Chlamydia psittaci WC](http://www.ncbi.nlm.nih.gov/Taxonomy/Browser/wwwtax.cgi?id=1218358) ......................................... 97 [2 hits](http://blast.ncbi.nlm.nih.gov/Blast.cgi?CMD=Get&RID=5Y3VDS5A013&FORMAT_OBJECT=TaxBlast&NCBI_GI=off&DESCRIPTIONS=500&ALIGNMENTS=250&FORMAT_BLOCK_ON_RESPAGE=Top&MASK_COLOR=1&MASK_CHAR=2#1218358) [[chlamydias](http://www.ncbi.nlm.nih.gov/Taxonomy/Browser/wwwtax.cgi?id=51291)] [SET domain-containing protein [Chlamydia psittaci 6BC] >gi|](http://www.ncbi.nlm.nih.gov/entrez/query.fcgi?cmd=Retrieve&db=Protein&list_uids=407461079&dopt=GenPept)
162. . [Chlamydia psittaci 02DC16](http://www.ncbi.nlm.nih.gov/Taxonomy/Browser/wwwtax.cgi?id=1112255) ..................................... 97 [1 hit](http://blast.ncbi.nlm.nih.gov/Blast.cgi?CMD=Get&RID=5Y3VDS5A013&FORMAT_OBJECT=TaxBlast&NCBI_GI=off&DESCRIPTIONS=500&ALIGNMENTS=250&FORMAT_BLOCK_ON_RESPAGE=Top&MASK_COLOR=1&MASK_CHAR=2#1112255) [[chlamydias](http://www.ncbi.nlm.nih.gov/Taxonomy/Browser/wwwtax.cgi?id=51291)] [SET domain-containing protein [Chlamydia psittaci 6BC] >gi|](http://www.ncbi.nlm.nih.gov/entrez/query.fcgi?cmd=Retrieve&db=Protein&list_uids=514657422&dopt=GenPept)
163. . [Chlamydia psittaci 02DC22](http://www.ncbi.nlm.nih.gov/Taxonomy/Browser/wwwtax.cgi?id=1112258) ..................................... 97 [1 hit](http://blast.ncbi.nlm.nih.gov/Blast.cgi?CMD=Get&RID=5Y3VDS5A013&FORMAT_OBJECT=TaxBlast&NCBI_GI=off&DESCRIPTIONS=500&ALIGNMENTS=250&FORMAT_BLOCK_ON_RESPAGE=Top&MASK_COLOR=1&MASK_CHAR=2#1112258) [[chlamydias](http://www.ncbi.nlm.nih.gov/Taxonomy/Browser/wwwtax.cgi?id=51291)] [SET domain-containing protein [Chlamydia psittaci 6BC] >gi|](http://www.ncbi.nlm.nih.gov/entrez/query.fcgi?cmd=Retrieve&db=Protein&list_uids=514660839&dopt=GenPept)
164. . [Chlamydia psittaci 01DC11](http://www.ncbi.nlm.nih.gov/Taxonomy/Browser/wwwtax.cgi?id=1112252) ..................................... 97 [3 hits](http://blast.ncbi.nlm.nih.gov/Blast.cgi?CMD=Get&RID=5Y3VDS5A013&FORMAT_OBJECT=TaxBlast&NCBI_GI=off&DESCRIPTIONS=500&ALIGNMENTS=250&FORMAT_BLOCK_ON_RESPAGE=Top&MASK_COLOR=1&MASK_CHAR=2#1112252) [[chlamydias](http://www.ncbi.nlm.nih.gov/Taxonomy/Browser/wwwtax.cgi?id=51291)] [SET domain-containing protein [Chlamydia psittaci 6BC] >gi|](http://www.ncbi.nlm.nih.gov/entrez/query.fcgi?cmd=Retrieve&db=Protein&list_uids=514661744&dopt=GenPept)
165. . [Chlamydia psittaci 03DC29](http://www.ncbi.nlm.nih.gov/Taxonomy/Browser/wwwtax.cgi?id=1112262) ..................................... 97 [1 hit](http://blast.ncbi.nlm.nih.gov/Blast.cgi?CMD=Get&RID=5Y3VDS5A013&FORMAT_OBJECT=TaxBlast&NCBI_GI=off&DESCRIPTIONS=500&ALIGNMENTS=250&FORMAT_BLOCK_ON_RESPAGE=Top&MASK_COLOR=1&MASK_CHAR=2#1112262) [[chlamydias](http://www.ncbi.nlm.nih.gov/Taxonomy/Browser/wwwtax.cgi?id=51291)] [SET domain-containing protein [Chlamydia psittaci 6BC] >gi|](http://www.ncbi.nlm.nih.gov/entrez/query.fcgi?cmd=Retrieve&db=Protein&list_uids=514663233&dopt=GenPept)
166. . [Chlamydia psittaci C19/98](http://www.ncbi.nlm.nih.gov/Taxonomy/Browser/wwwtax.cgi?id=1112250) ..................................... 97 [3 hits](http://blast.ncbi.nlm.nih.gov/Blast.cgi?CMD=Get&RID=5Y3VDS5A013&FORMAT_OBJECT=TaxBlast&NCBI_GI=off&DESCRIPTIONS=500&ALIGNMENTS=250&FORMAT_BLOCK_ON_RESPAGE=Top&MASK_COLOR=1&MASK_CHAR=2#1112250) [[chlamydias](http://www.ncbi.nlm.nih.gov/Taxonomy/Browser/wwwtax.cgi?id=51291)] [SET domain-containing protein [Chlamydia psittaci 6BC] >gi|](http://www.ncbi.nlm.nih.gov/entrez/query.fcgi?cmd=Retrieve&db=Protein&list_uids=514673335&dopt=GenPept)
167. . [Chlamydia psittaci 06-1683](http://www.ncbi.nlm.nih.gov/Taxonomy/Browser/wwwtax.cgi?id=1112244) .................................... 97 [1 hit](http://blast.ncbi.nlm.nih.gov/Blast.cgi?CMD=Get&RID=5Y3VDS5A013&FORMAT_OBJECT=TaxBlast&NCBI_GI=off&DESCRIPTIONS=500&ALIGNMENTS=250&FORMAT_BLOCK_ON_RESPAGE=Top&MASK_COLOR=1&MASK_CHAR=2#1112244) [[chlamydias](http://www.ncbi.nlm.nih.gov/Taxonomy/Browser/wwwtax.cgi?id=51291)] [SET domain-containing protein [Chlamydia psittaci 6BC] >gi|](http://www.ncbi.nlm.nih.gov/entrez/query.fcgi?cmd=Retrieve&db=Protein&list_uids=514676274&dopt=GenPept)
168. . [Chlamydia psittaci 02DC24](http://www.ncbi.nlm.nih.gov/Taxonomy/Browser/wwwtax.cgi?id=1112260) ..................................... 97 [1 hit](http://blast.ncbi.nlm.nih.gov/Blast.cgi?CMD=Get&RID=5Y3VDS5A013&FORMAT_OBJECT=TaxBlast&NCBI_GI=off&DESCRIPTIONS=500&ALIGNMENTS=250&FORMAT_BLOCK_ON_RESPAGE=Top&MASK_COLOR=1&MASK_CHAR=2#1112260) [[chlamydias](http://www.ncbi.nlm.nih.gov/Taxonomy/Browser/wwwtax.cgi?id=51291)] [SET domain-containing protein [Chlamydia psittaci 6BC] >gi|](http://www.ncbi.nlm.nih.gov/entrez/query.fcgi?cmd=Retrieve&db=Protein&list_uids=515450001&dopt=GenPept)
169. . [Chlamydia psittaci 08-2626_L3](http://www.ncbi.nlm.nih.gov/Taxonomy/Browser/wwwtax.cgi?id=1112246) ................................. 97 [1 hit](http://blast.ncbi.nlm.nih.gov/Blast.cgi?CMD=Get&RID=5Y3VDS5A013&FORMAT_OBJECT=TaxBlast&NCBI_GI=off&DESCRIPTIONS=500&ALIGNMENTS=250&FORMAT_BLOCK_ON_RESPAGE=Top&MASK_COLOR=1&MASK_CHAR=2#1112246) [[chlamydias](http://www.ncbi.nlm.nih.gov/Taxonomy/Browser/wwwtax.cgi?id=51291)] [SET domain-containing protein [Chlamydia psittaci 6BC] >gi|](http://www.ncbi.nlm.nih.gov/entrez/query.fcgi?cmd=Retrieve&db=Protein&list_uids=519781983&dopt=GenPept)
170. . [Chlamydia psittaci C6/98](http://www.ncbi.nlm.nih.gov/Taxonomy/Browser/wwwtax.cgi?id=1112249) ...................................... 97 [1 hit](http://blast.ncbi.nlm.nih.gov/Blast.cgi?CMD=Get&RID=5Y3VDS5A013&FORMAT_OBJECT=TaxBlast&NCBI_GI=off&DESCRIPTIONS=500&ALIGNMENTS=250&FORMAT_BLOCK_ON_RESPAGE=Top&MASK_COLOR=1&MASK_CHAR=2#1112249) [[chlamydias](http://www.ncbi.nlm.nih.gov/Taxonomy/Browser/wwwtax.cgi?id=51291)] [SET domain-containing protein [Chlamydia psittaci 6BC] >gi|](http://www.ncbi.nlm.nih.gov/entrez/query.fcgi?cmd=Retrieve&db=Protein&list_uids=519789136&dopt=GenPept)
171. . [Chlamydia psittaci 08DC60](http://www.ncbi.nlm.nih.gov/Taxonomy/Browser/wwwtax.cgi?id=1112267) ..................................... 97 [3 hits](http://blast.ncbi.nlm.nih.gov/Blast.cgi?CMD=Get&RID=5Y3VDS5A013&FORMAT_OBJECT=TaxBlast&NCBI_GI=off&DESCRIPTIONS=500&ALIGNMENTS=250&FORMAT_BLOCK_ON_RESPAGE=Top&MASK_COLOR=1&MASK_CHAR=2#1112267) [[chlamydias](http://www.ncbi.nlm.nih.gov/Taxonomy/Browser/wwwtax.cgi?id=51291)] [SET domain-containing protein [Chlamydia psittaci 01DC11] >](http://www.ncbi.nlm.nih.gov/entrez/query.fcgi?cmd=Retrieve&db=Protein&list_uids=384452933&dopt=GenPept)
172. . [Chlamydia psittaci 02DC15](http://www.ncbi.nlm.nih.gov/Taxonomy/Browser/wwwtax.cgi?id=1112254) ..................................... 97 [3 hits](http://blast.ncbi.nlm.nih.gov/Blast.cgi?CMD=Get&RID=5Y3VDS5A013&FORMAT_OBJECT=TaxBlast&NCBI_GI=off&DESCRIPTIONS=500&ALIGNMENTS=250&FORMAT_BLOCK_ON_RESPAGE=Top&MASK_COLOR=1&MASK_CHAR=2#1112254) [[chlamydias](http://www.ncbi.nlm.nih.gov/Taxonomy/Browser/wwwtax.cgi?id=51291)] [SET domain-containing protein [Chlamydia psittaci 01DC11] >](http://www.ncbi.nlm.nih.gov/entrez/query.fcgi?cmd=Retrieve&db=Protein&list_uids=384454891&dopt=GenPept)
173. . [Chlamydia psittaci VS225](http://www.ncbi.nlm.nih.gov/Taxonomy/Browser/wwwtax.cgi?id=1218355) ...................................... 97 [2 hits](http://blast.ncbi.nlm.nih.gov/Blast.cgi?CMD=Get&RID=5Y3VDS5A013&FORMAT_OBJECT=TaxBlast&NCBI_GI=off&DESCRIPTIONS=500&ALIGNMENTS=250&FORMAT_BLOCK_ON_RESPAGE=Top&MASK_COLOR=1&MASK_CHAR=2#1218355) [[chlamydias](http://www.ncbi.nlm.nih.gov/Taxonomy/Browser/wwwtax.cgi?id=51291)] [SET domain-containing protein [Chlamydia psittaci 01DC11] >](http://www.ncbi.nlm.nih.gov/entrez/query.fcgi?cmd=Retrieve&db=Protein&list_uids=407457142&dopt=GenPept)
174. . [Chlamydia psittaci Cal10](http://www.ncbi.nlm.nih.gov/Taxonomy/Browser/wwwtax.cgi?id=984894) ...................................... 97 [1 hit](http://blast.ncbi.nlm.nih.gov/Blast.cgi?CMD=Get&RID=5Y3VDS5A013&FORMAT_OBJECT=TaxBlast&NCBI_GI=off&DESCRIPTIONS=500&ALIGNMENTS=250&FORMAT_BLOCK_ON_RESPAGE=Top&MASK_COLOR=1&MASK_CHAR=2#984894) [[chlamydias](http://www.ncbi.nlm.nih.gov/Taxonomy/Browser/wwwtax.cgi?id=51291)] [SET domain-containing protein [Chlamydia psittaci 01DC11] >](http://www.ncbi.nlm.nih.gov/entrez/query.fcgi?cmd=Retrieve&db=Protein&list_uids=328814772&dopt=GenPept)
175. . [Chlamydia psittaci 02DC18](http://www.ncbi.nlm.nih.gov/Taxonomy/Browser/wwwtax.cgi?id=1112256) ..................................... 97 [1 hit](http://blast.ncbi.nlm.nih.gov/Blast.cgi?CMD=Get&RID=5Y3VDS5A013&FORMAT_OBJECT=TaxBlast&NCBI_GI=off&DESCRIPTIONS=500&ALIGNMENTS=250&FORMAT_BLOCK_ON_RESPAGE=Top&MASK_COLOR=1&MASK_CHAR=2#1112256) [[chlamydias](http://www.ncbi.nlm.nih.gov/Taxonomy/Browser/wwwtax.cgi?id=51291)] [SET domain-containing protein [Chlamydia psittaci 01DC11] >](http://www.ncbi.nlm.nih.gov/entrez/query.fcgi?cmd=Retrieve&db=Protein&list_uids=514659496&dopt=GenPept)
176. . [Chlamydia psittaci 02DC23](http://www.ncbi.nlm.nih.gov/Taxonomy/Browser/wwwtax.cgi?id=1112259) ..................................... 97 [1 hit](http://blast.ncbi.nlm.nih.gov/Blast.cgi?CMD=Get&RID=5Y3VDS5A013&FORMAT_OBJECT=TaxBlast&NCBI_GI=off&DESCRIPTIONS=500&ALIGNMENTS=250&FORMAT_BLOCK_ON_RESPAGE=Top&MASK_COLOR=1&MASK_CHAR=2#1112259) [[chlamydias](http://www.ncbi.nlm.nih.gov/Taxonomy/Browser/wwwtax.cgi?id=51291)] [SET domain-containing protein [Chlamydia psittaci 01DC11] >](http://www.ncbi.nlm.nih.gov/entrez/query.fcgi?cmd=Retrieve&db=Protein&list_uids=514663564&dopt=GenPept)
177. . [Chlamydia psittaci 02DC21](http://www.ncbi.nlm.nih.gov/Taxonomy/Browser/wwwtax.cgi?id=1112257) ..................................... 97 [1 hit](http://blast.ncbi.nlm.nih.gov/Blast.cgi?CMD=Get&RID=5Y3VDS5A013&FORMAT_OBJECT=TaxBlast&NCBI_GI=off&DESCRIPTIONS=500&ALIGNMENTS=250&FORMAT_BLOCK_ON_RESPAGE=Top&MASK_COLOR=1&MASK_CHAR=2#1112257) [[chlamydias](http://www.ncbi.nlm.nih.gov/Taxonomy/Browser/wwwtax.cgi?id=51291)] [SET domain-containing protein [Chlamydia psittaci 01DC11] >](http://www.ncbi.nlm.nih.gov/entrez/query.fcgi?cmd=Retrieve&db=Protein&list_uids=514664692&dopt=GenPept)
178. . [Chlamydia psittaci 04DC42](http://www.ncbi.nlm.nih.gov/Taxonomy/Browser/wwwtax.cgi?id=1112265) ..................................... 97 [1 hit](http://blast.ncbi.nlm.nih.gov/Blast.cgi?CMD=Get&RID=5Y3VDS5A013&FORMAT_OBJECT=TaxBlast&NCBI_GI=off&DESCRIPTIONS=500&ALIGNMENTS=250&FORMAT_BLOCK_ON_RESPAGE=Top&MASK_COLOR=1&MASK_CHAR=2#1112265) [[chlamydias](http://www.ncbi.nlm.nih.gov/Taxonomy/Browser/wwwtax.cgi?id=51291)] [SET domain-containing protein [Chlamydia psittaci 01DC11] >](http://www.ncbi.nlm.nih.gov/entrez/query.fcgi?cmd=Retrieve&db=Protein&list_uids=514666640&dopt=GenPept)
179. . [Chlamydia psittaci 99DC5](http://www.ncbi.nlm.nih.gov/Taxonomy/Browser/wwwtax.cgi?id=1112251) ...................................... 97 [1 hit](http://blast.ncbi.nlm.nih.gov/Blast.cgi?CMD=Get&RID=5Y3VDS5A013&FORMAT_OBJECT=TaxBlast&NCBI_GI=off&DESCRIPTIONS=500&ALIGNMENTS=250&FORMAT_BLOCK_ON_RESPAGE=Top&MASK_COLOR=1&MASK_CHAR=2#1112251) [[chlamydias](http://www.ncbi.nlm.nih.gov/Taxonomy/Browser/wwwtax.cgi?id=51291)] [SET domain-containing protein [Chlamydia psittaci 01DC11] >](http://www.ncbi.nlm.nih.gov/entrez/query.fcgi?cmd=Retrieve&db=Protein&list_uids=514672980&dopt=GenPept)
180. . [Chlamydia psittaci 03DC35](http://www.ncbi.nlm.nih.gov/Taxonomy/Browser/wwwtax.cgi?id=1112264) ..................................... 97 [1 hit](http://blast.ncbi.nlm.nih.gov/Blast.cgi?CMD=Get&RID=5Y3VDS5A013&FORMAT_OBJECT=TaxBlast&NCBI_GI=off&DESCRIPTIONS=500&ALIGNMENTS=250&FORMAT_BLOCK_ON_RESPAGE=Top&MASK_COLOR=1&MASK_CHAR=2#1112264) [[chlamydias](http://www.ncbi.nlm.nih.gov/Taxonomy/Browser/wwwtax.cgi?id=51291)] [SET domain-containing protein [Chlamydia psittaci 01DC11] >](http://www.ncbi.nlm.nih.gov/entrez/query.fcgi?cmd=Retrieve&db=Protein&list_uids=514676605&dopt=GenPept)
181. . [Chlamydia psittaci 02DC14](http://www.ncbi.nlm.nih.gov/Taxonomy/Browser/wwwtax.cgi?id=1112253) ..................................... 97 [1 hit](http://blast.ncbi.nlm.nih.gov/Blast.cgi?CMD=Get&RID=5Y3VDS5A013&FORMAT_OBJECT=TaxBlast&NCBI_GI=off&DESCRIPTIONS=500&ALIGNMENTS=250&FORMAT_BLOCK_ON_RESPAGE=Top&MASK_COLOR=1&MASK_CHAR=2#1112253) [[chlamydias](http://www.ncbi.nlm.nih.gov/Taxonomy/Browser/wwwtax.cgi?id=51291)] [SET domain-containing protein [Chlamydia psittaci 01DC11] >](http://www.ncbi.nlm.nih.gov/entrez/query.fcgi?cmd=Retrieve&db=Protein&list_uids=515451307&dopt=GenPept)
182. . [Chlamydia psittaci C1/97](http://www.ncbi.nlm.nih.gov/Taxonomy/Browser/wwwtax.cgi?id=1112248) ...................................... 97 [1 hit](http://blast.ncbi.nlm.nih.gov/Blast.cgi?CMD=Get&RID=5Y3VDS5A013&FORMAT_OBJECT=TaxBlast&NCBI_GI=off&DESCRIPTIONS=500&ALIGNMENTS=250&FORMAT_BLOCK_ON_RESPAGE=Top&MASK_COLOR=1&MASK_CHAR=2#1112248) [[chlamydias](http://www.ncbi.nlm.nih.gov/Taxonomy/Browser/wwwtax.cgi?id=51291)] [SET domain-containing protein [Chlamydia psittaci 01DC11] >](http://www.ncbi.nlm.nih.gov/entrez/query.fcgi?cmd=Retrieve&db=Protein&list_uids=519784778&dopt=GenPept)
183. . [Bradyrhizobium elkanii](http://www.ncbi.nlm.nih.gov/Taxonomy/Browser/wwwtax.cgi?id=29448) ........................................ 97 [1 hit](http://blast.ncbi.nlm.nih.gov/Blast.cgi?CMD=Get&RID=5Y3VDS5A013&FORMAT_OBJECT=TaxBlast&NCBI_GI=off&DESCRIPTIONS=500&ALIGNMENTS=250&FORMAT_BLOCK_ON_RESPAGE=Top&MASK_COLOR=1&MASK_CHAR=2#29448) [[a-proteobacteria](http://www.ncbi.nlm.nih.gov/Taxonomy/Browser/wwwtax.cgi?id=28211)] [hypothetical protein [Bradyrhizobium elkanii]](http://www.ncbi.nlm.nih.gov/entrez/query.fcgi?cmd=Retrieve&db=Protein&list_uids=517083634&dopt=GenPept)
184. . [Chlamydia psittaci CP3](http://www.ncbi.nlm.nih.gov/Taxonomy/Browser/wwwtax.cgi?id=1050219) ........................................ 97 [2 hits](http://blast.ncbi.nlm.nih.gov/Blast.cgi?CMD=Get&RID=5Y3VDS5A013&FORMAT_OBJECT=TaxBlast&NCBI_GI=off&DESCRIPTIONS=500&ALIGNMENTS=250&FORMAT_BLOCK_ON_RESPAGE=Top&MASK_COLOR=1&MASK_CHAR=2#1050219) [[chlamydias](http://www.ncbi.nlm.nih.gov/Taxonomy/Browser/wwwtax.cgi?id=51291)] [SET domain-containing protein [Chlamydia psittaci CP3] >gi|](http://www.ncbi.nlm.nih.gov/entrez/query.fcgi?cmd=Retrieve&db=Protein&list_uids=406592781&dopt=GenPept)
185. . [Chlamydia psittaci MN](http://www.ncbi.nlm.nih.gov/Taxonomy/Browser/wwwtax.cgi?id=1218354) ......................................... 97 [2 hits](http://blast.ncbi.nlm.nih.gov/Blast.cgi?CMD=Get&RID=5Y3VDS5A013&FORMAT_OBJECT=TaxBlast&NCBI_GI=off&DESCRIPTIONS=500&ALIGNMENTS=250&FORMAT_BLOCK_ON_RESPAGE=Top&MASK_COLOR=1&MASK_CHAR=2#1218354) [[chlamydias](http://www.ncbi.nlm.nih.gov/Taxonomy/Browser/wwwtax.cgi?id=51291)] [SET domain-containing protein [Chlamydia psittaci CP3] >gi|](http://www.ncbi.nlm.nih.gov/entrez/query.fcgi?cmd=Retrieve&db=Protein&list_uids=406594501&dopt=GenPept)
186. . [Chlamydia psittaci 01DC12](http://www.ncbi.nlm.nih.gov/Taxonomy/Browser/wwwtax.cgi?id=1221877) ..................................... 97 [2 hits](http://blast.ncbi.nlm.nih.gov/Blast.cgi?CMD=Get&RID=5Y3VDS5A013&FORMAT_OBJECT=TaxBlast&NCBI_GI=off&DESCRIPTIONS=500&ALIGNMENTS=250&FORMAT_BLOCK_ON_RESPAGE=Top&MASK_COLOR=1&MASK_CHAR=2#1221877) [[chlamydias](http://www.ncbi.nlm.nih.gov/Taxonomy/Browser/wwwtax.cgi?id=51291)] [SET domain-containing protein [Chlamydia psittaci CP3] >gi|](http://www.ncbi.nlm.nih.gov/entrez/query.fcgi?cmd=Retrieve&db=Protein&list_uids=410858817&dopt=GenPept)
187. . [Chlamydia psittaci 09DC77](http://www.ncbi.nlm.nih.gov/Taxonomy/Browser/wwwtax.cgi?id=1112268) ..................................... 97 [1 hit](http://blast.ncbi.nlm.nih.gov/Blast.cgi?CMD=Get&RID=5Y3VDS5A013&FORMAT_OBJECT=TaxBlast&NCBI_GI=off&DESCRIPTIONS=500&ALIGNMENTS=250&FORMAT_BLOCK_ON_RESPAGE=Top&MASK_COLOR=1&MASK_CHAR=2#1112268) [[chlamydias](http://www.ncbi.nlm.nih.gov/Taxonomy/Browser/wwwtax.cgi?id=51291)] [SET domain-containing protein [Chlamydia psittaci CP3] >gi|](http://www.ncbi.nlm.nih.gov/entrez/query.fcgi?cmd=Retrieve&db=Protein&list_uids=514669785&dopt=GenPept)
188. . [Chlamydia psittaci 09DC80](http://www.ncbi.nlm.nih.gov/Taxonomy/Browser/wwwtax.cgi?id=1112271) ..................................... 97 [1 hit](http://blast.ncbi.nlm.nih.gov/Blast.cgi?CMD=Get&RID=5Y3VDS5A013&FORMAT_OBJECT=TaxBlast&NCBI_GI=off&DESCRIPTIONS=500&ALIGNMENTS=250&FORMAT_BLOCK_ON_RESPAGE=Top&MASK_COLOR=1&MASK_CHAR=2#1112271) [[chlamydias](http://www.ncbi.nlm.nih.gov/Taxonomy/Browser/wwwtax.cgi?id=51291)] [SET domain-containing protein [Chlamydia psittaci CP3] >gi|](http://www.ncbi.nlm.nih.gov/entrez/query.fcgi?cmd=Retrieve&db=Protein&list_uids=514671415&dopt=GenPept)
189. . [Chlamydia psittaci 09DC78](http://www.ncbi.nlm.nih.gov/Taxonomy/Browser/wwwtax.cgi?id=1112269) ..................................... 97 [1 hit](http://blast.ncbi.nlm.nih.gov/Blast.cgi?CMD=Get&RID=5Y3VDS5A013&FORMAT_OBJECT=TaxBlast&NCBI_GI=off&DESCRIPTIONS=500&ALIGNMENTS=250&FORMAT_BLOCK_ON_RESPAGE=Top&MASK_COLOR=1&MASK_CHAR=2#1112269) [[chlamydias](http://www.ncbi.nlm.nih.gov/Taxonomy/Browser/wwwtax.cgi?id=51291)] [SET domain-containing protein [Chlamydia psittaci CP3] >gi|](http://www.ncbi.nlm.nih.gov/entrez/query.fcgi?cmd=Retrieve&db=Protein&list_uids=514675608&dopt=GenPept)
190. . [Chlamydia psittaci 09DC79](http://www.ncbi.nlm.nih.gov/Taxonomy/Browser/wwwtax.cgi?id=1112270) ..................................... 97 [1 hit](http://blast.ncbi.nlm.nih.gov/Blast.cgi?CMD=Get&RID=5Y3VDS5A013&FORMAT_OBJECT=TaxBlast&NCBI_GI=off&DESCRIPTIONS=500&ALIGNMENTS=250&FORMAT_BLOCK_ON_RESPAGE=Top&MASK_COLOR=1&MASK_CHAR=2#1112270) [[chlamydias](http://www.ncbi.nlm.nih.gov/Taxonomy/Browser/wwwtax.cgi?id=51291)] [SET domain-containing protein [Chlamydia psittaci CP3] >gi|](http://www.ncbi.nlm.nih.gov/entrez/query.fcgi?cmd=Retrieve&db=Protein&list_uids=515453458&dopt=GenPept)
191. . [Xanthomonas vasicola](http://www.ncbi.nlm.nih.gov/Taxonomy/Browser/wwwtax.cgi?id=56459) .......................................... 95 [1 hit](http://blast.ncbi.nlm.nih.gov/Blast.cgi?CMD=Get&RID=5Y3VDS5A013&FORMAT_OBJECT=TaxBlast&NCBI_GI=off&DESCRIPTIONS=500&ALIGNMENTS=250&FORMAT_BLOCK_ON_RESPAGE=Top&MASK_COLOR=1&MASK_CHAR=2#56459) [[g-proteobacteria](http://www.ncbi.nlm.nih.gov/Taxonomy/Browser/wwwtax.cgi?id=1236)] [nuclear protein SET [Xanthomonas vasicola]](http://www.ncbi.nlm.nih.gov/entrez/query.fcgi?cmd=Retrieve&db=Protein&list_uids=498055148&dopt=GenPept)
192. . [Stenotrophomonas maltophilia Ab55555](http://www.ncbi.nlm.nih.gov/Taxonomy/Browser/wwwtax.cgi?id=1183154) .......................... 96 [1 hit](http://blast.ncbi.nlm.nih.gov/Blast.cgi?CMD=Get&RID=5Y3VDS5A013&FORMAT_OBJECT=TaxBlast&NCBI_GI=off&DESCRIPTIONS=500&ALIGNMENTS=250&FORMAT_BLOCK_ON_RESPAGE=Top&MASK_COLOR=1&MASK_CHAR=2#1183154) [[g-proteobacteria](http://www.ncbi.nlm.nih.gov/Taxonomy/Browser/wwwtax.cgi?id=1236)] [hypothetical protein [Stenotrophomonas maltophilia] >gi|401](http://www.ncbi.nlm.nih.gov/entrez/query.fcgi?cmd=Retrieve&db=Protein&list_uids=401069070&dopt=GenPept)
193. . [Herbaspirillum sp. JC206](http://www.ncbi.nlm.nih.gov/Taxonomy/Browser/wwwtax.cgi?id=1095769) ...................................... 95 [1 hit](http://blast.ncbi.nlm.nih.gov/Blast.cgi?CMD=Get&RID=5Y3VDS5A013&FORMAT_OBJECT=TaxBlast&NCBI_GI=off&DESCRIPTIONS=500&ALIGNMENTS=250&FORMAT_BLOCK_ON_RESPAGE=Top&MASK_COLOR=1&MASK_CHAR=2#1095769) [[b-proteobacteria](http://www.ncbi.nlm.nih.gov/Taxonomy/Browser/wwwtax.cgi?id=28216)] [hypothetical protein [Herbaspirillum sp. JC206]](http://www.ncbi.nlm.nih.gov/entrez/query.fcgi?cmd=Retrieve&db=Protein&list_uids=517972052&dopt=GenPept)
194. . [Chlamydophila sp. 08-1274/3](http://www.ncbi.nlm.nih.gov/Taxonomy/Browser/wwwtax.cgi?id=1351837) ................................... 97 [2 hits](http://blast.ncbi.nlm.nih.gov/Blast.cgi?CMD=Get&RID=5Y3VDS5A013&FORMAT_OBJECT=TaxBlast&NCBI_GI=off&DESCRIPTIONS=500&ALIGNMENTS=250&FORMAT_BLOCK_ON_RESPAGE=Top&MASK_COLOR=1&MASK_CHAR=2#1351837) [[chlamydias](http://www.ncbi.nlm.nih.gov/Taxonomy/Browser/wwwtax.cgi?id=51291)] [SET domain protein [Chlamydophila sp. 08-1274/3] >gi|545672](http://www.ncbi.nlm.nih.gov/entrez/query.fcgi?cmd=Retrieve&db=Protein&list_uids=546198437&dopt=GenPept)
195. . [Chlamydophila caviae GPIC](http://www.ncbi.nlm.nih.gov/Taxonomy/Browser/wwwtax.cgi?id=227941) ..................................... 97 [2 hits](http://blast.ncbi.nlm.nih.gov/Blast.cgi?CMD=Get&RID=5Y3VDS5A013&FORMAT_OBJECT=TaxBlast&NCBI_GI=off&DESCRIPTIONS=500&ALIGNMENTS=250&FORMAT_BLOCK_ON_RESPAGE=Top&MASK_COLOR=1&MASK_CHAR=2#227941) [[chlamydias](http://www.ncbi.nlm.nih.gov/Taxonomy/Browser/wwwtax.cgi?id=51291)] [SET domain-containing protein [Chlamydophila caviae GPIC] >](http://www.ncbi.nlm.nih.gov/entrez/query.fcgi?cmd=Retrieve&db=Protein&list_uids=29840645&dopt=GenPept)
196. . [Chlamydophila caviae](http://www.ncbi.nlm.nih.gov/Taxonomy/Browser/wwwtax.cgi?id=83557) .......................................... 97 [1 hit](http://blast.ncbi.nlm.nih.gov/Blast.cgi?CMD=Get&RID=5Y3VDS5A013&FORMAT_OBJECT=TaxBlast&NCBI_GI=off&DESCRIPTIONS=500&ALIGNMENTS=250&FORMAT_BLOCK_ON_RESPAGE=Top&MASK_COLOR=1&MASK_CHAR=2#83557) [[chlamydias](http://www.ncbi.nlm.nih.gov/Taxonomy/Browser/wwwtax.cgi?id=51291)] [SET domain-containing protein [Chlamydophila caviae GPIC] >](http://www.ncbi.nlm.nih.gov/entrez/query.fcgi?cmd=Retrieve&db=Protein&list_uids=499316351&dopt=GenPept)
197. . Chlamydophila pneumoniae J138 1 hit [[chlamydias](http://www.ncbi.nlm.nih.gov/Taxonomy/Browser/wwwtax.cgi?id=51291)] SET domain protein BAA99086 [Chlamydophila pneumoniae J138]
198. . [Verrucomicrobium spinosum](http://www.ncbi.nlm.nih.gov/Taxonomy/Browser/wwwtax.cgi?id=2736) ..................................... 97 [1 hit](http://blast.ncbi.nlm.nih.gov/Blast.cgi?CMD=Get&RID=5Y3VDS5A013&FORMAT_OBJECT=TaxBlast&NCBI_GI=off&DESCRIPTIONS=500&ALIGNMENTS=250&FORMAT_BLOCK_ON_RESPAGE=Top&MASK_COLOR=1&MASK_CHAR=2#2736) [[verrucomicrobia](http://www.ncbi.nlm.nih.gov/Taxonomy/Browser/wwwtax.cgi?id=74201)] [Nuclear protein SET [Verrucomicrobium spinosum]](http://www.ncbi.nlm.nih.gov/entrez/query.fcgi?cmd=Retrieve&db=Protein&list_uids=497644944&dopt=GenPept)
199. . [Chlamydophila abortus S26/3](http://www.ncbi.nlm.nih.gov/Taxonomy/Browser/wwwtax.cgi?id=218497) ................................... 97 [2 hits](http://blast.ncbi.nlm.nih.gov/Blast.cgi?CMD=Get&RID=5Y3VDS5A013&FORMAT_OBJECT=TaxBlast&NCBI_GI=off&DESCRIPTIONS=500&ALIGNMENTS=250&FORMAT_BLOCK_ON_RESPAGE=Top&MASK_COLOR=1&MASK_CHAR=2#218497) [[chlamydias](http://www.ncbi.nlm.nih.gov/Taxonomy/Browser/wwwtax.cgi?id=51291)] [hypothetical protein CAB857 [Chlamydophila abortus S26/3] >](http://www.ncbi.nlm.nih.gov/entrez/query.fcgi?cmd=Retrieve&db=Protein&list_uids=62185459&dopt=GenPept)
200. . [Chlamydophila abortus](http://www.ncbi.nlm.nih.gov/Taxonomy/Browser/wwwtax.cgi?id=83555) ......................................... 97 [2 hits](http://blast.ncbi.nlm.nih.gov/Blast.cgi?CMD=Get&RID=5Y3VDS5A013&FORMAT_OBJECT=TaxBlast&NCBI_GI=off&DESCRIPTIONS=500&ALIGNMENTS=250&FORMAT_BLOCK_ON_RESPAGE=Top&MASK_COLOR=1&MASK_CHAR=2#83555) [[chlamydias](http://www.ncbi.nlm.nih.gov/Taxonomy/Browser/wwwtax.cgi?id=51291)] [hypothetical protein CAB857 [Chlamydophila abortus S26/3] >](http://www.ncbi.nlm.nih.gov/entrez/query.fcgi?cmd=Retrieve&db=Protein&list_uids=499409914&dopt=GenPept)
201. . [Xanthomonas axonopodis](http://www.ncbi.nlm.nih.gov/Taxonomy/Browser/wwwtax.cgi?id=53413) ........................................ 95 [3 hits](http://blast.ncbi.nlm.nih.gov/Blast.cgi?CMD=Get&RID=5Y3VDS5A013&FORMAT_OBJECT=TaxBlast&NCBI_GI=off&DESCRIPTIONS=500&ALIGNMENTS=250&FORMAT_BLOCK_ON_RESPAGE=Top&MASK_COLOR=1&MASK_CHAR=2#53413) [[g-proteobacteria](http://www.ncbi.nlm.nih.gov/Taxonomy/Browser/wwwtax.cgi?id=1236)] [SET domain-containing protein [Xanthomonas axonopodis] >gi|](http://www.ncbi.nlm.nih.gov/entrez/query.fcgi?cmd=Retrieve&db=Protein&list_uids=492656715&dopt=GenPept)
202. . [Xanthomonas axonopodis pv. malvacearum str. GSPB2388](http://www.ncbi.nlm.nih.gov/Taxonomy/Browser/wwwtax.cgi?id=1127439) .......... 95 [1 hit](http://blast.ncbi.nlm.nih.gov/Blast.cgi?CMD=Get&RID=5Y3VDS5A013&FORMAT_OBJECT=TaxBlast&NCBI_GI=off&DESCRIPTIONS=500&ALIGNMENTS=250&FORMAT_BLOCK_ON_RESPAGE=Top&MASK_COLOR=1&MASK_CHAR=2#1127439) [[g-proteobacteria](http://www.ncbi.nlm.nih.gov/Taxonomy/Browser/wwwtax.cgi?id=1236)] [SET domain-containing protein [Xanthomonas axonopodis] >gi|](http://www.ncbi.nlm.nih.gov/entrez/query.fcgi?cmd=Retrieve&db=Protein&list_uids=410705000&dopt=GenPept)
203. . [Xanthomonas axonopodis pv. malvacearum str. GSPB1386](http://www.ncbi.nlm.nih.gov/Taxonomy/Browser/wwwtax.cgi?id=1118965) .......... 95 [1 hit](http://blast.ncbi.nlm.nih.gov/Blast.cgi?CMD=Get&RID=5Y3VDS5A013&FORMAT_OBJECT=TaxBlast&NCBI_GI=off&DESCRIPTIONS=500&ALIGNMENTS=250&FORMAT_BLOCK_ON_RESPAGE=Top&MASK_COLOR=1&MASK_CHAR=2#1118965) [[g-proteobacteria](http://www.ncbi.nlm.nih.gov/Taxonomy/Browser/wwwtax.cgi?id=1236)] [SET domain-containing protein [Xanthomonas axonopodis] >gi|](http://www.ncbi.nlm.nih.gov/entrez/query.fcgi?cmd=Retrieve&db=Protein&list_uids=410706658&dopt=GenPept)
204. . [Rhodanobacter sp. 115](http://www.ncbi.nlm.nih.gov/Taxonomy/Browser/wwwtax.cgi?id=1162282) ......................................... 95 [2 hits](http://blast.ncbi.nlm.nih.gov/Blast.cgi?CMD=Get&RID=5Y3VDS5A013&FORMAT_OBJECT=TaxBlast&NCBI_GI=off&DESCRIPTIONS=500&ALIGNMENTS=250&FORMAT_BLOCK_ON_RESPAGE=Top&MASK_COLOR=1&MASK_CHAR=2#1162282) [[g-proteobacteria](http://www.ncbi.nlm.nih.gov/Taxonomy/Browser/wwwtax.cgi?id=1236)] [SET domain-containing protein [Rhodanobacter sp. 115] >gi|3](http://www.ncbi.nlm.nih.gov/entrez/query.fcgi?cmd=Retrieve&db=Protein&list_uids=495483855&dopt=GenPept)
205. . [Nitrobacter winogradskyi Nb-255](http://www.ncbi.nlm.nih.gov/Taxonomy/Browser/wwwtax.cgi?id=323098) ............................... 96 [2 hits](http://blast.ncbi.nlm.nih.gov/Blast.cgi?CMD=Get&RID=5Y3VDS5A013&FORMAT_OBJECT=TaxBlast&NCBI_GI=off&DESCRIPTIONS=500&ALIGNMENTS=250&FORMAT_BLOCK_ON_RESPAGE=Top&MASK_COLOR=1&MASK_CHAR=2#323098) [[a-proteobacteria](http://www.ncbi.nlm.nih.gov/Taxonomy/Browser/wwwtax.cgi?id=28211)] [nuclear protein SET [Nitrobacter winogradskyi Nb-255] >gi|4](http://www.ncbi.nlm.nih.gov/entrez/query.fcgi?cmd=Retrieve&db=Protein&list_uids=75676346&dopt=GenPept)
206. . [Nitrobacter winogradskyi](http://www.ncbi.nlm.nih.gov/Taxonomy/Browser/wwwtax.cgi?id=913) ...................................... 96 [1 hit](http://blast.ncbi.nlm.nih.gov/Blast.cgi?CMD=Get&RID=5Y3VDS5A013&FORMAT_OBJECT=TaxBlast&NCBI_GI=off&DESCRIPTIONS=500&ALIGNMENTS=250&FORMAT_BLOCK_ON_RESPAGE=Top&MASK_COLOR=1&MASK_CHAR=2#913) [[a-proteobacteria](http://www.ncbi.nlm.nih.gov/Taxonomy/Browser/wwwtax.cgi?id=28211)] [nuclear protein SET [Nitrobacter winogradskyi Nb-255] >gi|4](http://www.ncbi.nlm.nih.gov/entrez/query.fcgi?cmd=Retrieve&db=Protein&list_uids=499634650&dopt=GenPept)
207. . [Stenotrophomonas maltophilia K279a](http://www.ncbi.nlm.nih.gov/Taxonomy/Browser/wwwtax.cgi?id=522373) ............................ 95 [2 hits](http://blast.ncbi.nlm.nih.gov/Blast.cgi?CMD=Get&RID=5Y3VDS5A013&FORMAT_OBJECT=TaxBlast&NCBI_GI=off&DESCRIPTIONS=500&ALIGNMENTS=250&FORMAT_BLOCK_ON_RESPAGE=Top&MASK_COLOR=1&MASK_CHAR=2#522373) [[g-proteobacteria](http://www.ncbi.nlm.nih.gov/Taxonomy/Browser/wwwtax.cgi?id=1236)] [hypothetical protein Smlt1159 [Stenotrophomonas maltophilia](http://www.ncbi.nlm.nih.gov/entrez/query.fcgi?cmd=Retrieve&db=Protein&list_uids=190573184&dopt=GenPept)
208. . [Stenotrophomonas maltophilia D457](http://www.ncbi.nlm.nih.gov/Taxonomy/Browser/wwwtax.cgi?id=1163399) ............................. 95 [2 hits](http://blast.ncbi.nlm.nih.gov/Blast.cgi?CMD=Get&RID=5Y3VDS5A013&FORMAT_OBJECT=TaxBlast&NCBI_GI=off&DESCRIPTIONS=500&ALIGNMENTS=250&FORMAT_BLOCK_ON_RESPAGE=Top&MASK_COLOR=1&MASK_CHAR=2#1163399) [[g-proteobacteria](http://www.ncbi.nlm.nih.gov/Taxonomy/Browser/wwwtax.cgi?id=1236)] [hypothetical protein Smlt1159 [Stenotrophomonas maltophilia](http://www.ncbi.nlm.nih.gov/entrez/query.fcgi?cmd=Retrieve&db=Protein&list_uids=386717496&dopt=GenPept)
209. . [Stenotrophomonas maltophilia JV3](http://www.ncbi.nlm.nih.gov/Taxonomy/Browser/wwwtax.cgi?id=868597) .............................. 95 [2 hits](http://blast.ncbi.nlm.nih.gov/Blast.cgi?CMD=Get&RID=5Y3VDS5A013&FORMAT_OBJECT=TaxBlast&NCBI_GI=off&DESCRIPTIONS=500&ALIGNMENTS=250&FORMAT_BLOCK_ON_RESPAGE=Top&MASK_COLOR=1&MASK_CHAR=2#868597) [[g-proteobacteria](http://www.ncbi.nlm.nih.gov/Taxonomy/Browser/wwwtax.cgi?id=1236)] [nuclear protein SET [Stenotrophomonas maltophilia JV3] >gi|](http://www.ncbi.nlm.nih.gov/entrez/query.fcgi?cmd=Retrieve&db=Protein&list_uids=344206415&dopt=GenPept)
210. . [Stenotrophomonas](http://www.ncbi.nlm.nih.gov/Taxonomy/Browser/wwwtax.cgi?id=40323) .............................................. 95 [1 hit](http://blast.ncbi.nlm.nih.gov/Blast.cgi?CMD=Get&RID=5Y3VDS5A013&FORMAT_OBJECT=TaxBlast&NCBI_GI=off&DESCRIPTIONS=500&ALIGNMENTS=250&FORMAT_BLOCK_ON_RESPAGE=Top&MASK_COLOR=1&MASK_CHAR=2#40323) [[g-proteobacteria](http://www.ncbi.nlm.nih.gov/Taxonomy/Browser/wwwtax.cgi?id=1236)] [nuclear protein SET [Stenotrophomonas maltophilia JV3] >gi|](http://www.ncbi.nlm.nih.gov/entrez/query.fcgi?cmd=Retrieve&db=Protein&list_uids=495542259&dopt=GenPept)
211. . [Stenotrophomonas sp. SKA14](http://www.ncbi.nlm.nih.gov/Taxonomy/Browser/wwwtax.cgi?id=391601) .................................... 95 [1 hit](http://blast.ncbi.nlm.nih.gov/Blast.cgi?CMD=Get&RID=5Y3VDS5A013&FORMAT_OBJECT=TaxBlast&NCBI_GI=off&DESCRIPTIONS=500&ALIGNMENTS=250&FORMAT_BLOCK_ON_RESPAGE=Top&MASK_COLOR=1&MASK_CHAR=2#391601) [[g-proteobacteria](http://www.ncbi.nlm.nih.gov/Taxonomy/Browser/wwwtax.cgi?id=1236)] [nuclear protein SET [Stenotrophomonas maltophilia JV3] >gi|](http://www.ncbi.nlm.nih.gov/entrez/query.fcgi?cmd=Retrieve&db=Protein&list_uids=219720926&dopt=GenPept)
212. . [Nitrobacter hamburgensis X14](http://www.ncbi.nlm.nih.gov/Taxonomy/Browser/wwwtax.cgi?id=323097) .................................. 96 [2 hits](http://blast.ncbi.nlm.nih.gov/Blast.cgi?CMD=Get&RID=5Y3VDS5A013&FORMAT_OBJECT=TaxBlast&NCBI_GI=off&DESCRIPTIONS=500&ALIGNMENTS=250&FORMAT_BLOCK_ON_RESPAGE=Top&MASK_COLOR=1&MASK_CHAR=2#323097) [[a-proteobacteria](http://www.ncbi.nlm.nih.gov/Taxonomy/Browser/wwwtax.cgi?id=28211)] [nuclear protein SET [Nitrobacter hamburgensis X14] >gi|4998](http://www.ncbi.nlm.nih.gov/entrez/query.fcgi?cmd=Retrieve&db=Protein&list_uids=92118074&dopt=GenPept)
213. . [Nitrobacter hamburgensis](http://www.ncbi.nlm.nih.gov/Taxonomy/Browser/wwwtax.cgi?id=912) ...................................... 96 [1 hit](http://blast.ncbi.nlm.nih.gov/Blast.cgi?CMD=Get&RID=5Y3VDS5A013&FORMAT_OBJECT=TaxBlast&NCBI_GI=off&DESCRIPTIONS=500&ALIGNMENTS=250&FORMAT_BLOCK_ON_RESPAGE=Top&MASK_COLOR=1&MASK_CHAR=2#912) [[a-proteobacteria](http://www.ncbi.nlm.nih.gov/Taxonomy/Browser/wwwtax.cgi?id=28211)] [nuclear protein SET [Nitrobacter hamburgensis X14] >gi|4998](http://www.ncbi.nlm.nih.gov/entrez/query.fcgi?cmd=Retrieve&db=Protein&list_uids=499830276&dopt=GenPept)
214. . [Chlorobium luteolum DSM 273](http://www.ncbi.nlm.nih.gov/Taxonomy/Browser/wwwtax.cgi?id=319225) ................................... 95 [2 hits](http://blast.ncbi.nlm.nih.gov/Blast.cgi?CMD=Get&RID=5Y3VDS5A013&FORMAT_OBJECT=TaxBlast&NCBI_GI=off&DESCRIPTIONS=500&ALIGNMENTS=250&FORMAT_BLOCK_ON_RESPAGE=Top&MASK_COLOR=1&MASK_CHAR=2#319225) [[green sulfur bacteria](http://www.ncbi.nlm.nih.gov/Taxonomy/Browser/wwwtax.cgi?id=1090)] [nuclear protein SET [Chlorobium luteolum DSM 273] >gi|49967](http://www.ncbi.nlm.nih.gov/entrez/query.fcgi?cmd=Retrieve&db=Protein&list_uids=78186988&dopt=GenPept)
215. . [Pelodictyon luteolum](http://www.ncbi.nlm.nih.gov/Taxonomy/Browser/wwwtax.cgi?id=1100) .......................................... 95 [1 hit](http://blast.ncbi.nlm.nih.gov/Blast.cgi?CMD=Get&RID=5Y3VDS5A013&FORMAT_OBJECT=TaxBlast&NCBI_GI=off&DESCRIPTIONS=500&ALIGNMENTS=250&FORMAT_BLOCK_ON_RESPAGE=Top&MASK_COLOR=1&MASK_CHAR=2#1100) [[green sulfur bacteria](http://www.ncbi.nlm.nih.gov/Taxonomy/Browser/wwwtax.cgi?id=1090)] [nuclear protein SET [Chlorobium luteolum DSM 273] >gi|49967](http://www.ncbi.nlm.nih.gov/entrez/query.fcgi?cmd=Retrieve&db=Protein&list_uids=499677126&dopt=GenPept)
216. . [Chlamydophila abortus LLG](http://www.ncbi.nlm.nih.gov/Taxonomy/Browser/wwwtax.cgi?id=1003238) ..................................... 97 [1 hit](http://blast.ncbi.nlm.nih.gov/Blast.cgi?CMD=Get&RID=5Y3VDS5A013&FORMAT_OBJECT=TaxBlast&NCBI_GI=off&DESCRIPTIONS=500&ALIGNMENTS=250&FORMAT_BLOCK_ON_RESPAGE=Top&MASK_COLOR=1&MASK_CHAR=2#1003238) [[chlamydias](http://www.ncbi.nlm.nih.gov/Taxonomy/Browser/wwwtax.cgi?id=51291)] [lysine methyltransferase [Chlamydophila abortus] >gi|333410](http://www.ncbi.nlm.nih.gov/entrez/query.fcgi?cmd=Retrieve&db=Protein&list_uids=333410601&dopt=GenPept)
217. . [Acidovorax sp. JS42](http://www.ncbi.nlm.nih.gov/Taxonomy/Browser/wwwtax.cgi?id=232721) ........................................... 97 [3 hits](http://blast.ncbi.nlm.nih.gov/Blast.cgi?CMD=Get&RID=5Y3VDS5A013&FORMAT_OBJECT=TaxBlast&NCBI_GI=off&DESCRIPTIONS=500&ALIGNMENTS=250&FORMAT_BLOCK_ON_RESPAGE=Top&MASK_COLOR=1&MASK_CHAR=2#232721) [[b-proteobacteria](http://www.ncbi.nlm.nih.gov/Taxonomy/Browser/wwwtax.cgi?id=28216)] [nuclear protein SET [Acidovorax sp. JS42] >gi|500131069|ref](http://www.ncbi.nlm.nih.gov/entrez/query.fcgi?cmd=Retrieve&db=Protein&list_uids=121596275&dopt=GenPept)
218. . [Rhodopseudomonas palustris BisB18](http://www.ncbi.nlm.nih.gov/Taxonomy/Browser/wwwtax.cgi?id=316056) ............................. 97 [2 hits](http://blast.ncbi.nlm.nih.gov/Blast.cgi?CMD=Get&RID=5Y3VDS5A013&FORMAT_OBJECT=TaxBlast&NCBI_GI=off&DESCRIPTIONS=500&ALIGNMENTS=250&FORMAT_BLOCK_ON_RESPAGE=Top&MASK_COLOR=1&MASK_CHAR=2#316056) [[a-proteobacteria](http://www.ncbi.nlm.nih.gov/Taxonomy/Browser/wwwtax.cgi?id=28211)] [nuclear protein SET [Rhodopseudomonas palustris BisB18] >gi](http://www.ncbi.nlm.nih.gov/entrez/query.fcgi?cmd=Retrieve&db=Protein&list_uids=90423049&dopt=GenPept)
219. . [Thiomonas intermedia K12](http://www.ncbi.nlm.nih.gov/Taxonomy/Browser/wwwtax.cgi?id=75379) ...................................... 95 [2 hits](http://blast.ncbi.nlm.nih.gov/Blast.cgi?CMD=Get&RID=5Y3VDS5A013&FORMAT_OBJECT=TaxBlast&NCBI_GI=off&DESCRIPTIONS=500&ALIGNMENTS=250&FORMAT_BLOCK_ON_RESPAGE=Top&MASK_COLOR=1&MASK_CHAR=2#75379) [[b-proteobacteria](http://www.ncbi.nlm.nih.gov/Taxonomy/Browser/wwwtax.cgi?id=28216)] [nuclear protein SET [Thiomonas intermedia K12] >gi|50288735](http://www.ncbi.nlm.nih.gov/entrez/query.fcgi?cmd=Retrieve&db=Protein&list_uids=296135069&dopt=GenPept)
220. . [Thiomonas intermedia](http://www.ncbi.nlm.nih.gov/Taxonomy/Browser/wwwtax.cgi?id=926) .......................................... 95 [1 hit](http://blast.ncbi.nlm.nih.gov/Blast.cgi?CMD=Get&RID=5Y3VDS5A013&FORMAT_OBJECT=TaxBlast&NCBI_GI=off&DESCRIPTIONS=500&ALIGNMENTS=250&FORMAT_BLOCK_ON_RESPAGE=Top&MASK_COLOR=1&MASK_CHAR=2#926) [[b-proteobacteria](http://www.ncbi.nlm.nih.gov/Taxonomy/Browser/wwwtax.cgi?id=28216)] [nuclear protein SET [Thiomonas intermedia K12] >gi|50288735](http://www.ncbi.nlm.nih.gov/entrez/query.fcgi?cmd=Retrieve&db=Protein&list_uids=502887357&dopt=GenPept)
221. . [Xanthomonas albilineans GPE PC73](http://www.ncbi.nlm.nih.gov/Taxonomy/Browser/wwwtax.cgi?id=380358) .............................. 95 [2 hits](http://blast.ncbi.nlm.nih.gov/Blast.cgi?CMD=Get&RID=5Y3VDS5A013&FORMAT_OBJECT=TaxBlast&NCBI_GI=off&DESCRIPTIONS=500&ALIGNMENTS=250&FORMAT_BLOCK_ON_RESPAGE=Top&MASK_COLOR=1&MASK_CHAR=2#380358) [[g-proteobacteria](http://www.ncbi.nlm.nih.gov/Taxonomy/Browser/wwwtax.cgi?id=1236)] [hypothetical protein XALc_1033 [Xanthomonas albilineans GPE](http://www.ncbi.nlm.nih.gov/entrez/query.fcgi?cmd=Retrieve&db=Protein&list_uids=285017825&dopt=GenPept)
222. . [Xanthomonas albilineans](http://www.ncbi.nlm.nih.gov/Taxonomy/Browser/wwwtax.cgi?id=29447) ....................................... 95 [1 hit](http://blast.ncbi.nlm.nih.gov/Blast.cgi?CMD=Get&RID=5Y3VDS5A013&FORMAT_OBJECT=TaxBlast&NCBI_GI=off&DESCRIPTIONS=500&ALIGNMENTS=250&FORMAT_BLOCK_ON_RESPAGE=Top&MASK_COLOR=1&MASK_CHAR=2#29447) [[g-proteobacteria](http://www.ncbi.nlm.nih.gov/Taxonomy/Browser/wwwtax.cgi?id=1236)] [hypothetical protein XALc_1033 [Xanthomonas albilineans GPE](http://www.ncbi.nlm.nih.gov/entrez/query.fcgi?cmd=Retrieve&db=Protein&list_uids=502679895&dopt=GenPept)
223. . [Xanthomonas campestris pv. vesicatoria str. 85-10](http://www.ncbi.nlm.nih.gov/Taxonomy/Browser/wwwtax.cgi?id=316273) ............. 95 [2 hits](http://blast.ncbi.nlm.nih.gov/Blast.cgi?CMD=Get&RID=5Y3VDS5A013&FORMAT_OBJECT=TaxBlast&NCBI_GI=off&DESCRIPTIONS=500&ALIGNMENTS=250&FORMAT_BLOCK_ON_RESPAGE=Top&MASK_COLOR=1&MASK_CHAR=2#316273) [[g-proteobacteria](http://www.ncbi.nlm.nih.gov/Taxonomy/Browser/wwwtax.cgi?id=1236)] [hypothetical protein XCV3077 [Xanthomonas campestris pv. ve](http://www.ncbi.nlm.nih.gov/entrez/query.fcgi?cmd=Retrieve&db=Protein&list_uids=78048633&dopt=GenPept)
224. . [Xanthomonas euvesicatoria](http://www.ncbi.nlm.nih.gov/Taxonomy/Browser/wwwtax.cgi?id=456327) ..................................... 95 [1 hit](http://blast.ncbi.nlm.nih.gov/Blast.cgi?CMD=Get&RID=5Y3VDS5A013&FORMAT_OBJECT=TaxBlast&NCBI_GI=off&DESCRIPTIONS=500&ALIGNMENTS=250&FORMAT_BLOCK_ON_RESPAGE=Top&MASK_COLOR=1&MASK_CHAR=2#456327) [[g-proteobacteria](http://www.ncbi.nlm.nih.gov/Taxonomy/Browser/wwwtax.cgi?id=1236)] [hypothetical protein XCV3077 [Xanthomonas campestris pv. ve](http://www.ncbi.nlm.nih.gov/entrez/query.fcgi?cmd=Retrieve&db=Protein&list_uids=499667382&dopt=GenPept)
225. . [Comamonas testosteroni](http://www.ncbi.nlm.nih.gov/Taxonomy/Browser/wwwtax.cgi?id=285) ........................................ 95 [3 hits](http://blast.ncbi.nlm.nih.gov/Blast.cgi?CMD=Get&RID=5Y3VDS5A013&FORMAT_OBJECT=TaxBlast&NCBI_GI=off&DESCRIPTIONS=500&ALIGNMENTS=250&FORMAT_BLOCK_ON_RESPAGE=Top&MASK_COLOR=1&MASK_CHAR=2#285) [[b-proteobacteria](http://www.ncbi.nlm.nih.gov/Taxonomy/Browser/wwwtax.cgi?id=28216)] [nuclear protein SET [Comamonas testosteroni] >gi|371454647|](http://www.ncbi.nlm.nih.gov/entrez/query.fcgi?cmd=Retrieve&db=Protein&list_uids=489162517&dopt=GenPept)
226. . [Comamonas testosteroni ATCC 11996](http://www.ncbi.nlm.nih.gov/Taxonomy/Browser/wwwtax.cgi?id=1009852) ............................. 95 [1 hit](http://blast.ncbi.nlm.nih.gov/Blast.cgi?CMD=Get&RID=5Y3VDS5A013&FORMAT_OBJECT=TaxBlast&NCBI_GI=off&DESCRIPTIONS=500&ALIGNMENTS=250&FORMAT_BLOCK_ON_RESPAGE=Top&MASK_COLOR=1&MASK_CHAR=2#1009852) [[b-proteobacteria](http://www.ncbi.nlm.nih.gov/Taxonomy/Browser/wwwtax.cgi?id=28216)] [nuclear protein SET [Comamonas testosteroni] >gi|371454647|](http://www.ncbi.nlm.nih.gov/entrez/query.fcgi?cmd=Retrieve&db=Protein&list_uids=371454647&dopt=GenPept)
227. . [Xanthomonas oryzae pv. oryzae KACC 10331](http://www.ncbi.nlm.nih.gov/Taxonomy/Browser/wwwtax.cgi?id=291331) ...................... 95 [2 hits](http://blast.ncbi.nlm.nih.gov/Blast.cgi?CMD=Get&RID=5Y3VDS5A013&FORMAT_OBJECT=TaxBlast&NCBI_GI=off&DESCRIPTIONS=500&ALIGNMENTS=250&FORMAT_BLOCK_ON_RESPAGE=Top&MASK_COLOR=1&MASK_CHAR=2#291331) [[g-proteobacteria](http://www.ncbi.nlm.nih.gov/Taxonomy/Browser/wwwtax.cgi?id=1236)] [hypothetical protein XOO1407 [Xanthomonas oryzae pv. oryzae](http://www.ncbi.nlm.nih.gov/entrez/query.fcgi?cmd=Retrieve&db=Protein&list_uids=58581030&dopt=GenPept)
228. . [Xanthomonas oryzae pv. oryzae PXO99A](http://www.ncbi.nlm.nih.gov/Taxonomy/Browser/wwwtax.cgi?id=360094) .......................... 95 [2 hits](http://blast.ncbi.nlm.nih.gov/Blast.cgi?CMD=Get&RID=5Y3VDS5A013&FORMAT_OBJECT=TaxBlast&NCBI_GI=off&DESCRIPTIONS=500&ALIGNMENTS=250&FORMAT_BLOCK_ON_RESPAGE=Top&MASK_COLOR=1&MASK_CHAR=2#360094) [[g-proteobacteria](http://www.ncbi.nlm.nih.gov/Taxonomy/Browser/wwwtax.cgi?id=1236)] [hypothetical protein XOO1407 [Xanthomonas oryzae pv. oryzae](http://www.ncbi.nlm.nih.gov/entrez/query.fcgi?cmd=Retrieve&db=Protein&list_uids=188577994&dopt=GenPept)
229. . [Xanthomonas axonopodis pv. citrumelo F1](http://www.ncbi.nlm.nih.gov/Taxonomy/Browser/wwwtax.cgi?id=981368) ....................... 95 [2 hits](http://blast.ncbi.nlm.nih.gov/Blast.cgi?CMD=Get&RID=5Y3VDS5A013&FORMAT_OBJECT=TaxBlast&NCBI_GI=off&DESCRIPTIONS=500&ALIGNMENTS=250&FORMAT_BLOCK_ON_RESPAGE=Top&MASK_COLOR=1&MASK_CHAR=2#981368) [[g-proteobacteria](http://www.ncbi.nlm.nih.gov/Taxonomy/Browser/wwwtax.cgi?id=1236)] [hypothetical protein XOO1407 [Xanthomonas oryzae pv. oryzae](http://www.ncbi.nlm.nih.gov/entrez/query.fcgi?cmd=Retrieve&db=Protein&list_uids=346725747&dopt=GenPept)
230. . [Xanthomonas oryzae pv. oryzicola BLS256](http://www.ncbi.nlm.nih.gov/Taxonomy/Browser/wwwtax.cgi?id=383407) ....................... 95 [2 hits](http://blast.ncbi.nlm.nih.gov/Blast.cgi?CMD=Get&RID=5Y3VDS5A013&FORMAT_OBJECT=TaxBlast&NCBI_GI=off&DESCRIPTIONS=500&ALIGNMENTS=250&FORMAT_BLOCK_ON_RESPAGE=Top&MASK_COLOR=1&MASK_CHAR=2#383407) [[g-proteobacteria](http://www.ncbi.nlm.nih.gov/Taxonomy/Browser/wwwtax.cgi?id=1236)] [hypothetical protein XOO1407 [Xanthomonas oryzae pv. oryzae](http://www.ncbi.nlm.nih.gov/entrez/query.fcgi?cmd=Retrieve&db=Protein&list_uids=384418465&dopt=GenPept)
231. . [Xanthomonas axonopodis Xac29-1](http://www.ncbi.nlm.nih.gov/Taxonomy/Browser/wwwtax.cgi?id=1304892) ................................ 95 [2 hits](http://blast.ncbi.nlm.nih.gov/Blast.cgi?CMD=Get&RID=5Y3VDS5A013&FORMAT_OBJECT=TaxBlast&NCBI_GI=off&DESCRIPTIONS=500&ALIGNMENTS=250&FORMAT_BLOCK_ON_RESPAGE=Top&MASK_COLOR=1&MASK_CHAR=2#1304892) [[g-proteobacteria](http://www.ncbi.nlm.nih.gov/Taxonomy/Browser/wwwtax.cgi?id=1236)] [hypothetical protein XOO1407 [Xanthomonas oryzae pv. oryzae](http://www.ncbi.nlm.nih.gov/entrez/query.fcgi?cmd=Retrieve&db=Protein&list_uids=470472801&dopt=GenPept)
232. . [Xanthomonas citri subsp. citri Aw12879](http://www.ncbi.nlm.nih.gov/Taxonomy/Browser/wwwtax.cgi?id=1137651) ........................ 95 [2 hits](http://blast.ncbi.nlm.nih.gov/Blast.cgi?CMD=Get&RID=5Y3VDS5A013&FORMAT_OBJECT=TaxBlast&NCBI_GI=off&DESCRIPTIONS=500&ALIGNMENTS=250&FORMAT_BLOCK_ON_RESPAGE=Top&MASK_COLOR=1&MASK_CHAR=2#1137651) [[g-proteobacteria](http://www.ncbi.nlm.nih.gov/Taxonomy/Browser/wwwtax.cgi?id=1236)] [hypothetical protein XOO1407 [Xanthomonas oryzae pv. oryzae](http://www.ncbi.nlm.nih.gov/entrez/query.fcgi?cmd=Retrieve&db=Protein&list_uids=471268719&dopt=GenPept)
233. . [Xanthomonas](http://www.ncbi.nlm.nih.gov/Taxonomy/Browser/wwwtax.cgi?id=338) ................................................... 95 [2 hits](http://blast.ncbi.nlm.nih.gov/Blast.cgi?CMD=Get&RID=5Y3VDS5A013&FORMAT_OBJECT=TaxBlast&NCBI_GI=off&DESCRIPTIONS=500&ALIGNMENTS=250&FORMAT_BLOCK_ON_RESPAGE=Top&MASK_COLOR=1&MASK_CHAR=2#338) [[g-proteobacteria](http://www.ncbi.nlm.nih.gov/Taxonomy/Browser/wwwtax.cgi?id=1236)] [hypothetical protein XOO1407 [Xanthomonas oryzae pv. oryzae](http://www.ncbi.nlm.nih.gov/entrez/query.fcgi?cmd=Retrieve&db=Protein&list_uids=489583311&dopt=GenPept)
234. . [Xanthomonas fuscans subsp. aurantifolii str. ICPB 11122](http://www.ncbi.nlm.nih.gov/Taxonomy/Browser/wwwtax.cgi?id=427081) ....... 95 [1 hit](http://blast.ncbi.nlm.nih.gov/Blast.cgi?CMD=Get&RID=5Y3VDS5A013&FORMAT_OBJECT=TaxBlast&NCBI_GI=off&DESCRIPTIONS=500&ALIGNMENTS=250&FORMAT_BLOCK_ON_RESPAGE=Top&MASK_COLOR=1&MASK_CHAR=2#427081) [[g-proteobacteria](http://www.ncbi.nlm.nih.gov/Taxonomy/Browser/wwwtax.cgi?id=1236)] [hypothetical protein XOO1407 [Xanthomonas oryzae pv. oryzae](http://www.ncbi.nlm.nih.gov/entrez/query.fcgi?cmd=Retrieve&db=Protein&list_uids=292599419&dopt=GenPept)
235. . [Xanthomonas fuscans subsp. aurantifolii str. ICPB 10535](http://www.ncbi.nlm.nih.gov/Taxonomy/Browser/wwwtax.cgi?id=427082) ....... 95 [1 hit](http://blast.ncbi.nlm.nih.gov/Blast.cgi?CMD=Get&RID=5Y3VDS5A013&FORMAT_OBJECT=TaxBlast&NCBI_GI=off&DESCRIPTIONS=500&ALIGNMENTS=250&FORMAT_BLOCK_ON_RESPAGE=Top&MASK_COLOR=1&MASK_CHAR=2#427082) [[g-proteobacteria](http://www.ncbi.nlm.nih.gov/Taxonomy/Browser/wwwtax.cgi?id=1236)] [hypothetical protein XOO1407 [Xanthomonas oryzae pv. oryzae](http://www.ncbi.nlm.nih.gov/entrez/query.fcgi?cmd=Retrieve&db=Protein&list_uids=292605465&dopt=GenPept)
236. . [Xanthomonas axonopodis pv. punicae str. LMG 859](http://www.ncbi.nlm.nih.gov/Taxonomy/Browser/wwwtax.cgi?id=1085630) ............... 95 [1 hit](http://blast.ncbi.nlm.nih.gov/Blast.cgi?CMD=Get&RID=5Y3VDS5A013&FORMAT_OBJECT=TaxBlast&NCBI_GI=off&DESCRIPTIONS=500&ALIGNMENTS=250&FORMAT_BLOCK_ON_RESPAGE=Top&MASK_COLOR=1&MASK_CHAR=2#1085630) [[g-proteobacteria](http://www.ncbi.nlm.nih.gov/Taxonomy/Browser/wwwtax.cgi?id=1236)] [hypothetical protein XOO1407 [Xanthomonas oryzae pv. oryzae](http://www.ncbi.nlm.nih.gov/entrez/query.fcgi?cmd=Retrieve&db=Protein&list_uids=372555354&dopt=GenPept)
237. . [Xanthomonas citri pv. mangiferaeindicae LMG 941](http://www.ncbi.nlm.nih.gov/Taxonomy/Browser/wwwtax.cgi?id=1156940) ............... 95 [1 hit](http://blast.ncbi.nlm.nih.gov/Blast.cgi?CMD=Get&RID=5Y3VDS5A013&FORMAT_OBJECT=TaxBlast&NCBI_GI=off&DESCRIPTIONS=500&ALIGNMENTS=250&FORMAT_BLOCK_ON_RESPAGE=Top&MASK_COLOR=1&MASK_CHAR=2#1156940) [[g-proteobacteria](http://www.ncbi.nlm.nih.gov/Taxonomy/Browser/wwwtax.cgi?id=1236)] [hypothetical protein XOO1407 [Xanthomonas oryzae pv. oryzae](http://www.ncbi.nlm.nih.gov/entrez/query.fcgi?cmd=Retrieve&db=Protein&list_uids=380687170&dopt=GenPept)
238. . [Xanthomonas fuscans subsp. fuscans](http://www.ncbi.nlm.nih.gov/Taxonomy/Browser/wwwtax.cgi?id=366649) ............................ 95 [1 hit](http://blast.ncbi.nlm.nih.gov/Blast.cgi?CMD=Get&RID=5Y3VDS5A013&FORMAT_OBJECT=TaxBlast&NCBI_GI=off&DESCRIPTIONS=500&ALIGNMENTS=250&FORMAT_BLOCK_ON_RESPAGE=Top&MASK_COLOR=1&MASK_CHAR=2#366649) [[g-proteobacteria](http://www.ncbi.nlm.nih.gov/Taxonomy/Browser/wwwtax.cgi?id=1236)] [hypothetical protein XOO1407 [Xanthomonas oryzae pv. oryzae](http://www.ncbi.nlm.nih.gov/entrez/query.fcgi?cmd=Retrieve&db=Protein&list_uids=549145553&dopt=GenPept)
239. . [Bradyrhizobium sp. DFCI-1](http://www.ncbi.nlm.nih.gov/Taxonomy/Browser/wwwtax.cgi?id=1230476) ..................................... 97 [2 hits](http://blast.ncbi.nlm.nih.gov/Blast.cgi?CMD=Get&RID=5Y3VDS5A013&FORMAT_OBJECT=TaxBlast&NCBI_GI=off&DESCRIPTIONS=500&ALIGNMENTS=250&FORMAT_BLOCK_ON_RESPAGE=Top&MASK_COLOR=1&MASK_CHAR=2#1230476) [[a-proteobacteria](http://www.ncbi.nlm.nih.gov/Taxonomy/Browser/wwwtax.cgi?id=28211)] [hypothetical protein [Bradyrhizobium sp. DFCI-1] >gi|540140](http://www.ncbi.nlm.nih.gov/entrez/query.fcgi?cmd=Retrieve&db=Protein&list_uids=544643567&dopt=GenPept)
240. . [Thiomonas sp. 3As](http://www.ncbi.nlm.nih.gov/Taxonomy/Browser/wwwtax.cgi?id=426114) ............................................. 95 [3 hits](http://blast.ncbi.nlm.nih.gov/Blast.cgi?CMD=Get&RID=5Y3VDS5A013&FORMAT_OBJECT=TaxBlast&NCBI_GI=off&DESCRIPTIONS=500&ALIGNMENTS=250&FORMAT_BLOCK_ON_RESPAGE=Top&MASK_COLOR=1&MASK_CHAR=2#426114) [[b-proteobacteria](http://www.ncbi.nlm.nih.gov/Taxonomy/Browser/wwwtax.cgi?id=28216)] [Putative Histone-lysine N-methyltransferase [Thiomonas sp.](http://www.ncbi.nlm.nih.gov/entrez/query.fcgi?cmd=Retrieve&db=Protein&list_uids=410692691&dopt=GenPept)
241. . [Comamonas testosteroni KF-1](http://www.ncbi.nlm.nih.gov/Taxonomy/Browser/wwwtax.cgi?id=399795) ................................... 95 [1 hit](http://blast.ncbi.nlm.nih.gov/Blast.cgi?CMD=Get&RID=5Y3VDS5A013&FORMAT_OBJECT=TaxBlast&NCBI_GI=off&DESCRIPTIONS=500&ALIGNMENTS=250&FORMAT_BLOCK_ON_RESPAGE=Top&MASK_COLOR=1&MASK_CHAR=2#399795) [[b-proteobacteria](http://www.ncbi.nlm.nih.gov/Taxonomy/Browser/wwwtax.cgi?id=28216)] [Histone-lysine N-methyltransferase [Comamonas testosteroni]](http://www.ncbi.nlm.nih.gov/entrez/query.fcgi?cmd=Retrieve&db=Protein&list_uids=220714784&dopt=GenPept)
242. . [Hyphomicrobium sp. MC1](http://www.ncbi.nlm.nih.gov/Taxonomy/Browser/wwwtax.cgi?id=717785) ........................................ 94 [3 hits](http://blast.ncbi.nlm.nih.gov/Blast.cgi?CMD=Get&RID=5Y3VDS5A013&FORMAT_OBJECT=TaxBlast&NCBI_GI=off&DESCRIPTIONS=500&ALIGNMENTS=250&FORMAT_BLOCK_ON_RESPAGE=Top&MASK_COLOR=1&MASK_CHAR=2#717785) [[a-proteobacteria](http://www.ncbi.nlm.nih.gov/Taxonomy/Browser/wwwtax.cgi?id=28211)] [Nuclear protein SET [Hyphomicrobium sp. MC1] >gi|503713296|](http://www.ncbi.nlm.nih.gov/entrez/query.fcgi?cmd=Retrieve&db=Protein&list_uids=338738577&dopt=GenPept)
243. . [Polaromonas naphthalenivorans CJ2](http://www.ncbi.nlm.nih.gov/Taxonomy/Browser/wwwtax.cgi?id=365044) ............................. 95 [2 hits](http://blast.ncbi.nlm.nih.gov/Blast.cgi?CMD=Get&RID=5Y3VDS5A013&FORMAT_OBJECT=TaxBlast&NCBI_GI=off&DESCRIPTIONS=500&ALIGNMENTS=250&FORMAT_BLOCK_ON_RESPAGE=Top&MASK_COLOR=1&MASK_CHAR=2#365044) [[b-proteobacteria](http://www.ncbi.nlm.nih.gov/Taxonomy/Browser/wwwtax.cgi?id=28216)] [nuclear protein SET [Polaromonas naphthalenivorans CJ2] >gi](http://www.ncbi.nlm.nih.gov/entrez/query.fcgi?cmd=Retrieve&db=Protein&list_uids=121606851&dopt=GenPept)
244. . [Polaromonas naphthalenivorans](http://www.ncbi.nlm.nih.gov/Taxonomy/Browser/wwwtax.cgi?id=216465) ................................. 95 [1 hit](http://blast.ncbi.nlm.nih.gov/Blast.cgi?CMD=Get&RID=5Y3VDS5A013&FORMAT_OBJECT=TaxBlast&NCBI_GI=off&DESCRIPTIONS=500&ALIGNMENTS=250&FORMAT_BLOCK_ON_RESPAGE=Top&MASK_COLOR=1&MASK_CHAR=2#216465) [[b-proteobacteria](http://www.ncbi.nlm.nih.gov/Taxonomy/Browser/wwwtax.cgi?id=28216)] [nuclear protein SET [Polaromonas naphthalenivorans CJ2] >gi](http://www.ncbi.nlm.nih.gov/entrez/query.fcgi?cmd=Retrieve&db=Protein&list_uids=500127320&dopt=GenPept)
245. . [Afipia broomeae](http://www.ncbi.nlm.nih.gov/Taxonomy/Browser/wwwtax.cgi?id=56946) ............................................... 97 [1 hit](http://blast.ncbi.nlm.nih.gov/Blast.cgi?CMD=Get&RID=5Y3VDS5A013&FORMAT_OBJECT=TaxBlast&NCBI_GI=off&DESCRIPTIONS=500&ALIGNMENTS=250&FORMAT_BLOCK_ON_RESPAGE=Top&MASK_COLOR=1&MASK_CHAR=2#56946) [[a-proteobacteria](http://www.ncbi.nlm.nih.gov/Taxonomy/Browser/wwwtax.cgi?id=28211)] [hypothetical protein [Afipia broomeae] >gi|410888466|gb|EKS](http://www.ncbi.nlm.nih.gov/entrez/query.fcgi?cmd=Retrieve&db=Protein&list_uids=492882552&dopt=GenPept)
246. . [Afipia broomeae ATCC 49717](http://www.ncbi.nlm.nih.gov/Taxonomy/Browser/wwwtax.cgi?id=883078) .................................... 97 [1 hit](http://blast.ncbi.nlm.nih.gov/Blast.cgi?CMD=Get&RID=5Y3VDS5A013&FORMAT_OBJECT=TaxBlast&NCBI_GI=off&DESCRIPTIONS=500&ALIGNMENTS=250&FORMAT_BLOCK_ON_RESPAGE=Top&MASK_COLOR=1&MASK_CHAR=2#883078) [[a-proteobacteria](http://www.ncbi.nlm.nih.gov/Taxonomy/Browser/wwwtax.cgi?id=28211)] [hypothetical protein [Afipia broomeae] >gi|410888466|gb|EKS](http://www.ncbi.nlm.nih.gov/entrez/query.fcgi?cmd=Retrieve&db=Protein&list_uids=410888466&dopt=GenPept)
247. . [Dyella ginsengisoli](http://www.ncbi.nlm.nih.gov/Taxonomy/Browser/wwwtax.cgi?id=363848) ........................................... 95 [1 hit](http://blast.ncbi.nlm.nih.gov/Blast.cgi?CMD=Get&RID=5Y3VDS5A013&FORMAT_OBJECT=TaxBlast&NCBI_GI=off&DESCRIPTIONS=500&ALIGNMENTS=250&FORMAT_BLOCK_ON_RESPAGE=Top&MASK_COLOR=1&MASK_CHAR=2#363848) [[g-proteobacteria](http://www.ncbi.nlm.nih.gov/Taxonomy/Browser/wwwtax.cgi?id=1236)] [nuclear protein SET [Dyella ginsengisoli]](http://www.ncbi.nlm.nih.gov/entrez/query.fcgi?cmd=Retrieve&db=Protein&list_uids=516033395&dopt=GenPept)
248. . [Cystobacter fuscus](http://www.ncbi.nlm.nih.gov/Taxonomy/Browser/wwwtax.cgi?id=43) ............................................ 96 [1 hit](http://blast.ncbi.nlm.nih.gov/Blast.cgi?CMD=Get&RID=5Y3VDS5A013&FORMAT_OBJECT=TaxBlast&NCBI_GI=off&DESCRIPTIONS=500&ALIGNMENTS=250&FORMAT_BLOCK_ON_RESPAGE=Top&MASK_COLOR=1&MASK_CHAR=2#43) [[d-proteobacteria](http://www.ncbi.nlm.nih.gov/Taxonomy/Browser/wwwtax.cgi?id=28221)] [SET domain protein [Cystobacter fuscus] >gi|528054740|gb|EP](http://www.ncbi.nlm.nih.gov/entrez/query.fcgi?cmd=Retrieve&db=Protein&list_uids=488701513&dopt=GenPept)
249. . [Cystobacter fuscus DSM 2262](http://www.ncbi.nlm.nih.gov/Taxonomy/Browser/wwwtax.cgi?id=1242864) ................................... 96 [1 hit](http://blast.ncbi.nlm.nih.gov/Blast.cgi?CMD=Get&RID=5Y3VDS5A013&FORMAT_OBJECT=TaxBlast&NCBI_GI=off&DESCRIPTIONS=500&ALIGNMENTS=250&FORMAT_BLOCK_ON_RESPAGE=Top&MASK_COLOR=1&MASK_CHAR=2#1242864) [[d-proteobacteria](http://www.ncbi.nlm.nih.gov/Taxonomy/Browser/wwwtax.cgi?id=28221)] [SET domain protein [Cystobacter fuscus] >gi|528054740|gb|EP](http://www.ncbi.nlm.nih.gov/entrez/query.fcgi?cmd=Retrieve&db=Protein&list_uids=528054740&dopt=GenPept)
250. . [Ralstonia sp. AU12-08](http://www.ncbi.nlm.nih.gov/Taxonomy/Browser/wwwtax.cgi?id=1235457) ......................................... 95 [2 hits](http://blast.ncbi.nlm.nih.gov/Blast.cgi?CMD=Get&RID=5Y3VDS5A013&FORMAT_OBJECT=TaxBlast&NCBI_GI=off&DESCRIPTIONS=500&ALIGNMENTS=250&FORMAT_BLOCK_ON_RESPAGE=Top&MASK_COLOR=1&MASK_CHAR=2#1235457) [[b-proteobacteria](http://www.ncbi.nlm.nih.gov/Taxonomy/Browser/wwwtax.cgi?id=28216)] [nuclear protein SET [Ralstonia sp. AU12-08] >gi|528184535|g](http://www.ncbi.nlm.nih.gov/entrez/query.fcgi?cmd=Retrieve&db=Protein&list_uids=544771840&dopt=GenPept)
251. . [Rhodopseudomonas palustris HaA2](http://www.ncbi.nlm.nih.gov/Taxonomy/Browser/wwwtax.cgi?id=316058) ............................... 97 [2 hits](http://blast.ncbi.nlm.nih.gov/Blast.cgi?CMD=Get&RID=5Y3VDS5A013&FORMAT_OBJECT=TaxBlast&NCBI_GI=off&DESCRIPTIONS=500&ALIGNMENTS=250&FORMAT_BLOCK_ON_RESPAGE=Top&MASK_COLOR=1&MASK_CHAR=2#316058) [[a-proteobacteria](http://www.ncbi.nlm.nih.gov/Taxonomy/Browser/wwwtax.cgi?id=28211)] [nuclear protein SET [Rhodopseudomonas palustris HaA2] >gi|4](http://www.ncbi.nlm.nih.gov/entrez/query.fcgi?cmd=Retrieve&db=Protein&list_uids=86750868&dopt=GenPept)
252. . [Stenotrophomonas maltophilia EPM1](http://www.ncbi.nlm.nih.gov/Taxonomy/Browser/wwwtax.cgi?id=1190567) ............................. 95 [1 hit](http://blast.ncbi.nlm.nih.gov/Blast.cgi?CMD=Get&RID=5Y3VDS5A013&FORMAT_OBJECT=TaxBlast&NCBI_GI=off&DESCRIPTIONS=500&ALIGNMENTS=250&FORMAT_BLOCK_ON_RESPAGE=Top&MASK_COLOR=1&MASK_CHAR=2#1190567) [[g-proteobacteria](http://www.ncbi.nlm.nih.gov/Taxonomy/Browser/wwwtax.cgi?id=1236)] [Hypothetical protein [Stenotrophomonas maltophilia] >gi|456](http://www.ncbi.nlm.nih.gov/entrez/query.fcgi?cmd=Retrieve&db=Protein&list_uids=456735440&dopt=GenPept)
253. . [Hydrogenophaga sp. PBC](http://www.ncbi.nlm.nih.gov/Taxonomy/Browser/wwwtax.cgi?id=795665) ........................................ 95 [2 hits](http://blast.ncbi.nlm.nih.gov/Blast.cgi?CMD=Get&RID=5Y3VDS5A013&FORMAT_OBJECT=TaxBlast&NCBI_GI=off&DESCRIPTIONS=500&ALIGNMENTS=250&FORMAT_BLOCK_ON_RESPAGE=Top&MASK_COLOR=1&MASK_CHAR=2#795665) [[b-proteobacteria](http://www.ncbi.nlm.nih.gov/Taxonomy/Browser/wwwtax.cgi?id=28216)] [nuclear protein SET [Hydrogenophaga sp. PBC] >gi|388262530|](http://www.ncbi.nlm.nih.gov/entrez/query.fcgi?cmd=Retrieve&db=Protein&list_uids=497206444&dopt=GenPept)
254. . [Mesorhizobium sp. WSM4349](http://www.ncbi.nlm.nih.gov/Taxonomy/Browser/wwwtax.cgi?id=1040988) ..................................... 95 [1 hit](http://blast.ncbi.nlm.nih.gov/Blast.cgi?CMD=Get&RID=5Y3VDS5A013&FORMAT_OBJECT=TaxBlast&NCBI_GI=off&DESCRIPTIONS=500&ALIGNMENTS=250&FORMAT_BLOCK_ON_RESPAGE=Top&MASK_COLOR=1&MASK_CHAR=2#1040988) [[a-proteobacteria](http://www.ncbi.nlm.nih.gov/Taxonomy/Browser/wwwtax.cgi?id=28211)] [histone-lysine N-methyltransferase with a SET domain [Mesor](http://www.ncbi.nlm.nih.gov/entrez/query.fcgi?cmd=Retrieve&db=Protein&list_uids=517267579&dopt=GenPept)
255. . [Burkholderia phytofirmans PsJN](http://www.ncbi.nlm.nih.gov/Taxonomy/Browser/wwwtax.cgi?id=398527) ................................ 95 [2 hits](http://blast.ncbi.nlm.nih.gov/Blast.cgi?CMD=Get&RID=5Y3VDS5A013&FORMAT_OBJECT=TaxBlast&NCBI_GI=off&DESCRIPTIONS=500&ALIGNMENTS=250&FORMAT_BLOCK_ON_RESPAGE=Top&MASK_COLOR=1&MASK_CHAR=2#398527) [[b-proteobacteria](http://www.ncbi.nlm.nih.gov/Taxonomy/Browser/wwwtax.cgi?id=28216)] [nuclear protein SET [Burkholderia phytofirmans PsJN] >gi|50](http://www.ncbi.nlm.nih.gov/entrez/query.fcgi?cmd=Retrieve&db=Protein&list_uids=187925895&dopt=GenPept)
256. . [Burkholderia phytofirmans](http://www.ncbi.nlm.nih.gov/Taxonomy/Browser/wwwtax.cgi?id=261302) ..................................... 95 [1 hit](http://blast.ncbi.nlm.nih.gov/Blast.cgi?CMD=Get&RID=5Y3VDS5A013&FORMAT_OBJECT=TaxBlast&NCBI_GI=off&DESCRIPTIONS=500&ALIGNMENTS=250&FORMAT_BLOCK_ON_RESPAGE=Top&MASK_COLOR=1&MASK_CHAR=2#261302) [[b-proteobacteria](http://www.ncbi.nlm.nih.gov/Taxonomy/Browser/wwwtax.cgi?id=28216)] [nuclear protein SET [Burkholderia phytofirmans PsJN] >gi|50](http://www.ncbi.nlm.nih.gov/entrez/query.fcgi?cmd=Retrieve&db=Protein&list_uids=501403263&dopt=GenPept)
257. . [Prosthecochloris aestuarii DSM 271](http://www.ncbi.nlm.nih.gov/Taxonomy/Browser/wwwtax.cgi?id=290512) ............................ 95 [2 hits](http://blast.ncbi.nlm.nih.gov/Blast.cgi?CMD=Get&RID=5Y3VDS5A013&FORMAT_OBJECT=TaxBlast&NCBI_GI=off&DESCRIPTIONS=500&ALIGNMENTS=250&FORMAT_BLOCK_ON_RESPAGE=Top&MASK_COLOR=1&MASK_CHAR=2#290512) [[green sulfur bacteria](http://www.ncbi.nlm.nih.gov/Taxonomy/Browser/wwwtax.cgi?id=1090)] [nuclear protein SET [Prosthecochloris aestuarii DSM 271] >g](http://www.ncbi.nlm.nih.gov/entrez/query.fcgi?cmd=Retrieve&db=Protein&list_uids=194334053&dopt=GenPept)
258. . [Prosthecochloris aestuarii](http://www.ncbi.nlm.nih.gov/Taxonomy/Browser/wwwtax.cgi?id=1102) .................................... 95 [1 hit](http://blast.ncbi.nlm.nih.gov/Blast.cgi?CMD=Get&RID=5Y3VDS5A013&FORMAT_OBJECT=TaxBlast&NCBI_GI=off&DESCRIPTIONS=500&ALIGNMENTS=250&FORMAT_BLOCK_ON_RESPAGE=Top&MASK_COLOR=1&MASK_CHAR=2#1102) [[green sulfur bacteria](http://www.ncbi.nlm.nih.gov/Taxonomy/Browser/wwwtax.cgi?id=1090)] [nuclear protein SET [Prosthecochloris aestuarii DSM 271] >g](http://www.ncbi.nlm.nih.gov/entrez/query.fcgi?cmd=Retrieve&db=Protein&list_uids=501497538&dopt=GenPept)
259. . [Paramecium bursaria Chlorella virus CvsA1](http://www.ncbi.nlm.nih.gov/Taxonomy/Browser/wwwtax.cgi?id=1278254) ..................... 93 [1 hit](http://blast.ncbi.nlm.nih.gov/Blast.cgi?CMD=Get&RID=5Y3VDS5A013&FORMAT_OBJECT=TaxBlast&NCBI_GI=off&DESCRIPTIONS=500&ALIGNMENTS=250&FORMAT_BLOCK_ON_RESPAGE=Top&MASK_COLOR=1&MASK_CHAR=2#1278254) [[viruses](http://www.ncbi.nlm.nih.gov/Taxonomy/Browser/wwwtax.cgi?id=10239)] [SET domain-containing protein [Paramecium bursaria Chlorell](http://www.ncbi.nlm.nih.gov/entrez/query.fcgi?cmd=Retrieve&db=Protein&list_uids=448929100&dopt=GenPept)
260. . [Nitrobacter sp. Nb-311A](http://www.ncbi.nlm.nih.gov/Taxonomy/Browser/wwwtax.cgi?id=314253) ....................................... 95 [2 hits](http://blast.ncbi.nlm.nih.gov/Blast.cgi?CMD=Get&RID=5Y3VDS5A013&FORMAT_OBJECT=TaxBlast&NCBI_GI=off&DESCRIPTIONS=500&ALIGNMENTS=250&FORMAT_BLOCK_ON_RESPAGE=Top&MASK_COLOR=1&MASK_CHAR=2#314253) [[a-proteobacteria](http://www.ncbi.nlm.nih.gov/Taxonomy/Browser/wwwtax.cgi?id=28211)] [histone-lysine N-methyltransferase with a SET domain [Nitro](http://www.ncbi.nlm.nih.gov/entrez/query.fcgi?cmd=Retrieve&db=Protein&list_uids=497485797&dopt=GenPept)
261. . [Acidovorax radicis](http://www.ncbi.nlm.nih.gov/Taxonomy/Browser/wwwtax.cgi?id=758826) ............................................ 95 [1 hit](http://blast.ncbi.nlm.nih.gov/Blast.cgi?CMD=Get&RID=5Y3VDS5A013&FORMAT_OBJECT=TaxBlast&NCBI_GI=off&DESCRIPTIONS=500&ALIGNMENTS=250&FORMAT_BLOCK_ON_RESPAGE=Top&MASK_COLOR=1&MASK_CHAR=2#758826) [[b-proteobacteria](http://www.ncbi.nlm.nih.gov/Taxonomy/Browser/wwwtax.cgi?id=28216)] [lysine methyltransferase [Acidovorax radicis]](http://www.ncbi.nlm.nih.gov/entrez/query.fcgi?cmd=Retrieve&db=Protein&list_uids=498150325&dopt=GenPept)
262. . [Comamonas testosteroni CNB-2](http://www.ncbi.nlm.nih.gov/Taxonomy/Browser/wwwtax.cgi?id=688245) .................................. 95 [2 hits](http://blast.ncbi.nlm.nih.gov/Blast.cgi?CMD=Get&RID=5Y3VDS5A013&FORMAT_OBJECT=TaxBlast&NCBI_GI=off&DESCRIPTIONS=500&ALIGNMENTS=250&FORMAT_BLOCK_ON_RESPAGE=Top&MASK_COLOR=1&MASK_CHAR=2#688245) [[b-proteobacteria](http://www.ncbi.nlm.nih.gov/Taxonomy/Browser/wwwtax.cgi?id=28216)] [nuclear protein SET [Comamonas testosteroni CNB-2] >gi|4891](http://www.ncbi.nlm.nih.gov/entrez/query.fcgi?cmd=Retrieve&db=Protein&list_uids=264680920&dopt=GenPept)
263. . [Comamonas testosteroni S44](http://www.ncbi.nlm.nih.gov/Taxonomy/Browser/wwwtax.cgi?id=563045) .................................... 95 [1 hit](http://blast.ncbi.nlm.nih.gov/Blast.cgi?CMD=Get&RID=5Y3VDS5A013&FORMAT_OBJECT=TaxBlast&NCBI_GI=off&DESCRIPTIONS=500&ALIGNMENTS=250&FORMAT_BLOCK_ON_RESPAGE=Top&MASK_COLOR=1&MASK_CHAR=2#563045) [[b-proteobacteria](http://www.ncbi.nlm.nih.gov/Taxonomy/Browser/wwwtax.cgi?id=28216)] [nuclear protein SET [Comamonas testosteroni CNB-2] >gi|4891](http://www.ncbi.nlm.nih.gov/entrez/query.fcgi?cmd=Retrieve&db=Protein&list_uids=298721133&dopt=GenPept)
264. . [Delftia acidovorans SPH-1](http://www.ncbi.nlm.nih.gov/Taxonomy/Browser/wwwtax.cgi?id=398578) ..................................... 96 [2 hits](http://blast.ncbi.nlm.nih.gov/Blast.cgi?CMD=Get&RID=5Y3VDS5A013&FORMAT_OBJECT=TaxBlast&NCBI_GI=off&DESCRIPTIONS=500&ALIGNMENTS=250&FORMAT_BLOCK_ON_RESPAGE=Top&MASK_COLOR=1&MASK_CHAR=2#398578) [[b-proteobacteria](http://www.ncbi.nlm.nih.gov/Taxonomy/Browser/wwwtax.cgi?id=28216)] [nuclear protein SET [Delftia acidovorans SPH-1] >gi|5011583](http://www.ncbi.nlm.nih.gov/entrez/query.fcgi?cmd=Retrieve&db=Protein&list_uids=160896332&dopt=GenPept)
265. . [Advenella kashmirensis WT001](http://www.ncbi.nlm.nih.gov/Taxonomy/Browser/wwwtax.cgi?id=1036672) .................................. 95 [2 hits](http://blast.ncbi.nlm.nih.gov/Blast.cgi?CMD=Get&RID=5Y3VDS5A013&FORMAT_OBJECT=TaxBlast&NCBI_GI=off&DESCRIPTIONS=500&ALIGNMENTS=250&FORMAT_BLOCK_ON_RESPAGE=Top&MASK_COLOR=1&MASK_CHAR=2#1036672) [[b-proteobacteria](http://www.ncbi.nlm.nih.gov/Taxonomy/Browser/wwwtax.cgi?id=28216)] [hypothetical protein TKWG_01965 [Advenella kashmirensis WT0](http://www.ncbi.nlm.nih.gov/entrez/query.fcgi?cmd=Retrieve&db=Protein&list_uids=389870606&dopt=GenPept)
266. . [Advenella kashmirensis](http://www.ncbi.nlm.nih.gov/Taxonomy/Browser/wwwtax.cgi?id=310575) ........................................ 95 [1 hit](http://blast.ncbi.nlm.nih.gov/Blast.cgi?CMD=Get&RID=5Y3VDS5A013&FORMAT_OBJECT=TaxBlast&NCBI_GI=off&DESCRIPTIONS=500&ALIGNMENTS=250&FORMAT_BLOCK_ON_RESPAGE=Top&MASK_COLOR=1&MASK_CHAR=2#310575) [[b-proteobacteria](http://www.ncbi.nlm.nih.gov/Taxonomy/Browser/wwwtax.cgi?id=28216)] [hypothetical protein TKWG_01965 [Advenella kashmirensis WT0](http://www.ncbi.nlm.nih.gov/entrez/query.fcgi?cmd=Retrieve&db=Protein&list_uids=504562032&dopt=GenPept)
267. . [Burkholderia graminis](http://www.ncbi.nlm.nih.gov/Taxonomy/Browser/wwwtax.cgi?id=60548) ......................................... 95 [1 hit](http://blast.ncbi.nlm.nih.gov/Blast.cgi?CMD=Get&RID=5Y3VDS5A013&FORMAT_OBJECT=TaxBlast&NCBI_GI=off&DESCRIPTIONS=500&ALIGNMENTS=250&FORMAT_BLOCK_ON_RESPAGE=Top&MASK_COLOR=1&MASK_CHAR=2#60548) [[b-proteobacteria](http://www.ncbi.nlm.nih.gov/Taxonomy/Browser/wwwtax.cgi?id=28216)] [nuclear protein SET [Burkholderia graminis] >gi|170140712|g](http://www.ncbi.nlm.nih.gov/entrez/query.fcgi?cmd=Retrieve&db=Protein&list_uids=492933564&dopt=GenPept)
268. . [Burkholderia graminis C4D1M](http://www.ncbi.nlm.nih.gov/Taxonomy/Browser/wwwtax.cgi?id=396598) ................................... 95 [1 hit](http://blast.ncbi.nlm.nih.gov/Blast.cgi?CMD=Get&RID=5Y3VDS5A013&FORMAT_OBJECT=TaxBlast&NCBI_GI=off&DESCRIPTIONS=500&ALIGNMENTS=250&FORMAT_BLOCK_ON_RESPAGE=Top&MASK_COLOR=1&MASK_CHAR=2#396598) [[b-proteobacteria](http://www.ncbi.nlm.nih.gov/Taxonomy/Browser/wwwtax.cgi?id=28216)] [nuclear protein SET [Burkholderia graminis] >gi|170140712|g](http://www.ncbi.nlm.nih.gov/entrez/query.fcgi?cmd=Retrieve&db=Protein&list_uids=170140712&dopt=GenPept)
269. . [Bradyrhizobium sp. WSM1253](http://www.ncbi.nlm.nih.gov/Taxonomy/Browser/wwwtax.cgi?id=319003) .................................... 95 [2 hits](http://blast.ncbi.nlm.nih.gov/Blast.cgi?CMD=Get&RID=5Y3VDS5A013&FORMAT_OBJECT=TaxBlast&NCBI_GI=off&DESCRIPTIONS=500&ALIGNMENTS=250&FORMAT_BLOCK_ON_RESPAGE=Top&MASK_COLOR=1&MASK_CHAR=2#319003) [[a-proteobacteria](http://www.ncbi.nlm.nih.gov/Taxonomy/Browser/wwwtax.cgi?id=28211)] [histone-lysine N-methyltransferase with a SET domain [Brady](http://www.ncbi.nlm.nih.gov/entrez/query.fcgi?cmd=Retrieve&db=Protein&list_uids=494876094&dopt=GenPept)
270. . [Bradyrhizobium sp. CCGE-LA001](http://www.ncbi.nlm.nih.gov/Taxonomy/Browser/wwwtax.cgi?id=1223566) ................................. 95 [2 hits](http://blast.ncbi.nlm.nih.gov/Blast.cgi?CMD=Get&RID=5Y3VDS5A013&FORMAT_OBJECT=TaxBlast&NCBI_GI=off&DESCRIPTIONS=500&ALIGNMENTS=250&FORMAT_BLOCK_ON_RESPAGE=Top&MASK_COLOR=1&MASK_CHAR=2#1223566) [[a-proteobacteria](http://www.ncbi.nlm.nih.gov/Taxonomy/Browser/wwwtax.cgi?id=28211)] [hypothetical protein [Bradyrhizobium sp. CCGE-LA001] >gi|40](http://www.ncbi.nlm.nih.gov/entrez/query.fcgi?cmd=Retrieve&db=Protein&list_uids=495839437&dopt=GenPept)
271. . [Oligella urethralis](http://www.ncbi.nlm.nih.gov/Taxonomy/Browser/wwwtax.cgi?id=90245) ........................................... 95 [1 hit](http://blast.ncbi.nlm.nih.gov/Blast.cgi?CMD=Get&RID=5Y3VDS5A013&FORMAT_OBJECT=TaxBlast&NCBI_GI=off&DESCRIPTIONS=500&ALIGNMENTS=250&FORMAT_BLOCK_ON_RESPAGE=Top&MASK_COLOR=1&MASK_CHAR=2#90245) [[b-proteobacteria](http://www.ncbi.nlm.nih.gov/Taxonomy/Browser/wwwtax.cgi?id=28216)] [hypothetical protein [Oligella urethralis]](http://www.ncbi.nlm.nih.gov/entrez/query.fcgi?cmd=Retrieve&db=Protein&list_uids=516659503&dopt=GenPept)
272. . [Acidovorax delafieldii](http://www.ncbi.nlm.nih.gov/Taxonomy/Browser/wwwtax.cgi?id=47920) ........................................ 95 [1 hit](http://blast.ncbi.nlm.nih.gov/Blast.cgi?CMD=Get&RID=5Y3VDS5A013&FORMAT_OBJECT=TaxBlast&NCBI_GI=off&DESCRIPTIONS=500&ALIGNMENTS=250&FORMAT_BLOCK_ON_RESPAGE=Top&MASK_COLOR=1&MASK_CHAR=2#47920) [[b-proteobacteria](http://www.ncbi.nlm.nih.gov/Taxonomy/Browser/wwwtax.cgi?id=28216)] [lysine methyltransferase [Acidovorax delafieldii] >gi|24136](http://www.ncbi.nlm.nih.gov/entrez/query.fcgi?cmd=Retrieve&db=Protein&list_uids=492279195&dopt=GenPept)
273. . [Acidovorax delafieldii 2AN](http://www.ncbi.nlm.nih.gov/Taxonomy/Browser/wwwtax.cgi?id=573060) .................................... 95 [1 hit](http://blast.ncbi.nlm.nih.gov/Blast.cgi?CMD=Get&RID=5Y3VDS5A013&FORMAT_OBJECT=TaxBlast&NCBI_GI=off&DESCRIPTIONS=500&ALIGNMENTS=250&FORMAT_BLOCK_ON_RESPAGE=Top&MASK_COLOR=1&MASK_CHAR=2#573060) [[b-proteobacteria](http://www.ncbi.nlm.nih.gov/Taxonomy/Browser/wwwtax.cgi?id=28216)] [lysine methyltransferase [Acidovorax delafieldii] >gi|24136](http://www.ncbi.nlm.nih.gov/entrez/query.fcgi?cmd=Retrieve&db=Protein&list_uids=241364916&dopt=GenPept)
274. . [Limnohabitans sp. Rim28](http://www.ncbi.nlm.nih.gov/Taxonomy/Browser/wwwtax.cgi?id=1100720) ....................................... 96 [1 hit](http://blast.ncbi.nlm.nih.gov/Blast.cgi?CMD=Get&RID=5Y3VDS5A013&FORMAT_OBJECT=TaxBlast&NCBI_GI=off&DESCRIPTIONS=500&ALIGNMENTS=250&FORMAT_BLOCK_ON_RESPAGE=Top&MASK_COLOR=1&MASK_CHAR=2#1100720) [[b-proteobacteria](http://www.ncbi.nlm.nih.gov/Taxonomy/Browser/wwwtax.cgi?id=28216)] [hypothetical protein [Limnohabitans sp. Rim28]](http://www.ncbi.nlm.nih.gov/entrez/query.fcgi?cmd=Retrieve&db=Protein&list_uids=518257190&dopt=GenPept)
275. . [Afipia sp. 1NLS2](http://www.ncbi.nlm.nih.gov/Taxonomy/Browser/wwwtax.cgi?id=666684) .............................................. 95 [2 hits](http://blast.ncbi.nlm.nih.gov/Blast.cgi?CMD=Get&RID=5Y3VDS5A013&FORMAT_OBJECT=TaxBlast&NCBI_GI=off&DESCRIPTIONS=500&ALIGNMENTS=250&FORMAT_BLOCK_ON_RESPAGE=Top&MASK_COLOR=1&MASK_CHAR=2#666684) [[a-proteobacteria](http://www.ncbi.nlm.nih.gov/Taxonomy/Browser/wwwtax.cgi?id=28211)] [methyltransferase [Afipia sp. 1NLS2] >gi|298591892|gb|EFI52](http://www.ncbi.nlm.nih.gov/entrez/query.fcgi?cmd=Retrieve&db=Protein&list_uids=496695394&dopt=GenPept)
276. . [Xanthomonas oryzae](http://www.ncbi.nlm.nih.gov/Taxonomy/Browser/wwwtax.cgi?id=347) ............................................ 94 [2 hits](http://blast.ncbi.nlm.nih.gov/Blast.cgi?CMD=Get&RID=5Y3VDS5A013&FORMAT_OBJECT=TaxBlast&NCBI_GI=off&DESCRIPTIONS=500&ALIGNMENTS=250&FORMAT_BLOCK_ON_RESPAGE=Top&MASK_COLOR=1&MASK_CHAR=2#347) [[g-proteobacteria](http://www.ncbi.nlm.nih.gov/Taxonomy/Browser/wwwtax.cgi?id=1236)] [nuclear protein SET [Xanthomonas oryzae]](http://www.ncbi.nlm.nih.gov/entrez/query.fcgi?cmd=Retrieve&db=Protein&list_uids=518134518&dopt=GenPept)
277. . [Bradyrhizobiaceae bacterium SG-6C](http://www.ncbi.nlm.nih.gov/Taxonomy/Browser/wwwtax.cgi?id=709797) ............................. 95 [2 hits](http://blast.ncbi.nlm.nih.gov/Blast.cgi?CMD=Get&RID=5Y3VDS5A013&FORMAT_OBJECT=TaxBlast&NCBI_GI=off&DESCRIPTIONS=500&ALIGNMENTS=250&FORMAT_BLOCK_ON_RESPAGE=Top&MASK_COLOR=1&MASK_CHAR=2#709797) [[a-proteobacteria](http://www.ncbi.nlm.nih.gov/Taxonomy/Browser/wwwtax.cgi?id=28211)] [methyltransferase [Bradyrhizobiaceae bacterium SG-6C] >gi|3](http://www.ncbi.nlm.nih.gov/entrez/query.fcgi?cmd=Retrieve&db=Protein&list_uids=497422733&dopt=GenPept)
278. . [Bordetella sp. FB-8](http://www.ncbi.nlm.nih.gov/Taxonomy/Browser/wwwtax.cgi?id=1159870) ........................................... 94 [1 hit](http://blast.ncbi.nlm.nih.gov/Blast.cgi?CMD=Get&RID=5Y3VDS5A013&FORMAT_OBJECT=TaxBlast&NCBI_GI=off&DESCRIPTIONS=500&ALIGNMENTS=250&FORMAT_BLOCK_ON_RESPAGE=Top&MASK_COLOR=1&MASK_CHAR=2#1159870) [[b-proteobacteria](http://www.ncbi.nlm.nih.gov/Taxonomy/Browser/wwwtax.cgi?id=28216)] [SET domain-containing protein [Bordetella sp. FB-8]](http://www.ncbi.nlm.nih.gov/entrez/query.fcgi?cmd=Retrieve&db=Protein&list_uids=518780172&dopt=GenPept)
279. . [Burkholderia sp. Ch1-1](http://www.ncbi.nlm.nih.gov/Taxonomy/Browser/wwwtax.cgi?id=243261) ........................................ 94 [2 hits](http://blast.ncbi.nlm.nih.gov/Blast.cgi?CMD=Get&RID=5Y3VDS5A013&FORMAT_OBJECT=TaxBlast&NCBI_GI=off&DESCRIPTIONS=500&ALIGNMENTS=250&FORMAT_BLOCK_ON_RESPAGE=Top&MASK_COLOR=1&MASK_CHAR=2#243261) [[b-proteobacteria](http://www.ncbi.nlm.nih.gov/Taxonomy/Browser/wwwtax.cgi?id=28216)] [SET domain-containing protein [Burkholderia sp. Ch1-1] >gi|](http://www.ncbi.nlm.nih.gov/entrez/query.fcgi?cmd=Retrieve&db=Protein&list_uids=494323498&dopt=GenPept)
280. . [Burkholderia bryophila](http://www.ncbi.nlm.nih.gov/Taxonomy/Browser/wwwtax.cgi?id=420952) ........................................ 95 [1 hit](http://blast.ncbi.nlm.nih.gov/Blast.cgi?CMD=Get&RID=5Y3VDS5A013&FORMAT_OBJECT=TaxBlast&NCBI_GI=off&DESCRIPTIONS=500&ALIGNMENTS=250&FORMAT_BLOCK_ON_RESPAGE=Top&MASK_COLOR=1&MASK_CHAR=2#420952) [[b-proteobacteria](http://www.ncbi.nlm.nih.gov/Taxonomy/Browser/wwwtax.cgi?id=28216)] [nuclear protein SET [Burkholderia bryophila]](http://www.ncbi.nlm.nih.gov/entrez/query.fcgi?cmd=Retrieve&db=Protein&list_uids=518911524&dopt=GenPept)
281. . [Burkholderia sp. SJ98](http://www.ncbi.nlm.nih.gov/Taxonomy/Browser/wwwtax.cgi?id=406819) ......................................... 93 [2 hits](http://blast.ncbi.nlm.nih.gov/Blast.cgi?CMD=Get&RID=5Y3VDS5A013&FORMAT_OBJECT=TaxBlast&NCBI_GI=off&DESCRIPTIONS=500&ALIGNMENTS=250&FORMAT_BLOCK_ON_RESPAGE=Top&MASK_COLOR=1&MASK_CHAR=2#406819) [[b-proteobacteria](http://www.ncbi.nlm.nih.gov/Taxonomy/Browser/wwwtax.cgi?id=28216)] [nuclear protein [Burkholderia sp. SJ98] >gi|413939749|gb|EK](http://www.ncbi.nlm.nih.gov/entrez/query.fcgi?cmd=Retrieve&db=Protein&list_uids=495620167&dopt=GenPept)
282. . [Pseudomonas geniculata](http://www.ncbi.nlm.nih.gov/Taxonomy/Browser/wwwtax.cgi?id=86188) ........................................ 93 [1 hit](http://blast.ncbi.nlm.nih.gov/Blast.cgi?CMD=Get&RID=5Y3VDS5A013&FORMAT_OBJECT=TaxBlast&NCBI_GI=off&DESCRIPTIONS=500&ALIGNMENTS=250&FORMAT_BLOCK_ON_RESPAGE=Top&MASK_COLOR=1&MASK_CHAR=2#86188) [[g-proteobacteria](http://www.ncbi.nlm.nih.gov/Taxonomy/Browser/wwwtax.cgi?id=1236)] [nuclear protein SET [Pseudomonas geniculata]](http://www.ncbi.nlm.nih.gov/entrez/query.fcgi?cmd=Retrieve&db=Protein&list_uids=498169393&dopt=GenPept)
283. . [Chlorobium phaeovibrioides DSM 265](http://www.ncbi.nlm.nih.gov/Taxonomy/Browser/wwwtax.cgi?id=290318) ............................ 93 [2 hits](http://blast.ncbi.nlm.nih.gov/Blast.cgi?CMD=Get&RID=5Y3VDS5A013&FORMAT_OBJECT=TaxBlast&NCBI_GI=off&DESCRIPTIONS=500&ALIGNMENTS=250&FORMAT_BLOCK_ON_RESPAGE=Top&MASK_COLOR=1&MASK_CHAR=2#290318) [[green sulfur bacteria](http://www.ncbi.nlm.nih.gov/Taxonomy/Browser/wwwtax.cgi?id=1090)] [nuclear protein SET [Chlorobium phaeovibrioides DSM 265] >g](http://www.ncbi.nlm.nih.gov/entrez/query.fcgi?cmd=Retrieve&db=Protein&list_uids=145219635&dopt=GenPept)
284. . [Chlorobium phaeovibrioides](http://www.ncbi.nlm.nih.gov/Taxonomy/Browser/wwwtax.cgi?id=1094) .................................... 93 [1 hit](http://blast.ncbi.nlm.nih.gov/Blast.cgi?CMD=Get&RID=5Y3VDS5A013&FORMAT_OBJECT=TaxBlast&NCBI_GI=off&DESCRIPTIONS=500&ALIGNMENTS=250&FORMAT_BLOCK_ON_RESPAGE=Top&MASK_COLOR=1&MASK_CHAR=2#1094) [[green sulfur bacteria](http://www.ncbi.nlm.nih.gov/Taxonomy/Browser/wwwtax.cgi?id=1090)] [nuclear protein SET [Chlorobium phaeovibrioides DSM 265] >g](http://www.ncbi.nlm.nih.gov/entrez/query.fcgi?cmd=Retrieve&db=Protein&list_uids=500219943&dopt=GenPept)
285. . [Stenotrophomonas maltophilia AU12-09](http://www.ncbi.nlm.nih.gov/Taxonomy/Browser/wwwtax.cgi?id=1235458) .......................... 93 [1 hit](http://blast.ncbi.nlm.nih.gov/Blast.cgi?CMD=Get&RID=5Y3VDS5A013&FORMAT_OBJECT=TaxBlast&NCBI_GI=off&DESCRIPTIONS=500&ALIGNMENTS=250&FORMAT_BLOCK_ON_RESPAGE=Top&MASK_COLOR=1&MASK_CHAR=2#1235458) [[g-proteobacteria](http://www.ncbi.nlm.nih.gov/Taxonomy/Browser/wwwtax.cgi?id=1236)] [protein containing SET domain [Stenotrophomonas maltophilia](http://www.ncbi.nlm.nih.gov/entrez/query.fcgi?cmd=Retrieve&db=Protein&list_uids=460869483&dopt=GenPept)
286. . [Stenotrophomonas maltophilia MF89](http://www.ncbi.nlm.nih.gov/Taxonomy/Browser/wwwtax.cgi?id=1333853) ............................. 94 [1 hit](http://blast.ncbi.nlm.nih.gov/Blast.cgi?CMD=Get&RID=5Y3VDS5A013&FORMAT_OBJECT=TaxBlast&NCBI_GI=off&DESCRIPTIONS=500&ALIGNMENTS=250&FORMAT_BLOCK_ON_RESPAGE=Top&MASK_COLOR=1&MASK_CHAR=2#1333853) [[g-proteobacteria](http://www.ncbi.nlm.nih.gov/Taxonomy/Browser/wwwtax.cgi?id=1236)] [nuclear protein SET [Stenotrophomonas maltophilia] >gi|5332](http://www.ncbi.nlm.nih.gov/entrez/query.fcgi?cmd=Retrieve&db=Protein&list_uids=533226935&dopt=GenPept)
287. . [Stenotrophomonas maltophilia R551-3](http://www.ncbi.nlm.nih.gov/Taxonomy/Browser/wwwtax.cgi?id=391008) ........................... 93 [2 hits](http://blast.ncbi.nlm.nih.gov/Blast.cgi?CMD=Get&RID=5Y3VDS5A013&FORMAT_OBJECT=TaxBlast&NCBI_GI=off&DESCRIPTIONS=500&ALIGNMENTS=250&FORMAT_BLOCK_ON_RESPAGE=Top&MASK_COLOR=1&MASK_CHAR=2#391008) [[g-proteobacteria](http://www.ncbi.nlm.nih.gov/Taxonomy/Browser/wwwtax.cgi?id=1236)] [nuclear protein SET [Stenotrophomonas maltophilia R551-3] >](http://www.ncbi.nlm.nih.gov/entrez/query.fcgi?cmd=Retrieve&db=Protein&list_uids=194364781&dopt=GenPept)
288. . [Polaromonas sp. JS666](http://www.ncbi.nlm.nih.gov/Taxonomy/Browser/wwwtax.cgi?id=296591) ......................................... 95 [3 hits](http://blast.ncbi.nlm.nih.gov/Blast.cgi?CMD=Get&RID=5Y3VDS5A013&FORMAT_OBJECT=TaxBlast&NCBI_GI=off&DESCRIPTIONS=500&ALIGNMENTS=250&FORMAT_BLOCK_ON_RESPAGE=Top&MASK_COLOR=1&MASK_CHAR=2#296591) [[b-proteobacteria](http://www.ncbi.nlm.nih.gov/Taxonomy/Browser/wwwtax.cgi?id=28216)] [nuclear protein SET [Polaromonas sp. JS666] >gi|499804846|r](http://www.ncbi.nlm.nih.gov/entrez/query.fcgi?cmd=Retrieve&db=Protein&list_uids=91790541&dopt=GenPept)
289. . [Stenotrophomonas maltophilia RA8](http://www.ncbi.nlm.nih.gov/Taxonomy/Browser/wwwtax.cgi?id=1118157) .............................. 93 [1 hit](http://blast.ncbi.nlm.nih.gov/Blast.cgi?CMD=Get&RID=5Y3VDS5A013&FORMAT_OBJECT=TaxBlast&NCBI_GI=off&DESCRIPTIONS=500&ALIGNMENTS=250&FORMAT_BLOCK_ON_RESPAGE=Top&MASK_COLOR=1&MASK_CHAR=2#1118157) [[g-proteobacteria](http://www.ncbi.nlm.nih.gov/Taxonomy/Browser/wwwtax.cgi?id=1236)] [Histone-lysine N-methyltransferase SETD1B [Stenotrophomonas](http://www.ncbi.nlm.nih.gov/entrez/query.fcgi?cmd=Retrieve&db=Protein&list_uids=475058468&dopt=GenPept)
290. . [Bradyrhizobium diazoefficiens USDA 110](http://www.ncbi.nlm.nih.gov/Taxonomy/Browser/wwwtax.cgi?id=224911) ........................ 94 [2 hits](http://blast.ncbi.nlm.nih.gov/Blast.cgi?CMD=Get&RID=5Y3VDS5A013&FORMAT_OBJECT=TaxBlast&NCBI_GI=off&DESCRIPTIONS=500&ALIGNMENTS=250&FORMAT_BLOCK_ON_RESPAGE=Top&MASK_COLOR=1&MASK_CHAR=2#224911) [[a-proteobacteria](http://www.ncbi.nlm.nih.gov/Taxonomy/Browser/wwwtax.cgi?id=28211)] [hypothetical protein bll5787 [Bradyrhizobium diazoefficiens](http://www.ncbi.nlm.nih.gov/entrez/query.fcgi?cmd=Retrieve&db=Protein&list_uids=27380898&dopt=GenPept)
291. . [Bradyrhizobium diazoefficiens](http://www.ncbi.nlm.nih.gov/Taxonomy/Browser/wwwtax.cgi?id=1355477) ................................. 94 [1 hit](http://blast.ncbi.nlm.nih.gov/Blast.cgi?CMD=Get&RID=5Y3VDS5A013&FORMAT_OBJECT=TaxBlast&NCBI_GI=off&DESCRIPTIONS=500&ALIGNMENTS=250&FORMAT_BLOCK_ON_RESPAGE=Top&MASK_COLOR=1&MASK_CHAR=2#1355477) [[a-proteobacteria](http://www.ncbi.nlm.nih.gov/Taxonomy/Browser/wwwtax.cgi?id=28211)] [hypothetical protein bll5787 [Bradyrhizobium diazoefficiens](http://www.ncbi.nlm.nih.gov/entrez/query.fcgi?cmd=Retrieve&db=Protein&list_uids=499401064&dopt=GenPept)
292. . [Rubrivivax gelatinosus IL144](http://www.ncbi.nlm.nih.gov/Taxonomy/Browser/wwwtax.cgi?id=983917) .................................. 93 [2 hits](http://blast.ncbi.nlm.nih.gov/Blast.cgi?CMD=Get&RID=5Y3VDS5A013&FORMAT_OBJECT=TaxBlast&NCBI_GI=off&DESCRIPTIONS=500&ALIGNMENTS=250&FORMAT_BLOCK_ON_RESPAGE=Top&MASK_COLOR=1&MASK_CHAR=2#983917) [[b-proteobacteria](http://www.ncbi.nlm.nih.gov/Taxonomy/Browser/wwwtax.cgi?id=28216)] [SET domain-containing protein [Rubrivivax gelatinosus IL144](http://www.ncbi.nlm.nih.gov/entrez/query.fcgi?cmd=Retrieve&db=Protein&list_uids=383760322&dopt=GenPept)
293. . [Rubrivivax gelatinosus](http://www.ncbi.nlm.nih.gov/Taxonomy/Browser/wwwtax.cgi?id=28068) ........................................ 93 [1 hit](http://blast.ncbi.nlm.nih.gov/Blast.cgi?CMD=Get&RID=5Y3VDS5A013&FORMAT_OBJECT=TaxBlast&NCBI_GI=off&DESCRIPTIONS=500&ALIGNMENTS=250&FORMAT_BLOCK_ON_RESPAGE=Top&MASK_COLOR=1&MASK_CHAR=2#28068) [[b-proteobacteria](http://www.ncbi.nlm.nih.gov/Taxonomy/Browser/wwwtax.cgi?id=28216)] [SET domain-containing protein [Rubrivivax gelatinosus IL144](http://www.ncbi.nlm.nih.gov/entrez/query.fcgi?cmd=Retrieve&db=Protein&list_uids=504243555&dopt=GenPept)
294. . [Bradyrhizobium sp. YR681](http://www.ncbi.nlm.nih.gov/Taxonomy/Browser/wwwtax.cgi?id=1144344) ...................................... 94 [2 hits](http://blast.ncbi.nlm.nih.gov/Blast.cgi?CMD=Get&RID=5Y3VDS5A013&FORMAT_OBJECT=TaxBlast&NCBI_GI=off&DESCRIPTIONS=500&ALIGNMENTS=250&FORMAT_BLOCK_ON_RESPAGE=Top&MASK_COLOR=1&MASK_CHAR=2#1144344) [[a-proteobacteria](http://www.ncbi.nlm.nih.gov/Taxonomy/Browser/wwwtax.cgi?id=28211)] [histone-lysine N-methyltransferase with a SET domain [Brady](http://www.ncbi.nlm.nih.gov/entrez/query.fcgi?cmd=Retrieve&db=Protein&list_uids=495415065&dopt=GenPept)
295. . [Acidovorax sp. KKS102](http://www.ncbi.nlm.nih.gov/Taxonomy/Browser/wwwtax.cgi?id=358220) ......................................... 94 [3 hits](http://blast.ncbi.nlm.nih.gov/Blast.cgi?CMD=Get&RID=5Y3VDS5A013&FORMAT_OBJECT=TaxBlast&NCBI_GI=off&DESCRIPTIONS=500&ALIGNMENTS=250&FORMAT_BLOCK_ON_RESPAGE=Top&MASK_COLOR=1&MASK_CHAR=2#358220) [[b-proteobacteria](http://www.ncbi.nlm.nih.gov/Taxonomy/Browser/wwwtax.cgi?id=28216)] [nuclear protein SET [Acidovorax sp. KKS102] >gi|504829207|r](http://www.ncbi.nlm.nih.gov/entrez/query.fcgi?cmd=Retrieve&db=Protein&list_uids=407941364&dopt=GenPept)
296. . [Afipia clevelandensis](http://www.ncbi.nlm.nih.gov/Taxonomy/Browser/wwwtax.cgi?id=1034) ......................................... 95 [1 hit](http://blast.ncbi.nlm.nih.gov/Blast.cgi?CMD=Get&RID=5Y3VDS5A013&FORMAT_OBJECT=TaxBlast&NCBI_GI=off&DESCRIPTIONS=500&ALIGNMENTS=250&FORMAT_BLOCK_ON_RESPAGE=Top&MASK_COLOR=1&MASK_CHAR=2#1034) [[a-proteobacteria](http://www.ncbi.nlm.nih.gov/Taxonomy/Browser/wwwtax.cgi?id=28211)] [hypothetical protein [Afipia clevelandensis] >gi|410889896|](http://www.ncbi.nlm.nih.gov/entrez/query.fcgi?cmd=Retrieve&db=Protein&list_uids=488800107&dopt=GenPept)
297. . [Afipia clevelandensis ATCC 49720](http://www.ncbi.nlm.nih.gov/Taxonomy/Browser/wwwtax.cgi?id=883079) .............................. 95 [1 hit](http://blast.ncbi.nlm.nih.gov/Blast.cgi?CMD=Get&RID=5Y3VDS5A013&FORMAT_OBJECT=TaxBlast&NCBI_GI=off&DESCRIPTIONS=500&ALIGNMENTS=250&FORMAT_BLOCK_ON_RESPAGE=Top&MASK_COLOR=1&MASK_CHAR=2#883079) [[a-proteobacteria](http://www.ncbi.nlm.nih.gov/Taxonomy/Browser/wwwtax.cgi?id=28211)] [hypothetical protein [Afipia clevelandensis] >gi|410889896|](http://www.ncbi.nlm.nih.gov/entrez/query.fcgi?cmd=Retrieve&db=Protein&list_uids=410889896&dopt=GenPept)
298. . [Bordetella avium 197N](http://www.ncbi.nlm.nih.gov/Taxonomy/Browser/wwwtax.cgi?id=360910) ......................................... 93 [2 hits](http://blast.ncbi.nlm.nih.gov/Blast.cgi?CMD=Get&RID=5Y3VDS5A013&FORMAT_OBJECT=TaxBlast&NCBI_GI=off&DESCRIPTIONS=500&ALIGNMENTS=250&FORMAT_BLOCK_ON_RESPAGE=Top&MASK_COLOR=1&MASK_CHAR=2#360910) [[b-proteobacteria](http://www.ncbi.nlm.nih.gov/Taxonomy/Browser/wwwtax.cgi?id=28216)] [hypothetical protein BAV3390 [Bordetella avium 197N] >gi|50](http://www.ncbi.nlm.nih.gov/entrez/query.fcgi?cmd=Retrieve&db=Protein&list_uids=187479859&dopt=GenPept)
299. . [Bordetella avium](http://www.ncbi.nlm.nih.gov/Taxonomy/Browser/wwwtax.cgi?id=521) .............................................. 93 [1 hit](http://blast.ncbi.nlm.nih.gov/Blast.cgi?CMD=Get&RID=5Y3VDS5A013&FORMAT_OBJECT=TaxBlast&NCBI_GI=off&DESCRIPTIONS=500&ALIGNMENTS=250&FORMAT_BLOCK_ON_RESPAGE=Top&MASK_COLOR=1&MASK_CHAR=2#521) [[b-proteobacteria](http://www.ncbi.nlm.nih.gov/Taxonomy/Browser/wwwtax.cgi?id=28216)] [hypothetical protein BAV3390 [Bordetella avium 197N] >gi|50](http://www.ncbi.nlm.nih.gov/entrez/query.fcgi?cmd=Retrieve&db=Protein&list_uids=501387460&dopt=GenPept)
300. . [Xanthomonas sacchari](http://www.ncbi.nlm.nih.gov/Taxonomy/Browser/wwwtax.cgi?id=56458) .......................................... 93 [1 hit](http://blast.ncbi.nlm.nih.gov/Blast.cgi?CMD=Get&RID=5Y3VDS5A013&FORMAT_OBJECT=TaxBlast&NCBI_GI=off&DESCRIPTIONS=500&ALIGNMENTS=250&FORMAT_BLOCK_ON_RESPAGE=Top&MASK_COLOR=1&MASK_CHAR=2#56458) [[g-proteobacteria](http://www.ncbi.nlm.nih.gov/Taxonomy/Browser/wwwtax.cgi?id=1236)] [hypothetical protein [Xanthomonas sacchari]](http://www.ncbi.nlm.nih.gov/entrez/query.fcgi?cmd=Retrieve&db=Protein&list_uids=498027855&dopt=GenPept)
301. . [Rhodopseudomonas palustris DX-1](http://www.ncbi.nlm.nih.gov/Taxonomy/Browser/wwwtax.cgi?id=652103) ............................... 95 [2 hits](http://blast.ncbi.nlm.nih.gov/Blast.cgi?CMD=Get&RID=5Y3VDS5A013&FORMAT_OBJECT=TaxBlast&NCBI_GI=off&DESCRIPTIONS=500&ALIGNMENTS=250&FORMAT_BLOCK_ON_RESPAGE=Top&MASK_COLOR=1&MASK_CHAR=2#652103) [[a-proteobacteria](http://www.ncbi.nlm.nih.gov/Taxonomy/Browser/wwwtax.cgi?id=28211)] [nuclear protein SET [Rhodopseudomonas palustris DX-1] >gi|5](http://www.ncbi.nlm.nih.gov/entrez/query.fcgi?cmd=Retrieve&db=Protein&list_uids=316932855&dopt=GenPept)
302. . [Acidovorax ebreus TPSY](http://www.ncbi.nlm.nih.gov/Taxonomy/Browser/wwwtax.cgi?id=535289) ........................................ 94 [2 hits](http://blast.ncbi.nlm.nih.gov/Blast.cgi?CMD=Get&RID=5Y3VDS5A013&FORMAT_OBJECT=TaxBlast&NCBI_GI=off&DESCRIPTIONS=500&ALIGNMENTS=250&FORMAT_BLOCK_ON_RESPAGE=Top&MASK_COLOR=1&MASK_CHAR=2#535289) [[b-proteobacteria](http://www.ncbi.nlm.nih.gov/Taxonomy/Browser/wwwtax.cgi?id=28216)] [nuclear protein set [Acidovorax ebreus TPSY] >gi|506394846|](http://www.ncbi.nlm.nih.gov/entrez/query.fcgi?cmd=Retrieve&db=Protein&list_uids=222112504&dopt=GenPept)
303. . [Acidovorax ebreus](http://www.ncbi.nlm.nih.gov/Taxonomy/Browser/wwwtax.cgi?id=721785) ............................................. 94 [1 hit](http://blast.ncbi.nlm.nih.gov/Blast.cgi?CMD=Get&RID=5Y3VDS5A013&FORMAT_OBJECT=TaxBlast&NCBI_GI=off&DESCRIPTIONS=500&ALIGNMENTS=250&FORMAT_BLOCK_ON_RESPAGE=Top&MASK_COLOR=1&MASK_CHAR=2#721785) [[b-proteobacteria](http://www.ncbi.nlm.nih.gov/Taxonomy/Browser/wwwtax.cgi?id=28216)] [nuclear protein set [Acidovorax ebreus TPSY] >gi|506394846|](http://www.ncbi.nlm.nih.gov/entrez/query.fcgi?cmd=Retrieve&db=Protein&list_uids=506394846&dopt=GenPept)
304. . [Afipia felis](http://www.ncbi.nlm.nih.gov/Taxonomy/Browser/wwwtax.cgi?id=1035) .................................................. 95 [1 hit](http://blast.ncbi.nlm.nih.gov/Blast.cgi?CMD=Get&RID=5Y3VDS5A013&FORMAT_OBJECT=TaxBlast&NCBI_GI=off&DESCRIPTIONS=500&ALIGNMENTS=250&FORMAT_BLOCK_ON_RESPAGE=Top&MASK_COLOR=1&MASK_CHAR=2#1035) [[a-proteobacteria](http://www.ncbi.nlm.nih.gov/Taxonomy/Browser/wwwtax.cgi?id=28211)] [hypothetical protein [Afipia felis] >gi|410879749|gb|EKS275](http://www.ncbi.nlm.nih.gov/entrez/query.fcgi?cmd=Retrieve&db=Protein&list_uids=488802786&dopt=GenPept)
305. . [Afipia felis ATCC 53690](http://www.ncbi.nlm.nih.gov/Taxonomy/Browser/wwwtax.cgi?id=883080) ....................................... 95 [1 hit](http://blast.ncbi.nlm.nih.gov/Blast.cgi?CMD=Get&RID=5Y3VDS5A013&FORMAT_OBJECT=TaxBlast&NCBI_GI=off&DESCRIPTIONS=500&ALIGNMENTS=250&FORMAT_BLOCK_ON_RESPAGE=Top&MASK_COLOR=1&MASK_CHAR=2#883080) [[a-proteobacteria](http://www.ncbi.nlm.nih.gov/Taxonomy/Browser/wwwtax.cgi?id=28211)] [hypothetical protein [Afipia felis] >gi|410879749|gb|EKS275](http://www.ncbi.nlm.nih.gov/entrez/query.fcgi?cmd=Retrieve&db=Protein&list_uids=410879749&dopt=GenPept)
306. . [Turneriella parva DSM 21527](http://www.ncbi.nlm.nih.gov/Taxonomy/Browser/wwwtax.cgi?id=869212) ................................... 93 [2 hits](http://blast.ncbi.nlm.nih.gov/Blast.cgi?CMD=Get&RID=5Y3VDS5A013&FORMAT_OBJECT=TaxBlast&NCBI_GI=off&DESCRIPTIONS=500&ALIGNMENTS=250&FORMAT_BLOCK_ON_RESPAGE=Top&MASK_COLOR=1&MASK_CHAR=2#869212) [[spirochetes](http://www.ncbi.nlm.nih.gov/Taxonomy/Browser/wwwtax.cgi?id=136)] [nuclear protein SET [Turneriella parva DSM 21527] >gi|50461](http://www.ncbi.nlm.nih.gov/entrez/query.fcgi?cmd=Retrieve&db=Protein&list_uids=392404635&dopt=GenPept)
307. . [Turneriella parva](http://www.ncbi.nlm.nih.gov/Taxonomy/Browser/wwwtax.cgi?id=29510) ............................................. 93 [1 hit](http://blast.ncbi.nlm.nih.gov/Blast.cgi?CMD=Get&RID=5Y3VDS5A013&FORMAT_OBJECT=TaxBlast&NCBI_GI=off&DESCRIPTIONS=500&ALIGNMENTS=250&FORMAT_BLOCK_ON_RESPAGE=Top&MASK_COLOR=1&MASK_CHAR=2#29510) [[spirochetes](http://www.ncbi.nlm.nih.gov/Taxonomy/Browser/wwwtax.cgi?id=136)] [nuclear protein SET [Turneriella parva DSM 21527] >gi|50461](http://www.ncbi.nlm.nih.gov/entrez/query.fcgi?cmd=Retrieve&db=Protein&list_uids=504617139&dopt=GenPept)
308. . [Leptospira kirschneri](http://www.ncbi.nlm.nih.gov/Taxonomy/Browser/wwwtax.cgi?id=29507) ............................................. 101 [5 hits](http://blast.ncbi.nlm.nih.gov/Blast.cgi?CMD=Get&RID=DAWEZ5AS01R&FORMAT_OBJECT=TaxBlast&NCBI_GI=off&DESCRIPTIONS=100&ALIGNMENTS=100&FORMAT_BLOCK_ON_RESPAGE=Top&MASK_COLOR=1&MASK_CHAR=2#29507) [[spirochetes](http://www.ncbi.nlm.nih.gov/Taxonomy/Browser/wwwtax.cgi?id=136)] [SET family protein [Leptospira kirschneri]](http://www.ncbi.nlm.nih.gov/entrez/query.fcgi?cmd=Retrieve&db=Protein&list_uids=515121640&dopt=GenPept)
309. . [Leptospira kirschneri serovar Grippotyphosa str. RM52](http://www.ncbi.nlm.nih.gov/Taxonomy/Browser/wwwtax.cgi?id=1049946) ............. 100 [1 hit](http://blast.ncbi.nlm.nih.gov/Blast.cgi?CMD=Get&RID=DAWEZ5AS01R&FORMAT_OBJECT=TaxBlast&NCBI_GI=off&DESCRIPTIONS=100&ALIGNMENTS=100&FORMAT_BLOCK_ON_RESPAGE=Top&MASK_COLOR=1&MASK_CHAR=2#1049946) [[spirochetes](http://www.ncbi.nlm.nih.gov/Taxonomy/Browser/wwwtax.cgi?id=136)] [SET domain protein [Leptospira kirschneri] >gi|400322330|gb](http://www.ncbi.nlm.nih.gov/entrez/query.fcgi?cmd=Retrieve&db=Protein&list_uids=400322330&dopt=GenPept)
310. . [Leptospira kirschneri str. 2008720114](http://www.ncbi.nlm.nih.gov/Taxonomy/Browser/wwwtax.cgi?id=1193049) ............................. 100 [1 hit](http://blast.ncbi.nlm.nih.gov/Blast.cgi?CMD=Get&RID=DAWEZ5AS01R&FORMAT_OBJECT=TaxBlast&NCBI_GI=off&DESCRIPTIONS=100&ALIGNMENTS=100&FORMAT_BLOCK_ON_RESPAGE=Top&MASK_COLOR=1&MASK_CHAR=2#1193049) [[spirochetes](http://www.ncbi.nlm.nih.gov/Taxonomy/Browser/wwwtax.cgi?id=136)] [SET domain protein [Leptospira kirschneri] >gi|400322330|gb](http://www.ncbi.nlm.nih.gov/entrez/query.fcgi?cmd=Retrieve&db=Protein&list_uids=410357168&dopt=GenPept)
311. . [Leptospira kirschneri str. MMD1493](http://www.ncbi.nlm.nih.gov/Taxonomy/Browser/wwwtax.cgi?id=1218583) ................................ 100 [1 hit](http://blast.ncbi.nlm.nih.gov/Blast.cgi?CMD=Get&RID=DAWEZ5AS01R&FORMAT_OBJECT=TaxBlast&NCBI_GI=off&DESCRIPTIONS=100&ALIGNMENTS=100&FORMAT_BLOCK_ON_RESPAGE=Top&MASK_COLOR=1&MASK_CHAR=2#1218583) [[spirochetes](http://www.ncbi.nlm.nih.gov/Taxonomy/Browser/wwwtax.cgi?id=136)] [SET domain protein [Leptospira kirschneri] >gi|400322330|gb](http://www.ncbi.nlm.nih.gov/entrez/query.fcgi?cmd=Retrieve&db=Protein&list_uids=463342307&dopt=GenPept)
312. . [Acanthamoeba castellanii str. Neff](http://www.ncbi.nlm.nih.gov/Taxonomy/Browser/wwwtax.cgi?id=1257118) ................................ 101 [4 hits](http://blast.ncbi.nlm.nih.gov/Blast.cgi?CMD=Get&RID=DAWEZ5AS01R&FORMAT_OBJECT=TaxBlast&NCBI_GI=off&DESCRIPTIONS=100&ALIGNMENTS=100&FORMAT_BLOCK_ON_RESPAGE=Top&MASK_COLOR=1&MASK_CHAR=2#1257118) [[eukaryotes](http://www.ncbi.nlm.nih.gov/Taxonomy/Browser/wwwtax.cgi?id=2759)] [SET family protein [Acanthamoeba castellanii str. Neff] >gi](http://www.ncbi.nlm.nih.gov/entrez/query.fcgi?cmd=Retrieve&db=Protein&list_uids=470520656&dopt=GenPept)
313. . [Leptospira](http://www.ncbi.nlm.nih.gov/Taxonomy/Browser/wwwtax.cgi?id=171) ........................................................ 100 [6 hits](http://blast.ncbi.nlm.nih.gov/Blast.cgi?CMD=Get&RID=DAWEZ5AS01R&FORMAT_OBJECT=TaxBlast&NCBI_GI=off&DESCRIPTIONS=100&ALIGNMENTS=100&FORMAT_BLOCK_ON_RESPAGE=Top&MASK_COLOR=1&MASK_CHAR=2#171) [[spirochetes](http://www.ncbi.nlm.nih.gov/Taxonomy/Browser/wwwtax.cgi?id=136)] [SET domain protein [Leptospira] >gi|410784583|gb|EKR73559.1](http://www.ncbi.nlm.nih.gov/entrez/query.fcgi?cmd=Retrieve&db=Protein&list_uids=488107665&dopt=GenPept)
314. . [Leptospira noguchii str. 2006001870](http://www.ncbi.nlm.nih.gov/Taxonomy/Browser/wwwtax.cgi?id=1001593) ............................... 100 [1 hit](http://blast.ncbi.nlm.nih.gov/Blast.cgi?CMD=Get&RID=DAWEZ5AS01R&FORMAT_OBJECT=TaxBlast&NCBI_GI=off&DESCRIPTIONS=100&ALIGNMENTS=100&FORMAT_BLOCK_ON_RESPAGE=Top&MASK_COLOR=1&MASK_CHAR=2#1001593) [[spirochetes](http://www.ncbi.nlm.nih.gov/Taxonomy/Browser/wwwtax.cgi?id=136)] [SET domain protein [Leptospira] >gi|410784583|gb|EKR73559.1](http://www.ncbi.nlm.nih.gov/entrez/query.fcgi?cmd=Retrieve&db=Protein&list_uids=410784583&dopt=GenPept)
315. . [Leptospira noguchii str. Bonito](http://www.ncbi.nlm.nih.gov/Taxonomy/Browser/wwwtax.cgi?id=1218586) ................................... 100 [1 hit](http://blast.ncbi.nlm.nih.gov/Blast.cgi?CMD=Get&RID=DAWEZ5AS01R&FORMAT_OBJECT=TaxBlast&NCBI_GI=off&DESCRIPTIONS=100&ALIGNMENTS=100&FORMAT_BLOCK_ON_RESPAGE=Top&MASK_COLOR=1&MASK_CHAR=2#1218586) [[spirochetes](http://www.ncbi.nlm.nih.gov/Taxonomy/Browser/wwwtax.cgi?id=136)] [SET domain protein [Leptospira] >gi|410784583|gb|EKR73559.1](http://www.ncbi.nlm.nih.gov/entrez/query.fcgi?cmd=Retrieve&db=Protein&list_uids=461486128&dopt=GenPept)
316. . [Leptospira noguchii str. 2007001578](http://www.ncbi.nlm.nih.gov/Taxonomy/Browser/wwwtax.cgi?id=1049974) ............................... 100 [1 hit](http://blast.ncbi.nlm.nih.gov/Blast.cgi?CMD=Get&RID=DAWEZ5AS01R&FORMAT_OBJECT=TaxBlast&NCBI_GI=off&DESCRIPTIONS=100&ALIGNMENTS=100&FORMAT_BLOCK_ON_RESPAGE=Top&MASK_COLOR=1&MASK_CHAR=2#1049974) [[spirochetes](http://www.ncbi.nlm.nih.gov/Taxonomy/Browser/wwwtax.cgi?id=136)] [SET domain protein [Leptospira] >gi|410784583|gb|EKR73559.1](http://www.ncbi.nlm.nih.gov/entrez/query.fcgi?cmd=Retrieve&db=Protein&list_uids=464180615&dopt=GenPept)
317. . [Leptospira noguchii serovar Autumnalis str. ZUN142](http://www.ncbi.nlm.nih.gov/Taxonomy/Browser/wwwtax.cgi?id=1085540) ................ 100 [1 hit](http://blast.ncbi.nlm.nih.gov/Blast.cgi?CMD=Get&RID=DAWEZ5AS01R&FORMAT_OBJECT=TaxBlast&NCBI_GI=off&DESCRIPTIONS=100&ALIGNMENTS=100&FORMAT_BLOCK_ON_RESPAGE=Top&MASK_COLOR=1&MASK_CHAR=2#1085540) [[spirochetes](http://www.ncbi.nlm.nih.gov/Taxonomy/Browser/wwwtax.cgi?id=136)] [SET domain protein [Leptospira] >gi|410784583|gb|EKR73559.1](http://www.ncbi.nlm.nih.gov/entrez/query.fcgi?cmd=Retrieve&db=Protein&list_uids=464365309&dopt=GenPept)
318. . [Leptospira interrogans str. HAI1536](http://www.ncbi.nlm.nih.gov/Taxonomy/Browser/wwwtax.cgi?id=1193041) ............................... 100 [1 hit](http://blast.ncbi.nlm.nih.gov/Blast.cgi?CMD=Get&RID=DAWEZ5AS01R&FORMAT_OBJECT=TaxBlast&NCBI_GI=off&DESCRIPTIONS=100&ALIGNMENTS=100&FORMAT_BLOCK_ON_RESPAGE=Top&MASK_COLOR=1&MASK_CHAR=2#1193041) [[spirochetes](http://www.ncbi.nlm.nih.gov/Taxonomy/Browser/wwwtax.cgi?id=136)] [SET domain protein [Leptospira] >gi|410784583|gb|EKR73559.1](http://www.ncbi.nlm.nih.gov/entrez/query.fcgi?cmd=Retrieve&db=Protein&list_uids=464381420&dopt=GenPept)
319. . [Leptospira noguchii str. 2001034031](http://www.ncbi.nlm.nih.gov/Taxonomy/Browser/wwwtax.cgi?id=1193053) ............................... 100 [1 hit](http://blast.ncbi.nlm.nih.gov/Blast.cgi?CMD=Get&RID=DAWEZ5AS01R&FORMAT_OBJECT=TaxBlast&NCBI_GI=off&DESCRIPTIONS=100&ALIGNMENTS=100&FORMAT_BLOCK_ON_RESPAGE=Top&MASK_COLOR=1&MASK_CHAR=2#1193053) [[spirochetes](http://www.ncbi.nlm.nih.gov/Taxonomy/Browser/wwwtax.cgi?id=136)] [SET domain protein [Leptospira] >gi|410784583|gb|EKR73559.1](http://www.ncbi.nlm.nih.gov/entrez/query.fcgi?cmd=Retrieve&db=Protein&list_uids=464432625&dopt=GenPept)
320. . [Leptospira noguchii str. Cascata](http://www.ncbi.nlm.nih.gov/Taxonomy/Browser/wwwtax.cgi?id=1193054) .................................. 100 [1 hit](http://blast.ncbi.nlm.nih.gov/Blast.cgi?CMD=Get&RID=DAWEZ5AS01R&FORMAT_OBJECT=TaxBlast&NCBI_GI=off&DESCRIPTIONS=100&ALIGNMENTS=100&FORMAT_BLOCK_ON_RESPAGE=Top&MASK_COLOR=1&MASK_CHAR=2#1193054) [[spirochetes](http://www.ncbi.nlm.nih.gov/Taxonomy/Browser/wwwtax.cgi?id=136)] [SET domain protein [Leptospira] >gi|410784583|gb|EKR73559.1](http://www.ncbi.nlm.nih.gov/entrez/query.fcgi?cmd=Retrieve&db=Protein&list_uids=474497952&dopt=GenPept)
321. . [Leptospira noguchii str. Hook](http://www.ncbi.nlm.nih.gov/Taxonomy/Browser/wwwtax.cgi?id=1193055) ..................................... 100 [1 hit](http://blast.ncbi.nlm.nih.gov/Blast.cgi?CMD=Get&RID=DAWEZ5AS01R&FORMAT_OBJECT=TaxBlast&NCBI_GI=off&DESCRIPTIONS=100&ALIGNMENTS=100&FORMAT_BLOCK_ON_RESPAGE=Top&MASK_COLOR=1&MASK_CHAR=2#1193055) [[spirochetes](http://www.ncbi.nlm.nih.gov/Taxonomy/Browser/wwwtax.cgi?id=136)] [SET domain protein [Leptospira] >gi|410784583|gb|EKR73559.1](http://www.ncbi.nlm.nih.gov/entrez/query.fcgi?cmd=Retrieve&db=Protein&list_uids=474498031&dopt=GenPept)
322. . [Leptospira noguchii str. 1993005606](http://www.ncbi.nlm.nih.gov/Taxonomy/Browser/wwwtax.cgi?id=1049973) ............................... 100 [1 hit](http://blast.ncbi.nlm.nih.gov/Blast.cgi?CMD=Get&RID=DAWEZ5AS01R&FORMAT_OBJECT=TaxBlast&NCBI_GI=off&DESCRIPTIONS=100&ALIGNMENTS=100&FORMAT_BLOCK_ON_RESPAGE=Top&MASK_COLOR=1&MASK_CHAR=2#1049973) [[spirochetes](http://www.ncbi.nlm.nih.gov/Taxonomy/Browser/wwwtax.cgi?id=136)] [SET domain protein [Leptospira] >gi|410784583|gb|EKR73559.1](http://www.ncbi.nlm.nih.gov/entrez/query.fcgi?cmd=Retrieve&db=Protein&list_uids=512768075&dopt=GenPept)
323. . [Leptospira noguchii serovar Panama str. CZ214](http://www.ncbi.nlm.nih.gov/Taxonomy/Browser/wwwtax.cgi?id=1001595) ..................... 100 [1 hit](http://blast.ncbi.nlm.nih.gov/Blast.cgi?CMD=Get&RID=DAWEZ5AS01R&FORMAT_OBJECT=TaxBlast&NCBI_GI=off&DESCRIPTIONS=100&ALIGNMENTS=100&FORMAT_BLOCK_ON_RESPAGE=Top&MASK_COLOR=1&MASK_CHAR=2#1001595) [[spirochetes](http://www.ncbi.nlm.nih.gov/Taxonomy/Browser/wwwtax.cgi?id=136)] [SET domain protein [Leptospira] >gi|410784583|gb|EKR73559.1](http://www.ncbi.nlm.nih.gov/entrez/query.fcgi?cmd=Retrieve&db=Protein&list_uids=529332369&dopt=GenPept)
324. . [Kribbella flavida DSM 17836](http://www.ncbi.nlm.nih.gov/Taxonomy/Browser/wwwtax.cgi?id=479435) ....................................... 102 [2 hits](http://blast.ncbi.nlm.nih.gov/Blast.cgi?CMD=Get&RID=DAWEZ5AS01R&FORMAT_OBJECT=TaxBlast&NCBI_GI=off&DESCRIPTIONS=100&ALIGNMENTS=100&FORMAT_BLOCK_ON_RESPAGE=Top&MASK_COLOR=1&MASK_CHAR=2#479435) [[high GC Gram+](http://www.ncbi.nlm.nih.gov/Taxonomy/Browser/wwwtax.cgi?id=1760)] [nuclear protein SET [Kribbella flavida DSM 17836] >gi|50268](http://www.ncbi.nlm.nih.gov/entrez/query.fcgi?cmd=Retrieve&db=Protein&list_uids=284028389&dopt=GenPept)
325. . [Kribbella flavida](http://www.ncbi.nlm.nih.gov/Taxonomy/Browser/wwwtax.cgi?id=182640) ................................................. 102 [1 hit](http://blast.ncbi.nlm.nih.gov/Blast.cgi?CMD=Get&RID=DAWEZ5AS01R&FORMAT_OBJECT=TaxBlast&NCBI_GI=off&DESCRIPTIONS=100&ALIGNMENTS=100&FORMAT_BLOCK_ON_RESPAGE=Top&MASK_COLOR=1&MASK_CHAR=2#182640) [[high GC Gram+](http://www.ncbi.nlm.nih.gov/Taxonomy/Browser/wwwtax.cgi?id=1760)] [nuclear protein SET [Kribbella flavida DSM 17836] >gi|50268](http://www.ncbi.nlm.nih.gov/entrez/query.fcgi?cmd=Retrieve&db=Protein&list_uids=502682463&dopt=GenPept)
326. . [Leptospira weilii str. 2006001853](http://www.ncbi.nlm.nih.gov/Taxonomy/Browser/wwwtax.cgi?id=1001589) ................................. 100 [1 hit](http://blast.ncbi.nlm.nih.gov/Blast.cgi?CMD=Get&RID=DAWEZ5AS01R&FORMAT_OBJECT=TaxBlast&NCBI_GI=off&DESCRIPTIONS=100&ALIGNMENTS=100&FORMAT_BLOCK_ON_RESPAGE=Top&MASK_COLOR=1&MASK_CHAR=2#1001589) [[spirochetes](http://www.ncbi.nlm.nih.gov/Taxonomy/Browser/wwwtax.cgi?id=136)] [SET domain protein [Leptospira] >gi|410780074|gb|EKR64676.1](http://www.ncbi.nlm.nih.gov/entrez/query.fcgi?cmd=Retrieve&db=Protein&list_uids=410780074&dopt=GenPept)
327. . [Leptospira sp. P2653](http://www.ncbi.nlm.nih.gov/Taxonomy/Browser/wwwtax.cgi?id=1218600) .............................................. 100 [1 hit](http://blast.ncbi.nlm.nih.gov/Blast.cgi?CMD=Get&RID=DAWEZ5AS01R&FORMAT_OBJECT=TaxBlast&NCBI_GI=off&DESCRIPTIONS=100&ALIGNMENTS=100&FORMAT_BLOCK_ON_RESPAGE=Top&MASK_COLOR=1&MASK_CHAR=2#1218600) [[spirochetes](http://www.ncbi.nlm.nih.gov/Taxonomy/Browser/wwwtax.cgi?id=136)] [SET domain protein [Leptospira] >gi|410780074|gb|EKR64676.1](http://www.ncbi.nlm.nih.gov/entrez/query.fcgi?cmd=Retrieve&db=Protein&list_uids=463298601&dopt=GenPept)
328. . [Leptospira weilii str. 2006001855](http://www.ncbi.nlm.nih.gov/Taxonomy/Browser/wwwtax.cgi?id=996804) ................................. 100 [1 hit](http://blast.ncbi.nlm.nih.gov/Blast.cgi?CMD=Get&RID=DAWEZ5AS01R&FORMAT_OBJECT=TaxBlast&NCBI_GI=off&DESCRIPTIONS=100&ALIGNMENTS=100&FORMAT_BLOCK_ON_RESPAGE=Top&MASK_COLOR=1&MASK_CHAR=2#996804) [[spirochetes](http://www.ncbi.nlm.nih.gov/Taxonomy/Browser/wwwtax.cgi?id=136)] [SET domain protein [Leptospira] >gi|410780074|gb|EKR64676.1](http://www.ncbi.nlm.nih.gov/entrez/query.fcgi?cmd=Retrieve&db=Protein&list_uids=464135572&dopt=GenPept)
329. . [Leptospira weilii str. LNT 1234](http://www.ncbi.nlm.nih.gov/Taxonomy/Browser/wwwtax.cgi?id=1088541) ................................... 100 [1 hit](http://blast.ncbi.nlm.nih.gov/Blast.cgi?CMD=Get&RID=DAWEZ5AS01R&FORMAT_OBJECT=TaxBlast&NCBI_GI=off&DESCRIPTIONS=100&ALIGNMENTS=100&FORMAT_BLOCK_ON_RESPAGE=Top&MASK_COLOR=1&MASK_CHAR=2#1088541) [[spirochetes](http://www.ncbi.nlm.nih.gov/Taxonomy/Browser/wwwtax.cgi?id=136)] [SET domain protein [Leptospira] >gi|410780074|gb|EKR64676.1](http://www.ncbi.nlm.nih.gov/entrez/query.fcgi?cmd=Retrieve&db=Protein&list_uids=464236251&dopt=GenPept)
330. . [Leptospira weilii str. Ecochallenge](http://www.ncbi.nlm.nih.gov/Taxonomy/Browser/wwwtax.cgi?id=1049986) ............................... 100 [1 hit](http://blast.ncbi.nlm.nih.gov/Blast.cgi?CMD=Get&RID=DAWEZ5AS01R&FORMAT_OBJECT=TaxBlast&NCBI_GI=off&DESCRIPTIONS=100&ALIGNMENTS=100&FORMAT_BLOCK_ON_RESPAGE=Top&MASK_COLOR=1&MASK_CHAR=2#1049986) [[spirochetes](http://www.ncbi.nlm.nih.gov/Taxonomy/Browser/wwwtax.cgi?id=136)] [SET domain protein [Leptospira] >gi|410780074|gb|EKR64676.1](http://www.ncbi.nlm.nih.gov/entrez/query.fcgi?cmd=Retrieve&db=Protein&list_uids=476389346&dopt=GenPept)
331. . [Leptospira weilii](http://www.ncbi.nlm.nih.gov/Taxonomy/Browser/wwwtax.cgi?id=28184) ................................................. 100 [2 hits](http://blast.ncbi.nlm.nih.gov/Blast.cgi?CMD=Get&RID=DAWEZ5AS01R&FORMAT_OBJECT=TaxBlast&NCBI_GI=off&DESCRIPTIONS=100&ALIGNMENTS=100&FORMAT_BLOCK_ON_RESPAGE=Top&MASK_COLOR=1&MASK_CHAR=2#28184) [[spirochetes](http://www.ncbi.nlm.nih.gov/Taxonomy/Browser/wwwtax.cgi?id=136)] [SET domain protein [Leptospira weilii] >gi|456865018|gb|EMF](http://www.ncbi.nlm.nih.gov/entrez/query.fcgi?cmd=Retrieve&db=Protein&list_uids=490640372&dopt=GenPept)
332. . [Leptospira weilii serovar Topaz str. LT2116](http://www.ncbi.nlm.nih.gov/Taxonomy/Browser/wwwtax.cgi?id=1088540) ....................... 100 [1 hit](http://blast.ncbi.nlm.nih.gov/Blast.cgi?CMD=Get&RID=DAWEZ5AS01R&FORMAT_OBJECT=TaxBlast&NCBI_GI=off&DESCRIPTIONS=100&ALIGNMENTS=100&FORMAT_BLOCK_ON_RESPAGE=Top&MASK_COLOR=1&MASK_CHAR=2#1088540) [[spirochetes](http://www.ncbi.nlm.nih.gov/Taxonomy/Browser/wwwtax.cgi?id=136)] [SET domain protein [Leptospira weilii] >gi|456865018|gb|EMF](http://www.ncbi.nlm.nih.gov/entrez/query.fcgi?cmd=Retrieve&db=Protein&list_uids=456865018&dopt=GenPept)
333. . [Leptospira kirschneri str. H2](http://www.ncbi.nlm.nih.gov/Taxonomy/Browser/wwwtax.cgi?id=1193050) ..................................... 99 [1 hit](http://blast.ncbi.nlm.nih.gov/Blast.cgi?CMD=Get&RID=DAWEZ5AS01R&FORMAT_OBJECT=TaxBlast&NCBI_GI=off&DESCRIPTIONS=100&ALIGNMENTS=100&FORMAT_BLOCK_ON_RESPAGE=Top&MASK_COLOR=1&MASK_CHAR=2#1193050) [[spirochetes](http://www.ncbi.nlm.nih.gov/Taxonomy/Browser/wwwtax.cgi?id=136)] [SET domain protein [Leptospira] >gi|410007170|gb|EKO60882.1](http://www.ncbi.nlm.nih.gov/entrez/query.fcgi?cmd=Retrieve&db=Protein&list_uids=410007170&dopt=GenPept)
334. . [Leptospira kirschneri str. JB](http://www.ncbi.nlm.nih.gov/Taxonomy/Browser/wwwtax.cgi?id=1218584) ..................................... 99 [1 hit](http://blast.ncbi.nlm.nih.gov/Blast.cgi?CMD=Get&RID=DAWEZ5AS01R&FORMAT_OBJECT=TaxBlast&NCBI_GI=off&DESCRIPTIONS=100&ALIGNMENTS=100&FORMAT_BLOCK_ON_RESPAGE=Top&MASK_COLOR=1&MASK_CHAR=2#1218584) [[spirochetes](http://www.ncbi.nlm.nih.gov/Taxonomy/Browser/wwwtax.cgi?id=136)] [SET domain protein [Leptospira] >gi|410007170|gb|EKO60882.1](http://www.ncbi.nlm.nih.gov/entrez/query.fcgi?cmd=Retrieve&db=Protein&list_uids=463326345&dopt=GenPept)
335. . [Leptospira santarosai str. CBC613](http://www.ncbi.nlm.nih.gov/Taxonomy/Browser/wwwtax.cgi?id=1218595) ................................. 99 [1 hit](http://blast.ncbi.nlm.nih.gov/Blast.cgi?CMD=Get&RID=DAWEZ5AS01R&FORMAT_OBJECT=TaxBlast&NCBI_GI=off&DESCRIPTIONS=100&ALIGNMENTS=100&FORMAT_BLOCK_ON_RESPAGE=Top&MASK_COLOR=1&MASK_CHAR=2#1218595) [[spirochetes](http://www.ncbi.nlm.nih.gov/Taxonomy/Browser/wwwtax.cgi?id=136)] [SET domain protein [Leptospira] >gi|410007170|gb|EKO60882.1](http://www.ncbi.nlm.nih.gov/entrez/query.fcgi?cmd=Retrieve&db=Protein&list_uids=463347608&dopt=GenPept)
336. . [Leptospira kirschneri serovar Bulgarica str. Nikolaevo](http://www.ncbi.nlm.nih.gov/Taxonomy/Browser/wwwtax.cgi?id=1240687) ............ 99 [1 hit](http://blast.ncbi.nlm.nih.gov/Blast.cgi?CMD=Get&RID=DAWEZ5AS01R&FORMAT_OBJECT=TaxBlast&NCBI_GI=off&DESCRIPTIONS=100&ALIGNMENTS=100&FORMAT_BLOCK_ON_RESPAGE=Top&MASK_COLOR=1&MASK_CHAR=2#1240687) [[spirochetes](http://www.ncbi.nlm.nih.gov/Taxonomy/Browser/wwwtax.cgi?id=136)] [SET domain protein [Leptospira] >gi|410007170|gb|EKO60882.1](http://www.ncbi.nlm.nih.gov/entrez/query.fcgi?cmd=Retrieve&db=Protein&list_uids=463358530&dopt=GenPept)
337. . [Leptospira interrogans](http://www.ncbi.nlm.nih.gov/Taxonomy/Browser/wwwtax.cgi?id=173) ............................................ 100 [9 hits](http://blast.ncbi.nlm.nih.gov/Blast.cgi?CMD=Get&RID=DAWEZ5AS01R&FORMAT_OBJECT=TaxBlast&NCBI_GI=off&DESCRIPTIONS=100&ALIGNMENTS=100&FORMAT_BLOCK_ON_RESPAGE=Top&MASK_COLOR=1&MASK_CHAR=2#173) [[spirochetes](http://www.ncbi.nlm.nih.gov/Taxonomy/Browser/wwwtax.cgi?id=136)] [SET family protein [Leptospira interrogans]](http://www.ncbi.nlm.nih.gov/entrez/query.fcgi?cmd=Retrieve&db=Protein&list_uids=446919348&dopt=GenPept)
338. . [Leptospira kirschneri str. 200802841](http://www.ncbi.nlm.nih.gov/Taxonomy/Browser/wwwtax.cgi?id=1193047) .............................. 99 [1 hit](http://blast.ncbi.nlm.nih.gov/Blast.cgi?CMD=Get&RID=DAWEZ5AS01R&FORMAT_OBJECT=TaxBlast&NCBI_GI=off&DESCRIPTIONS=100&ALIGNMENTS=100&FORMAT_BLOCK_ON_RESPAGE=Top&MASK_COLOR=1&MASK_CHAR=2#1193047) [[spirochetes](http://www.ncbi.nlm.nih.gov/Taxonomy/Browser/wwwtax.cgi?id=136)] [SET domain protein [Leptospira kirschneri] >gi|409998863|gb](http://www.ncbi.nlm.nih.gov/entrez/query.fcgi?cmd=Retrieve&db=Protein&list_uids=409998863&dopt=GenPept)
339. . [Leptospira kirschneri serovar Grippotyphosa str. Moskva](http://www.ncbi.nlm.nih.gov/Taxonomy/Browser/wwwtax.cgi?id=1049945) ........... 99 [1 hit](http://blast.ncbi.nlm.nih.gov/Blast.cgi?CMD=Get&RID=DAWEZ5AS01R&FORMAT_OBJECT=TaxBlast&NCBI_GI=off&DESCRIPTIONS=100&ALIGNMENTS=100&FORMAT_BLOCK_ON_RESPAGE=Top&MASK_COLOR=1&MASK_CHAR=2#1049945) [[spirochetes](http://www.ncbi.nlm.nih.gov/Taxonomy/Browser/wwwtax.cgi?id=136)] [SET domain protein [Leptospira kirschneri] >gi|409998863|gb](http://www.ncbi.nlm.nih.gov/entrez/query.fcgi?cmd=Retrieve&db=Protein&list_uids=410739580&dopt=GenPept)
340. . [Leptospira kirschneri serovar Valbuzzi str. 200702274](http://www.ncbi.nlm.nih.gov/Taxonomy/Browser/wwwtax.cgi?id=1049968) ............. 99 [1 hit](http://blast.ncbi.nlm.nih.gov/Blast.cgi?CMD=Get&RID=DAWEZ5AS01R&FORMAT_OBJECT=TaxBlast&NCBI_GI=off&DESCRIPTIONS=100&ALIGNMENTS=100&FORMAT_BLOCK_ON_RESPAGE=Top&MASK_COLOR=1&MASK_CHAR=2#1049968) [[spirochetes](http://www.ncbi.nlm.nih.gov/Taxonomy/Browser/wwwtax.cgi?id=136)] [SET domain protein [Leptospira kirschneri] >gi|409998863|gb](http://www.ncbi.nlm.nih.gov/entrez/query.fcgi?cmd=Retrieve&db=Protein&list_uids=410749755&dopt=GenPept)
341. . [Leptospira kirschneri serovar Bim str. PUO 1247](http://www.ncbi.nlm.nih.gov/Taxonomy/Browser/wwwtax.cgi?id=1240688) ................... 99 [1 hit](http://blast.ncbi.nlm.nih.gov/Blast.cgi?CMD=Get&RID=DAWEZ5AS01R&FORMAT_OBJECT=TaxBlast&NCBI_GI=off&DESCRIPTIONS=100&ALIGNMENTS=100&FORMAT_BLOCK_ON_RESPAGE=Top&MASK_COLOR=1&MASK_CHAR=2#1240688) [[spirochetes](http://www.ncbi.nlm.nih.gov/Taxonomy/Browser/wwwtax.cgi?id=136)] [SET domain protein [Leptospira kirschneri] >gi|409998863|gb](http://www.ncbi.nlm.nih.gov/entrez/query.fcgi?cmd=Retrieve&db=Protein&list_uids=463355519&dopt=GenPept)
342. . [Leptospira kirschneri serovar Bim str. 1051](http://www.ncbi.nlm.nih.gov/Taxonomy/Browser/wwwtax.cgi?id=1049941) ....................... 99 [1 hit](http://blast.ncbi.nlm.nih.gov/Blast.cgi?CMD=Get&RID=DAWEZ5AS01R&FORMAT_OBJECT=TaxBlast&NCBI_GI=off&DESCRIPTIONS=100&ALIGNMENTS=100&FORMAT_BLOCK_ON_RESPAGE=Top&MASK_COLOR=1&MASK_CHAR=2#1049941) [[spirochetes](http://www.ncbi.nlm.nih.gov/Taxonomy/Browser/wwwtax.cgi?id=136)] [SET domain protein [Leptospira kirschneri] >gi|409998863|gb](http://www.ncbi.nlm.nih.gov/entrez/query.fcgi?cmd=Retrieve&db=Protein&list_uids=464184990&dopt=GenPept)
343. . [Leptospira kirschneri serovar Sokoine str. RM1](http://www.ncbi.nlm.nih.gov/Taxonomy/Browser/wwwtax.cgi?id=1049964) .................... 99 [1 hit](http://blast.ncbi.nlm.nih.gov/Blast.cgi?CMD=Get&RID=DAWEZ5AS01R&FORMAT_OBJECT=TaxBlast&NCBI_GI=off&DESCRIPTIONS=100&ALIGNMENTS=100&FORMAT_BLOCK_ON_RESPAGE=Top&MASK_COLOR=1&MASK_CHAR=2#1049964) [[spirochetes](http://www.ncbi.nlm.nih.gov/Taxonomy/Browser/wwwtax.cgi?id=136)] [SET domain protein [Leptospira kirschneri] >gi|409998863|gb](http://www.ncbi.nlm.nih.gov/entrez/query.fcgi?cmd=Retrieve&db=Protein&list_uids=464212895&dopt=GenPept)
344. . [Leptospira kirschneri str. 200803703](http://www.ncbi.nlm.nih.gov/Taxonomy/Browser/wwwtax.cgi?id=1193048) .............................. 99 [1 hit](http://blast.ncbi.nlm.nih.gov/Blast.cgi?CMD=Get&RID=DAWEZ5AS01R&FORMAT_OBJECT=TaxBlast&NCBI_GI=off&DESCRIPTIONS=100&ALIGNMENTS=100&FORMAT_BLOCK_ON_RESPAGE=Top&MASK_COLOR=1&MASK_CHAR=2#1193048) [[spirochetes](http://www.ncbi.nlm.nih.gov/Taxonomy/Browser/wwwtax.cgi?id=136)] [SET domain protein [Leptospira kirschneri] >gi|409998863|gb](http://www.ncbi.nlm.nih.gov/entrez/query.fcgi?cmd=Retrieve&db=Protein&list_uids=464398634&dopt=GenPept)
345. . [Leptospira kirschneri str. 200801925](http://www.ncbi.nlm.nih.gov/Taxonomy/Browser/wwwtax.cgi?id=1193046) .............................. 99 [1 hit](http://blast.ncbi.nlm.nih.gov/Blast.cgi?CMD=Get&RID=DAWEZ5AS01R&FORMAT_OBJECT=TaxBlast&NCBI_GI=off&DESCRIPTIONS=100&ALIGNMENTS=100&FORMAT_BLOCK_ON_RESPAGE=Top&MASK_COLOR=1&MASK_CHAR=2#1193046) [[spirochetes](http://www.ncbi.nlm.nih.gov/Taxonomy/Browser/wwwtax.cgi?id=136)] [SET domain protein [Leptospira kirschneri] >gi|409998863|gb](http://www.ncbi.nlm.nih.gov/entrez/query.fcgi?cmd=Retrieve&db=Protein&list_uids=464408965&dopt=GenPept)
346. . [Leptospira kirschneri str. 200801774](http://www.ncbi.nlm.nih.gov/Taxonomy/Browser/wwwtax.cgi?id=1193045) .............................. 99 [1 hit](http://blast.ncbi.nlm.nih.gov/Blast.cgi?CMD=Get&RID=DAWEZ5AS01R&FORMAT_OBJECT=TaxBlast&NCBI_GI=off&DESCRIPTIONS=100&ALIGNMENTS=100&FORMAT_BLOCK_ON_RESPAGE=Top&MASK_COLOR=1&MASK_CHAR=2#1193045) [[spirochetes](http://www.ncbi.nlm.nih.gov/Taxonomy/Browser/wwwtax.cgi?id=136)] [SET domain protein [Leptospira kirschneri] >gi|409998863|gb](http://www.ncbi.nlm.nih.gov/entrez/query.fcgi?cmd=Retrieve&db=Protein&list_uids=464415511&dopt=GenPept)
347. . [Leptospira kirschneri serovar Cynopteri str. 3522 CT](http://www.ncbi.nlm.nih.gov/Taxonomy/Browser/wwwtax.cgi?id=1049942) .............. 99 [1 hit](http://blast.ncbi.nlm.nih.gov/Blast.cgi?CMD=Get&RID=DAWEZ5AS01R&FORMAT_OBJECT=TaxBlast&NCBI_GI=off&DESCRIPTIONS=100&ALIGNMENTS=100&FORMAT_BLOCK_ON_RESPAGE=Top&MASK_COLOR=1&MASK_CHAR=2#1049942) [[spirochetes](http://www.ncbi.nlm.nih.gov/Taxonomy/Browser/wwwtax.cgi?id=136)] [SET domain protein [Leptospira kirschneri] >gi|409998863|gb](http://www.ncbi.nlm.nih.gov/entrez/query.fcgi?cmd=Retrieve&db=Protein&list_uids=514168554&dopt=GenPept)
348. . [Leptospira interrogans serovar Copenhageni str. Fiocruz L1-130](http://www.ncbi.nlm.nih.gov/Taxonomy/Browser/wwwtax.cgi?id=267671) .... 99 [2 hits](http://blast.ncbi.nlm.nih.gov/Blast.cgi?CMD=Get&RID=DAWEZ5AS01R&FORMAT_OBJECT=TaxBlast&NCBI_GI=off&DESCRIPTIONS=100&ALIGNMENTS=100&FORMAT_BLOCK_ON_RESPAGE=Top&MASK_COLOR=1&MASK_CHAR=2#267671) [[spirochetes](http://www.ncbi.nlm.nih.gov/Taxonomy/Browser/wwwtax.cgi?id=136)] [hypothetical protein LIC12974 [Leptospira interrogans serov](http://www.ncbi.nlm.nih.gov/entrez/query.fcgi?cmd=Retrieve&db=Protein&list_uids=45658801&dopt=GenPept)
349. . [Leptospira interrogans serovar Copenhageni str. LT2050](http://www.ncbi.nlm.nih.gov/Taxonomy/Browser/wwwtax.cgi?id=1001598) ............ 99 [1 hit](http://blast.ncbi.nlm.nih.gov/Blast.cgi?CMD=Get&RID=DAWEZ5AS01R&FORMAT_OBJECT=TaxBlast&NCBI_GI=off&DESCRIPTIONS=100&ALIGNMENTS=100&FORMAT_BLOCK_ON_RESPAGE=Top&MASK_COLOR=1&MASK_CHAR=2#1001598) [[spirochetes](http://www.ncbi.nlm.nih.gov/Taxonomy/Browser/wwwtax.cgi?id=136)] [SET domain protein [Leptospira interrogans] >gi|456985825|g](http://www.ncbi.nlm.nih.gov/entrez/query.fcgi?cmd=Retrieve&db=Protein&list_uids=456985825&dopt=GenPept)
350. . [Streptomyces sp. ATexAB-D23](http://www.ncbi.nlm.nih.gov/Taxonomy/Browser/wwwtax.cgi?id=1157635) ....................................... 99 [1 hit](http://blast.ncbi.nlm.nih.gov/Blast.cgi?CMD=Get&RID=DAWEZ5AS01R&FORMAT_OBJECT=TaxBlast&NCBI_GI=off&DESCRIPTIONS=100&ALIGNMENTS=100&FORMAT_BLOCK_ON_RESPAGE=Top&MASK_COLOR=1&MASK_CHAR=2#1157635) [[high GC Gram+](http://www.ncbi.nlm.nih.gov/Taxonomy/Browser/wwwtax.cgi?id=1760)] [hypothetical protein [Streptomyces sp. ATexAB-D23]](http://www.ncbi.nlm.nih.gov/entrez/query.fcgi?cmd=Retrieve&db=Protein&list_uids=517377486&dopt=GenPept)
351. . [Leptospira interrogans serovar Lai str. 56601](http://www.ncbi.nlm.nih.gov/Taxonomy/Browser/wwwtax.cgi?id=189518) ..................... 98 [2 hits](http://blast.ncbi.nlm.nih.gov/Blast.cgi?CMD=Get&RID=DAWEZ5AS01R&FORMAT_OBJECT=TaxBlast&NCBI_GI=off&DESCRIPTIONS=100&ALIGNMENTS=100&FORMAT_BLOCK_ON_RESPAGE=Top&MASK_COLOR=1&MASK_CHAR=2#189518) [[spirochetes](http://www.ncbi.nlm.nih.gov/Taxonomy/Browser/wwwtax.cgi?id=136)] [SET family protein [Leptospira interrogans serovar Lai str.](http://www.ncbi.nlm.nih.gov/entrez/query.fcgi?cmd=Retrieve&db=Protein&list_uids=294827686&dopt=GenPept)
352. . [Leptospira interrogans serovar Lai str. IPAV](http://www.ncbi.nlm.nih.gov/Taxonomy/Browser/wwwtax.cgi?id=573825) ...................... 98 [2 hits](http://blast.ncbi.nlm.nih.gov/Blast.cgi?CMD=Get&RID=DAWEZ5AS01R&FORMAT_OBJECT=TaxBlast&NCBI_GI=off&DESCRIPTIONS=100&ALIGNMENTS=100&FORMAT_BLOCK_ON_RESPAGE=Top&MASK_COLOR=1&MASK_CHAR=2#573825) [[spirochetes](http://www.ncbi.nlm.nih.gov/Taxonomy/Browser/wwwtax.cgi?id=136)] [SET family protein [Leptospira interrogans serovar Lai str.](http://www.ncbi.nlm.nih.gov/entrez/query.fcgi?cmd=Retrieve&db=Protein&list_uids=386072973&dopt=GenPept)
353. . [Leptospira interrogans serovar Bulgarica str. Mallika](http://www.ncbi.nlm.nih.gov/Taxonomy/Browser/wwwtax.cgi?id=1001586) ............. 98 [1 hit](http://blast.ncbi.nlm.nih.gov/Blast.cgi?CMD=Get&RID=DAWEZ5AS01R&FORMAT_OBJECT=TaxBlast&NCBI_GI=off&DESCRIPTIONS=100&ALIGNMENTS=100&FORMAT_BLOCK_ON_RESPAGE=Top&MASK_COLOR=1&MASK_CHAR=2#1001586) [[spirochetes](http://www.ncbi.nlm.nih.gov/Taxonomy/Browser/wwwtax.cgi?id=136)] [SET family protein [Leptospira interrogans serovar Lai str.](http://www.ncbi.nlm.nih.gov/entrez/query.fcgi?cmd=Retrieve&db=Protein&list_uids=400351984&dopt=GenPept)
354. . [Pusillimonas sp. T7-7](http://www.ncbi.nlm.nih.gov/Taxonomy/Browser/wwwtax.cgi?id=1007105) ......................................... 93 [3 hits](http://blast.ncbi.nlm.nih.gov/Blast.cgi?CMD=Get&RID=5Y3VDS5A013&FORMAT_OBJECT=TaxBlast&NCBI_GI=off&DESCRIPTIONS=500&ALIGNMENTS=250&FORMAT_BLOCK_ON_RESPAGE=Top&MASK_COLOR=1&MASK_CHAR=2#1007105) [[b-proteobacteria](http://www.ncbi.nlm.nih.gov/Taxonomy/Browser/wwwtax.cgi?id=28216)] [hypothetical protein PT7_3014 [Pusillimonas sp. T7-7] >gi|5](http://www.ncbi.nlm.nih.gov/entrez/query.fcgi?cmd=Retrieve&db=Protein&list_uids=332286267&dopt=GenPept)
355. . [Burkholderia rhizoxinica HKI 454](http://www.ncbi.nlm.nih.gov/Taxonomy/Browser/wwwtax.cgi?id=882378) .............................. 94 [2 hits](http://blast.ncbi.nlm.nih.gov/Blast.cgi?CMD=Get&RID=5Y3VDS5A013&FORMAT_OBJECT=TaxBlast&NCBI_GI=off&DESCRIPTIONS=500&ALIGNMENTS=250&FORMAT_BLOCK_ON_RESPAGE=Top&MASK_COLOR=1&MASK_CHAR=2#882378) [[b-proteobacteria](http://www.ncbi.nlm.nih.gov/Taxonomy/Browser/wwwtax.cgi?id=28216)] [zinc finger protein [Burkholderia rhizoxinica HKI 454] >gi|](http://www.ncbi.nlm.nih.gov/entrez/query.fcgi?cmd=Retrieve&db=Protein&list_uids=312794867&dopt=GenPept)
356. . [Burkholderia rhizoxinica](http://www.ncbi.nlm.nih.gov/Taxonomy/Browser/wwwtax.cgi?id=412963) ...................................... 94 [1 hit](http://blast.ncbi.nlm.nih.gov/Blast.cgi?CMD=Get&RID=5Y3VDS5A013&FORMAT_OBJECT=TaxBlast&NCBI_GI=off&DESCRIPTIONS=500&ALIGNMENTS=250&FORMAT_BLOCK_ON_RESPAGE=Top&MASK_COLOR=1&MASK_CHAR=2#412963) [[b-proteobacteria](http://www.ncbi.nlm.nih.gov/Taxonomy/Browser/wwwtax.cgi?id=28216)] [zinc finger protein [Burkholderia rhizoxinica HKI 454] >gi|](http://www.ncbi.nlm.nih.gov/entrez/query.fcgi?cmd=Retrieve&db=Protein&list_uids=503199222&dopt=GenPept)
357. . [Chlamydia psittaci 10_881_SC42](http://www.ncbi.nlm.nih.gov/Taxonomy/Browser/wwwtax.cgi?id=1238235) ................................ 94 [1 hit](http://blast.ncbi.nlm.nih.gov/Blast.cgi?CMD=Get&RID=5Y3VDS5A013&FORMAT_OBJECT=TaxBlast&NCBI_GI=off&DESCRIPTIONS=500&ALIGNMENTS=250&FORMAT_BLOCK_ON_RESPAGE=Top&MASK_COLOR=1&MASK_CHAR=2#1238235) [[chlamydias](http://www.ncbi.nlm.nih.gov/Taxonomy/Browser/wwwtax.cgi?id=51291)] [SET domain protein [Chlamydia psittaci] >gi|519795995|gb|EP](http://www.ncbi.nlm.nih.gov/entrez/query.fcgi?cmd=Retrieve&db=Protein&list_uids=519795995&dopt=GenPept)
358. . [Acidovorax sp. CF316](http://www.ncbi.nlm.nih.gov/Taxonomy/Browser/wwwtax.cgi?id=1144317) .......................................... 94 [2 hits](http://blast.ncbi.nlm.nih.gov/Blast.cgi?CMD=Get&RID=5Y3VDS5A013&FORMAT_OBJECT=TaxBlast&NCBI_GI=off&DESCRIPTIONS=500&ALIGNMENTS=250&FORMAT_BLOCK_ON_RESPAGE=Top&MASK_COLOR=1&MASK_CHAR=2#1144317) [[b-proteobacteria](http://www.ncbi.nlm.nih.gov/Taxonomy/Browser/wwwtax.cgi?id=28216)] [lysine methyltransferase [Acidovorax sp. CF316] >gi|3943149](http://www.ncbi.nlm.nih.gov/entrez/query.fcgi?cmd=Retrieve&db=Protein&list_uids=495131321&dopt=GenPept)
359. . [Bradyrhizobium japonicum USDA 6](http://www.ncbi.nlm.nih.gov/Taxonomy/Browser/wwwtax.cgi?id=1037409) ............................... 93 [2 hits](http://blast.ncbi.nlm.nih.gov/Blast.cgi?CMD=Get&RID=5Y3VDS5A013&FORMAT_OBJECT=TaxBlast&NCBI_GI=off&DESCRIPTIONS=500&ALIGNMENTS=250&FORMAT_BLOCK_ON_RESPAGE=Top&MASK_COLOR=1&MASK_CHAR=2#1037409) [[a-proteobacteria](http://www.ncbi.nlm.nih.gov/Taxonomy/Browser/wwwtax.cgi?id=28211)] [hypothetical protein BJ6T_39220 [Bradyrhizobium japonicum U](http://www.ncbi.nlm.nih.gov/entrez/query.fcgi?cmd=Retrieve&db=Protein&list_uids=384217618&dopt=GenPept)
360. . [Acidovorax sp. MR-S7](http://www.ncbi.nlm.nih.gov/Taxonomy/Browser/wwwtax.cgi?id=1268622) .......................................... 93 [2 hits](http://blast.ncbi.nlm.nih.gov/Blast.cgi?CMD=Get&RID=5Y3VDS5A013&FORMAT_OBJECT=TaxBlast&NCBI_GI=off&DESCRIPTIONS=500&ALIGNMENTS=250&FORMAT_BLOCK_ON_RESPAGE=Top&MASK_COLOR=1&MASK_CHAR=2#1268622) [[b-proteobacteria](http://www.ncbi.nlm.nih.gov/Taxonomy/Browser/wwwtax.cgi?id=28216)] [lysine methyltransferase [Acidovorax sp. MR-S7] >gi|5245571](http://www.ncbi.nlm.nih.gov/entrez/query.fcgi?cmd=Retrieve&db=Protein&list_uids=519073372&dopt=GenPept)
361. . [Chlorobaculum parvum NCIB 8327](http://www.ncbi.nlm.nih.gov/Taxonomy/Browser/wwwtax.cgi?id=517417) ................................ 93 [2 hits](http://blast.ncbi.nlm.nih.gov/Blast.cgi?CMD=Get&RID=5Y3VDS5A013&FORMAT_OBJECT=TaxBlast&NCBI_GI=off&DESCRIPTIONS=500&ALIGNMENTS=250&FORMAT_BLOCK_ON_RESPAGE=Top&MASK_COLOR=1&MASK_CHAR=2#517417) [[green sulfur bacteria](http://www.ncbi.nlm.nih.gov/Taxonomy/Browser/wwwtax.cgi?id=1090)] [nuclear protein SET [Chlorobaculum parvum NCIB 8327] >gi|50](http://www.ncbi.nlm.nih.gov/entrez/query.fcgi?cmd=Retrieve&db=Protein&list_uids=193212820&dopt=GenPept)
362. . [Chlorobaculum parvum](http://www.ncbi.nlm.nih.gov/Taxonomy/Browser/wwwtax.cgi?id=274539) .......................................... 93 [1 hit](http://blast.ncbi.nlm.nih.gov/Blast.cgi?CMD=Get&RID=5Y3VDS5A013&FORMAT_OBJECT=TaxBlast&NCBI_GI=off&DESCRIPTIONS=500&ALIGNMENTS=250&FORMAT_BLOCK_ON_RESPAGE=Top&MASK_COLOR=1&MASK_CHAR=2#274539) [[green sulfur bacteria](http://www.ncbi.nlm.nih.gov/Taxonomy/Browser/wwwtax.cgi?id=1090)] [nuclear protein SET [Chlorobaculum parvum NCIB 8327] >gi|50](http://www.ncbi.nlm.nih.gov/entrez/query.fcgi?cmd=Retrieve&db=Protein&list_uids=501494025&dopt=GenPept)
363. . [Hylemonella gracilis](http://www.ncbi.nlm.nih.gov/Taxonomy/Browser/wwwtax.cgi?id=80880) .......................................... 93 [1 hit](http://blast.ncbi.nlm.nih.gov/Blast.cgi?CMD=Get&RID=5Y3VDS5A013&FORMAT_OBJECT=TaxBlast&NCBI_GI=off&DESCRIPTIONS=500&ALIGNMENTS=250&FORMAT_BLOCK_ON_RESPAGE=Top&MASK_COLOR=1&MASK_CHAR=2#80880) [[b-proteobacteria](http://www.ncbi.nlm.nih.gov/Taxonomy/Browser/wwwtax.cgi?id=28216)] [nuclear protein SET [Hylemonella gracilis] >gi|332039284|gb](http://www.ncbi.nlm.nih.gov/entrez/query.fcgi?cmd=Retrieve&db=Protein&list_uids=493342152&dopt=GenPept)
364. . [Hylemonella gracilis ATCC 19624](http://www.ncbi.nlm.nih.gov/Taxonomy/Browser/wwwtax.cgi?id=887062) ............................... 93 [1 hit](http://blast.ncbi.nlm.nih.gov/Blast.cgi?CMD=Get&RID=5Y3VDS5A013&FORMAT_OBJECT=TaxBlast&NCBI_GI=off&DESCRIPTIONS=500&ALIGNMENTS=250&FORMAT_BLOCK_ON_RESPAGE=Top&MASK_COLOR=1&MASK_CHAR=2#887062) [[b-proteobacteria](http://www.ncbi.nlm.nih.gov/Taxonomy/Browser/wwwtax.cgi?id=28216)] [nuclear protein SET [Hylemonella gracilis] >gi|332039284|gb](http://www.ncbi.nlm.nih.gov/entrez/query.fcgi?cmd=Retrieve&db=Protein&list_uids=332039284&dopt=GenPept)
365. . [Burkholderiales bacterium JOSHI_001](http://www.ncbi.nlm.nih.gov/Taxonomy/Browser/wwwtax.cgi?id=864051) ........................... 93 [2 hits](http://blast.ncbi.nlm.nih.gov/Blast.cgi?CMD=Get&RID=5Y3VDS5A013&FORMAT_OBJECT=TaxBlast&NCBI_GI=off&DESCRIPTIONS=500&ALIGNMENTS=250&FORMAT_BLOCK_ON_RESPAGE=Top&MASK_COLOR=1&MASK_CHAR=2#864051) [[b-proteobacteria](http://www.ncbi.nlm.nih.gov/Taxonomy/Browser/wwwtax.cgi?id=28216)] [SET domain-containing protein [Burkholderiales bacterium JO](http://www.ncbi.nlm.nih.gov/entrez/query.fcgi?cmd=Retrieve&db=Protein&list_uids=497234032&dopt=GenPept)
366. . [Rhodanobacter fulvus](http://www.ncbi.nlm.nih.gov/Taxonomy/Browser/wwwtax.cgi?id=219571) .......................................... 93 [1 hit](http://blast.ncbi.nlm.nih.gov/Blast.cgi?CMD=Get&RID=5Y3VDS5A013&FORMAT_OBJECT=TaxBlast&NCBI_GI=off&DESCRIPTIONS=500&ALIGNMENTS=250&FORMAT_BLOCK_ON_RESPAGE=Top&MASK_COLOR=1&MASK_CHAR=2#219571) [[g-proteobacteria](http://www.ncbi.nlm.nih.gov/Taxonomy/Browser/wwwtax.cgi?id=1236)] [nuclear protein SET [Rhodanobacter fulvus] >gi|388434184|gb](http://www.ncbi.nlm.nih.gov/entrez/query.fcgi?cmd=Retrieve&db=Protein&list_uids=494140798&dopt=GenPept)
367. . [Rhodanobacter fulvus Jip2](http://www.ncbi.nlm.nih.gov/Taxonomy/Browser/wwwtax.cgi?id=1163408) ..................................... 93 [1 hit](http://blast.ncbi.nlm.nih.gov/Blast.cgi?CMD=Get&RID=5Y3VDS5A013&FORMAT_OBJECT=TaxBlast&NCBI_GI=off&DESCRIPTIONS=500&ALIGNMENTS=250&FORMAT_BLOCK_ON_RESPAGE=Top&MASK_COLOR=1&MASK_CHAR=2#1163408) [[g-proteobacteria](http://www.ncbi.nlm.nih.gov/Taxonomy/Browser/wwwtax.cgi?id=1236)] [nuclear protein SET [Rhodanobacter fulvus] >gi|388434184|gb](http://www.ncbi.nlm.nih.gov/entrez/query.fcgi?cmd=Retrieve&db=Protein&list_uids=388434184&dopt=GenPept)
368. . [Chlamydia psittaci 10_743_SC13](http://www.ncbi.nlm.nih.gov/Taxonomy/Browser/wwwtax.cgi?id=1238236) ................................ 94 [1 hit](http://blast.ncbi.nlm.nih.gov/Blast.cgi?CMD=Get&RID=5Y3VDS5A013&FORMAT_OBJECT=TaxBlast&NCBI_GI=off&DESCRIPTIONS=500&ALIGNMENTS=250&FORMAT_BLOCK_ON_RESPAGE=Top&MASK_COLOR=1&MASK_CHAR=2#1238236) [[chlamydias](http://www.ncbi.nlm.nih.gov/Taxonomy/Browser/wwwtax.cgi?id=51291)] [SET domain protein [Chlamydia psittaci] >gi|519791860|gb|EP](http://www.ncbi.nlm.nih.gov/entrez/query.fcgi?cmd=Retrieve&db=Protein&list_uids=519791860&dopt=GenPept)
369. . [Burkholderia sp. CCGE1001](http://www.ncbi.nlm.nih.gov/Taxonomy/Browser/wwwtax.cgi?id=640510) ..................................... 93 [3 hits](http://blast.ncbi.nlm.nih.gov/Blast.cgi?CMD=Get&RID=5Y3VDS5A013&FORMAT_OBJECT=TaxBlast&NCBI_GI=off&DESCRIPTIONS=500&ALIGNMENTS=250&FORMAT_BLOCK_ON_RESPAGE=Top&MASK_COLOR=1&MASK_CHAR=2#640510) [[b-proteobacteria](http://www.ncbi.nlm.nih.gov/Taxonomy/Browser/wwwtax.cgi?id=28216)] [nuclear protein SET [Burkholderia sp. CCGE1001] >gi|5033554](http://www.ncbi.nlm.nih.gov/entrez/query.fcgi?cmd=Retrieve&db=Protein&list_uids=323527871&dopt=GenPept)
370. . [Verminephrobacter aporrectodeae](http://www.ncbi.nlm.nih.gov/Taxonomy/Browser/wwwtax.cgi?id=1110389) ............................... 93 [1 hit](http://blast.ncbi.nlm.nih.gov/Blast.cgi?CMD=Get&RID=5Y3VDS5A013&FORMAT_OBJECT=TaxBlast&NCBI_GI=off&DESCRIPTIONS=500&ALIGNMENTS=250&FORMAT_BLOCK_ON_RESPAGE=Top&MASK_COLOR=1&MASK_CHAR=2#1110389) [[b-proteobacteria](http://www.ncbi.nlm.nih.gov/Taxonomy/Browser/wwwtax.cgi?id=28216)] [nuclear protein SET, partial [Verminephrobacter aporrectode](http://www.ncbi.nlm.nih.gov/entrez/query.fcgi?cmd=Retrieve&db=Protein&list_uids=497791758&dopt=GenPept)
371. . [Rhodopseudomonas palustris BisB5](http://www.ncbi.nlm.nih.gov/Taxonomy/Browser/wwwtax.cgi?id=316057) .............................. 95 [2 hits](http://blast.ncbi.nlm.nih.gov/Blast.cgi?CMD=Get&RID=5Y3VDS5A013&FORMAT_OBJECT=TaxBlast&NCBI_GI=off&DESCRIPTIONS=500&ALIGNMENTS=250&FORMAT_BLOCK_ON_RESPAGE=Top&MASK_COLOR=1&MASK_CHAR=2#316057) [[a-proteobacteria](http://www.ncbi.nlm.nih.gov/Taxonomy/Browser/wwwtax.cgi?id=28211)] [nuclear protein SET [Rhodopseudomonas palustris BisB5] >gi|](http://www.ncbi.nlm.nih.gov/entrez/query.fcgi?cmd=Retrieve&db=Protein&list_uids=91976192&dopt=GenPept)
372. . [Pelodictyon phaeoclathratiforme BU-1](http://www.ncbi.nlm.nih.gov/Taxonomy/Browser/wwwtax.cgi?id=324925) .......................... 92 [2 hits](http://blast.ncbi.nlm.nih.gov/Blast.cgi?CMD=Get&RID=5Y3VDS5A013&FORMAT_OBJECT=TaxBlast&NCBI_GI=off&DESCRIPTIONS=500&ALIGNMENTS=250&FORMAT_BLOCK_ON_RESPAGE=Top&MASK_COLOR=1&MASK_CHAR=2#324925) [[green sulfur bacteria](http://www.ncbi.nlm.nih.gov/Taxonomy/Browser/wwwtax.cgi?id=1090)] [nuclear protein SET [Pelodictyon phaeoclathratiforme BU-1]](http://www.ncbi.nlm.nih.gov/entrez/query.fcgi?cmd=Retrieve&db=Protein&list_uids=194336362&dopt=GenPept)
373. . [Pelodictyon phaeoclathratiforme](http://www.ncbi.nlm.nih.gov/Taxonomy/Browser/wwwtax.cgi?id=34090) ............................... 92 [1 hit](http://blast.ncbi.nlm.nih.gov/Blast.cgi?CMD=Get&RID=5Y3VDS5A013&FORMAT_OBJECT=TaxBlast&NCBI_GI=off&DESCRIPTIONS=500&ALIGNMENTS=250&FORMAT_BLOCK_ON_RESPAGE=Top&MASK_COLOR=1&MASK_CHAR=2#34090) [[green sulfur bacteria](http://www.ncbi.nlm.nih.gov/Taxonomy/Browser/wwwtax.cgi?id=1090)] [nuclear protein SET [Pelodictyon phaeoclathratiforme BU-1]](http://www.ncbi.nlm.nih.gov/entrez/query.fcgi?cmd=Retrieve&db=Protein&list_uids=501499791&dopt=GenPept)
374. . [Burkholderia sp. YI23](http://www.ncbi.nlm.nih.gov/Taxonomy/Browser/wwwtax.cgi?id=1097668) ......................................... 92 [6 hits](http://blast.ncbi.nlm.nih.gov/Blast.cgi?CMD=Get&RID=5Y3VDS5A013&FORMAT_OBJECT=TaxBlast&NCBI_GI=off&DESCRIPTIONS=500&ALIGNMENTS=250&FORMAT_BLOCK_ON_RESPAGE=Top&MASK_COLOR=1&MASK_CHAR=2#1097668) [[b-proteobacteria](http://www.ncbi.nlm.nih.gov/Taxonomy/Browser/wwwtax.cgi?id=28216)] [nuclear protein [Burkholderia sp. YI23] >gi|504019207|ref|W](http://www.ncbi.nlm.nih.gov/entrez/query.fcgi?cmd=Retrieve&db=Protein&list_uids=377812033&dopt=GenPept)
375. . [Burkholderia sp. CCGE1002](http://www.ncbi.nlm.nih.gov/Taxonomy/Browser/wwwtax.cgi?id=640511) ..................................... 92 [6 hits](http://blast.ncbi.nlm.nih.gov/Blast.cgi?CMD=Get&RID=5Y3VDS5A013&FORMAT_OBJECT=TaxBlast&NCBI_GI=off&DESCRIPTIONS=500&ALIGNMENTS=250&FORMAT_BLOCK_ON_RESPAGE=Top&MASK_COLOR=1&MASK_CHAR=2#640511) [[b-proteobacteria](http://www.ncbi.nlm.nih.gov/Taxonomy/Browser/wwwtax.cgi?id=28216)] [nuclear protein SET [Burkholderia sp. CCGE1002] >gi|5028596](http://www.ncbi.nlm.nih.gov/entrez/query.fcgi?cmd=Retrieve&db=Protein&list_uids=295701379&dopt=GenPept)
376. . [Polynucleobacter necessarius subsp. necessarius STIR1](http://www.ncbi.nlm.nih.gov/Taxonomy/Browser/wwwtax.cgi?id=452638) ......... 92 [2 hits](http://blast.ncbi.nlm.nih.gov/Blast.cgi?CMD=Get&RID=5Y3VDS5A013&FORMAT_OBJECT=TaxBlast&NCBI_GI=off&DESCRIPTIONS=500&ALIGNMENTS=250&FORMAT_BLOCK_ON_RESPAGE=Top&MASK_COLOR=1&MASK_CHAR=2#452638) [[b-proteobacteria](http://www.ncbi.nlm.nih.gov/Taxonomy/Browser/wwwtax.cgi?id=28216)] [nuclear protein SET [Polynucleobacter necessarius subsp. ne](http://www.ncbi.nlm.nih.gov/entrez/query.fcgi?cmd=Retrieve&db=Protein&list_uids=171462836&dopt=GenPept)
377. . [Polynucleobacter necessarius](http://www.ncbi.nlm.nih.gov/Taxonomy/Browser/wwwtax.cgi?id=576610) .................................. 92 [2 hits](http://blast.ncbi.nlm.nih.gov/Blast.cgi?CMD=Get&RID=5Y3VDS5A013&FORMAT_OBJECT=TaxBlast&NCBI_GI=off&DESCRIPTIONS=500&ALIGNMENTS=250&FORMAT_BLOCK_ON_RESPAGE=Top&MASK_COLOR=1&MASK_CHAR=2#576610) [[b-proteobacteria](http://www.ncbi.nlm.nih.gov/Taxonomy/Browser/wwwtax.cgi?id=28216)] [nuclear protein SET [Polynucleobacter necessarius subsp. ne](http://www.ncbi.nlm.nih.gov/entrez/query.fcgi?cmd=Retrieve&db=Protein&list_uids=501325469&dopt=GenPept)
378. . [Burkholderia sp. CCGE1003](http://www.ncbi.nlm.nih.gov/Taxonomy/Browser/wwwtax.cgi?id=640512) ..................................... 93 [3 hits](http://blast.ncbi.nlm.nih.gov/Blast.cgi?CMD=Get&RID=5Y3VDS5A013&FORMAT_OBJECT=TaxBlast&NCBI_GI=off&DESCRIPTIONS=500&ALIGNMENTS=250&FORMAT_BLOCK_ON_RESPAGE=Top&MASK_COLOR=1&MASK_CHAR=2#640512) [[b-proteobacteria](http://www.ncbi.nlm.nih.gov/Taxonomy/Browser/wwwtax.cgi?id=28216)] [nuclear protein SET [Burkholderia sp. CCGE1003] >gi|5031062](http://www.ncbi.nlm.nih.gov/entrez/query.fcgi?cmd=Retrieve&db=Protein&list_uids=307731489&dopt=GenPept)
379. . [Pseudoxanthomonas spadix BD-a59](http://www.ncbi.nlm.nih.gov/Taxonomy/Browser/wwwtax.cgi?id=1045855) ............................... 92 [2 hits](http://blast.ncbi.nlm.nih.gov/Blast.cgi?CMD=Get&RID=5Y3VDS5A013&FORMAT_OBJECT=TaxBlast&NCBI_GI=off&DESCRIPTIONS=500&ALIGNMENTS=250&FORMAT_BLOCK_ON_RESPAGE=Top&MASK_COLOR=1&MASK_CHAR=2#1045855) [[g-proteobacteria](http://www.ncbi.nlm.nih.gov/Taxonomy/Browser/wwwtax.cgi?id=1236)] [nuclear protein SET [Pseudoxanthomonas spadix BD-a59] >gi|5](http://www.ncbi.nlm.nih.gov/entrez/query.fcgi?cmd=Retrieve&db=Protein&list_uids=357416835&dopt=GenPept)
380. . [Pseudoxanthomonas spadix](http://www.ncbi.nlm.nih.gov/Taxonomy/Browser/wwwtax.cgi?id=415229) ...................................... 92 [1 hit](http://blast.ncbi.nlm.nih.gov/Blast.cgi?CMD=Get&RID=5Y3VDS5A013&FORMAT_OBJECT=TaxBlast&NCBI_GI=off&DESCRIPTIONS=500&ALIGNMENTS=250&FORMAT_BLOCK_ON_RESPAGE=Top&MASK_COLOR=1&MASK_CHAR=2#415229) [[g-proteobacteria](http://www.ncbi.nlm.nih.gov/Taxonomy/Browser/wwwtax.cgi?id=1236)] [nuclear protein SET [Pseudoxanthomonas spadix BD-a59] >gi|5](http://www.ncbi.nlm.nih.gov/entrez/query.fcgi?cmd=Retrieve&db=Protein&list_uids=503925997&dopt=GenPept)
381. . [Hyphomicrobium denitrificans ATCC 51888](http://www.ncbi.nlm.nih.gov/Taxonomy/Browser/wwwtax.cgi?id=582899) ....................... 92 [2 hits](http://blast.ncbi.nlm.nih.gov/Blast.cgi?CMD=Get&RID=5Y3VDS5A013&FORMAT_OBJECT=TaxBlast&NCBI_GI=off&DESCRIPTIONS=500&ALIGNMENTS=250&FORMAT_BLOCK_ON_RESPAGE=Top&MASK_COLOR=1&MASK_CHAR=2#582899) [[a-proteobacteria](http://www.ncbi.nlm.nih.gov/Taxonomy/Browser/wwwtax.cgi?id=28211)] [nuclear protein SET [Hyphomicrobium denitrificans ATCC 5188](http://www.ncbi.nlm.nih.gov/entrez/query.fcgi?cmd=Retrieve&db=Protein&list_uids=300024301&dopt=GenPept)
382. . [Hyphomicrobium denitrificans](http://www.ncbi.nlm.nih.gov/Taxonomy/Browser/wwwtax.cgi?id=53399) .................................. 92 [1 hit](http://blast.ncbi.nlm.nih.gov/Blast.cgi?CMD=Get&RID=5Y3VDS5A013&FORMAT_OBJECT=TaxBlast&NCBI_GI=off&DESCRIPTIONS=500&ALIGNMENTS=250&FORMAT_BLOCK_ON_RESPAGE=Top&MASK_COLOR=1&MASK_CHAR=2#53399) [[a-proteobacteria](http://www.ncbi.nlm.nih.gov/Taxonomy/Browser/wwwtax.cgi?id=28211)] [nuclear protein SET [Hyphomicrobium denitrificans ATCC 5188](http://www.ncbi.nlm.nih.gov/entrez/query.fcgi?cmd=Retrieve&db=Protein&list_uids=502981774&dopt=GenPept)
383. . [Singulisphaera acidiphila DSM 18658](http://www.ncbi.nlm.nih.gov/Taxonomy/Browser/wwwtax.cgi?id=886293) ........................... 92 [2 hits](http://blast.ncbi.nlm.nih.gov/Blast.cgi?CMD=Get&RID=5Y3VDS5A013&FORMAT_OBJECT=TaxBlast&NCBI_GI=off&DESCRIPTIONS=500&ALIGNMENTS=250&FORMAT_BLOCK_ON_RESPAGE=Top&MASK_COLOR=1&MASK_CHAR=2#886293) [[planctomycetes](http://www.ncbi.nlm.nih.gov/Taxonomy/Browser/wwwtax.cgi?id=112)] [SET domain-containing protein [Singulisphaera acidiphila DS](http://www.ncbi.nlm.nih.gov/entrez/query.fcgi?cmd=Retrieve&db=Protein&list_uids=430745383&dopt=GenPept)
384. . [Singulisphaera acidiphila](http://www.ncbi.nlm.nih.gov/Taxonomy/Browser/wwwtax.cgi?id=466153) ..................................... 92 [1 hit](http://blast.ncbi.nlm.nih.gov/Blast.cgi?CMD=Get&RID=5Y3VDS5A013&FORMAT_OBJECT=TaxBlast&NCBI_GI=off&DESCRIPTIONS=500&ALIGNMENTS=250&FORMAT_BLOCK_ON_RESPAGE=Top&MASK_COLOR=1&MASK_CHAR=2#466153) [[planctomycetes](http://www.ncbi.nlm.nih.gov/Taxonomy/Browser/wwwtax.cgi?id=112)] [SET domain-containing protein [Singulisphaera acidiphila DS](http://www.ncbi.nlm.nih.gov/entrez/query.fcgi?cmd=Retrieve&db=Protein&list_uids=505060829&dopt=GenPept)
385. . [Microvirga sp. WSM3557](http://www.ncbi.nlm.nih.gov/Taxonomy/Browser/wwwtax.cgi?id=754501) ........................................ 93 [2 hits](http://blast.ncbi.nlm.nih.gov/Blast.cgi?CMD=Get&RID=5Y3VDS5A013&FORMAT_OBJECT=TaxBlast&NCBI_GI=off&DESCRIPTIONS=500&ALIGNMENTS=250&FORMAT_BLOCK_ON_RESPAGE=Top&MASK_COLOR=1&MASK_CHAR=2#754501) [[a-proteobacteria](http://www.ncbi.nlm.nih.gov/Taxonomy/Browser/wwwtax.cgi?id=28211)] [SET domain-containing protein [Microvirga sp. WSM3557] >gi|](http://www.ncbi.nlm.nih.gov/entrez/query.fcgi?cmd=Retrieve&db=Protein&list_uids=497162698&dopt=GenPept)
386. . [Burkholderia xenovorans LB400](http://www.ncbi.nlm.nih.gov/Taxonomy/Browser/wwwtax.cgi?id=266265) ................................. 92 [2 hits](http://blast.ncbi.nlm.nih.gov/Blast.cgi?CMD=Get&RID=5Y3VDS5A013&FORMAT_OBJECT=TaxBlast&NCBI_GI=off&DESCRIPTIONS=500&ALIGNMENTS=250&FORMAT_BLOCK_ON_RESPAGE=Top&MASK_COLOR=1&MASK_CHAR=2#266265) [[b-proteobacteria](http://www.ncbi.nlm.nih.gov/Taxonomy/Browser/wwwtax.cgi?id=28216)] [hypothetical protein Bxe_A0006 [Burkholderia xenovorans LB4](http://www.ncbi.nlm.nih.gov/entrez/query.fcgi?cmd=Retrieve&db=Protein&list_uids=91785767&dopt=GenPept)
387. . [Burkholderia xenovorans](http://www.ncbi.nlm.nih.gov/Taxonomy/Browser/wwwtax.cgi?id=36873) ....................................... 92 [1 hit](http://blast.ncbi.nlm.nih.gov/Blast.cgi?CMD=Get&RID=5Y3VDS5A013&FORMAT_OBJECT=TaxBlast&NCBI_GI=off&DESCRIPTIONS=500&ALIGNMENTS=250&FORMAT_BLOCK_ON_RESPAGE=Top&MASK_COLOR=1&MASK_CHAR=2#36873) [[b-proteobacteria](http://www.ncbi.nlm.nih.gov/Taxonomy/Browser/wwwtax.cgi?id=28216)] [hypothetical protein Bxe_A0006 [Burkholderia xenovorans LB4](http://www.ncbi.nlm.nih.gov/entrez/query.fcgi?cmd=Retrieve&db=Protein&list_uids=499809584&dopt=GenPept)
388. . [Chlamydia](http://www.ncbi.nlm.nih.gov/Taxonomy/Browser/wwwtax.cgi?id=810) ..................................................... 93 [1 hit](http://blast.ncbi.nlm.nih.gov/Blast.cgi?CMD=Get&RID=5Y3VDS5A013&FORMAT_OBJECT=TaxBlast&NCBI_GI=off&DESCRIPTIONS=500&ALIGNMENTS=250&FORMAT_BLOCK_ON_RESPAGE=Top&MASK_COLOR=1&MASK_CHAR=2#810) [[chlamydias](http://www.ncbi.nlm.nih.gov/Taxonomy/Browser/wwwtax.cgi?id=51291)] [SET domain protein [Chlamydia] >gi|519789833|gb|EPP34599.1|](http://www.ncbi.nlm.nih.gov/entrez/query.fcgi?cmd=Retrieve&db=Protein&list_uids=520983837&dopt=GenPept)
389. . [Chlamydia psittaci 10_1398_11](http://www.ncbi.nlm.nih.gov/Taxonomy/Browser/wwwtax.cgi?id=1238237) ................................. 93 [1 hit](http://blast.ncbi.nlm.nih.gov/Blast.cgi?CMD=Get&RID=5Y3VDS5A013&FORMAT_OBJECT=TaxBlast&NCBI_GI=off&DESCRIPTIONS=500&ALIGNMENTS=250&FORMAT_BLOCK_ON_RESPAGE=Top&MASK_COLOR=1&MASK_CHAR=2#1238237) [[chlamydias](http://www.ncbi.nlm.nih.gov/Taxonomy/Browser/wwwtax.cgi?id=51291)] [SET domain protein [Chlamydia] >gi|519789833|gb|EPP34599.1|](http://www.ncbi.nlm.nih.gov/entrez/query.fcgi?cmd=Retrieve&db=Protein&list_uids=519789833&dopt=GenPept)
390. . [Chlamydia ibidis 10-1398/6](http://www.ncbi.nlm.nih.gov/Taxonomy/Browser/wwwtax.cgi?id=1046581) .................................... 93 [1 hit](http://blast.ncbi.nlm.nih.gov/Blast.cgi?CMD=Get&RID=5Y3VDS5A013&FORMAT_OBJECT=TaxBlast&NCBI_GI=off&DESCRIPTIONS=500&ALIGNMENTS=250&FORMAT_BLOCK_ON_RESPAGE=Top&MASK_COLOR=1&MASK_CHAR=2#1046581) [[chlamydias](http://www.ncbi.nlm.nih.gov/Taxonomy/Browser/wwwtax.cgi?id=51291)] [SET domain protein [Chlamydia] >gi|519789833|gb|EPP34599.1|](http://www.ncbi.nlm.nih.gov/entrez/query.fcgi?cmd=Retrieve&db=Protein&list_uids=532821155&dopt=GenPept)
391. . [Acidovorax sp. NO-1](http://www.ncbi.nlm.nih.gov/Taxonomy/Browser/wwwtax.cgi?id=512030) ........................................... 93 [2 hits](http://blast.ncbi.nlm.nih.gov/Blast.cgi?CMD=Get&RID=5Y3VDS5A013&FORMAT_OBJECT=TaxBlast&NCBI_GI=off&DESCRIPTIONS=500&ALIGNMENTS=250&FORMAT_BLOCK_ON_RESPAGE=Top&MASK_COLOR=1&MASK_CHAR=2#512030) [[b-proteobacteria](http://www.ncbi.nlm.nih.gov/Taxonomy/Browser/wwwtax.cgi?id=28216)] [lysine methyltransferase [Acidovorax sp. NO-1] >gi|36341672](http://www.ncbi.nlm.nih.gov/entrez/query.fcgi?cmd=Retrieve&db=Protein&list_uids=496179300&dopt=GenPept)
392. . [Chlamydophila pecorum E58](http://www.ncbi.nlm.nih.gov/Taxonomy/Browser/wwwtax.cgi?id=331635) ..................................... 93 [2 hits](http://blast.ncbi.nlm.nih.gov/Blast.cgi?CMD=Get&RID=5Y3VDS5A013&FORMAT_OBJECT=TaxBlast&NCBI_GI=off&DESCRIPTIONS=500&ALIGNMENTS=250&FORMAT_BLOCK_ON_RESPAGE=Top&MASK_COLOR=1&MASK_CHAR=2#331635) [[chlamydias](http://www.ncbi.nlm.nih.gov/Taxonomy/Browser/wwwtax.cgi?id=51291)] [set domain-containing protein [Chlamydophila pecorum E58] >](http://www.ncbi.nlm.nih.gov/entrez/query.fcgi?cmd=Retrieve&db=Protein&list_uids=330443898&dopt=GenPept)
393. . [Chlamydia pecorum PV3056/3](http://www.ncbi.nlm.nih.gov/Taxonomy/Browser/wwwtax.cgi?id=1234367) .................................... 93 [2 hits](http://blast.ncbi.nlm.nih.gov/Blast.cgi?CMD=Get&RID=5Y3VDS5A013&FORMAT_OBJECT=TaxBlast&NCBI_GI=off&DESCRIPTIONS=500&ALIGNMENTS=250&FORMAT_BLOCK_ON_RESPAGE=Top&MASK_COLOR=1&MASK_CHAR=2#1234367) [[chlamydias](http://www.ncbi.nlm.nih.gov/Taxonomy/Browser/wwwtax.cgi?id=51291)] [set domain-containing protein [Chlamydophila pecorum E58] >](http://www.ncbi.nlm.nih.gov/entrez/query.fcgi?cmd=Retrieve&db=Protein&list_uids=545631557&dopt=GenPept)
394. . [Chlamydia pecorum W73](http://www.ncbi.nlm.nih.gov/Taxonomy/Browser/wwwtax.cgi?id=1234368) ......................................... 93 [2 hits](http://blast.ncbi.nlm.nih.gov/Blast.cgi?CMD=Get&RID=5Y3VDS5A013&FORMAT_OBJECT=TaxBlast&NCBI_GI=off&DESCRIPTIONS=500&ALIGNMENTS=250&FORMAT_BLOCK_ON_RESPAGE=Top&MASK_COLOR=1&MASK_CHAR=2#1234368) [[chlamydias](http://www.ncbi.nlm.nih.gov/Taxonomy/Browser/wwwtax.cgi?id=51291)] [set domain-containing protein [Chlamydophila pecorum E58] >](http://www.ncbi.nlm.nih.gov/entrez/query.fcgi?cmd=Retrieve&db=Protein&list_uids=545632483&dopt=GenPept)
395. . [Chlamydia pecorum P787](http://www.ncbi.nlm.nih.gov/Taxonomy/Browser/wwwtax.cgi?id=1234369) ........................................ 93 [2 hits](http://blast.ncbi.nlm.nih.gov/Blast.cgi?CMD=Get&RID=5Y3VDS5A013&FORMAT_OBJECT=TaxBlast&NCBI_GI=off&DESCRIPTIONS=500&ALIGNMENTS=250&FORMAT_BLOCK_ON_RESPAGE=Top&MASK_COLOR=1&MASK_CHAR=2#1234369) [[chlamydias](http://www.ncbi.nlm.nih.gov/Taxonomy/Browser/wwwtax.cgi?id=51291)] [set domain-containing protein [Chlamydophila pecorum E58] >](http://www.ncbi.nlm.nih.gov/entrez/query.fcgi?cmd=Retrieve&db=Protein&list_uids=545633409&dopt=GenPept)
396. . [Chlamydia pecorum](http://www.ncbi.nlm.nih.gov/Taxonomy/Browser/wwwtax.cgi?id=85991) ............................................. 93 [1 hit](http://blast.ncbi.nlm.nih.gov/Blast.cgi?CMD=Get&RID=5Y3VDS5A013&FORMAT_OBJECT=TaxBlast&NCBI_GI=off&DESCRIPTIONS=500&ALIGNMENTS=250&FORMAT_BLOCK_ON_RESPAGE=Top&MASK_COLOR=1&MASK_CHAR=2#85991) [[chlamydias](http://www.ncbi.nlm.nih.gov/Taxonomy/Browser/wwwtax.cgi?id=51291)] [set domain-containing protein [Chlamydophila pecorum E58] >](http://www.ncbi.nlm.nih.gov/entrez/query.fcgi?cmd=Retrieve&db=Protein&list_uids=503477598&dopt=GenPept)
397. . [Chlorobium limicola DSM 245](http://www.ncbi.nlm.nih.gov/Taxonomy/Browser/wwwtax.cgi?id=290315) ................................... 92 [2 hits](http://blast.ncbi.nlm.nih.gov/Blast.cgi?CMD=Get&RID=5Y3VDS5A013&FORMAT_OBJECT=TaxBlast&NCBI_GI=off&DESCRIPTIONS=500&ALIGNMENTS=250&FORMAT_BLOCK_ON_RESPAGE=Top&MASK_COLOR=1&MASK_CHAR=2#290315) [[green sulfur bacteria](http://www.ncbi.nlm.nih.gov/Taxonomy/Browser/wwwtax.cgi?id=1090)] [nuclear protein SET [Chlorobium limicola DSM 245] >gi|50144](http://www.ncbi.nlm.nih.gov/entrez/query.fcgi?cmd=Retrieve&db=Protein&list_uids=189346934&dopt=GenPept)
398. . [Chlorobium limicola](http://www.ncbi.nlm.nih.gov/Taxonomy/Browser/wwwtax.cgi?id=1092) ........................................... 92 [1 hit](http://blast.ncbi.nlm.nih.gov/Blast.cgi?CMD=Get&RID=5Y3VDS5A013&FORMAT_OBJECT=TaxBlast&NCBI_GI=off&DESCRIPTIONS=500&ALIGNMENTS=250&FORMAT_BLOCK_ON_RESPAGE=Top&MASK_COLOR=1&MASK_CHAR=2#1092) [[green sulfur bacteria](http://www.ncbi.nlm.nih.gov/Taxonomy/Browser/wwwtax.cgi?id=1090)] [nuclear protein SET [Chlorobium limicola DSM 245] >gi|50144](http://www.ncbi.nlm.nih.gov/entrez/query.fcgi?cmd=Retrieve&db=Protein&list_uids=501442912&dopt=GenPept)
399. . [Oligotropha carboxidovorans OM5](http://www.ncbi.nlm.nih.gov/Taxonomy/Browser/wwwtax.cgi?id=504832) ............................... 94 [4 hits](http://blast.ncbi.nlm.nih.gov/Blast.cgi?CMD=Get&RID=5Y3VDS5A013&FORMAT_OBJECT=TaxBlast&NCBI_GI=off&DESCRIPTIONS=500&ALIGNMENTS=250&FORMAT_BLOCK_ON_RESPAGE=Top&MASK_COLOR=1&MASK_CHAR=2#504832) [[a-proteobacteria](http://www.ncbi.nlm.nih.gov/Taxonomy/Browser/wwwtax.cgi?id=28211)] [nuclear protein SET [Oligotropha carboxidovorans OM5] >gi|3](http://www.ncbi.nlm.nih.gov/entrez/query.fcgi?cmd=Retrieve&db=Protein&list_uids=209884557&dopt=GenPept)
400. . [Oligotropha carboxidovorans OM4](http://www.ncbi.nlm.nih.gov/Taxonomy/Browser/wwwtax.cgi?id=1031710) ............................... 94 [2 hits](http://blast.ncbi.nlm.nih.gov/Blast.cgi?CMD=Get&RID=5Y3VDS5A013&FORMAT_OBJECT=TaxBlast&NCBI_GI=off&DESCRIPTIONS=500&ALIGNMENTS=250&FORMAT_BLOCK_ON_RESPAGE=Top&MASK_COLOR=1&MASK_CHAR=2#1031710) [[a-proteobacteria](http://www.ncbi.nlm.nih.gov/Taxonomy/Browser/wwwtax.cgi?id=28211)] [nuclear protein SET [Oligotropha carboxidovorans OM5] >gi|3](http://www.ncbi.nlm.nih.gov/entrez/query.fcgi?cmd=Retrieve&db=Protein&list_uids=386030787&dopt=GenPept)
401. . [Oligotropha carboxidovorans](http://www.ncbi.nlm.nih.gov/Taxonomy/Browser/wwwtax.cgi?id=40137) ................................... 94 [1 hit](http://blast.ncbi.nlm.nih.gov/Blast.cgi?CMD=Get&RID=5Y3VDS5A013&FORMAT_OBJECT=TaxBlast&NCBI_GI=off&DESCRIPTIONS=500&ALIGNMENTS=250&FORMAT_BLOCK_ON_RESPAGE=Top&MASK_COLOR=1&MASK_CHAR=2#40137) [[a-proteobacteria](http://www.ncbi.nlm.nih.gov/Taxonomy/Browser/wwwtax.cgi?id=28211)] [nuclear protein SET [Oligotropha carboxidovorans OM5] >gi|3](http://www.ncbi.nlm.nih.gov/entrez/query.fcgi?cmd=Retrieve&db=Protein&list_uids=501558079&dopt=GenPept)
402. . [Methylibium petroleiphilum PM1](http://www.ncbi.nlm.nih.gov/Taxonomy/Browser/wwwtax.cgi?id=420662) ................................ 92 [2 hits](http://blast.ncbi.nlm.nih.gov/Blast.cgi?CMD=Get&RID=5Y3VDS5A013&FORMAT_OBJECT=TaxBlast&NCBI_GI=off&DESCRIPTIONS=500&ALIGNMENTS=250&FORMAT_BLOCK_ON_RESPAGE=Top&MASK_COLOR=1&MASK_CHAR=2#420662) [[b-proteobacteria](http://www.ncbi.nlm.nih.gov/Taxonomy/Browser/wwwtax.cgi?id=28216)] [hypothetical protein Mpe_A0264 [Methylibium petroleiphilum](http://www.ncbi.nlm.nih.gov/entrez/query.fcgi?cmd=Retrieve&db=Protein&list_uids=124265457&dopt=GenPept)
403. . [Methylibium petroleiphilum](http://www.ncbi.nlm.nih.gov/Taxonomy/Browser/wwwtax.cgi?id=105560) .................................... 92 [1 hit](http://blast.ncbi.nlm.nih.gov/Blast.cgi?CMD=Get&RID=5Y3VDS5A013&FORMAT_OBJECT=TaxBlast&NCBI_GI=off&DESCRIPTIONS=500&ALIGNMENTS=250&FORMAT_BLOCK_ON_RESPAGE=Top&MASK_COLOR=1&MASK_CHAR=2#105560) [[b-proteobacteria](http://www.ncbi.nlm.nih.gov/Taxonomy/Browser/wwwtax.cgi?id=28216)] [hypothetical protein Mpe_A0264 [Methylibium petroleiphilum](http://www.ncbi.nlm.nih.gov/entrez/query.fcgi?cmd=Retrieve&db=Protein&list_uids=500151862&dopt=GenPept)
404. . [Massilia niastensis](http://www.ncbi.nlm.nih.gov/Taxonomy/Browser/wwwtax.cgi?id=544911) ........................................... 92 [1 hit](http://blast.ncbi.nlm.nih.gov/Blast.cgi?CMD=Get&RID=5Y3VDS5A013&FORMAT_OBJECT=TaxBlast&NCBI_GI=off&DESCRIPTIONS=500&ALIGNMENTS=250&FORMAT_BLOCK_ON_RESPAGE=Top&MASK_COLOR=1&MASK_CHAR=2#544911) [[b-proteobacteria](http://www.ncbi.nlm.nih.gov/Taxonomy/Browser/wwwtax.cgi?id=28216)] [hypothetical protein [Massilia niastensis]](http://www.ncbi.nlm.nih.gov/entrez/query.fcgi?cmd=Retrieve&db=Protein&list_uids=522144529&dopt=GenPept)
405. . [Ralstonia sp. PBA](http://www.ncbi.nlm.nih.gov/Taxonomy/Browser/wwwtax.cgi?id=795666) ............................................. 93 [2 hits](http://blast.ncbi.nlm.nih.gov/Blast.cgi?CMD=Get&RID=5Y3VDS5A013&FORMAT_OBJECT=TaxBlast&NCBI_GI=off&DESCRIPTIONS=500&ALIGNMENTS=250&FORMAT_BLOCK_ON_RESPAGE=Top&MASK_COLOR=1&MASK_CHAR=2#795666) [[b-proteobacteria](http://www.ncbi.nlm.nih.gov/Taxonomy/Browser/wwwtax.cgi?id=28216)] [histone-lysine n-methyltransferase [Ralstonia sp. PBA] >gi|](http://www.ncbi.nlm.nih.gov/entrez/query.fcgi?cmd=Retrieve&db=Protein&list_uids=497208516&dopt=GenPept)
406. . [Burkholderia kururiensis](http://www.ncbi.nlm.nih.gov/Taxonomy/Browser/wwwtax.cgi?id=984307) ...................................... 92 [1 hit](http://blast.ncbi.nlm.nih.gov/Blast.cgi?CMD=Get&RID=5Y3VDS5A013&FORMAT_OBJECT=TaxBlast&NCBI_GI=off&DESCRIPTIONS=500&ALIGNMENTS=250&FORMAT_BLOCK_ON_RESPAGE=Top&MASK_COLOR=1&MASK_CHAR=2#984307) [[b-proteobacteria](http://www.ncbi.nlm.nih.gov/Taxonomy/Browser/wwwtax.cgi?id=28216)] [nuclear protein SET [Burkholderia kururiensis]](http://www.ncbi.nlm.nih.gov/entrez/query.fcgi?cmd=Retrieve&db=Protein&list_uids=516382239&dopt=GenPept)
407. . [Stigmatella aurantiaca](http://www.ncbi.nlm.nih.gov/Taxonomy/Browser/wwwtax.cgi?id=41) ........................................ 93 [2 hits](http://blast.ncbi.nlm.nih.gov/Blast.cgi?CMD=Get&RID=5Y3VDS5A013&FORMAT_OBJECT=TaxBlast&NCBI_GI=off&DESCRIPTIONS=500&ALIGNMENTS=250&FORMAT_BLOCK_ON_RESPAGE=Top&MASK_COLOR=1&MASK_CHAR=2#41) [[d-proteobacteria](http://www.ncbi.nlm.nih.gov/Taxonomy/Browser/wwwtax.cgi?id=28221)] [lysine methyltransferase [Stigmatella aurantiaca] >gi|11536](http://www.ncbi.nlm.nih.gov/entrez/query.fcgi?cmd=Retrieve&db=Protein&list_uids=488689535&dopt=GenPept)
408. . [Stigmatella aurantiaca DW4/3-1](http://www.ncbi.nlm.nih.gov/Taxonomy/Browser/wwwtax.cgi?id=378806) ................................ 93 [3 hits](http://blast.ncbi.nlm.nih.gov/Blast.cgi?CMD=Get&RID=5Y3VDS5A013&FORMAT_OBJECT=TaxBlast&NCBI_GI=off&DESCRIPTIONS=500&ALIGNMENTS=250&FORMAT_BLOCK_ON_RESPAGE=Top&MASK_COLOR=1&MASK_CHAR=2#378806) [[d-proteobacteria](http://www.ncbi.nlm.nih.gov/Taxonomy/Browser/wwwtax.cgi?id=28221)] [lysine methyltransferase [Stigmatella aurantiaca] >gi|11536](http://www.ncbi.nlm.nih.gov/entrez/query.fcgi?cmd=Retrieve&db=Protein&list_uids=115367706&dopt=GenPept)
409. . [Burkholderia sp. H160](http://www.ncbi.nlm.nih.gov/Taxonomy/Browser/wwwtax.cgi?id=516466) ......................................... 92 [2 hits](http://blast.ncbi.nlm.nih.gov/Blast.cgi?CMD=Get&RID=5Y3VDS5A013&FORMAT_OBJECT=TaxBlast&NCBI_GI=off&DESCRIPTIONS=500&ALIGNMENTS=250&FORMAT_BLOCK_ON_RESPAGE=Top&MASK_COLOR=1&MASK_CHAR=2#516466) [[b-proteobacteria](http://www.ncbi.nlm.nih.gov/Taxonomy/Browser/wwwtax.cgi?id=28216)] [nuclear protein SET [Burkholderia sp. H160] >gi|209503835|g](http://www.ncbi.nlm.nih.gov/entrez/query.fcgi?cmd=Retrieve&db=Protein&list_uids=496198113&dopt=GenPept)
410. . [Burkholderia sp. JPY251](http://www.ncbi.nlm.nih.gov/Taxonomy/Browser/wwwtax.cgi?id=667585) ....................................... 92 [1 hit](http://blast.ncbi.nlm.nih.gov/Blast.cgi?CMD=Get&RID=5Y3VDS5A013&FORMAT_OBJECT=TaxBlast&NCBI_GI=off&DESCRIPTIONS=500&ALIGNMENTS=250&FORMAT_BLOCK_ON_RESPAGE=Top&MASK_COLOR=1&MASK_CHAR=2#667585) [[b-proteobacteria](http://www.ncbi.nlm.nih.gov/Taxonomy/Browser/wwwtax.cgi?id=28216)] [nuclear protein SET [Burkholderia sp. JPY251]](http://www.ncbi.nlm.nih.gov/entrez/query.fcgi?cmd=Retrieve&db=Protein&list_uids=517243614&dopt=GenPept)
411. . [Bradyrhizobium sp. S23321](http://www.ncbi.nlm.nih.gov/Taxonomy/Browser/wwwtax.cgi?id=335659) ..................................... 92 [3 hits](http://blast.ncbi.nlm.nih.gov/Blast.cgi?CMD=Get&RID=5Y3VDS5A013&FORMAT_OBJECT=TaxBlast&NCBI_GI=off&DESCRIPTIONS=500&ALIGNMENTS=250&FORMAT_BLOCK_ON_RESPAGE=Top&MASK_COLOR=1&MASK_CHAR=2#335659) [[a-proteobacteria](http://www.ncbi.nlm.nih.gov/Taxonomy/Browser/wwwtax.cgi?id=28211)] [hypothetical protein S23_24500 [Bradyrhizobium sp. S23321]](http://www.ncbi.nlm.nih.gov/entrez/query.fcgi?cmd=Retrieve&db=Protein&list_uids=383770712&dopt=GenPept)
412. . [Burkholderia sp. WSM4176](http://www.ncbi.nlm.nih.gov/Taxonomy/Browser/wwwtax.cgi?id=935543) ...................................... 92 [1 hit](http://blast.ncbi.nlm.nih.gov/Blast.cgi?CMD=Get&RID=5Y3VDS5A013&FORMAT_OBJECT=TaxBlast&NCBI_GI=off&DESCRIPTIONS=500&ALIGNMENTS=250&FORMAT_BLOCK_ON_RESPAGE=Top&MASK_COLOR=1&MASK_CHAR=2#935543) [[b-proteobacteria](http://www.ncbi.nlm.nih.gov/Taxonomy/Browser/wwwtax.cgi?id=28216)] [nuclear protein SET [Burkholderia sp. WSM4176]](http://www.ncbi.nlm.nih.gov/entrez/query.fcgi?cmd=Retrieve&db=Protein&list_uids=517230064&dopt=GenPept)
413. . [Burkholderia vietnamiensis G4](http://www.ncbi.nlm.nih.gov/Taxonomy/Browser/wwwtax.cgi?id=269482) ................................. 92 [2 hits](http://blast.ncbi.nlm.nih.gov/Blast.cgi?CMD=Get&RID=5Y3VDS5A013&FORMAT_OBJECT=TaxBlast&NCBI_GI=off&DESCRIPTIONS=500&ALIGNMENTS=250&FORMAT_BLOCK_ON_RESPAGE=Top&MASK_COLOR=1&MASK_CHAR=2#269482) [[b-proteobacteria](http://www.ncbi.nlm.nih.gov/Taxonomy/Browser/wwwtax.cgi?id=28216)] [nuclear protein SET [Burkholderia vietnamiensis G4] >gi|500](http://www.ncbi.nlm.nih.gov/entrez/query.fcgi?cmd=Retrieve&db=Protein&list_uids=134294206&dopt=GenPept)
414. . [Burkholderia vietnamiensis](http://www.ncbi.nlm.nih.gov/Taxonomy/Browser/wwwtax.cgi?id=60552) .................................... 92 [1 hit](http://blast.ncbi.nlm.nih.gov/Blast.cgi?CMD=Get&RID=5Y3VDS5A013&FORMAT_OBJECT=TaxBlast&NCBI_GI=off&DESCRIPTIONS=500&ALIGNMENTS=250&FORMAT_BLOCK_ON_RESPAGE=Top&MASK_COLOR=1&MASK_CHAR=2#60552) [[b-proteobacteria](http://www.ncbi.nlm.nih.gov/Taxonomy/Browser/wwwtax.cgi?id=28216)] [nuclear protein SET [Burkholderia vietnamiensis G4] >gi|500](http://www.ncbi.nlm.nih.gov/entrez/query.fcgi?cmd=Retrieve&db=Protein&list_uids=500212607&dopt=GenPept)
415. . [Hydrocarboniphaga effusa](http://www.ncbi.nlm.nih.gov/Taxonomy/Browser/wwwtax.cgi?id=243629) ...................................... 91 [1 hit](http://blast.ncbi.nlm.nih.gov/Blast.cgi?CMD=Get&RID=5Y3VDS5A013&FORMAT_OBJECT=TaxBlast&NCBI_GI=off&DESCRIPTIONS=500&ALIGNMENTS=250&FORMAT_BLOCK_ON_RESPAGE=Top&MASK_COLOR=1&MASK_CHAR=2#243629) [[g-proteobacteria](http://www.ncbi.nlm.nih.gov/Taxonomy/Browser/wwwtax.cgi?id=1236)] [nuclear protein SET [Hydrocarboniphaga effusa] >gi|39185871](http://www.ncbi.nlm.nih.gov/entrez/query.fcgi?cmd=Retrieve&db=Protein&list_uids=494337898&dopt=GenPept)
416. . [Hydrocarboniphaga effusa AP103](http://www.ncbi.nlm.nih.gov/Taxonomy/Browser/wwwtax.cgi?id=1172194) ................................ 91 [1 hit](http://blast.ncbi.nlm.nih.gov/Blast.cgi?CMD=Get&RID=5Y3VDS5A013&FORMAT_OBJECT=TaxBlast&NCBI_GI=off&DESCRIPTIONS=500&ALIGNMENTS=250&FORMAT_BLOCK_ON_RESPAGE=Top&MASK_COLOR=1&MASK_CHAR=2#1172194) [[g-proteobacteria](http://www.ncbi.nlm.nih.gov/Taxonomy/Browser/wwwtax.cgi?id=1236)] [nuclear protein SET [Hydrocarboniphaga effusa] >gi|39185871](http://www.ncbi.nlm.nih.gov/entrez/query.fcgi?cmd=Retrieve&db=Protein&list_uids=391858712&dopt=GenPept)
417. . [Alcaligenes](http://www.ncbi.nlm.nih.gov/Taxonomy/Browser/wwwtax.cgi?id=507) ................................................... 93 [1 hit](http://blast.ncbi.nlm.nih.gov/Blast.cgi?CMD=Get&RID=5Y3VDS5A013&FORMAT_OBJECT=TaxBlast&NCBI_GI=off&DESCRIPTIONS=500&ALIGNMENTS=250&FORMAT_BLOCK_ON_RESPAGE=Top&MASK_COLOR=1&MASK_CHAR=2#507) [[b-proteobacteria](http://www.ncbi.nlm.nih.gov/Taxonomy/Browser/wwwtax.cgi?id=28216)] [hypothetical protein [Alcaligenes] >gi|422885845|gb|EKU2828](http://www.ncbi.nlm.nih.gov/entrez/query.fcgi?cmd=Retrieve&db=Protein&list_uids=497082313&dopt=GenPept)
418. . [Alcaligenes sp. HPC1271](http://www.ncbi.nlm.nih.gov/Taxonomy/Browser/wwwtax.cgi?id=1069631) ....................................... 93 [1 hit](http://blast.ncbi.nlm.nih.gov/Blast.cgi?CMD=Get&RID=5Y3VDS5A013&FORMAT_OBJECT=TaxBlast&NCBI_GI=off&DESCRIPTIONS=500&ALIGNMENTS=250&FORMAT_BLOCK_ON_RESPAGE=Top&MASK_COLOR=1&MASK_CHAR=2#1069631) [[b-proteobacteria](http://www.ncbi.nlm.nih.gov/Taxonomy/Browser/wwwtax.cgi?id=28216)] [hypothetical protein [Alcaligenes] >gi|422885845|gb|EKU2828](http://www.ncbi.nlm.nih.gov/entrez/query.fcgi?cmd=Retrieve&db=Protein&list_uids=422885845&dopt=GenPept)
419. . [Alcaligenes sp. EGD-AK7](http://www.ncbi.nlm.nih.gov/Taxonomy/Browser/wwwtax.cgi?id=1386079) ....................................... 93 [1 hit](http://blast.ncbi.nlm.nih.gov/Blast.cgi?CMD=Get&RID=5Y3VDS5A013&FORMAT_OBJECT=TaxBlast&NCBI_GI=off&DESCRIPTIONS=500&ALIGNMENTS=250&FORMAT_BLOCK_ON_RESPAGE=Top&MASK_COLOR=1&MASK_CHAR=2#1386079) [[b-proteobacteria](http://www.ncbi.nlm.nih.gov/Taxonomy/Browser/wwwtax.cgi?id=28216)] [hypothetical protein [Alcaligenes] >gi|422885845|gb|EKU2828](http://www.ncbi.nlm.nih.gov/entrez/query.fcgi?cmd=Retrieve&db=Protein&list_uids=542103716&dopt=GenPept)
420. . [Methanosarcina mazei Go1](http://www.ncbi.nlm.nih.gov/Taxonomy/Browser/wwwtax.cgi?id=192952) ...................................... 90 [2 hits](http://blast.ncbi.nlm.nih.gov/Blast.cgi?CMD=Get&RID=5Y3VDS5A013&FORMAT_OBJECT=TaxBlast&NCBI_GI=off&DESCRIPTIONS=500&ALIGNMENTS=250&FORMAT_BLOCK_ON_RESPAGE=Top&MASK_COLOR=1&MASK_CHAR=2#192952) [[euryarchaeotes](http://www.ncbi.nlm.nih.gov/Taxonomy/Browser/wwwtax.cgi?id=28890)] [hypothetical protein MM_2845 [Methanosarcina mazei Go1] >gi](http://www.ncbi.nlm.nih.gov/entrez/query.fcgi?cmd=Retrieve&db=Protein&list_uids=21228947&dopt=GenPept)
421. . [Methanosarcina mazei](http://www.ncbi.nlm.nih.gov/Taxonomy/Browser/wwwtax.cgi?id=2209) .......................................... 90 [1 hit](http://blast.ncbi.nlm.nih.gov/Blast.cgi?CMD=Get&RID=5Y3VDS5A013&FORMAT_OBJECT=TaxBlast&NCBI_GI=off&DESCRIPTIONS=500&ALIGNMENTS=250&FORMAT_BLOCK_ON_RESPAGE=Top&MASK_COLOR=1&MASK_CHAR=2#2209) [[euryarchaeotes](http://www.ncbi.nlm.nih.gov/Taxonomy/Browser/wwwtax.cgi?id=28890)] [hypothetical protein MM_2845 [Methanosarcina mazei Go1] >gi](http://www.ncbi.nlm.nih.gov/entrez/query.fcgi?cmd=Retrieve&db=Protein&list_uids=499345213&dopt=GenPept)
422. . [Variovorax paradoxus](http://www.ncbi.nlm.nih.gov/Taxonomy/Browser/wwwtax.cgi?id=34073) .......................................... 92 [2 hits](http://blast.ncbi.nlm.nih.gov/Blast.cgi?CMD=Get&RID=5Y3VDS5A013&FORMAT_OBJECT=TaxBlast&NCBI_GI=off&DESCRIPTIONS=500&ALIGNMENTS=250&FORMAT_BLOCK_ON_RESPAGE=Top&MASK_COLOR=1&MASK_CHAR=2#34073) [[b-proteobacteria](http://www.ncbi.nlm.nih.gov/Taxonomy/Browser/wwwtax.cgi?id=28216)] [lysine methyltransferase [Variovorax paradoxus]](http://www.ncbi.nlm.nih.gov/entrez/query.fcgi?cmd=Retrieve&db=Protein&list_uids=518483602&dopt=GenPept)
423. . [Pandoraea sp. B-6](http://www.ncbi.nlm.nih.gov/Taxonomy/Browser/wwwtax.cgi?id=1204340) ............................................. 91 [1 hit](http://blast.ncbi.nlm.nih.gov/Blast.cgi?CMD=Get&RID=5Y3VDS5A013&FORMAT_OBJECT=TaxBlast&NCBI_GI=off&DESCRIPTIONS=500&ALIGNMENTS=250&FORMAT_BLOCK_ON_RESPAGE=Top&MASK_COLOR=1&MASK_CHAR=2#1204340) [[b-proteobacteria](http://www.ncbi.nlm.nih.gov/Taxonomy/Browser/wwwtax.cgi?id=28216)] [hypothetical protein [Pandoraea sp. B-6]](http://www.ncbi.nlm.nih.gov/entrez/query.fcgi?cmd=Retrieve&db=Protein&list_uids=515803111&dopt=GenPept)
424. . [Xylella fastidiosa M12](http://www.ncbi.nlm.nih.gov/Taxonomy/Browser/wwwtax.cgi?id=405440) ........................................ 91 [2 hits](http://blast.ncbi.nlm.nih.gov/Blast.cgi?CMD=Get&RID=5Y3VDS5A013&FORMAT_OBJECT=TaxBlast&NCBI_GI=off&DESCRIPTIONS=500&ALIGNMENTS=250&FORMAT_BLOCK_ON_RESPAGE=Top&MASK_COLOR=1&MASK_CHAR=2#405440) [[g-proteobacteria](http://www.ncbi.nlm.nih.gov/Taxonomy/Browser/wwwtax.cgi?id=1236)] [hypothetical protein Xfasm12_0820 [Xylella fastidiosa M12]](http://www.ncbi.nlm.nih.gov/entrez/query.fcgi?cmd=Retrieve&db=Protein&list_uids=170730005&dopt=GenPept)
425. . [Xylella fastidiosa M23](http://www.ncbi.nlm.nih.gov/Taxonomy/Browser/wwwtax.cgi?id=405441) ........................................ 91 [2 hits](http://blast.ncbi.nlm.nih.gov/Blast.cgi?CMD=Get&RID=5Y3VDS5A013&FORMAT_OBJECT=TaxBlast&NCBI_GI=off&DESCRIPTIONS=500&ALIGNMENTS=250&FORMAT_BLOCK_ON_RESPAGE=Top&MASK_COLOR=1&MASK_CHAR=2#405441) [[g-proteobacteria](http://www.ncbi.nlm.nih.gov/Taxonomy/Browser/wwwtax.cgi?id=1236)] [hypothetical protein Xfasm12_0820 [Xylella fastidiosa M12]](http://www.ncbi.nlm.nih.gov/entrez/query.fcgi?cmd=Retrieve&db=Protein&list_uids=182681286&dopt=GenPept)
426. . [Xylella fastidiosa subsp. fastidiosa GB514](http://www.ncbi.nlm.nih.gov/Taxonomy/Browser/wwwtax.cgi?id=788929) .................... 91 [2 hits](http://blast.ncbi.nlm.nih.gov/Blast.cgi?CMD=Get&RID=5Y3VDS5A013&FORMAT_OBJECT=TaxBlast&NCBI_GI=off&DESCRIPTIONS=500&ALIGNMENTS=250&FORMAT_BLOCK_ON_RESPAGE=Top&MASK_COLOR=1&MASK_CHAR=2#788929) [[g-proteobacteria](http://www.ncbi.nlm.nih.gov/Taxonomy/Browser/wwwtax.cgi?id=1236)] [hypothetical protein Xfasm12_0820 [Xylella fastidiosa M12]](http://www.ncbi.nlm.nih.gov/entrez/query.fcgi?cmd=Retrieve&db=Protein&list_uids=386084790&dopt=GenPept)
427. . [Xylella fastidiosa](http://www.ncbi.nlm.nih.gov/Taxonomy/Browser/wwwtax.cgi?id=2371) ............................................ 91 [1 hit](http://blast.ncbi.nlm.nih.gov/Blast.cgi?CMD=Get&RID=5Y3VDS5A013&FORMAT_OBJECT=TaxBlast&NCBI_GI=off&DESCRIPTIONS=500&ALIGNMENTS=250&FORMAT_BLOCK_ON_RESPAGE=Top&MASK_COLOR=1&MASK_CHAR=2#2371) [[g-proteobacteria](http://www.ncbi.nlm.nih.gov/Taxonomy/Browser/wwwtax.cgi?id=1236)] [hypothetical protein Xfasm12_0820 [Xylella fastidiosa M12]](http://www.ncbi.nlm.nih.gov/entrez/query.fcgi?cmd=Retrieve&db=Protein&list_uids=490185112&dopt=GenPept)
428. . [Xylella fastidiosa Dixon](http://www.ncbi.nlm.nih.gov/Taxonomy/Browser/wwwtax.cgi?id=155919) ...................................... 91 [1 hit](http://blast.ncbi.nlm.nih.gov/Blast.cgi?CMD=Get&RID=5Y3VDS5A013&FORMAT_OBJECT=TaxBlast&NCBI_GI=off&DESCRIPTIONS=500&ALIGNMENTS=250&FORMAT_BLOCK_ON_RESPAGE=Top&MASK_COLOR=1&MASK_CHAR=2#155919) [[g-proteobacteria](http://www.ncbi.nlm.nih.gov/Taxonomy/Browser/wwwtax.cgi?id=1236)] [hypothetical protein Xfasm12_0820 [Xylella fastidiosa M12]](http://www.ncbi.nlm.nih.gov/entrez/query.fcgi?cmd=Retrieve&db=Protein&list_uids=71164485&dopt=GenPept)
429. . [Xylella fastidiosa subsp. sandyi Ann-1](http://www.ncbi.nlm.nih.gov/Taxonomy/Browser/wwwtax.cgi?id=155920) ........................ 91 [2 hits](http://blast.ncbi.nlm.nih.gov/Blast.cgi?CMD=Get&RID=5Y3VDS5A013&FORMAT_OBJECT=TaxBlast&NCBI_GI=off&DESCRIPTIONS=500&ALIGNMENTS=250&FORMAT_BLOCK_ON_RESPAGE=Top&MASK_COLOR=1&MASK_CHAR=2#155920) [[g-proteobacteria](http://www.ncbi.nlm.nih.gov/Taxonomy/Browser/wwwtax.cgi?id=1236)] [hypothetical protein Xfasm12_0820 [Xylella fastidiosa M12]](http://www.ncbi.nlm.nih.gov/entrez/query.fcgi?cmd=Retrieve&db=Protein&list_uids=71729035&dopt=GenPept)
430. . [Xylella fastidiosa EB92.1](http://www.ncbi.nlm.nih.gov/Taxonomy/Browser/wwwtax.cgi?id=945689) ..................................... 91 [1 hit](http://blast.ncbi.nlm.nih.gov/Blast.cgi?CMD=Get&RID=5Y3VDS5A013&FORMAT_OBJECT=TaxBlast&NCBI_GI=off&DESCRIPTIONS=500&ALIGNMENTS=250&FORMAT_BLOCK_ON_RESPAGE=Top&MASK_COLOR=1&MASK_CHAR=2#945689) [[g-proteobacteria](http://www.ncbi.nlm.nih.gov/Taxonomy/Browser/wwwtax.cgi?id=1236)] [hypothetical protein Xfasm12_0820 [Xylella fastidiosa M12]](http://www.ncbi.nlm.nih.gov/entrez/query.fcgi?cmd=Retrieve&db=Protein&list_uids=338179087&dopt=GenPept)
431. . [Xylella fastidiosa subsp. multiplex Griffin-1](http://www.ncbi.nlm.nih.gov/Taxonomy/Browser/wwwtax.cgi?id=1343737) ................. 91 [1 hit](http://blast.ncbi.nlm.nih.gov/Blast.cgi?CMD=Get&RID=5Y3VDS5A013&FORMAT_OBJECT=TaxBlast&NCBI_GI=off&DESCRIPTIONS=500&ALIGNMENTS=250&FORMAT_BLOCK_ON_RESPAGE=Top&MASK_COLOR=1&MASK_CHAR=2#1343737) [[g-proteobacteria](http://www.ncbi.nlm.nih.gov/Taxonomy/Browser/wwwtax.cgi?id=1236)] [hypothetical protein Xfasm12_0820 [Xylella fastidiosa M12]](http://www.ncbi.nlm.nih.gov/entrez/query.fcgi?cmd=Retrieve&db=Protein&list_uids=542424863&dopt=GenPept)
432. . [Limnohabitans sp. Rim47](http://www.ncbi.nlm.nih.gov/Taxonomy/Browser/wwwtax.cgi?id=1100721) ....................................... 92 [1 hit](http://blast.ncbi.nlm.nih.gov/Blast.cgi?CMD=Get&RID=5Y3VDS5A013&FORMAT_OBJECT=TaxBlast&NCBI_GI=off&DESCRIPTIONS=500&ALIGNMENTS=250&FORMAT_BLOCK_ON_RESPAGE=Top&MASK_COLOR=1&MASK_CHAR=2#1100721) [[b-proteobacteria](http://www.ncbi.nlm.nih.gov/Taxonomy/Browser/wwwtax.cgi?id=28216)] [hypothetical protein [Limnohabitans sp. Rim47]](http://www.ncbi.nlm.nih.gov/entrez/query.fcgi?cmd=Retrieve&db=Protein&list_uids=518259722&dopt=GenPept)
433. . [Rubrivivax benzoatilyticus](http://www.ncbi.nlm.nih.gov/Taxonomy/Browser/wwwtax.cgi?id=316997) .................................... 90 [1 hit](http://blast.ncbi.nlm.nih.gov/Blast.cgi?CMD=Get&RID=5Y3VDS5A013&FORMAT_OBJECT=TaxBlast&NCBI_GI=off&DESCRIPTIONS=500&ALIGNMENTS=250&FORMAT_BLOCK_ON_RESPAGE=Top&MASK_COLOR=1&MASK_CHAR=2#316997) [[b-proteobacteria](http://www.ncbi.nlm.nih.gov/Taxonomy/Browser/wwwtax.cgi?id=28216)] [hypothetical protein [Rubrivivax benzoatilyticus] >gi|33210](http://www.ncbi.nlm.nih.gov/entrez/query.fcgi?cmd=Retrieve&db=Protein&list_uids=497541560&dopt=GenPept)
434. . [Rubrivivax benzoatilyticus JA2 = ATCC BAA-35](http://www.ncbi.nlm.nih.gov/Taxonomy/Browser/wwwtax.cgi?id=987059) .................. 90 [1 hit](http://blast.ncbi.nlm.nih.gov/Blast.cgi?CMD=Get&RID=5Y3VDS5A013&FORMAT_OBJECT=TaxBlast&NCBI_GI=off&DESCRIPTIONS=500&ALIGNMENTS=250&FORMAT_BLOCK_ON_RESPAGE=Top&MASK_COLOR=1&MASK_CHAR=2#987059) [[b-proteobacteria](http://www.ncbi.nlm.nih.gov/Taxonomy/Browser/wwwtax.cgi?id=28216)] [hypothetical protein [Rubrivivax benzoatilyticus] >gi|33210](http://www.ncbi.nlm.nih.gov/entrez/query.fcgi?cmd=Retrieve&db=Protein&list_uids=332107967&dopt=GenPept)
435. . [Alcaligenes faecalis](http://www.ncbi.nlm.nih.gov/Taxonomy/Browser/wwwtax.cgi?id=511) .......................................... 93 [1 hit](http://blast.ncbi.nlm.nih.gov/Blast.cgi?CMD=Get&RID=5Y3VDS5A013&FORMAT_OBJECT=TaxBlast&NCBI_GI=off&DESCRIPTIONS=500&ALIGNMENTS=250&FORMAT_BLOCK_ON_RESPAGE=Top&MASK_COLOR=1&MASK_CHAR=2#511) [[b-proteobacteria](http://www.ncbi.nlm.nih.gov/Taxonomy/Browser/wwwtax.cgi?id=28216)] [hypothetical protein [Alcaligenes faecalis] >gi|393165015|g](http://www.ncbi.nlm.nih.gov/entrez/query.fcgi?cmd=Retrieve&db=Protein&list_uids=489897132&dopt=GenPept)
436. . [Alcaligenes faecalis subsp. faecalis NCIB 8687](http://www.ncbi.nlm.nih.gov/Taxonomy/Browser/wwwtax.cgi?id=1156918) ................ 93 [1 hit](http://blast.ncbi.nlm.nih.gov/Blast.cgi?CMD=Get&RID=5Y3VDS5A013&FORMAT_OBJECT=TaxBlast&NCBI_GI=off&DESCRIPTIONS=500&ALIGNMENTS=250&FORMAT_BLOCK_ON_RESPAGE=Top&MASK_COLOR=1&MASK_CHAR=2#1156918) [[b-proteobacteria](http://www.ncbi.nlm.nih.gov/Taxonomy/Browser/wwwtax.cgi?id=28216)] [hypothetical protein [Alcaligenes faecalis] >gi|393165015|g](http://www.ncbi.nlm.nih.gov/entrez/query.fcgi?cmd=Retrieve&db=Protein&list_uids=393165015&dopt=GenPept)
437. . [Acidovorax avenae subsp. avenae ATCC 19860](http://www.ncbi.nlm.nih.gov/Taxonomy/Browser/wwwtax.cgi?id=643561) .................... 91 [2 hits](http://blast.ncbi.nlm.nih.gov/Blast.cgi?CMD=Get&RID=5Y3VDS5A013&FORMAT_OBJECT=TaxBlast&NCBI_GI=off&DESCRIPTIONS=500&ALIGNMENTS=250&FORMAT_BLOCK_ON_RESPAGE=Top&MASK_COLOR=1&MASK_CHAR=2#643561) [[b-proteobacteria](http://www.ncbi.nlm.nih.gov/Taxonomy/Browser/wwwtax.cgi?id=28216)] [nuclear protein SET [Acidovorax avenae subsp. avenae ATCC 1](http://www.ncbi.nlm.nih.gov/entrez/query.fcgi?cmd=Retrieve&db=Protein&list_uids=326319334&dopt=GenPept)
438. . [Acidovorax avenae](http://www.ncbi.nlm.nih.gov/Taxonomy/Browser/wwwtax.cgi?id=80867) ............................................. 91 [2 hits](http://blast.ncbi.nlm.nih.gov/Blast.cgi?CMD=Get&RID=5Y3VDS5A013&FORMAT_OBJECT=TaxBlast&NCBI_GI=off&DESCRIPTIONS=500&ALIGNMENTS=250&FORMAT_BLOCK_ON_RESPAGE=Top&MASK_COLOR=1&MASK_CHAR=2#80867) [[b-proteobacteria](http://www.ncbi.nlm.nih.gov/Taxonomy/Browser/wwwtax.cgi?id=28216)] [nuclear protein SET [Acidovorax avenae subsp. avenae ATCC 1](http://www.ncbi.nlm.nih.gov/entrez/query.fcgi?cmd=Retrieve&db=Protein&list_uids=503362242&dopt=GenPept)
439. . [Burkholderia sp. KJ006](http://www.ncbi.nlm.nih.gov/Taxonomy/Browser/wwwtax.cgi?id=416344) ........................................ 91 [3 hits](http://blast.ncbi.nlm.nih.gov/Blast.cgi?CMD=Get&RID=5Y3VDS5A013&FORMAT_OBJECT=TaxBlast&NCBI_GI=off&DESCRIPTIONS=500&ALIGNMENTS=250&FORMAT_BLOCK_ON_RESPAGE=Top&MASK_COLOR=1&MASK_CHAR=2#416344) [[b-proteobacteria](http://www.ncbi.nlm.nih.gov/Taxonomy/Browser/wwwtax.cgi?id=28216)] [Zinc finger protein HRX [Burkholderia sp. KJ006] >gi|504535](http://www.ncbi.nlm.nih.gov/entrez/query.fcgi?cmd=Retrieve&db=Protein&list_uids=387900829&dopt=GenPept)
440. . [blood disease bacterium R229](http://www.ncbi.nlm.nih.gov/Taxonomy/Browser/wwwtax.cgi?id=741978) .................................. 91 [1 hit](http://blast.ncbi.nlm.nih.gov/Blast.cgi?CMD=Get&RID=5Y3VDS5A013&FORMAT_OBJECT=TaxBlast&NCBI_GI=off&DESCRIPTIONS=500&ALIGNMENTS=250&FORMAT_BLOCK_ON_RESPAGE=Top&MASK_COLOR=1&MASK_CHAR=2#741978) [[b-proteobacteria](http://www.ncbi.nlm.nih.gov/Taxonomy/Browser/wwwtax.cgi?id=28216)] [putative histone-lysine N-methyltransferase fragment [blood](http://www.ncbi.nlm.nih.gov/entrez/query.fcgi?cmd=Retrieve&db=Protein&list_uids=344168416&dopt=GenPept)
441. . [Caldimonas manganoxidans](http://www.ncbi.nlm.nih.gov/Taxonomy/Browser/wwwtax.cgi?id=196015) ...................................... 90 [1 hit](http://blast.ncbi.nlm.nih.gov/Blast.cgi?CMD=Get&RID=5Y3VDS5A013&FORMAT_OBJECT=TaxBlast&NCBI_GI=off&DESCRIPTIONS=500&ALIGNMENTS=250&FORMAT_BLOCK_ON_RESPAGE=Top&MASK_COLOR=1&MASK_CHAR=2#196015) [[b-proteobacteria](http://www.ncbi.nlm.nih.gov/Taxonomy/Browser/wwwtax.cgi?id=28216)] [nuclear protein SET [Caldimonas manganoxidans]](http://www.ncbi.nlm.nih.gov/entrez/query.fcgi?cmd=Retrieve&db=Protein&list_uids=518391330&dopt=GenPept)
442. . [Xanthomonas arboricola](http://www.ncbi.nlm.nih.gov/Taxonomy/Browser/wwwtax.cgi?id=56448) ........................................ 90 [1 hit](http://blast.ncbi.nlm.nih.gov/Blast.cgi?CMD=Get&RID=5Y3VDS5A013&FORMAT_OBJECT=TaxBlast&NCBI_GI=off&DESCRIPTIONS=500&ALIGNMENTS=250&FORMAT_BLOCK_ON_RESPAGE=Top&MASK_COLOR=1&MASK_CHAR=2#56448) [[g-proteobacteria](http://www.ncbi.nlm.nih.gov/Taxonomy/Browser/wwwtax.cgi?id=1236)] [nuclear protein SET [Xanthomonas arboricola]](http://www.ncbi.nlm.nih.gov/entrez/query.fcgi?cmd=Retrieve&db=Protein&list_uids=515421959&dopt=GenPept)
443. . [Rudaea cellulosilytica](http://www.ncbi.nlm.nih.gov/Taxonomy/Browser/wwwtax.cgi?id=540746) ........................................ 91 [1 hit](http://blast.ncbi.nlm.nih.gov/Blast.cgi?CMD=Get&RID=5Y3VDS5A013&FORMAT_OBJECT=TaxBlast&NCBI_GI=off&DESCRIPTIONS=500&ALIGNMENTS=250&FORMAT_BLOCK_ON_RESPAGE=Top&MASK_COLOR=1&MASK_CHAR=2#540746) [[g-proteobacteria](http://www.ncbi.nlm.nih.gov/Taxonomy/Browser/wwwtax.cgi?id=1236)] [hypothetical protein [Rudaea cellulosilytica]](http://www.ncbi.nlm.nih.gov/entrez/query.fcgi?cmd=Retrieve&db=Protein&list_uids=522175539&dopt=GenPept)
444. . [Burkholderia dolosa](http://www.ncbi.nlm.nih.gov/Taxonomy/Browser/wwwtax.cgi?id=152500) ........................................... 92 [1 hit](http://blast.ncbi.nlm.nih.gov/Blast.cgi?CMD=Get&RID=5Y3VDS5A013&FORMAT_OBJECT=TaxBlast&NCBI_GI=off&DESCRIPTIONS=500&ALIGNMENTS=250&FORMAT_BLOCK_ON_RESPAGE=Top&MASK_COLOR=1&MASK_CHAR=2#152500) [[b-proteobacteria](http://www.ncbi.nlm.nih.gov/Taxonomy/Browser/wwwtax.cgi?id=28216)] [lysine methyltransferase [Burkholderia dolosa] >gi|12489361](http://www.ncbi.nlm.nih.gov/entrez/query.fcgi?cmd=Retrieve&db=Protein&list_uids=493815398&dopt=GenPept)
445. . [Burkholderia dolosa AUO158](http://www.ncbi.nlm.nih.gov/Taxonomy/Browser/wwwtax.cgi?id=350701) .................................... 92 [1 hit](http://blast.ncbi.nlm.nih.gov/Blast.cgi?CMD=Get&RID=5Y3VDS5A013&FORMAT_OBJECT=TaxBlast&NCBI_GI=off&DESCRIPTIONS=500&ALIGNMENTS=250&FORMAT_BLOCK_ON_RESPAGE=Top&MASK_COLOR=1&MASK_CHAR=2#350701) [[b-proteobacteria](http://www.ncbi.nlm.nih.gov/Taxonomy/Browser/wwwtax.cgi?id=28216)] [lysine methyltransferase [Burkholderia dolosa] >gi|12489361](http://www.ncbi.nlm.nih.gov/entrez/query.fcgi?cmd=Retrieve&db=Protein&list_uids=124893612&dopt=GenPept)
446. . [Burkholderia cenocepacia](http://www.ncbi.nlm.nih.gov/Taxonomy/Browser/wwwtax.cgi?id=95486) ...................................... 91 [3 hits](http://blast.ncbi.nlm.nih.gov/Blast.cgi?CMD=Get&RID=5Y3VDS5A013&FORMAT_OBJECT=TaxBlast&NCBI_GI=off&DESCRIPTIONS=500&ALIGNMENTS=250&FORMAT_BLOCK_ON_RESPAGE=Top&MASK_COLOR=1&MASK_CHAR=2#95486) [[b-proteobacteria](http://www.ncbi.nlm.nih.gov/Taxonomy/Browser/wwwtax.cgi?id=28216)] [SET domain protein [Burkholderia cenocepacia] >gi|529215269](http://www.ncbi.nlm.nih.gov/entrez/query.fcgi?cmd=Retrieve&db=Protein&list_uids=537729383&dopt=GenPept)
447. . [Burkholderia cenocepacia K56-2Valvano](http://www.ncbi.nlm.nih.gov/Taxonomy/Browser/wwwtax.cgi?id=985076) ......................... 91 [1 hit](http://blast.ncbi.nlm.nih.gov/Blast.cgi?CMD=Get&RID=5Y3VDS5A013&FORMAT_OBJECT=TaxBlast&NCBI_GI=off&DESCRIPTIONS=500&ALIGNMENTS=250&FORMAT_BLOCK_ON_RESPAGE=Top&MASK_COLOR=1&MASK_CHAR=2#985076) [[b-proteobacteria](http://www.ncbi.nlm.nih.gov/Taxonomy/Browser/wwwtax.cgi?id=28216)] [SET domain protein [Burkholderia cenocepacia] >gi|529215269](http://www.ncbi.nlm.nih.gov/entrez/query.fcgi?cmd=Retrieve&db=Protein&list_uids=529215269&dopt=GenPept)
448. . [Burkholderia cenocepacia BC7](http://www.ncbi.nlm.nih.gov/Taxonomy/Browser/wwwtax.cgi?id=985077) .................................. 91 [1 hit](http://blast.ncbi.nlm.nih.gov/Blast.cgi?CMD=Get&RID=5Y3VDS5A013&FORMAT_OBJECT=TaxBlast&NCBI_GI=off&DESCRIPTIONS=500&ALIGNMENTS=250&FORMAT_BLOCK_ON_RESPAGE=Top&MASK_COLOR=1&MASK_CHAR=2#985077) [[b-proteobacteria](http://www.ncbi.nlm.nih.gov/Taxonomy/Browser/wwwtax.cgi?id=28216)] [SET domain protein [Burkholderia cenocepacia] >gi|529215269](http://www.ncbi.nlm.nih.gov/entrez/query.fcgi?cmd=Retrieve&db=Protein&list_uids=542088159&dopt=GenPept)
449. . [Herminiimonas arsenicoxydans](http://www.ncbi.nlm.nih.gov/Taxonomy/Browser/wwwtax.cgi?id=204773) .................................. 90 [3 hits](http://blast.ncbi.nlm.nih.gov/Blast.cgi?CMD=Get&RID=5Y3VDS5A013&FORMAT_OBJECT=TaxBlast&NCBI_GI=off&DESCRIPTIONS=500&ALIGNMENTS=250&FORMAT_BLOCK_ON_RESPAGE=Top&MASK_COLOR=1&MASK_CHAR=2#204773) [[b-proteobacteria](http://www.ncbi.nlm.nih.gov/Taxonomy/Browser/wwwtax.cgi?id=28216)] [histone-lysine N-methyltransferase [Herminiimonas arsenicox](http://www.ncbi.nlm.nih.gov/entrez/query.fcgi?cmd=Retrieve&db=Protein&list_uids=134093363&dopt=GenPept)
450. . [Burkholderia cepacia GG4](http://www.ncbi.nlm.nih.gov/Taxonomy/Browser/wwwtax.cgi?id=1009846) ...................................... 90 [2 hits](http://blast.ncbi.nlm.nih.gov/Blast.cgi?CMD=Get&RID=5Y3VDS5A013&FORMAT_OBJECT=TaxBlast&NCBI_GI=off&DESCRIPTIONS=500&ALIGNMENTS=250&FORMAT_BLOCK_ON_RESPAGE=Top&MASK_COLOR=1&MASK_CHAR=2#1009846) [[b-proteobacteria](http://www.ncbi.nlm.nih.gov/Taxonomy/Browser/wwwtax.cgi?id=28216)] [nuclear protein SET [Burkholderia cepacia GG4] >gi|50470837](http://www.ncbi.nlm.nih.gov/entrez/query.fcgi?cmd=Retrieve&db=Protein&list_uids=402564878&dopt=GenPept)
451. . [Burkholderia cepacia](http://www.ncbi.nlm.nih.gov/Taxonomy/Browser/wwwtax.cgi?id=292) .......................................... 90 [1 hit](http://blast.ncbi.nlm.nih.gov/Blast.cgi?CMD=Get&RID=5Y3VDS5A013&FORMAT_OBJECT=TaxBlast&NCBI_GI=off&DESCRIPTIONS=500&ALIGNMENTS=250&FORMAT_BLOCK_ON_RESPAGE=Top&MASK_COLOR=1&MASK_CHAR=2#292) [[b-proteobacteria](http://www.ncbi.nlm.nih.gov/Taxonomy/Browser/wwwtax.cgi?id=28216)] [nuclear protein SET [Burkholderia cepacia GG4] >gi|50470837](http://www.ncbi.nlm.nih.gov/entrez/query.fcgi?cmd=Retrieve&db=Protein&list_uids=504708370&dopt=GenPept)
452. . [Chlorobium ferrooxidans](http://www.ncbi.nlm.nih.gov/Taxonomy/Browser/wwwtax.cgi?id=84205) ....................................... 89 [1 hit](http://blast.ncbi.nlm.nih.gov/Blast.cgi?CMD=Get&RID=5Y3VDS5A013&FORMAT_OBJECT=TaxBlast&NCBI_GI=off&DESCRIPTIONS=500&ALIGNMENTS=250&FORMAT_BLOCK_ON_RESPAGE=Top&MASK_COLOR=1&MASK_CHAR=2#84205) [[green sulfur bacteria](http://www.ncbi.nlm.nih.gov/Taxonomy/Browser/wwwtax.cgi?id=1090)] [lysine methyltransferase [Chlorobium ferrooxidans] >gi|1103](http://www.ncbi.nlm.nih.gov/entrez/query.fcgi?cmd=Retrieve&db=Protein&list_uids=493410864&dopt=GenPept)
453. . [Chlorobium ferrooxidans DSM 13031](http://www.ncbi.nlm.nih.gov/Taxonomy/Browser/wwwtax.cgi?id=377431) ............................. 89 [1 hit](http://blast.ncbi.nlm.nih.gov/Blast.cgi?CMD=Get&RID=5Y3VDS5A013&FORMAT_OBJECT=TaxBlast&NCBI_GI=off&DESCRIPTIONS=500&ALIGNMENTS=250&FORMAT_BLOCK_ON_RESPAGE=Top&MASK_COLOR=1&MASK_CHAR=2#377431) [[green sulfur bacteria](http://www.ncbi.nlm.nih.gov/Taxonomy/Browser/wwwtax.cgi?id=1090)] [lysine methyltransferase [Chlorobium ferrooxidans] >gi|1103](http://www.ncbi.nlm.nih.gov/entrez/query.fcgi?cmd=Retrieve&db=Protein&list_uids=110340060&dopt=GenPept)
454. . [Corallococcus coralloides DSM 2259](http://www.ncbi.nlm.nih.gov/Taxonomy/Browser/wwwtax.cgi?id=1144275) ............................ 92 [2 hits](http://blast.ncbi.nlm.nih.gov/Blast.cgi?CMD=Get&RID=5Y3VDS5A013&FORMAT_OBJECT=TaxBlast&NCBI_GI=off&DESCRIPTIONS=500&ALIGNMENTS=250&FORMAT_BLOCK_ON_RESPAGE=Top&MASK_COLOR=1&MASK_CHAR=2#1144275) [[d-proteobacteria](http://www.ncbi.nlm.nih.gov/Taxonomy/Browser/wwwtax.cgi?id=28221)] [SET domain-containing protein [Corallococcus coralloides DS](http://www.ncbi.nlm.nih.gov/entrez/query.fcgi?cmd=Retrieve&db=Protein&list_uids=383457656&dopt=GenPept)
455. . [Corallococcus coralloides](http://www.ncbi.nlm.nih.gov/Taxonomy/Browser/wwwtax.cgi?id=184914) ..................................... 92 [1 hit](http://blast.ncbi.nlm.nih.gov/Blast.cgi?CMD=Get&RID=5Y3VDS5A013&FORMAT_OBJECT=TaxBlast&NCBI_GI=off&DESCRIPTIONS=500&ALIGNMENTS=250&FORMAT_BLOCK_ON_RESPAGE=Top&MASK_COLOR=1&MASK_CHAR=2#184914) [[d-proteobacteria](http://www.ncbi.nlm.nih.gov/Taxonomy/Browser/wwwtax.cgi?id=28221)] [SET domain-containing protein [Corallococcus coralloides DS](http://www.ncbi.nlm.nih.gov/entrez/query.fcgi?cmd=Retrieve&db=Protein&list_uids=504211378&dopt=GenPept)
456. . [Acidovorax citrulli AAC00-1](http://www.ncbi.nlm.nih.gov/Taxonomy/Browser/wwwtax.cgi?id=397945) ................................... 91 [2 hits](http://blast.ncbi.nlm.nih.gov/Blast.cgi?CMD=Get&RID=5Y3VDS5A013&FORMAT_OBJECT=TaxBlast&NCBI_GI=off&DESCRIPTIONS=500&ALIGNMENTS=250&FORMAT_BLOCK_ON_RESPAGE=Top&MASK_COLOR=1&MASK_CHAR=2#397945) [[b-proteobacteria](http://www.ncbi.nlm.nih.gov/Taxonomy/Browser/wwwtax.cgi?id=28216)] [nuclear protein SET [Acidovorax citrulli AAC00-1] >gi|50012](http://www.ncbi.nlm.nih.gov/entrez/query.fcgi?cmd=Retrieve&db=Protein&list_uids=120613259&dopt=GenPept)
457. . [Acidovorax citrulli](http://www.ncbi.nlm.nih.gov/Taxonomy/Browser/wwwtax.cgi?id=80869) ........................................... 91 [1 hit](http://blast.ncbi.nlm.nih.gov/Blast.cgi?CMD=Get&RID=5Y3VDS5A013&FORMAT_OBJECT=TaxBlast&NCBI_GI=off&DESCRIPTIONS=500&ALIGNMENTS=250&FORMAT_BLOCK_ON_RESPAGE=Top&MASK_COLOR=1&MASK_CHAR=2#80869) [[b-proteobacteria](http://www.ncbi.nlm.nih.gov/Taxonomy/Browser/wwwtax.cgi?id=28216)] [nuclear protein SET [Acidovorax citrulli AAC00-1] >gi|50012](http://www.ncbi.nlm.nih.gov/entrez/query.fcgi?cmd=Retrieve&db=Protein&list_uids=500121624&dopt=GenPept)
458. . [Burkholderia glumae BGR1](http://www.ncbi.nlm.nih.gov/Taxonomy/Browser/wwwtax.cgi?id=626418) ...................................... 91 [2 hits](http://blast.ncbi.nlm.nih.gov/Blast.cgi?CMD=Get&RID=5Y3VDS5A013&FORMAT_OBJECT=TaxBlast&NCBI_GI=off&DESCRIPTIONS=500&ALIGNMENTS=250&FORMAT_BLOCK_ON_RESPAGE=Top&MASK_COLOR=1&MASK_CHAR=2#626418) [[b-proteobacteria](http://www.ncbi.nlm.nih.gov/Taxonomy/Browser/wwwtax.cgi?id=28216)] [SET domain-containing protein [Burkholderia glumae BGR1] >g](http://www.ncbi.nlm.nih.gov/entrez/query.fcgi?cmd=Retrieve&db=Protein&list_uids=238025726&dopt=GenPept)
459. . [Burkholderia glumae](http://www.ncbi.nlm.nih.gov/Taxonomy/Browser/wwwtax.cgi?id=337) ........................................... 91 [1 hit](http://blast.ncbi.nlm.nih.gov/Blast.cgi?CMD=Get&RID=5Y3VDS5A013&FORMAT_OBJECT=TaxBlast&NCBI_GI=off&DESCRIPTIONS=500&ALIGNMENTS=250&FORMAT_BLOCK_ON_RESPAGE=Top&MASK_COLOR=1&MASK_CHAR=2#337) [[b-proteobacteria](http://www.ncbi.nlm.nih.gov/Taxonomy/Browser/wwwtax.cgi?id=28216)] [SET domain-containing protein [Burkholderia glumae BGR1] >g](http://www.ncbi.nlm.nih.gov/entrez/query.fcgi?cmd=Retrieve&db=Protein&list_uids=502212962&dopt=GenPept)
460. . [Simkania negevensis Z](http://www.ncbi.nlm.nih.gov/Taxonomy/Browser/wwwtax.cgi?id=331113) ......................................... 92 [2 hits](http://blast.ncbi.nlm.nih.gov/Blast.cgi?CMD=Get&RID=5Y3VDS5A013&FORMAT_OBJECT=TaxBlast&NCBI_GI=off&DESCRIPTIONS=500&ALIGNMENTS=250&FORMAT_BLOCK_ON_RESPAGE=Top&MASK_COLOR=1&MASK_CHAR=2#331113) [[chlamydias](http://www.ncbi.nlm.nih.gov/Taxonomy/Browser/wwwtax.cgi?id=51291)] [seT domain-containing protein [Simkania negevensis Z] >gi|5](http://www.ncbi.nlm.nih.gov/entrez/query.fcgi?cmd=Retrieve&db=Protein&list_uids=338733099&dopt=GenPept)
461. . [Simkania negevensis](http://www.ncbi.nlm.nih.gov/Taxonomy/Browser/wwwtax.cgi?id=83561) ........................................... 92 [1 hit](http://blast.ncbi.nlm.nih.gov/Blast.cgi?CMD=Get&RID=5Y3VDS5A013&FORMAT_OBJECT=TaxBlast&NCBI_GI=off&DESCRIPTIONS=500&ALIGNMENTS=250&FORMAT_BLOCK_ON_RESPAGE=Top&MASK_COLOR=1&MASK_CHAR=2#83561) [[chlamydias](http://www.ncbi.nlm.nih.gov/Taxonomy/Browser/wwwtax.cgi?id=51291)] [seT domain-containing protein [Simkania negevensis Z] >gi|5](http://www.ncbi.nlm.nih.gov/entrez/query.fcgi?cmd=Retrieve&db=Protein&list_uids=503709472&dopt=GenPept)
462. . [Burkholderia phenoliruptrix BR3459a](http://www.ncbi.nlm.nih.gov/Taxonomy/Browser/wwwtax.cgi?id=1229205) ........................... 90 [2 hits](http://blast.ncbi.nlm.nih.gov/Blast.cgi?CMD=Get&RID=5Y3VDS5A013&FORMAT_OBJECT=TaxBlast&NCBI_GI=off&DESCRIPTIONS=500&ALIGNMENTS=250&FORMAT_BLOCK_ON_RESPAGE=Top&MASK_COLOR=1&MASK_CHAR=2#1229205) [[b-proteobacteria](http://www.ncbi.nlm.nih.gov/Taxonomy/Browser/wwwtax.cgi?id=28216)] [nuclear protein SET [Burkholderia phenoliruptrix BR3459a] >](http://www.ncbi.nlm.nih.gov/entrez/query.fcgi?cmd=Retrieve&db=Protein&list_uids=407715215&dopt=GenPept)
463. . [Burkholderia phenoliruptrix](http://www.ncbi.nlm.nih.gov/Taxonomy/Browser/wwwtax.cgi?id=252970) ................................... 90 [1 hit](http://blast.ncbi.nlm.nih.gov/Blast.cgi?CMD=Get&RID=5Y3VDS5A013&FORMAT_OBJECT=TaxBlast&NCBI_GI=off&DESCRIPTIONS=500&ALIGNMENTS=250&FORMAT_BLOCK_ON_RESPAGE=Top&MASK_COLOR=1&MASK_CHAR=2#252970) [[b-proteobacteria](http://www.ncbi.nlm.nih.gov/Taxonomy/Browser/wwwtax.cgi?id=28216)] [nuclear protein SET [Burkholderia phenoliruptrix BR3459a] >](http://www.ncbi.nlm.nih.gov/entrez/query.fcgi?cmd=Retrieve&db=Protein&list_uids=504817000&dopt=GenPept)
464. . [Opitutus terrae PB90-1](http://www.ncbi.nlm.nih.gov/Taxonomy/Browser/wwwtax.cgi?id=452637) ........................................ 91 [2 hits](http://blast.ncbi.nlm.nih.gov/Blast.cgi?CMD=Get&RID=5Y3VDS5A013&FORMAT_OBJECT=TaxBlast&NCBI_GI=off&DESCRIPTIONS=500&ALIGNMENTS=250&FORMAT_BLOCK_ON_RESPAGE=Top&MASK_COLOR=1&MASK_CHAR=2#452637) [[verrucomicrobia](http://www.ncbi.nlm.nih.gov/Taxonomy/Browser/wwwtax.cgi?id=74201)] [nuclear protein SET [Opitutus terrae PB90-1] >gi|501345678|](http://www.ncbi.nlm.nih.gov/entrez/query.fcgi?cmd=Retrieve&db=Protein&list_uids=182416333&dopt=GenPept)
465. . [Opitutus terrae](http://www.ncbi.nlm.nih.gov/Taxonomy/Browser/wwwtax.cgi?id=107709) ............................................... 91 [1 hit](http://blast.ncbi.nlm.nih.gov/Blast.cgi?CMD=Get&RID=5Y3VDS5A013&FORMAT_OBJECT=TaxBlast&NCBI_GI=off&DESCRIPTIONS=500&ALIGNMENTS=250&FORMAT_BLOCK_ON_RESPAGE=Top&MASK_COLOR=1&MASK_CHAR=2#107709) [[verrucomicrobia](http://www.ncbi.nlm.nih.gov/Taxonomy/Browser/wwwtax.cgi?id=74201)] [nuclear protein SET [Opitutus terrae PB90-1] >gi|501345678|](http://www.ncbi.nlm.nih.gov/entrez/query.fcgi?cmd=Retrieve&db=Protein&list_uids=501345678&dopt=GenPept)
466. . [Burkholderia cenocepacia J2315](http://www.ncbi.nlm.nih.gov/Taxonomy/Browser/wwwtax.cgi?id=216591) ................................ 90 [2 hits](http://blast.ncbi.nlm.nih.gov/Blast.cgi?CMD=Get&RID=5Y3VDS5A013&FORMAT_OBJECT=TaxBlast&NCBI_GI=off&DESCRIPTIONS=500&ALIGNMENTS=250&FORMAT_BLOCK_ON_RESPAGE=Top&MASK_COLOR=1&MASK_CHAR=2#216591) [[b-proteobacteria](http://www.ncbi.nlm.nih.gov/Taxonomy/Browser/wwwtax.cgi?id=28216)] [putative protein lysine methyltransferase protein [Burkhold](http://www.ncbi.nlm.nih.gov/entrez/query.fcgi?cmd=Retrieve&db=Protein&list_uids=206558409&dopt=GenPept)
467. . [Burkholderia cenocepacia H111](http://www.ncbi.nlm.nih.gov/Taxonomy/Browser/wwwtax.cgi?id=1055524) ................................. 90 [1 hit](http://blast.ncbi.nlm.nih.gov/Blast.cgi?CMD=Get&RID=5Y3VDS5A013&FORMAT_OBJECT=TaxBlast&NCBI_GI=off&DESCRIPTIONS=500&ALIGNMENTS=250&FORMAT_BLOCK_ON_RESPAGE=Top&MASK_COLOR=1&MASK_CHAR=2#1055524) [[b-proteobacteria](http://www.ncbi.nlm.nih.gov/Taxonomy/Browser/wwwtax.cgi?id=28216)] [putative protein lysine methyltransferase protein [Burkhold](http://www.ncbi.nlm.nih.gov/entrez/query.fcgi?cmd=Retrieve&db=Protein&list_uids=358073314&dopt=GenPept)
468. . [Leptothrix cholodnii SP-6](http://www.ncbi.nlm.nih.gov/Taxonomy/Browser/wwwtax.cgi?id=395495) ..................................... 90 [2 hits](http://blast.ncbi.nlm.nih.gov/Blast.cgi?CMD=Get&RID=5Y3VDS5A013&FORMAT_OBJECT=TaxBlast&NCBI_GI=off&DESCRIPTIONS=500&ALIGNMENTS=250&FORMAT_BLOCK_ON_RESPAGE=Top&MASK_COLOR=1&MASK_CHAR=2#395495) [[b-proteobacteria](http://www.ncbi.nlm.nih.gov/Taxonomy/Browser/wwwtax.cgi?id=28216)] [nuclear protein SET [Leptothrix cholodnii SP-6] >gi|5013136](http://www.ncbi.nlm.nih.gov/entrez/query.fcgi?cmd=Retrieve&db=Protein&list_uids=171056919&dopt=GenPept)
469. . [Leptothrix cholodnii](http://www.ncbi.nlm.nih.gov/Taxonomy/Browser/wwwtax.cgi?id=34029) .......................................... 90 [1 hit](http://blast.ncbi.nlm.nih.gov/Blast.cgi?CMD=Get&RID=5Y3VDS5A013&FORMAT_OBJECT=TaxBlast&NCBI_GI=off&DESCRIPTIONS=500&ALIGNMENTS=250&FORMAT_BLOCK_ON_RESPAGE=Top&MASK_COLOR=1&MASK_CHAR=2#34029) [[b-proteobacteria](http://www.ncbi.nlm.nih.gov/Taxonomy/Browser/wwwtax.cgi?id=28216)] [nuclear protein SET [Leptothrix cholodnii SP-6] >gi|5013136](http://www.ncbi.nlm.nih.gov/entrez/query.fcgi?cmd=Retrieve&db=Protein&list_uids=501313630&dopt=GenPept)
470. . [Xanthomonas gardneri](http://www.ncbi.nlm.nih.gov/Taxonomy/Browser/wwwtax.cgi?id=90270) .......................................... 90 [1 hit](http://blast.ncbi.nlm.nih.gov/Blast.cgi?CMD=Get&RID=5Y3VDS5A013&FORMAT_OBJECT=TaxBlast&NCBI_GI=off&DESCRIPTIONS=500&ALIGNMENTS=250&FORMAT_BLOCK_ON_RESPAGE=Top&MASK_COLOR=1&MASK_CHAR=2#90270) [[g-proteobacteria](http://www.ncbi.nlm.nih.gov/Taxonomy/Browser/wwwtax.cgi?id=1236)] [SET domain-containing protein [Xanthomonas gardneri] >gi|32](http://www.ncbi.nlm.nih.gov/entrez/query.fcgi?cmd=Retrieve&db=Protein&list_uids=493494252&dopt=GenPept)
471. . [Xanthomonas gardneri ATCC 19865](http://www.ncbi.nlm.nih.gov/Taxonomy/Browser/wwwtax.cgi?id=925777) ............................... 90 [1 hit](http://blast.ncbi.nlm.nih.gov/Blast.cgi?CMD=Get&RID=5Y3VDS5A013&FORMAT_OBJECT=TaxBlast&NCBI_GI=off&DESCRIPTIONS=500&ALIGNMENTS=250&FORMAT_BLOCK_ON_RESPAGE=Top&MASK_COLOR=1&MASK_CHAR=2#925777) [[g-proteobacteria](http://www.ncbi.nlm.nih.gov/Taxonomy/Browser/wwwtax.cgi?id=1236)] [SET domain-containing protein [Xanthomonas gardneri] >gi|32](http://www.ncbi.nlm.nih.gov/entrez/query.fcgi?cmd=Retrieve&db=Protein&list_uids=325549943&dopt=GenPept)
472. . [Pedosphaera parvula](http://www.ncbi.nlm.nih.gov/Taxonomy/Browser/wwwtax.cgi?id=1032527) ........................................... 90 [1 hit](http://blast.ncbi.nlm.nih.gov/Blast.cgi?CMD=Get&RID=5Y3VDS5A013&FORMAT_OBJECT=TaxBlast&NCBI_GI=off&DESCRIPTIONS=500&ALIGNMENTS=250&FORMAT_BLOCK_ON_RESPAGE=Top&MASK_COLOR=1&MASK_CHAR=2#1032527) [[verrucomicrobia](http://www.ncbi.nlm.nih.gov/Taxonomy/Browser/wwwtax.cgi?id=74201)] [nuclear protein SET [Pedosphaera parvula] >gi|223896103|gb|](http://www.ncbi.nlm.nih.gov/entrez/query.fcgi?cmd=Retrieve&db=Protein&list_uids=494655482&dopt=GenPept)
473. . [Pedosphaera parvula Ellin514](http://www.ncbi.nlm.nih.gov/Taxonomy/Browser/wwwtax.cgi?id=320771) .................................. 90 [1 hit](http://blast.ncbi.nlm.nih.gov/Blast.cgi?CMD=Get&RID=5Y3VDS5A013&FORMAT_OBJECT=TaxBlast&NCBI_GI=off&DESCRIPTIONS=500&ALIGNMENTS=250&FORMAT_BLOCK_ON_RESPAGE=Top&MASK_COLOR=1&MASK_CHAR=2#320771) [[verrucomicrobia](http://www.ncbi.nlm.nih.gov/Taxonomy/Browser/wwwtax.cgi?id=74201)] [nuclear protein SET [Pedosphaera parvula] >gi|223896103|gb|](http://www.ncbi.nlm.nih.gov/entrez/query.fcgi?cmd=Retrieve&db=Protein&list_uids=223896103&dopt=GenPept)
474. . [Xanthomonas fragariae](http://www.ncbi.nlm.nih.gov/Taxonomy/Browser/wwwtax.cgi?id=48664) ......................................... 90 [1 hit](http://blast.ncbi.nlm.nih.gov/Blast.cgi?CMD=Get&RID=5Y3VDS5A013&FORMAT_OBJECT=TaxBlast&NCBI_GI=off&DESCRIPTIONS=500&ALIGNMENTS=250&FORMAT_BLOCK_ON_RESPAGE=Top&MASK_COLOR=1&MASK_CHAR=2#48664) [[g-proteobacteria](http://www.ncbi.nlm.nih.gov/Taxonomy/Browser/wwwtax.cgi?id=1236)] [nuclear protein SET [Xanthomonas fragariae] >gi|481587897|g](http://www.ncbi.nlm.nih.gov/entrez/query.fcgi?cmd=Retrieve&db=Protein&list_uids=488890151&dopt=GenPept)
475. . [Xanthomonas fragariae LMG 25863](http://www.ncbi.nlm.nih.gov/Taxonomy/Browser/wwwtax.cgi?id=1131451) ............................... 90 [1 hit](http://blast.ncbi.nlm.nih.gov/Blast.cgi?CMD=Get&RID=5Y3VDS5A013&FORMAT_OBJECT=TaxBlast&NCBI_GI=off&DESCRIPTIONS=500&ALIGNMENTS=250&FORMAT_BLOCK_ON_RESPAGE=Top&MASK_COLOR=1&MASK_CHAR=2#1131451) [[g-proteobacteria](http://www.ncbi.nlm.nih.gov/Taxonomy/Browser/wwwtax.cgi?id=1236)] [nuclear protein SET [Xanthomonas fragariae] >gi|481587897|g](http://www.ncbi.nlm.nih.gov/entrez/query.fcgi?cmd=Retrieve&db=Protein&list_uids=481587897&dopt=GenPept)
476. . [Segetibacter koreensis](http://www.ncbi.nlm.nih.gov/Taxonomy/Browser/wwwtax.cgi?id=398037) ........................................ 89 [1 hit](http://blast.ncbi.nlm.nih.gov/Blast.cgi?CMD=Get&RID=5Y3VDS5A013&FORMAT_OBJECT=TaxBlast&NCBI_GI=off&DESCRIPTIONS=500&ALIGNMENTS=250&FORMAT_BLOCK_ON_RESPAGE=Top&MASK_COLOR=1&MASK_CHAR=2#398037) [[CFB group bacteria](http://www.ncbi.nlm.nih.gov/Taxonomy/Browser/wwwtax.cgi?id=976)] [hypothetical protein [Segetibacter koreensis]](http://www.ncbi.nlm.nih.gov/entrez/query.fcgi?cmd=Retrieve&db=Protein&list_uids=517445607&dopt=GenPept)
477. . [Polaromonas sp. CF318](http://www.ncbi.nlm.nih.gov/Taxonomy/Browser/wwwtax.cgi?id=1144318) ......................................... 92 [2 hits](http://blast.ncbi.nlm.nih.gov/Blast.cgi?CMD=Get&RID=5Y3VDS5A013&FORMAT_OBJECT=TaxBlast&NCBI_GI=off&DESCRIPTIONS=500&ALIGNMENTS=250&FORMAT_BLOCK_ON_RESPAGE=Top&MASK_COLOR=1&MASK_CHAR=2#1144318) [[b-proteobacteria](http://www.ncbi.nlm.nih.gov/Taxonomy/Browser/wwwtax.cgi?id=28216)] [lysine methyltransferase [Polaromonas sp. CF318] >gi|398092](http://www.ncbi.nlm.nih.gov/entrez/query.fcgi?cmd=Retrieve&db=Protein&list_uids=495145527&dopt=GenPept)
478. . [Ralstonia pickettii 12J](http://www.ncbi.nlm.nih.gov/Taxonomy/Browser/wwwtax.cgi?id=402626) ....................................... 90 [2 hits](http://blast.ncbi.nlm.nih.gov/Blast.cgi?CMD=Get&RID=5Y3VDS5A013&FORMAT_OBJECT=TaxBlast&NCBI_GI=off&DESCRIPTIONS=500&ALIGNMENTS=250&FORMAT_BLOCK_ON_RESPAGE=Top&MASK_COLOR=1&MASK_CHAR=2#402626) [[b-proteobacteria](http://www.ncbi.nlm.nih.gov/Taxonomy/Browser/wwwtax.cgi?id=28216)] [nuclear protein SET [Ralstonia pickettii 12J] >gi|501405181](http://www.ncbi.nlm.nih.gov/entrez/query.fcgi?cmd=Retrieve&db=Protein&list_uids=187930618&dopt=GenPept)
479. . [Ralstonia pickettii](http://www.ncbi.nlm.nih.gov/Taxonomy/Browser/wwwtax.cgi?id=329) ........................................... 90 [2 hits](http://blast.ncbi.nlm.nih.gov/Blast.cgi?CMD=Get&RID=5Y3VDS5A013&FORMAT_OBJECT=TaxBlast&NCBI_GI=off&DESCRIPTIONS=500&ALIGNMENTS=250&FORMAT_BLOCK_ON_RESPAGE=Top&MASK_COLOR=1&MASK_CHAR=2#329) [[b-proteobacteria](http://www.ncbi.nlm.nih.gov/Taxonomy/Browser/wwwtax.cgi?id=28216)] [nuclear protein SET [Ralstonia pickettii 12J] >gi|501405181](http://www.ncbi.nlm.nih.gov/entrez/query.fcgi?cmd=Retrieve&db=Protein&list_uids=501405181&dopt=GenPept)
480. . [Burkholderia ambifaria AMMD](http://www.ncbi.nlm.nih.gov/Taxonomy/Browser/wwwtax.cgi?id=339670) ................................... 90 [2 hits](http://blast.ncbi.nlm.nih.gov/Blast.cgi?CMD=Get&RID=5Y3VDS5A013&FORMAT_OBJECT=TaxBlast&NCBI_GI=off&DESCRIPTIONS=500&ALIGNMENTS=250&FORMAT_BLOCK_ON_RESPAGE=Top&MASK_COLOR=1&MASK_CHAR=2#339670) [[b-proteobacteria](http://www.ncbi.nlm.nih.gov/Taxonomy/Browser/wwwtax.cgi?id=28216)] [nuclear protein SET [Burkholderia ambifaria AMMD] >gi|49997](http://www.ncbi.nlm.nih.gov/entrez/query.fcgi?cmd=Retrieve&db=Protein&list_uids=115350120&dopt=GenPept)
481. . [Burkholderia ambifaria](http://www.ncbi.nlm.nih.gov/Taxonomy/Browser/wwwtax.cgi?id=152480) ........................................ 90 [2 hits](http://blast.ncbi.nlm.nih.gov/Blast.cgi?CMD=Get&RID=5Y3VDS5A013&FORMAT_OBJECT=TaxBlast&NCBI_GI=off&DESCRIPTIONS=500&ALIGNMENTS=250&FORMAT_BLOCK_ON_RESPAGE=Top&MASK_COLOR=1&MASK_CHAR=2#152480) [[b-proteobacteria](http://www.ncbi.nlm.nih.gov/Taxonomy/Browser/wwwtax.cgi?id=28216)] [nuclear protein SET [Burkholderia ambifaria AMMD] >gi|49997](http://www.ncbi.nlm.nih.gov/entrez/query.fcgi?cmd=Retrieve&db=Protein&list_uids=499974876&dopt=GenPept)
482. . [Xanthomonas translucens](http://www.ncbi.nlm.nih.gov/Taxonomy/Browser/wwwtax.cgi?id=343) ....................................... 90 [2 hits](http://blast.ncbi.nlm.nih.gov/Blast.cgi?CMD=Get&RID=5Y3VDS5A013&FORMAT_OBJECT=TaxBlast&NCBI_GI=off&DESCRIPTIONS=500&ALIGNMENTS=250&FORMAT_BLOCK_ON_RESPAGE=Top&MASK_COLOR=1&MASK_CHAR=2#343) [[g-proteobacteria](http://www.ncbi.nlm.nih.gov/Taxonomy/Browser/wwwtax.cgi?id=1236)] [hypothetical protein [Xanthomonas translucens] >gi|42279711](http://www.ncbi.nlm.nih.gov/entrez/query.fcgi?cmd=Retrieve&db=Protein&list_uids=489573755&dopt=GenPept)
483. . [Xanthomonas translucens pv. graminis ART-Xtg29](http://www.ncbi.nlm.nih.gov/Taxonomy/Browser/wwwtax.cgi?id=1195885) ................ 90 [1 hit](http://blast.ncbi.nlm.nih.gov/Blast.cgi?CMD=Get&RID=5Y3VDS5A013&FORMAT_OBJECT=TaxBlast&NCBI_GI=off&DESCRIPTIONS=500&ALIGNMENTS=250&FORMAT_BLOCK_ON_RESPAGE=Top&MASK_COLOR=1&MASK_CHAR=2#1195885) [[g-proteobacteria](http://www.ncbi.nlm.nih.gov/Taxonomy/Browser/wwwtax.cgi?id=1236)] [hypothetical protein [Xanthomonas translucens] >gi|42279711](http://www.ncbi.nlm.nih.gov/entrez/query.fcgi?cmd=Retrieve&db=Protein&list_uids=422797114&dopt=GenPept)
484. . [Xanthomonas translucens pv. translucens DSM 18974](http://www.ncbi.nlm.nih.gov/Taxonomy/Browser/wwwtax.cgi?id=1261556) ............. 90 [1 hit](http://blast.ncbi.nlm.nih.gov/Blast.cgi?CMD=Get&RID=5Y3VDS5A013&FORMAT_OBJECT=TaxBlast&NCBI_GI=off&DESCRIPTIONS=500&ALIGNMENTS=250&FORMAT_BLOCK_ON_RESPAGE=Top&MASK_COLOR=1&MASK_CHAR=2#1261556) [[g-proteobacteria](http://www.ncbi.nlm.nih.gov/Taxonomy/Browser/wwwtax.cgi?id=1236)] [hypothetical protein [Xanthomonas translucens] >gi|42279711](http://www.ncbi.nlm.nih.gov/entrez/query.fcgi?cmd=Retrieve&db=Protein&list_uids=430816544&dopt=GenPept)
485. . [Xanthomonas translucens DAR61454](http://www.ncbi.nlm.nih.gov/Taxonomy/Browser/wwwtax.cgi?id=1205753) .............................. 90 [1 hit](http://blast.ncbi.nlm.nih.gov/Blast.cgi?CMD=Get&RID=5Y3VDS5A013&FORMAT_OBJECT=TaxBlast&NCBI_GI=off&DESCRIPTIONS=500&ALIGNMENTS=250&FORMAT_BLOCK_ON_RESPAGE=Top&MASK_COLOR=1&MASK_CHAR=2#1205753) [[g-proteobacteria](http://www.ncbi.nlm.nih.gov/Taxonomy/Browser/wwwtax.cgi?id=1236)] [hypothetical protein [Xanthomonas translucens] >gi|44036357](http://www.ncbi.nlm.nih.gov/entrez/query.fcgi?cmd=Retrieve&db=Protein&list_uids=440363577&dopt=GenPept)
486. . [Variovorax sp. CF313](http://www.ncbi.nlm.nih.gov/Taxonomy/Browser/wwwtax.cgi?id=1144315) .......................................... 90 [2 hits](http://blast.ncbi.nlm.nih.gov/Blast.cgi?CMD=Get&RID=5Y3VDS5A013&FORMAT_OBJECT=TaxBlast&NCBI_GI=off&DESCRIPTIONS=500&ALIGNMENTS=250&FORMAT_BLOCK_ON_RESPAGE=Top&MASK_COLOR=1&MASK_CHAR=2#1144315) [[b-proteobacteria](http://www.ncbi.nlm.nih.gov/Taxonomy/Browser/wwwtax.cgi?id=28216)] [lysine methyltransferase [Variovorax sp. CF313] >gi|3980891](http://www.ncbi.nlm.nih.gov/entrez/query.fcgi?cmd=Retrieve&db=Protein&list_uids=495103863&dopt=GenPept)
487. . [beta proteobacterium CB](http://www.ncbi.nlm.nih.gov/Taxonomy/Browser/wwwtax.cgi?id=543913) ....................................... 90 [3 hits](http://blast.ncbi.nlm.nih.gov/Blast.cgi?CMD=Get&RID=5Y3VDS5A013&FORMAT_OBJECT=TaxBlast&NCBI_GI=off&DESCRIPTIONS=500&ALIGNMENTS=250&FORMAT_BLOCK_ON_RESPAGE=Top&MASK_COLOR=1&MASK_CHAR=2#543913) [[b-proteobacteria](http://www.ncbi.nlm.nih.gov/Taxonomy/Browser/wwwtax.cgi?id=28216)] [Nuclear protein SET [beta proteobacterium CB] >gi|505233088](http://www.ncbi.nlm.nih.gov/entrez/query.fcgi?cmd=Retrieve&db=Protein&list_uids=456062341&dopt=GenPept)
488. . [Chlamydia psittaci Mat116](http://www.ncbi.nlm.nih.gov/Taxonomy/Browser/wwwtax.cgi?id=500464) ..................................... 91 [2 hits](http://blast.ncbi.nlm.nih.gov/Blast.cgi?CMD=Get&RID=5Y3VDS5A013&FORMAT_OBJECT=TaxBlast&NCBI_GI=off&DESCRIPTIONS=500&ALIGNMENTS=250&FORMAT_BLOCK_ON_RESPAGE=Top&MASK_COLOR=1&MASK_CHAR=2#500464) [[chlamydias](http://www.ncbi.nlm.nih.gov/Taxonomy/Browser/wwwtax.cgi?id=51291)] [hypothetical protein AO9_04565 [Chlamydia psittaci Mat116]](http://www.ncbi.nlm.nih.gov/entrez/query.fcgi?cmd=Retrieve&db=Protein&list_uids=449071532&dopt=GenPept)
489. . [Candidatus Chloracidobacterium thermophilum B](http://www.ncbi.nlm.nih.gov/Taxonomy/Browser/wwwtax.cgi?id=981222) ................. 90 [2 hits](http://blast.ncbi.nlm.nih.gov/Blast.cgi?CMD=Get&RID=5Y3VDS5A013&FORMAT_OBJECT=TaxBlast&NCBI_GI=off&DESCRIPTIONS=500&ALIGNMENTS=250&FORMAT_BLOCK_ON_RESPAGE=Top&MASK_COLOR=1&MASK_CHAR=2#981222) [[bacteria](http://www.ncbi.nlm.nih.gov/Taxonomy/Browser/wwwtax.cgi?id=2)] [hypothetical protein [Candidatus Chloracidobacterium thermo](http://www.ncbi.nlm.nih.gov/entrez/query.fcgi?cmd=Retrieve&db=Protein&list_uids=347756045&dopt=GenPept)
490. . [Candidatus Chloracidobacterium thermophilum](http://www.ncbi.nlm.nih.gov/Taxonomy/Browser/wwwtax.cgi?id=458033) ................... 90 [1 hit](http://blast.ncbi.nlm.nih.gov/Blast.cgi?CMD=Get&RID=5Y3VDS5A013&FORMAT_OBJECT=TaxBlast&NCBI_GI=off&DESCRIPTIONS=500&ALIGNMENTS=250&FORMAT_BLOCK_ON_RESPAGE=Top&MASK_COLOR=1&MASK_CHAR=2#458033) [[bacteria](http://www.ncbi.nlm.nih.gov/Taxonomy/Browser/wwwtax.cgi?id=2)] [hypothetical protein [Candidatus Chloracidobacterium thermo](http://www.ncbi.nlm.nih.gov/entrez/query.fcgi?cmd=Retrieve&db=Protein&list_uids=503866835&dopt=GenPept)
491. . [Burkholderia ambifaria MC40-6](http://www.ncbi.nlm.nih.gov/Taxonomy/Browser/wwwtax.cgi?id=398577) ................................. 90 [2 hits](http://blast.ncbi.nlm.nih.gov/Blast.cgi?CMD=Get&RID=5Y3VDS5A013&FORMAT_OBJECT=TaxBlast&NCBI_GI=off&DESCRIPTIONS=500&ALIGNMENTS=250&FORMAT_BLOCK_ON_RESPAGE=Top&MASK_COLOR=1&MASK_CHAR=2#398577) [[b-proteobacteria](http://www.ncbi.nlm.nih.gov/Taxonomy/Browser/wwwtax.cgi?id=28216)] [nuclear protein SET [Burkholderia ambifaria MC40-6] >gi|501](http://www.ncbi.nlm.nih.gov/entrez/query.fcgi?cmd=Retrieve&db=Protein&list_uids=172059138&dopt=GenPept)
492. . [Curvibacter putative symbiont of Hydra magnipapillata](http://www.ncbi.nlm.nih.gov/Taxonomy/Browser/wwwtax.cgi?id=667019) ......... 90 [1 hit](http://blast.ncbi.nlm.nih.gov/Blast.cgi?CMD=Get&RID=5Y3VDS5A013&FORMAT_OBJECT=TaxBlast&NCBI_GI=off&DESCRIPTIONS=500&ALIGNMENTS=250&FORMAT_BLOCK_ON_RESPAGE=Top&MASK_COLOR=1&MASK_CHAR=2#667019) [[b-proteobacteria](http://www.ncbi.nlm.nih.gov/Taxonomy/Browser/wwwtax.cgi?id=28216)] [hypothetical protein Csp_C25820 [Curvibacter putative symbi](http://www.ncbi.nlm.nih.gov/entrez/query.fcgi?cmd=Retrieve&db=Protein&list_uids=260221883&dopt=GenPept)
493. . [Ralstonia pickettii 12D](http://www.ncbi.nlm.nih.gov/Taxonomy/Browser/wwwtax.cgi?id=428406) ....................................... 90 [2 hits](http://blast.ncbi.nlm.nih.gov/Blast.cgi?CMD=Get&RID=5Y3VDS5A013&FORMAT_OBJECT=TaxBlast&NCBI_GI=off&DESCRIPTIONS=500&ALIGNMENTS=250&FORMAT_BLOCK_ON_RESPAGE=Top&MASK_COLOR=1&MASK_CHAR=2#428406) [[b-proteobacteria](http://www.ncbi.nlm.nih.gov/Taxonomy/Browser/wwwtax.cgi?id=28216)] [nuclear protein SET [Ralstonia pickettii 12D] >gi|506336250](http://www.ncbi.nlm.nih.gov/entrez/query.fcgi?cmd=Retrieve&db=Protein&list_uids=241664808&dopt=GenPept)
494. . [Methanosarcina acetivorans C2A](http://www.ncbi.nlm.nih.gov/Taxonomy/Browser/wwwtax.cgi?id=188937) ................................ 88 [2 hits](http://blast.ncbi.nlm.nih.gov/Blast.cgi?CMD=Get&RID=5Y3VDS5A013&FORMAT_OBJECT=TaxBlast&NCBI_GI=off&DESCRIPTIONS=500&ALIGNMENTS=250&FORMAT_BLOCK_ON_RESPAGE=Top&MASK_COLOR=1&MASK_CHAR=2#188937) [[euryarchaeotes](http://www.ncbi.nlm.nih.gov/Taxonomy/Browser/wwwtax.cgi?id=28890)] [hypothetical protein MA1679 [Methanosarcina acetivorans C2A](http://www.ncbi.nlm.nih.gov/entrez/query.fcgi?cmd=Retrieve&db=Protein&list_uids=20090531&dopt=GenPept)
495. . [Methanosarcina acetivorans](http://www.ncbi.nlm.nih.gov/Taxonomy/Browser/wwwtax.cgi?id=2214) .................................... 88 [1 hit](http://blast.ncbi.nlm.nih.gov/Blast.cgi?CMD=Get&RID=5Y3VDS5A013&FORMAT_OBJECT=TaxBlast&NCBI_GI=off&DESCRIPTIONS=500&ALIGNMENTS=250&FORMAT_BLOCK_ON_RESPAGE=Top&MASK_COLOR=1&MASK_CHAR=2#2214) [[euryarchaeotes](http://www.ncbi.nlm.nih.gov/Taxonomy/Browser/wwwtax.cgi?id=28890)] [hypothetical protein MA1679 [Methanosarcina acetivorans C2A](http://www.ncbi.nlm.nih.gov/entrez/query.fcgi?cmd=Retrieve&db=Protein&list_uids=499331191&dopt=GenPept)
496. . [Polynucleobacter necessarius subsp. asymbioticus QLW-P1DMWA-1](http://www.ncbi.nlm.nih.gov/Taxonomy/Browser/wwwtax.cgi?id=312153) . 90 [2 hits](http://blast.ncbi.nlm.nih.gov/Blast.cgi?CMD=Get&RID=5Y3VDS5A013&FORMAT_OBJECT=TaxBlast&NCBI_GI=off&DESCRIPTIONS=500&ALIGNMENTS=250&FORMAT_BLOCK_ON_RESPAGE=Top&MASK_COLOR=1&MASK_CHAR=2#312153) [[b-proteobacteria](http://www.ncbi.nlm.nih.gov/Taxonomy/Browser/wwwtax.cgi?id=28216)] [nuclear protein SET [Polynucleobacter necessarius subsp. as](http://www.ncbi.nlm.nih.gov/entrez/query.fcgi?cmd=Retrieve&db=Protein&list_uids=145588193&dopt=GenPept)
497. . [Variovorax paradoxus EPS](http://www.ncbi.nlm.nih.gov/Taxonomy/Browser/wwwtax.cgi?id=595537) ...................................... 90 [2 hits](http://blast.ncbi.nlm.nih.gov/Blast.cgi?CMD=Get&RID=5Y3VDS5A013&FORMAT_OBJECT=TaxBlast&NCBI_GI=off&DESCRIPTIONS=500&ALIGNMENTS=250&FORMAT_BLOCK_ON_RESPAGE=Top&MASK_COLOR=1&MASK_CHAR=2#595537) [[b-proteobacteria](http://www.ncbi.nlm.nih.gov/Taxonomy/Browser/wwwtax.cgi?id=28216)] [nuclear protein set [Variovorax paradoxus EPS] >gi|50330961](http://www.ncbi.nlm.nih.gov/entrez/query.fcgi?cmd=Retrieve&db=Protein&list_uids=319796552&dopt=GenPept)
498. . [Burkholderia sp. BT03](http://www.ncbi.nlm.nih.gov/Taxonomy/Browser/wwwtax.cgi?id=1144309) ......................................... 89 [2 hits](http://blast.ncbi.nlm.nih.gov/Blast.cgi?CMD=Get&RID=5Y3VDS5A013&FORMAT_OBJECT=TaxBlast&NCBI_GI=off&DESCRIPTIONS=500&ALIGNMENTS=250&FORMAT_BLOCK_ON_RESPAGE=Top&MASK_COLOR=1&MASK_CHAR=2#1144309) [[b-proteobacteria](http://www.ncbi.nlm.nih.gov/Taxonomy/Browser/wwwtax.cgi?id=28216)] [SET domain-containing protein [Burkholderia sp. BT03] >gi|3](http://www.ncbi.nlm.nih.gov/entrez/query.fcgi?cmd=Retrieve&db=Protein&list_uids=495011797&dopt=GenPept)
499. . [Ralstonia solanacearum](http://www.ncbi.nlm.nih.gov/Taxonomy/Browser/wwwtax.cgi?id=305) ........................................ 90 [2 hits](http://blast.ncbi.nlm.nih.gov/Blast.cgi?CMD=Get&RID=5Y3VDS5A013&FORMAT_OBJECT=TaxBlast&NCBI_GI=off&DESCRIPTIONS=500&ALIGNMENTS=250&FORMAT_BLOCK_ON_RESPAGE=Top&MASK_COLOR=1&MASK_CHAR=2#305) [[b-proteobacteria](http://www.ncbi.nlm.nih.gov/Taxonomy/Browser/wwwtax.cgi?id=28216)] [nuclear protein SET [Ralstonia solanacearum]](http://www.ncbi.nlm.nih.gov/entrez/query.fcgi?cmd=Retrieve&db=Protein&list_uids=521094223&dopt=GenPept)
500. . [Ramlibacter tataouinensis TTB310](http://www.ncbi.nlm.nih.gov/Taxonomy/Browser/wwwtax.cgi?id=365046) .............................. 89 [2 hits](http://blast.ncbi.nlm.nih.gov/Blast.cgi?CMD=Get&RID=5Y3VDS5A013&FORMAT_OBJECT=TaxBlast&NCBI_GI=off&DESCRIPTIONS=500&ALIGNMENTS=250&FORMAT_BLOCK_ON_RESPAGE=Top&MASK_COLOR=1&MASK_CHAR=2#365046) [[b-proteobacteria](http://www.ncbi.nlm.nih.gov/Taxonomy/Browser/wwwtax.cgi?id=28216)] [proteins containing SET domain [Ramlibacter tataouinensis T](http://www.ncbi.nlm.nih.gov/entrez/query.fcgi?cmd=Retrieve&db=Protein&list_uids=337281414&dopt=GenPept)
501. . [Ramlibacter tataouinensis](http://www.ncbi.nlm.nih.gov/Taxonomy/Browser/wwwtax.cgi?id=94132) ..................................... 89 [1 hit](http://blast.ncbi.nlm.nih.gov/Blast.cgi?CMD=Get&RID=5Y3VDS5A013&FORMAT_OBJECT=TaxBlast&NCBI_GI=off&DESCRIPTIONS=500&ALIGNMENTS=250&FORMAT_BLOCK_ON_RESPAGE=Top&MASK_COLOR=1&MASK_CHAR=2#94132) [[b-proteobacteria](http://www.ncbi.nlm.nih.gov/Taxonomy/Browser/wwwtax.cgi?id=28216)] [proteins containing SET domain [Ramlibacter tataouinensis T](http://www.ncbi.nlm.nih.gov/entrez/query.fcgi?cmd=Retrieve&db=Protein&list_uids=503669019&dopt=GenPept)
502. . [Cupriavidus sp. HPC(L)](http://www.ncbi.nlm.nih.gov/Taxonomy/Browser/wwwtax.cgi?id=1217418) ........................................ 91 [2 hits](http://blast.ncbi.nlm.nih.gov/Blast.cgi?CMD=Get&RID=5Y3VDS5A013&FORMAT_OBJECT=TaxBlast&NCBI_GI=off&DESCRIPTIONS=500&ALIGNMENTS=250&FORMAT_BLOCK_ON_RESPAGE=Top&MASK_COLOR=1&MASK_CHAR=2#1217418) [[b-proteobacteria](http://www.ncbi.nlm.nih.gov/Taxonomy/Browser/wwwtax.cgi?id=28216)] [nuclear protein SET [Cupriavidus sp. HPC(L)] >gi|409770920|](http://www.ncbi.nlm.nih.gov/entrez/query.fcgi?cmd=Retrieve&db=Protein&list_uids=493627669&dopt=GenPept)
503. . [Cupriavidus sp. UYPR2.512](http://www.ncbi.nlm.nih.gov/Taxonomy/Browser/wwwtax.cgi?id=1080187) ..................................... 52 [1 hit](http://blast.ncbi.nlm.nih.gov/Blast.cgi?CMD=Get&RID=5Y2VSSZC016&FORMAT_OBJECT=TaxBlast&NCBI_GI=off&DESCRIPTIONS=100&ALIGNMENTS=100&FORMAT_BLOCK_ON_RESPAGE=Top&MASK_COLOR=1&MASK_CHAR=2#1080187) [[b-proteobacteria](http://www.ncbi.nlm.nih.gov/Taxonomy/Browser/wwwtax.cgi?id=28216)] [nuclear protein SET [Cupriavidus sp. UYPR2.512]](http://www.ncbi.nlm.nih.gov/entrez/query.fcgi?cmd=Retrieve&db=Protein&list_uids=517123204&dopt=GenPept)
504. . [Myxococcus stipitatus DSM 14675](http://www.ncbi.nlm.nih.gov/Taxonomy/Browser/wwwtax.cgi?id=1278073) ............................... 90 [2 hits](http://blast.ncbi.nlm.nih.gov/Blast.cgi?CMD=Get&RID=5Y3VDS5A013&FORMAT_OBJECT=TaxBlast&NCBI_GI=off&DESCRIPTIONS=500&ALIGNMENTS=250&FORMAT_BLOCK_ON_RESPAGE=Top&MASK_COLOR=1&MASK_CHAR=2#1278073) [[d-proteobacteria](http://www.ncbi.nlm.nih.gov/Taxonomy/Browser/wwwtax.cgi?id=28221)] [SET domain-containing protein [Myxococcus stipitatus DSM 14](http://www.ncbi.nlm.nih.gov/entrez/query.fcgi?cmd=Retrieve&db=Protein&list_uids=442319745&dopt=GenPept)
505. . [Myxococcus stipitatus](http://www.ncbi.nlm.nih.gov/Taxonomy/Browser/wwwtax.cgi?id=83455) ......................................... 90 [1 hit](http://blast.ncbi.nlm.nih.gov/Blast.cgi?CMD=Get&RID=5Y3VDS5A013&FORMAT_OBJECT=TaxBlast&NCBI_GI=off&DESCRIPTIONS=500&ALIGNMENTS=250&FORMAT_BLOCK_ON_RESPAGE=Top&MASK_COLOR=1&MASK_CHAR=2#83455) [[d-proteobacteria](http://www.ncbi.nlm.nih.gov/Taxonomy/Browser/wwwtax.cgi?id=28221)] [SET domain-containing protein [Myxococcus stipitatus DSM 14](http://www.ncbi.nlm.nih.gov/entrez/query.fcgi?cmd=Retrieve&db=Protein&list_uids=505161241&dopt=GenPept)
506. . [Burkholderia gladioli BSR3](http://www.ncbi.nlm.nih.gov/Taxonomy/Browser/wwwtax.cgi?id=999541) .................................... 90 [2 hits](http://blast.ncbi.nlm.nih.gov/Blast.cgi?CMD=Get&RID=5Y3VDS5A013&FORMAT_OBJECT=TaxBlast&NCBI_GI=off&DESCRIPTIONS=500&ALIGNMENTS=250&FORMAT_BLOCK_ON_RESPAGE=Top&MASK_COLOR=1&MASK_CHAR=2#999541) [[b-proteobacteria](http://www.ncbi.nlm.nih.gov/Taxonomy/Browser/wwwtax.cgi?id=28216)] [SET domain-containing protein [Burkholderia gladioli BSR3]](http://www.ncbi.nlm.nih.gov/entrez/query.fcgi?cmd=Retrieve&db=Protein&list_uids=330815014&dopt=GenPept)
507. . [Burkholderia gladioli](http://www.ncbi.nlm.nih.gov/Taxonomy/Browser/wwwtax.cgi?id=28095) ......................................... 90 [1 hit](http://blast.ncbi.nlm.nih.gov/Blast.cgi?CMD=Get&RID=5Y3VDS5A013&FORMAT_OBJECT=TaxBlast&NCBI_GI=off&DESCRIPTIONS=500&ALIGNMENTS=250&FORMAT_BLOCK_ON_RESPAGE=Top&MASK_COLOR=1&MASK_CHAR=2#28095) [[b-proteobacteria](http://www.ncbi.nlm.nih.gov/Taxonomy/Browser/wwwtax.cgi?id=28216)] [SET domain-containing protein [Burkholderia gladioli BSR3]](http://www.ncbi.nlm.nih.gov/entrez/query.fcgi?cmd=Retrieve&db=Protein&list_uids=503461483&dopt=GenPept)
508. . [Ralstonia solanacearum Po82](http://www.ncbi.nlm.nih.gov/Taxonomy/Browser/wwwtax.cgi?id=1031711) ................................... 90 [2 hits](http://blast.ncbi.nlm.nih.gov/Blast.cgi?CMD=Get&RID=5Y3VDS5A013&FORMAT_OBJECT=TaxBlast&NCBI_GI=off&DESCRIPTIONS=500&ALIGNMENTS=250&FORMAT_BLOCK_ON_RESPAGE=Top&MASK_COLOR=1&MASK_CHAR=2#1031711) [[b-proteobacteria](http://www.ncbi.nlm.nih.gov/Taxonomy/Browser/wwwtax.cgi?id=28216)] [set domain protein [Ralstonia solanacearum Po82] >gi|504428](http://www.ncbi.nlm.nih.gov/entrez/query.fcgi?cmd=Retrieve&db=Protein&list_uids=386331753&dopt=GenPept)
509. . [Niastella koreensis GR20-10](http://www.ncbi.nlm.nih.gov/Taxonomy/Browser/wwwtax.cgi?id=700598) ................................. 60 [2 hits](http://blast.ncbi.nlm.nih.gov/Blast.cgi?CMD=Get&RID=5Y2VSSZC016&FORMAT_OBJECT=TaxBlast&NCBI_GI=off&DESCRIPTIONS=100&ALIGNMENTS=100&FORMAT_BLOCK_ON_RESPAGE=Top&MASK_COLOR=1&MASK_CHAR=2#700598) [[CFB group bacteria](http://www.ncbi.nlm.nih.gov/Taxonomy/Browser/wwwtax.cgi?id=976)] [nuclear protein SET [Niastella koreensis GR20-10] >gi|50398](http://www.ncbi.nlm.nih.gov/entrez/query.fcgi?cmd=Retrieve&db=Protein&list_uids=375143696&dopt=GenPept)
510. . [Niastella koreensis](http://www.ncbi.nlm.nih.gov/Taxonomy/Browser/wwwtax.cgi?id=354356) ......................................... 60 [1 hit](http://blast.ncbi.nlm.nih.gov/Blast.cgi?CMD=Get&RID=5Y2VSSZC016&FORMAT_OBJECT=TaxBlast&NCBI_GI=off&DESCRIPTIONS=100&ALIGNMENTS=100&FORMAT_BLOCK_ON_RESPAGE=Top&MASK_COLOR=1&MASK_CHAR=2#354356) [[CFB group bacteria](http://www.ncbi.nlm.nih.gov/Taxonomy/Browser/wwwtax.cgi?id=976)] [nuclear protein SET [Niastella koreensis GR20-10] >gi|50398](http://www.ncbi.nlm.nih.gov/entrez/query.fcgi?cmd=Retrieve&db=Protein&list_uids=503982653&dopt=GenPept)
511. . [Haliscomenobacter hydrossis DSM 1100](http://www.ncbi.nlm.nih.gov/Taxonomy/Browser/wwwtax.cgi?id=760192) ------------------------ 69 [2 hits](http://blast.ncbi.nlm.nih.gov/Blast.cgi?CMD=Get&RID=5Y2VSSZC016&FORMAT_OBJECT=TaxBlast&NCBI_GI=off&DESCRIPTIONS=100&ALIGNMENTS=100&FORMAT_BLOCK_ON_RESPAGE=Top&MASK_COLOR=1&MASK_CHAR=2#760192) [[CFB group bacteria](http://www.ncbi.nlm.nih.gov/Taxonomy/Browser/wwwtax.cgi?id=976)] [nuclear protein SET [Haliscomenobacter hydrossis DSM 1100]](http://www.ncbi.nlm.nih.gov/entrez/query.fcgi?cmd=Retrieve&db=Protein&list_uids=332662681&dopt=GenPept)
512. . [Haliscomenobacter hydrossis](http://www.ncbi.nlm.nih.gov/Taxonomy/Browser/wwwtax.cgi?id=2350) ................................. 69 [1 hit](http://blast.ncbi.nlm.nih.gov/Blast.cgi?CMD=Get&RID=5Y2VSSZC016&FORMAT_OBJECT=TaxBlast&NCBI_GI=off&DESCRIPTIONS=100&ALIGNMENTS=100&FORMAT_BLOCK_ON_RESPAGE=Top&MASK_COLOR=1&MASK_CHAR=2#2350) [[CFB group bacteria](http://www.ncbi.nlm.nih.gov/Taxonomy/Browser/wwwtax.cgi?id=976)] [nuclear protein SET [Haliscomenobacter hydrossis DSM 1100]](http://www.ncbi.nlm.nih.gov/entrez/query.fcgi?cmd=Retrieve&db=Protein&list_uids=503529030&dopt=GenPept)
513. . [Saprospira grandis str. Lewin](http://www.ncbi.nlm.nih.gov/Taxonomy/Browser/wwwtax.cgi?id=984262) ............................... 55 [2 hits](http://blast.ncbi.nlm.nih.gov/Blast.cgi?CMD=Get&RID=5Y2VSSZC016&FORMAT_OBJECT=TaxBlast&NCBI_GI=off&DESCRIPTIONS=100&ALIGNMENTS=100&FORMAT_BLOCK_ON_RESPAGE=Top&MASK_COLOR=1&MASK_CHAR=2#984262) [[CFB group bacteria](http://www.ncbi.nlm.nih.gov/Taxonomy/Browser/wwwtax.cgi?id=976)] [nuclear protein SET [Saprospira grandis str. Lewin] >gi|505](http://www.ncbi.nlm.nih.gov/entrez/query.fcgi?cmd=Retrieve&db=Protein&list_uids=379730569&dopt=GenPept)
514. . [Saprospira grandis](http://www.ncbi.nlm.nih.gov/Taxonomy/Browser/wwwtax.cgi?id=1008) .......................................... 55 [2 hits](http://blast.ncbi.nlm.nih.gov/Blast.cgi?CMD=Get&RID=5Y2VSSZC016&FORMAT_OBJECT=TaxBlast&NCBI_GI=off&DESCRIPTIONS=100&ALIGNMENTS=100&FORMAT_BLOCK_ON_RESPAGE=Top&MASK_COLOR=1&MASK_CHAR=2#1008) [[CFB group bacteria](http://www.ncbi.nlm.nih.gov/Taxonomy/Browser/wwwtax.cgi?id=976)] [nuclear protein SET [Saprospira grandis str. Lewin] >gi|505](http://www.ncbi.nlm.nih.gov/entrez/query.fcgi?cmd=Retrieve&db=Protein&list_uids=505730822&dopt=GenPept)
515. . [Saprospira grandis DSM 2844](http://www.ncbi.nlm.nih.gov/Taxonomy/Browser/wwwtax.cgi?id=694433) ................................. 54 [1 hit](http://blast.ncbi.nlm.nih.gov/Blast.cgi?CMD=Get&RID=5Y2VSSZC016&FORMAT_OBJECT=TaxBlast&NCBI_GI=off&DESCRIPTIONS=100&ALIGNMENTS=100&FORMAT_BLOCK_ON_RESPAGE=Top&MASK_COLOR=1&MASK_CHAR=2#694433) [[CFB group bacteria](http://www.ncbi.nlm.nih.gov/Taxonomy/Browser/wwwtax.cgi?id=976)] [lysine methyltransferase [Saprospira grandis] >gi|395319968](http://www.ncbi.nlm.nih.gov/entrez/query.fcgi?cmd=Retrieve&db=Protein&list_uids=395319968&dopt=GenPept)
516. . [Chitinophaga pinensis DSM 2588](http://www.ncbi.nlm.nih.gov/Taxonomy/Browser/wwwtax.cgi?id=485918) .............................. 55 [2 hits](http://blast.ncbi.nlm.nih.gov/Blast.cgi?CMD=Get&RID=5Y2VSSZC016&FORMAT_OBJECT=TaxBlast&NCBI_GI=off&DESCRIPTIONS=100&ALIGNMENTS=100&FORMAT_BLOCK_ON_RESPAGE=Top&MASK_COLOR=1&MASK_CHAR=2#485918) [[CFB group bacteria](http://www.ncbi.nlm.nih.gov/Taxonomy/Browser/wwwtax.cgi?id=976)] [nuclear protein SET [Chitinophaga pinensis DSM 2588] >gi|50](http://www.ncbi.nlm.nih.gov/entrez/query.fcgi?cmd=Retrieve&db=Protein&list_uids=256421047&dopt=GenPept)
517. . [Chitinophaga pinensis](http://www.ncbi.nlm.nih.gov/Taxonomy/Browser/wwwtax.cgi?id=79329) ....................................... 55 [1 hit](http://blast.ncbi.nlm.nih.gov/Blast.cgi?CMD=Get&RID=5Y2VSSZC016&FORMAT_OBJECT=TaxBlast&NCBI_GI=off&DESCRIPTIONS=100&ALIGNMENTS=100&FORMAT_BLOCK_ON_RESPAGE=Top&MASK_COLOR=1&MASK_CHAR=2#79329) [[CFB group bacteria](http://www.ncbi.nlm.nih.gov/Taxonomy/Browser/wwwtax.cgi?id=976)] [nuclear protein SET [Chitinophaga pinensis DSM 2588] >gi|50](http://www.ncbi.nlm.nih.gov/entrez/query.fcgi?cmd=Retrieve&db=Protein&list_uids=502447036&dopt=GenPept)
518. . [Anabaena cylindrica PCC 7122](http://www.ncbi.nlm.nih.gov/Taxonomy/Browser/wwwtax.cgi?id=272123) .................................. 55 [2 hits](http://blast.ncbi.nlm.nih.gov/Blast.cgi?CMD=Get&RID=5Y2VSSZC016&FORMAT_OBJECT=TaxBlast&NCBI_GI=off&DESCRIPTIONS=100&ALIGNMENTS=100&FORMAT_BLOCK_ON_RESPAGE=Top&MASK_COLOR=1&MASK_CHAR=2#272123) [[cyanobacteria](http://www.ncbi.nlm.nih.gov/Taxonomy/Browser/wwwtax.cgi?id=1117)] [nuclear protein SET [Anabaena cylindrica PCC 7122] >gi|5050](http://www.ncbi.nlm.nih.gov/entrez/query.fcgi?cmd=Retrieve&db=Protein&list_uids=440681812&dopt=GenPept)
519. . [Anabaena cylindrica](http://www.ncbi.nlm.nih.gov/Taxonomy/Browser/wwwtax.cgi?id=1165) ........................................... 55 [1 hit](http://blast.ncbi.nlm.nih.gov/Blast.cgi?CMD=Get&RID=5Y2VSSZC016&FORMAT_OBJECT=TaxBlast&NCBI_GI=off&DESCRIPTIONS=100&ALIGNMENTS=100&FORMAT_BLOCK_ON_RESPAGE=Top&MASK_COLOR=1&MASK_CHAR=2#1165) [[cyanobacteria](http://www.ncbi.nlm.nih.gov/Taxonomy/Browser/wwwtax.cgi?id=1117)] [nuclear protein SET [Anabaena cylindrica PCC 7122] >gi|5050](http://www.ncbi.nlm.nih.gov/entrez/query.fcgi?cmd=Retrieve&db=Protein&list_uids=505027231&dopt=GenPept)
520. . [Cylindrospermum stagnale PCC 7417](http://www.ncbi.nlm.nih.gov/Taxonomy/Browser/wwwtax.cgi?id=56107) ............................. 52 [2 hits](http://blast.ncbi.nlm.nih.gov/Blast.cgi?CMD=Get&RID=5Y2VSSZC016&FORMAT_OBJECT=TaxBlast&NCBI_GI=off&DESCRIPTIONS=100&ALIGNMENTS=100&FORMAT_BLOCK_ON_RESPAGE=Top&MASK_COLOR=1&MASK_CHAR=2#56107) [[cyanobacteria](http://www.ncbi.nlm.nih.gov/Taxonomy/Browser/wwwtax.cgi?id=1117)] [SET domain-containing protein [Cylindrospermum stagnale PCC](http://www.ncbi.nlm.nih.gov/entrez/query.fcgi?cmd=Retrieve&db=Protein&list_uids=434407260&dopt=GenPept)
521. . [Cylindrospermum stagnale](http://www.ncbi.nlm.nih.gov/Taxonomy/Browser/wwwtax.cgi?id=142864) ...................................... 52 [1 hit](http://blast.ncbi.nlm.nih.gov/Blast.cgi?CMD=Get&RID=5Y2VSSZC016&FORMAT_OBJECT=TaxBlast&NCBI_GI=off&DESCRIPTIONS=100&ALIGNMENTS=100&FORMAT_BLOCK_ON_RESPAGE=Top&MASK_COLOR=1&MASK_CHAR=2#142864) [[cyanobacteria](http://www.ncbi.nlm.nih.gov/Taxonomy/Browser/wwwtax.cgi?id=1117)] [SET domain-containing protein [Cylindrospermum stagnale PCC](http://www.ncbi.nlm.nih.gov/entrez/query.fcgi?cmd=Retrieve&db=Protein&list_uids=505023598&dopt=GenPept)
522. . [Bacillus coahuilensis](http://www.ncbi.nlm.nih.gov/Taxonomy/Browser/wwwtax.cgi?id=408580) ......................................... 53 [1 hit](http://blast.ncbi.nlm.nih.gov/Blast.cgi?CMD=Get&RID=5Y2VSSZC016&FORMAT_OBJECT=TaxBlast&NCBI_GI=off&DESCRIPTIONS=100&ALIGNMENTS=100&FORMAT_BLOCK_ON_RESPAGE=Top&MASK_COLOR=1&MASK_CHAR=2#408580) [[firmicutes](http://www.ncbi.nlm.nih.gov/Taxonomy/Browser/wwwtax.cgi?id=1239)] [lysine methyltransferase [Bacillus coahuilensis]](http://www.ncbi.nlm.nih.gov/entrez/query.fcgi?cmd=Retrieve&db=Protein&list_uids=497858455&dopt=GenPept)
523. . [Bacillus massiliosenegalensis](http://www.ncbi.nlm.nih.gov/Taxonomy/Browser/wwwtax.cgi?id=1287657) ................................. 54 [1 hit](http://blast.ncbi.nlm.nih.gov/Blast.cgi?CMD=Get&RID=5Y2VSSZC016&FORMAT_OBJECT=TaxBlast&NCBI_GI=off&DESCRIPTIONS=100&ALIGNMENTS=100&FORMAT_BLOCK_ON_RESPAGE=Top&MASK_COLOR=1&MASK_CHAR=2#1287657) [[firmicutes](http://www.ncbi.nlm.nih.gov/Taxonomy/Browser/wwwtax.cgi?id=1239)] [lysine methyltransferase [Bacillus massiliosenegalensis]](http://www.ncbi.nlm.nih.gov/entrez/query.fcgi?cmd=Retrieve&db=Protein&list_uids=517986254&dopt=GenPept)
524. . [Bacillus megaterium WSH-002](http://www.ncbi.nlm.nih.gov/Taxonomy/Browser/wwwtax.cgi?id=1006007) ................................... 52 [2 hits](http://blast.ncbi.nlm.nih.gov/Blast.cgi?CMD=Get&RID=5Y2VSSZC016&FORMAT_OBJECT=TaxBlast&NCBI_GI=off&DESCRIPTIONS=100&ALIGNMENTS=100&FORMAT_BLOCK_ON_RESPAGE=Top&MASK_COLOR=1&MASK_CHAR=2#1006007) [[firmicutes](http://www.ncbi.nlm.nih.gov/Taxonomy/Browser/wwwtax.cgi?id=1239)] [sET domain-containing protein [Bacillus megaterium WSH-002]](http://www.ncbi.nlm.nih.gov/entrez/query.fcgi?cmd=Retrieve&db=Protein&list_uids=384048889&dopt=GenPept)
525. . [Bacillus megaterium DSM 319](http://www.ncbi.nlm.nih.gov/Taxonomy/Browser/wwwtax.cgi?id=592022) ................................... 53 [2 hits](http://blast.ncbi.nlm.nih.gov/Blast.cgi?CMD=Get&RID=5Y2VSSZC016&FORMAT_OBJECT=TaxBlast&NCBI_GI=off&DESCRIPTIONS=100&ALIGNMENTS=100&FORMAT_BLOCK_ON_RESPAGE=Top&MASK_COLOR=1&MASK_CHAR=2#592022) [[firmicutes](http://www.ncbi.nlm.nih.gov/Taxonomy/Browser/wwwtax.cgi?id=1239)] [hypothetical protein BMD_0518 [Bacillus megaterium DSM 319]](http://www.ncbi.nlm.nih.gov/entrez/query.fcgi?cmd=Retrieve&db=Protein&list_uids=295702679&dopt=GenPept)
526. . Bacillus cereus E33L 1 hit [[firmicutes](http://www.ncbi.nlm.nih.gov/Taxonomy/Browser/wwwtax.cgi?id=1239)] SET domain-containing protein YP_086370 [Bacillus cereus E33L]
527. . Bacillus anthracis str. A0248 1 hit [[firmicutes](http://www.ncbi.nlm.nih.gov/Taxonomy/Browser/wwwtax.cgi?id=1239)] SET domain protein YP_002869308 [Bacillus anthracis str. A0248]
528. . Bacillus thuringiensis IBL 4222 1 hit [[firmicutes](http://www.ncbi.nlm.nih.gov/Taxonomy/Browser/wwwtax.cgi?id=1239)] SET domain protein EEN00542 [Bacillus thuringiensis IBL 4222]
529. . [Solibacillus silvestris StLB046](http://www.ncbi.nlm.nih.gov/Taxonomy/Browser/wwwtax.cgi?id=1002809) ............................... 52 [2 hits](http://blast.ncbi.nlm.nih.gov/Blast.cgi?CMD=Get&RID=5Y2VSSZC016&FORMAT_OBJECT=TaxBlast&NCBI_GI=off&DESCRIPTIONS=100&ALIGNMENTS=100&FORMAT_BLOCK_ON_RESPAGE=Top&MASK_COLOR=1&MASK_CHAR=2#1002809) [[firmicutes](http://www.ncbi.nlm.nih.gov/Taxonomy/Browser/wwwtax.cgi?id=1239)] [protein containing SET domain [Solibacillus silvestris StLB](http://www.ncbi.nlm.nih.gov/entrez/query.fcgi?cmd=Retrieve&db=Protein&list_uids=393200745&dopt=GenPept)
530. . [Gemmata obscuriglobus](http://www.ncbi.nlm.nih.gov/Taxonomy/Browser/wwwtax.cgi?id=114) ......................................... 69 [2 hits](http://blast.ncbi.nlm.nih.gov/Blast.cgi?CMD=Get&RID=5Y2VSSZC016&FORMAT_OBJECT=TaxBlast&NCBI_GI=off&DESCRIPTIONS=100&ALIGNMENTS=100&FORMAT_BLOCK_ON_RESPAGE=Top&MASK_COLOR=1&MASK_CHAR=2#114) [[planctomycetes](http://www.ncbi.nlm.nih.gov/Taxonomy/Browser/wwwtax.cgi?id=112)] [hypothetical protein [Gemmata obscuriglobus]](http://www.ncbi.nlm.nih.gov/entrez/query.fcgi?cmd=Retrieve&db=Protein&list_uids=497730542&dopt=GenPept)
531. . Methanoregula boonei ......................................... [1 hit](http://blast.ncbi.nlm.nih.gov/Blast.cgi?CMD=Get&RID=5Y3VDS5A013&FORMAT_OBJECT=TaxBlast&NCBI_GI=off&DESCRIPTIONS=500&ALIGNMENTS=250&FORMAT_BLOCK_ON_RESPAGE=Top&MASK_COLOR=1&MASK_CHAR=2#2209) [[euryarchaeotes](http://www.ncbi.nlm.nih.gov/Taxonomy/Browser/wwwtax.cgi?id=28890)] nuclear protein SET [Methanoregula boonei]
532. . . . . . . [Desulfitobacterium metallireducens](http://www.ncbi.nlm.nih.gov/Taxonomy/Browser/wwwtax.cgi?id=142877) ............................ 58 [1 hit](http://blast.ncbi.nlm.nih.gov/Blast.cgi?CMD=Get&RID=5Y2VSSZC016&FORMAT_OBJECT=TaxBlast&NCBI_GI=off&DESCRIPTIONS=100&ALIGNMENTS=100&FORMAT_BLOCK_ON_RESPAGE=Top&MASK_COLOR=1&MASK_CHAR=2#142877) [[firmicutes](http://www.ncbi.nlm.nih.gov/Taxonomy/Browser/wwwtax.cgi?id=1239)] [lysine methyltransferase [Desulfitobacterium metallireducen](http://www.ncbi.nlm.nih.gov/entrez/query.fcgi?cmd=Retrieve&db=Protein&list_uids=493766347&dopt=GenPept)
533. . . . . . . [Desulfitobacterium metallireducens DSM 15288](http://www.ncbi.nlm.nih.gov/Taxonomy/Browser/wwwtax.cgi?id=871968) .................. 58 [1 hit](http://blast.ncbi.nlm.nih.gov/Blast.cgi?CMD=Get&RID=5Y2VSSZC016&FORMAT_OBJECT=TaxBlast&NCBI_GI=off&DESCRIPTIONS=100&ALIGNMENTS=100&FORMAT_BLOCK_ON_RESPAGE=Top&MASK_COLOR=1&MASK_CHAR=2#871968) [[firmicutes](http://www.ncbi.nlm.nih.gov/Taxonomy/Browser/wwwtax.cgi?id=1239)] [lysine methyltransferase [Desulfitobacterium metallireducen](http://www.ncbi.nlm.nih.gov/entrez/query.fcgi?cmd=Retrieve&db=Protein&list_uids=353550614&dopt=GenPept)

Etc…
